# A Mechanistic Study on the Tautomerism of *H*-Phosphonates, *H*-Phosphinates and Secondary Phosphine Oxides

**DOI:** 10.3390/molecules24213859

**Published:** 2019-10-25

**Authors:** Daniella Vincze, Péter Ábrányi-Balogh, Péter Bagi, György Keglevich

**Affiliations:** 1 Department of Organic Chemistry and Technology, Budapest University of Technology and Economics, 1521 Budapest, Hungary; vincze.daniella@gmail.com (D.V.); pbagi@mail.bme.hu (P.B.); 2 Research Centre for Natural Sciences, Medicinal Chemistry Research Group, 1117 Budapest, Hungary

**Keywords:** *H*-phosphonates, *H*-phosphinates, secondary phosphine oxides, tautomerism, quantum chemical calculations, mechanism

## Abstract

*H*-phosphonates, *H*-phosphinates and secondary phosphine oxides may be preligands, and are important building blocks in the synthesis of pharmaceuticals, pesticides, and *P*-ligands. The prototropic tautomerism influenced by substituent effects plays an important role in the reactivity of these species. The main goal of our research was to study the tautomerism of the >P(O)H reagents by means of computational investigations applying several DFT methods at different levels. We focused on the effect of implicit solvents, and on explaining the observed trends with physical chemical molecular descriptors. In addition, multiple reaction pathways incorporating three *P*-molecules were elucidated for the mechanism of the interconversion.

## 1. Introduction

>P(O)H derivatives may be important preligands in transition metal complexes, and are essential starting materials in the synthesis of bioactive substrates, such as pharmaceuticals, pesticides, and *P*-ligands [1]. *H*-phosphinates, *H*-phosphonates and secondary phosphine oxides show a prototropic tautomeric equilibrium between the tetracoordinated pentavalent *P*-oxide and the trivalent *P*-hydroxy form (a hydrogen atom moves from a P atom to oxygen). Notably, these species have different reactivity, which is influenced by the substituents effect on the dominating tautomer in the equilibrium mixture. For example, it has been suggested that the phosphinous acid tautomer takes a major part in coordinating with transition metal complexes, which is obvious given that the P(III) form is able to donate a lone pair of electrons [2]. In order to understand the behavior of these compounds in organic chemical reactions, it is a key to investigate the interconversion between the two tautomers, and the effect of the surrounding media on the molecules in determining the thermodynamics, kinetics and the mechanism of the tautomeric equilibrium.

Several papers have examined the distribution of the phosphine oxide and phosphinous acid tautomeric forms in different solvents. In most cases, the investigated compounds were substituted with electron withdrawing fluorinated groups, therefore the P(III) form was detectable by spectroscopic methods. In some instances, a solvent-dependent equilibrium was observed between the two tautomeric forms [3,4,5,6], however, the solvents stabilizing the trivalent form usually contain traces of water, thus, the distribution reported might be the consequence of two factors: the properties of the solvent and the water content. This is also supported by the findings of Allefeld who found that the number of H-bond donors affects the equilibrium [6].

Earlier computational studies revealed that the interconversion of one form into the other without any catalysis requires a large investment of energy, around 240–260 kJ mol^–1^ [7,8]. This indicates that the intramolecular tautomerization might not be relevant at room temperature. However, the reactivity of >P(O)H derivatives suggests the presence of both forms in the mixture. To understand this inconsistency, several catalytic approaches were assumed that allowed the presence of both tautomers in reactions performed at room temperature. Previous researchers applied transition metals (e.g., Ru) [7] or water as the catalyst [9], and the P(III) forms were trapped by Lewis acids or by silylation, as well [10]. Moreover, it was found that in a few cases the reactions of *H*-phosphonates and *H*-phosphinates were started with a base catalyzed tautomerization [11,12,13]. Despite the knowledge available on the interconversion of the P(V) and P(III) forms, further exploration and study was required in order to be able to avoid using any catalyst. Accordingly, a dyad form was proposed, where two >P(O)H derivatives form a dimeric complex and switch hydrogens [14,15,16]. Considering this mechanism, DFT (density functional theory) computations were performed for a small number of compounds in the gas phase, and the outcome was explained by the tunneling effect of the protons [14,15,16]. 

Regarding the reactivity of >P(O)H derivatives, the proton exchange between the phosphorus and the oxygen atom is a critical step [17]. The trivalent form is usually more reactive according to its enhanced nucleophilicity and the lower steric hindrance. At the same time, the pentavalent tetracoordinated form is usually the more stable tautomer, dominating by a large excess in the equilibrium mixture [9]. However, it was observed that electron withdrawing groups may increase the stability of the trivalent form [18]. This was demonstrated by the example of species (CF_3_)_2_P(O)H and (C_6_F_5_)_2_P(O)H, where the equilibrium was shifted to the corresponding phosphinous acid forms due to the strong electron withdrawing substituents [3]. The effect of the solvent on the tautomeric equilibrium was also studied, and it was found that while (CF_3_)_2_P(O)H does not exhibit a solvent dependent equilibrium, (C_6_F_5_)_2_P(O)H clearly showed a solvent dependency. This observation was explained by the difference between the zero point energies of the two compounds, but none of other physical chemical parameters were investigated. Among these latter findings, ab initio and free energy perturbation (FEP) calculations were performed in water for the phosphinous acid–phosphine oxide interconversion, but no other solvents were taken into account [19]. Börner et al. investigated the tautomeric equilibria of five secondary phosphine oxides by NMR, IR, X-ray and quantum chemical calculations, and addressed the observed solvent dependency to the hydrogen-bond acceptor properties of the medium [5]. In a recent paper, Keshavarzipour and Tavakol applied high level quantum chemical calculations to investigate various possible pathways, mostly dealing with proton transfer by solvent molecules, particularly one, two or three explicit water or methanol molecules. The work has been done for five phosphinous acids and phosphine oxides. The polarizable continuum model (PCM) solvent effects for three solvents were also computed. It was found that the compounds containing electron withdrawing halogen preferred the P(III) form, and the solvation decreased the rate constants of the tautomeric processes [20]. Summarizing the results of earlier studies, the tautomerization of simple secondary phosphine oxides and phosphine oxide (H_3_PO) itself has already been investigated [5,20,21,22], and the effect of substituents was revealed [9,14]. However, these studies contained a limited number of compounds and solvents, hence a systematic investigation of larger datasets is still missing.

In order to broaden the characterization of the tautomerism of >P(O)H derivatives, and as a continuation of our efforts [23] to obtain mechanistic insights into the reactivity of *P*-species, we performed systematic quantum chemical computations on a series of acyclic >P(O)H derivatives using the Gaussian 09 program package [24] to apply various methods, basis sets, and taking into account the effect of a wide range of solvents. Our intent was to study the effect of these solvents and to explain the observed phenomena with different molecular descriptors as physical chemical parameters.

## 2. Results and Discussion

We aimed to systematically investigate the tautomerization mechanism of twenty chosen *H*-phosphonates, *H*-phosphinates or secondary phosphine oxides (Table 1). The substituents were selected from the electron donating ones (e.g., methyl (**1**), ethyl (**2**), methoxy (**3**), ethoxy (**4**)) to slight or strong electron withdrawing groups (e.g., phenyl (**12**) or trifluoromethyl (**6**), nitromethyl (**11**), 2,4,6-trinitrophenyl (**19**)). The identically substituted derivatives (e.g., bis(carboxymethyl) (**10**)), species with different substituents (e.g., methyl-trifluoromethyl (**7**)) were also investigated.

As the first step of our study, we optimized the geometry and calculated the stability of the P=O and P-OH tautomeric forms for compounds **1**-**20**. The chosen computational methods were the B3LYP functional [25,26] with the 6-31+G(d,p) (**A**) [27], 6-311++G(3df,3pd) (**B**) [28,29] and the cc-pVTZ (**C**) [30] basis sets. Moreover, we applied the B3LYP-D3/6-31+G(d,p) (**D**) [31] and the ωB97XD/6-311++G(3df,3pd) (**E**) [32] methods as well. Due to the large number of computations, we choose relatively inexpensive, but still accurate DFT methods. In order to find the best method, we compared standard hybrid functionals (B3LYP) with different basis sets, the same functional with dispersion correction (B3LYP-D3) and a dispersion model (ωB97XD), as well. The geometries of the molecules were optimized in all cases, and frequency calculations were also performed. The computations were carried out without using a solvent model, or applying the integral equation formalism model (IEFPCM), a variant of the polarizable continuum model [33] taking into account the effect of various solvents. Moreover, in selected cases, the SMD (Solvation Model based on Density) implicit solvent model (Truhlar’s SMD variation of IEFPCM) [34] was also applied, and it was proven that in most cases the results were in good accordance with the IEFPCM, and the trends obtained were independent from the solvent model. For the detailed energetical data, see Tables S1 and S6 in the Supplementary Materials.

As preliminary computations, the energetics for the Y_2_P(O)H ⇄ Y_2_POH equilibrium were determined with method **A** in the gas phase. We wished to characterize the relative stability of the two tautomers by the Gibbs free energy difference. We found that in accord with earlier studies [8], the compounds with electron withdrawing substituents, (Y = F (**5**), CF_3_ (**6**), CO_2_Me (**10**), CH_2_NO_2_ (**11**), 4-NO_2_C_6_H_4_ (**17**), 2,4-(NO_2_)_2_C_6_H_3_ (**18**), 2,4,6-(NO_2_)_3_C_6_H_2_ (**19**) and C_6_F_5_ (**20**) exist under an equilibrium shifted towards the trivalent form, while those with electron donating substituents (Y = Me (**1**), Et (**2**), OMe (**3**), OEt (**4**), Ph (**12**)) have a strong preference towards the pentavalent form (Table 2).

**Figure 1:**
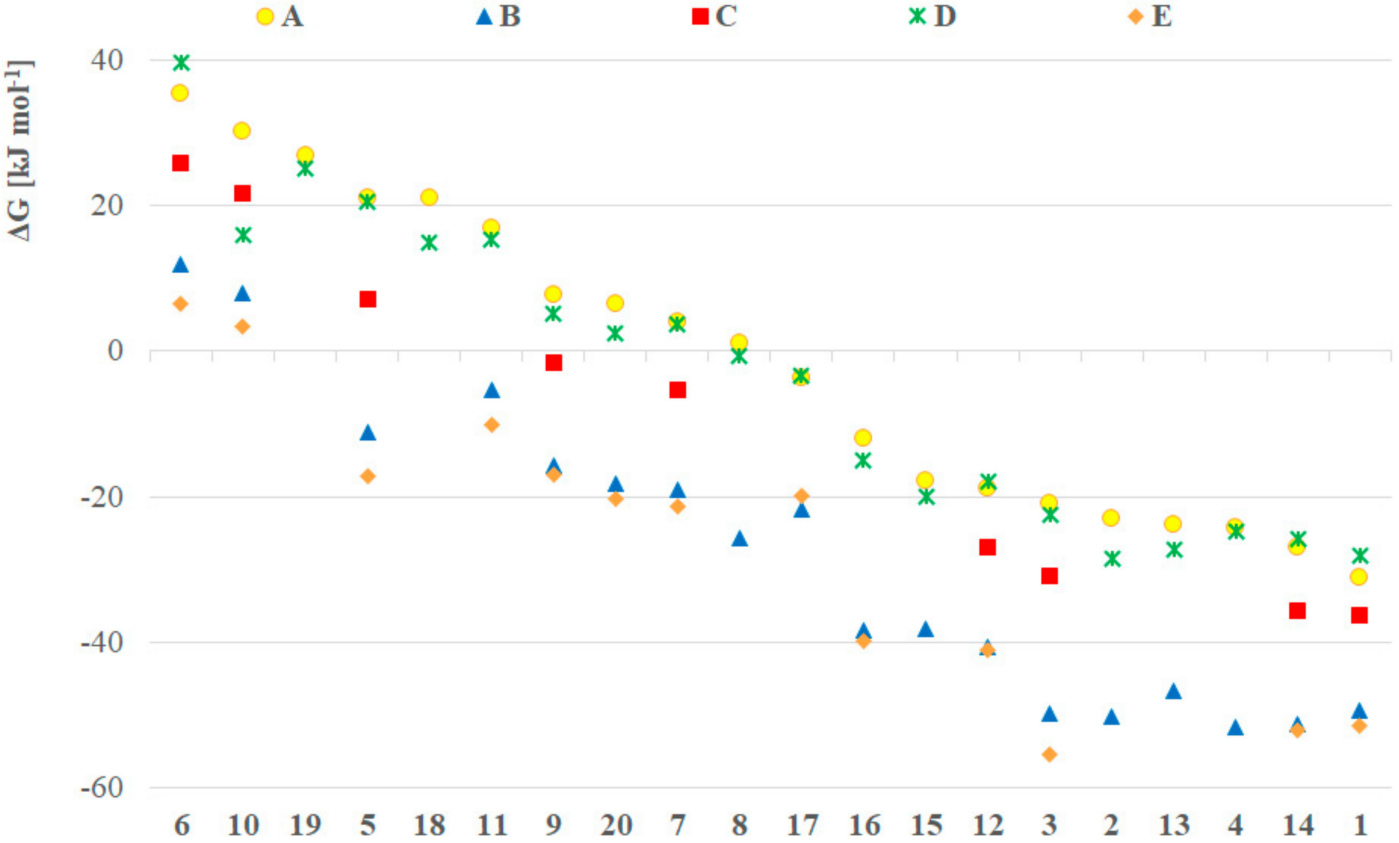
ΔG values for the G_P(V)_–G_P(III)_ tautomeric equilibrium of compounds **1**–**20** calculated by methods **A** (B3LYP/6-31+G(d,p)), **B** (B3LYP/6-311++G(3df,3pd)), **C** (B3LYP/cc-pVTZ), **D** (B3LYP-D3/6-31+G(d,p)), **E** (ωB97XD/6-311++G(3df,3pd)) considering the implicit solvent effect of dichloromethane by all methods. The missing data points could not be computed due to the lack of convergence.

In order to create a more detailed dataset and presumably more accurate results, we performed the computations in dichloromethane as implicit solvent applying all the five methods (**A**–**E**). The Gibbs free energy for all model compounds was determined, and the difference in the G_P(V)_ and G_P(III)_ is depicted in Figure 1. It should be mentioned that the compounds with positive ΔG values, exist under an equilibrium shifted to the P(III) form, while negative ΔG values suggest equilibria shifted towards the pentavalent forms [3]. Using method **A**, in the case of electron withdrawing substituents, like F (**5**), CF_3_ (**6**), CO_2_Me (**10**), nitromethyl (**11**), nitrophenyl (**17**–**19**), the trivalent form was found to be more stable, and the species with more electron donating substituents preferred the P(V) form (Figure 1, yellow dots). This was followed by calculations using larger basis sets (**B**, **C**, Figure 1, blue triangles and red squares, respectively) and methods (**D**, Figure 1, green crosses) (**E**, Figure 1, orange rhombs) taking into account the dispersion. Most of the compounds were more stable in the pentavalent form, only the bis(trifluoromethyl) (**6**) and the bis(carboxymethyl) (**10**) derivatives remained stable as P(III) species. According to our observations, the trend in the stability change was similar for all the methods applied. In particular, methods **A** and **D** produced almost the same results, and similar proximity of the ΔG values were obtained by method **B** and **E**, suggesting that by these type of computations the dispersion is not significant, either by considering it as a correction (**D**) or as a dispersion model (**E**). It should be noted that methods **A** and **D** gave the highest ΔG values, while the cc-pVTZ basis set (**C**) decreased these, and the largest basis sets (**B** and **E**) resulted in the lowest ΔGs. Eventually, the dialkyl and dialkoxy >P(O)H species were found to be more stable in P(V) form, while the species with the aforementioned electron withdrawing substituents are more stable in the P(III) form. One can see in Figure 1 that the Gibbs free energy differences between the different methods were in the same range (20–40 kJ mol^–1^) for each compound. Notably, the results computed with the SMD implicit solvent model did not differ significantly from that computed with the IEFPCM. In particular, the average difference for the models was in the range of −1.1 to 0.0 kJ mol^−1^ with a standard deviation in the range of 1.7 to 3.7 kJ mol^−1^, respectively (Supplementary Table S1 and Figure S1).

**Figure 2:**
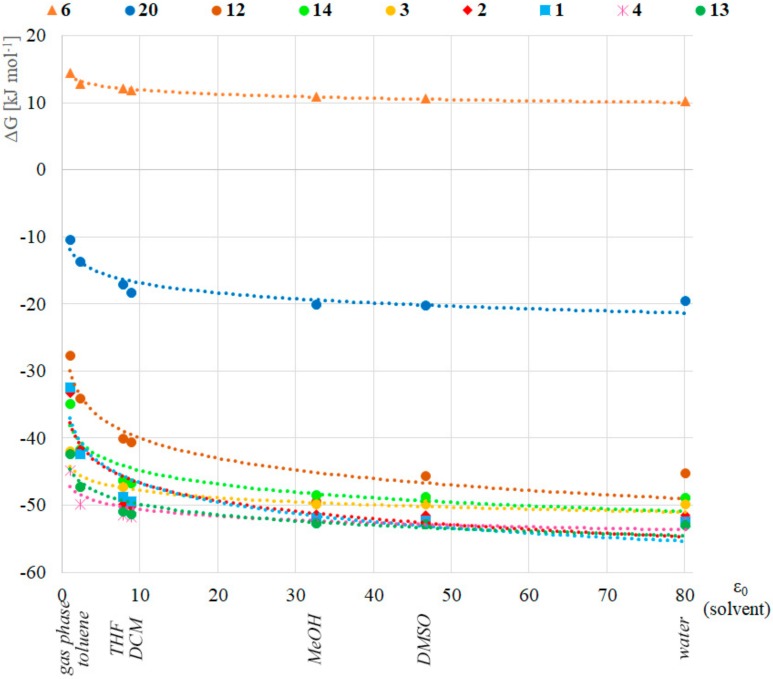
Stability of the P(V) form in various solvents. Each point means the energy difference in Gibbs free energy between the P(V) and P(III) forms. The color of the equation of the logarithmic trendline with the corresponding R^2^ refers to the corresponding symbol.

Next, the effect of different solvents was investigated applying the B3LYP/6-311++G(3df,3pd) (**B**) method and the IEFPCM solvent model for toluene, tetrahydrofuran, dichloromethane, methanol, dimethyl sulfoxide and water (ε_0_ = 2.38, 7.85, 8.93, 32.7, 46.7 and 80.1, respectively) for a selected set of compounds **1**, **2**, **3**, **4**, **6**, **12**, **13**, **14** and **20**. This method was chosen because although it is not the cheapest, it produces the most accurate results for the energetics that were compared in this part of the research. The reference point was the stability value calculated in gas phase (ε_0_ = 1.0). The Gibbs free energy difference between the pentavalent and the trivalent form in the function of the relative permittivity of the solvents is depicted in Figure 2. Interestingly, we found a logarithmic relationship (R^2^ of the fitted trendline is between 0.72 and 0.96, see Table S2 in the Supplementary Materials), namely, the pentavalent form was more stable in solvents with higher relative permittivity (e.g., water, DMSO). The significance of this correlation was investigated with the *t*- and *p*-tests, and one can see that the confidence level is more than 98% in all cases (Table S3 in the Supplementary Materials), when the R value of the logarithmic trendline is used. The largest difference was in the case of dimethyl phosphine oxide (**1**, blue squares in Figure 2) where the stability in water was 20 kJ mol^–1^ higher than in vacuum. The lowest difference (4.3 kJ mol^–1^) was found in the case of bis(trifluoromethyl) phosphine oxide (**6**, orange triangles in Figure 2) suggesting that the P_III_-P_V_ equilibrium does not depend significantly on the solvent of that model compound. It should be noted that this result might explain the solvent independency of compound **6** as reported earlier [3]. Regarding the observations described therein, a comparison of the equilibria for species **6** and **20** suggest that in the case of (C_6_F_5_)_2_P(O)H (**20**), the energy difference between the gas phase and the aqueous solution stability is two times higher (9.1 kJ mol^–1^) according to Figure 2, supporting a higher solvent dependency. The accuracy of the IEFPCM solvent model was tested in this case as well, and compared with the results computed with the SMD implicit solvent model. The average difference for the models was in the range of -1.8 to 1.6 kJ mol^−1^ with a standard deviation in the range of 1.1 to 4.5 kJ mol^−1^, respectively (Supplementary Table S1 and Figure S1).

Taking a closer look at the explanation of this phenomenon, we computed the highest occupied molecular orbital (HOMO) and the lowest unoccupied molecular orbital (LUMO) energies (εHOMO and εLUMO, respectively), and the relative natural atomic charges using the natural bond orbital (NBO) method [35,36] for the compounds investigated. Moreover, we calculated the chemical hardness (H = εHOMO–εLUMO), and the Parr-index (P = EP^2^/2H) as a descriptor for electrophilicity [37]. Plotting the constants from the *y* = *a* ln(*x*) + *b* logarithmic equations in the function of the calculated descriptors, significant correlations (*p* < 0.01) were found (for the descriptors and “*a*”, “*b*” parameters, see Table S2 in the Supplementary Materials). The “*a*” parameter of the logarithmic equation correlated with an R^2^ = 0.44 (confidence level is 94.8%) with the HOMO energy of P(V) tautomers. The “*b*” constant correlated well with the relative charge on the P(V) atom (R^2^ = 0.76, confidence level is 99.8%). Although these correlations are considered as acceptable, and show a trend rather than quantitative correspondence, it seems clear that the change of the stability in solvents with different relative permittivity values might be easily influenced by the descriptors mentioned above because the polarizability of the phosphorus is determined by the charge on the P atom and the HOMO energy of the molecule. In addition, we investigated the dipole moment of the P(III) and P(V) species (for the full dataset, see Table S5 in the Supplementary Materials). It was clearly seen that except for compound **6** with CF_3_ substituents, the dipole moments were larger for the P(V) tautomer than for the P(III). Moreover, the dipole moments are higher with the increase in the relative permittivity of the solvents. These observations support the theory suggesting that the more polar solvents stabilize the more polar tautomeric form.

**Scheme 1:**
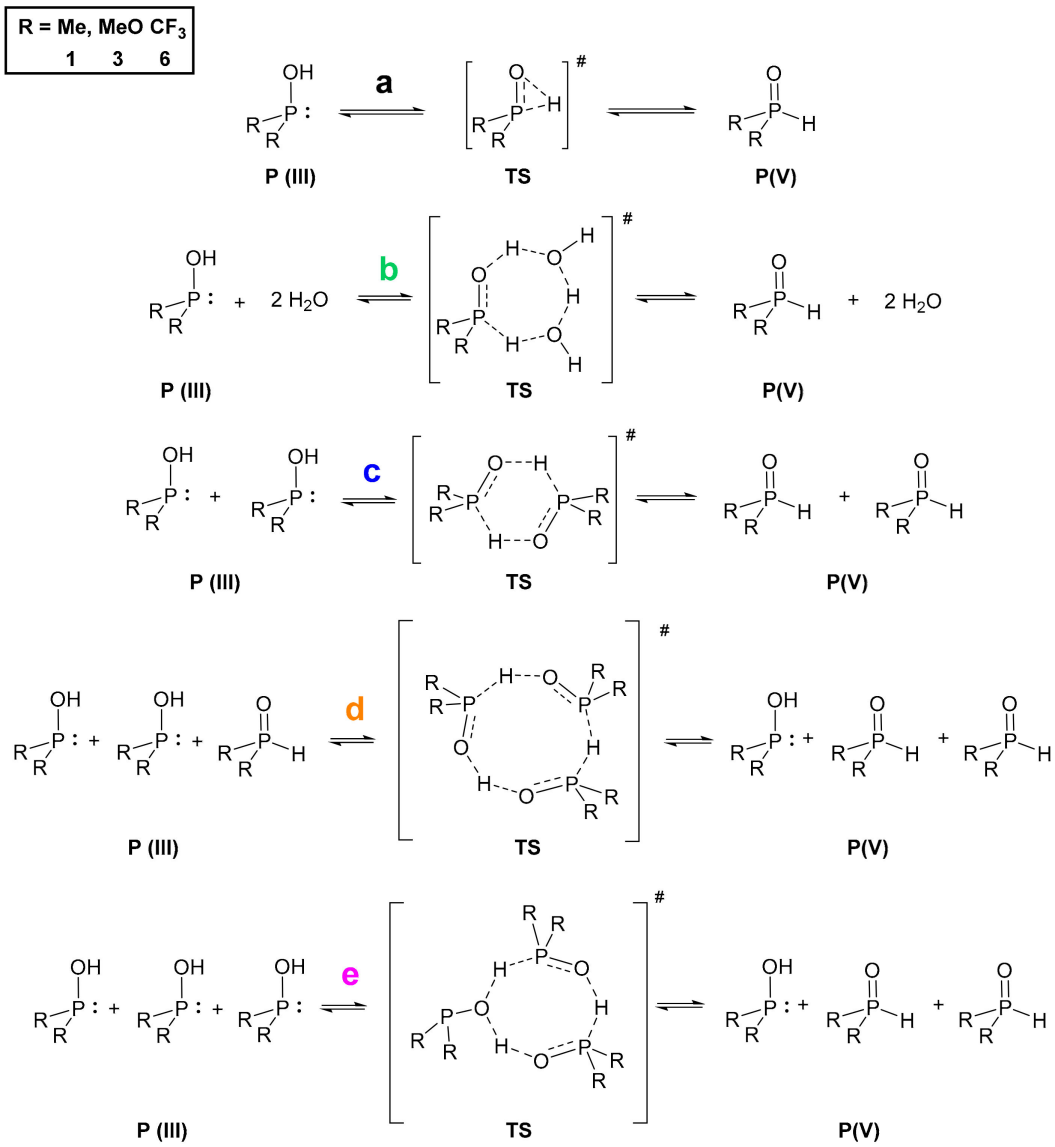
Proposed reaction mechanisms (**a**–**e**) for the tautomeric equilibria of the >P(O)H compounds **1**, **3** and **6**.

**Figure 3:**
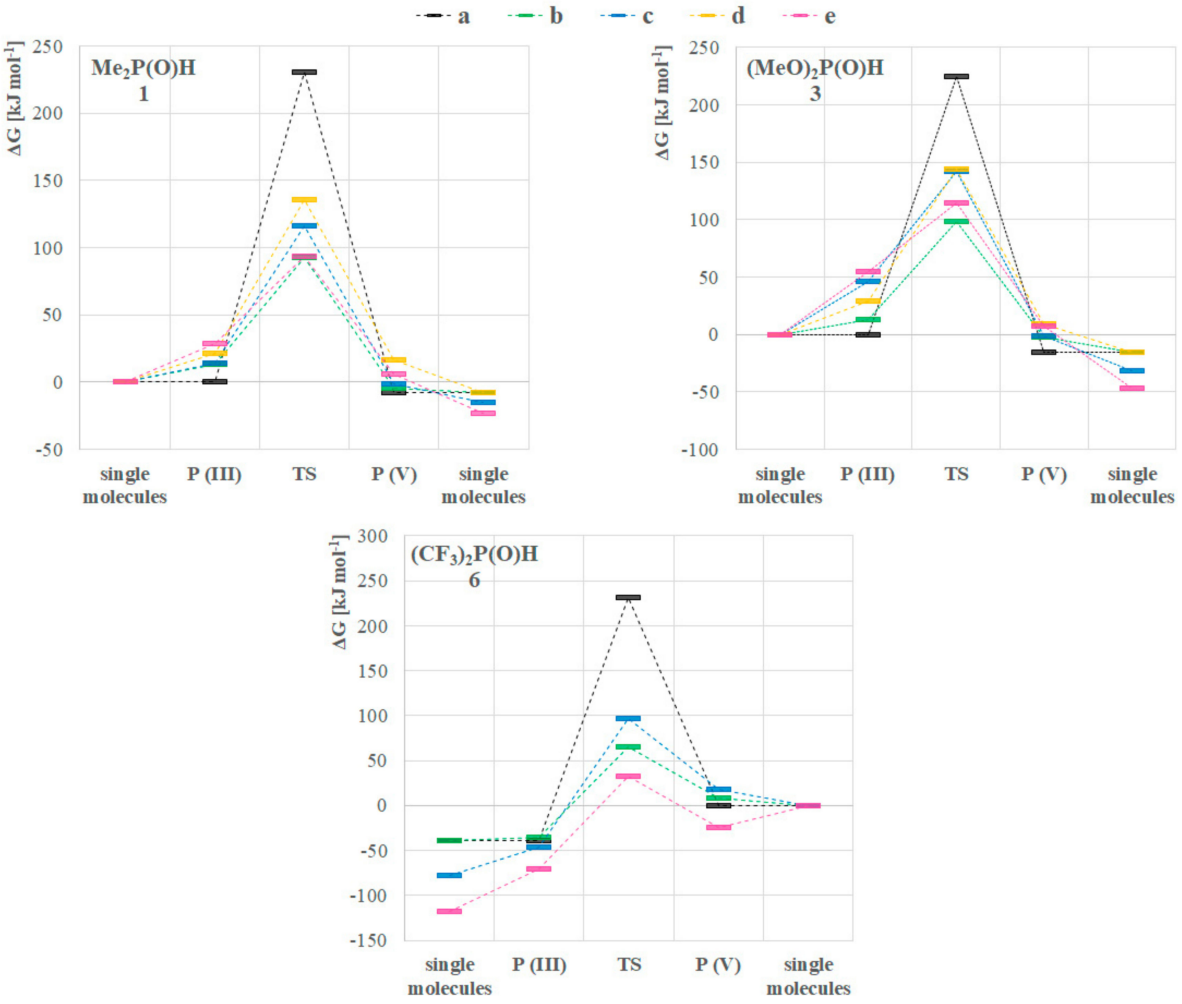
Computed reaction energetics of mechanisms (**a**–**e**) for the tautomeric equilibria of the >P(O)H compounds **1**, **3** and **6** with method **A** in the gas phase.

Turning our attention to the mechanism of the tautomerization, we computed reaction pathways transforming the P(III) species to their pentavalent tautomers for model compounds dimethylphosphine oxide (**1**) and dimethyl phosphite (**3**), and the opposite way for bis(trifluoromethyl)phosphine oxide (**6**). This analysis was based on previous mechanisms proposed in the literature [7,9,14]. We used method **A** in the gas phase, and considered various implicit solvents as well. The Gibbs free energies for the single starting compounds and products were computed, and the reaction energetics were mapped by scanning. The starting and the final reaction complexes, as well as the transition states (TS) were optimized (Scheme 1, Figure 3). The height of the TS discussed is the difference between the Gibbs free energy of the starting single molecules and the computed TS except in one case when the formation of the reaction complex was exothermic. In the case of all three compounds (**1**, **3** and **6**), the more stable state is depicted as the product, namely, the P(V) form for >P(O)H species **1** and **3**, and P(III) for compound **6** (Figure 3). 

We investigated five different pathways as shown in Scheme 1. At first, the intramolecular proton transfer (**a**) from the phosphorus to the oxygen atom was modelled [7,9]. It was found that the activation barrier of this path would be disadvantageous by 230.4 kJ mol^–1^, 224.9 kJ mol^–1^ and 231.7 kJ mol^–1^ for >P(O)H species **1**, **3** and **6,** respectively (Table 3, column **a**). On the contrary, the second option suggested by Janesko et al. containing two water molecules forming a proton transfer system (**b**) [9] lowered the energy of the TS to 92.8 kJ mol^–1^, 98.2 kJ mol^–1^ and 64.7 kJ mol^–1^, respectively (Table 3, column **b**). This significant change in the energetics can be easily explained with the structure of the transition states. In the intramolecular case, the TS contains a strained three-membered structural moiety that is sterically less advantageous than a larger seven-membered proton transfer system. Although the low energy TSs belonging to pathway **b** seemed persuasive, we wished to investigate the option of lowering the energy of the TS in the absence of water as well. One should note that in many cases working under dry conditions is crucial. This led us to model the dimeric pathway (**c**) [14], and two other reaction pathways as well, i.e., the transformation of three molecules in trimer systems (**d** and **e**) to the other tautomeric form. The detailed analysis of these pathways suggested that the dimeric route (**c**) through a six-membered TS requires an activation energy of 115.9 kJ mol^–1^, 141.6 kJ mol^–1^ and 96.9 kJ mol^–1^ for >P(O)H species **1**, **3** and **6,** respectively (Table 3, column **c**). That is higher for all P-reagents than the results obtained for route **b**, but still significantly lower than the intramolecular way (**a**). In the case of the trimeric pathway **d**, the system comprising species **1** and **3** was computed, but possibility **e** was calculated for all the three models (**1**, **3** and **6**). Pathway **d** involved a TS with an activation energy of 135.3 kJ mol^–1^ and 143.5 kJ mol^–1^ for compound **1** and **3**, respectively (Table 3, column **d**). Comparing the formation energies of the single molecules, the reaction complex in route **d** was 7.6 kJ mol^–1^ more favorable than that for route **e** (see Figure 3, and for detailed dataset Supplementary Material). In the case of phosphine **1** and phosphite **3**, the transition via route **e** turned out to be more advantageous than that for route **d**, as they required an energy investment of 93.6 kJ mol^–1^ and 114.9 kJ mol^–1^, respectively, while for phosphine **6** the Gibbs free energy of the trimeric TS (**e**) was found to be 57.2 kJ mol^–1^ (Table 3, column **e**). Notably, this latter case was the only one when the formation of the reaction complex was computed as exothermic. It is worth mentioning that the lowest activation barrier without catalysis was found in route **e**, however the water catalyzed (**b**) turned out to be the lowest among all the five mechanisms for **1** and **3**, and similar for **6**. For the detailed energetical data, see Table S6 in the Supplementary Materials. One should take into account that for pathways **b**–**e**, the corresponding two or three molecules are required to meet in an appropriate orientation. Therefore, in low concentrations these low-energy pathways might be less probable than should be considered by the design phase of reactions with these compounds.

It should be mentioned that the P(III) → P(V) tautomerization of phosphine **1** was exothermic considering all mechanisms. Nevertheless, one can see that the formation of the reaction complexes (P(III) in Figure 3/(**1**)) from single molecules requires energy investment in all cases, in the order of **e** > **d** > **b** > **c**, in accord with the fact that two molecules might encounter easier than three, and two water molecules need less space in the reaction complex than two phosphines. Analyzing the same dataset for phosphite **3** (Figure 3, compound **3**), exothermic pathways can be seen, but the formation of the reaction complexes are endothermic, and route **b** is the less disadvantageous, followed by **d**, **c** and **e**. In the case of the P(V) → P(III) interconversion of phosphine **6** (Figure 3, compound **6**), again an exothermicity can be observed. However, the formation of the P(V) starting reaction complexes show different energetics than for phosphine **1**, namely, in the case of three phosphines (**e**) the formation is exothermic. The order of the activation Gibbs free energies in this case is **c** > **b** > **e**.

**Figure 4:**
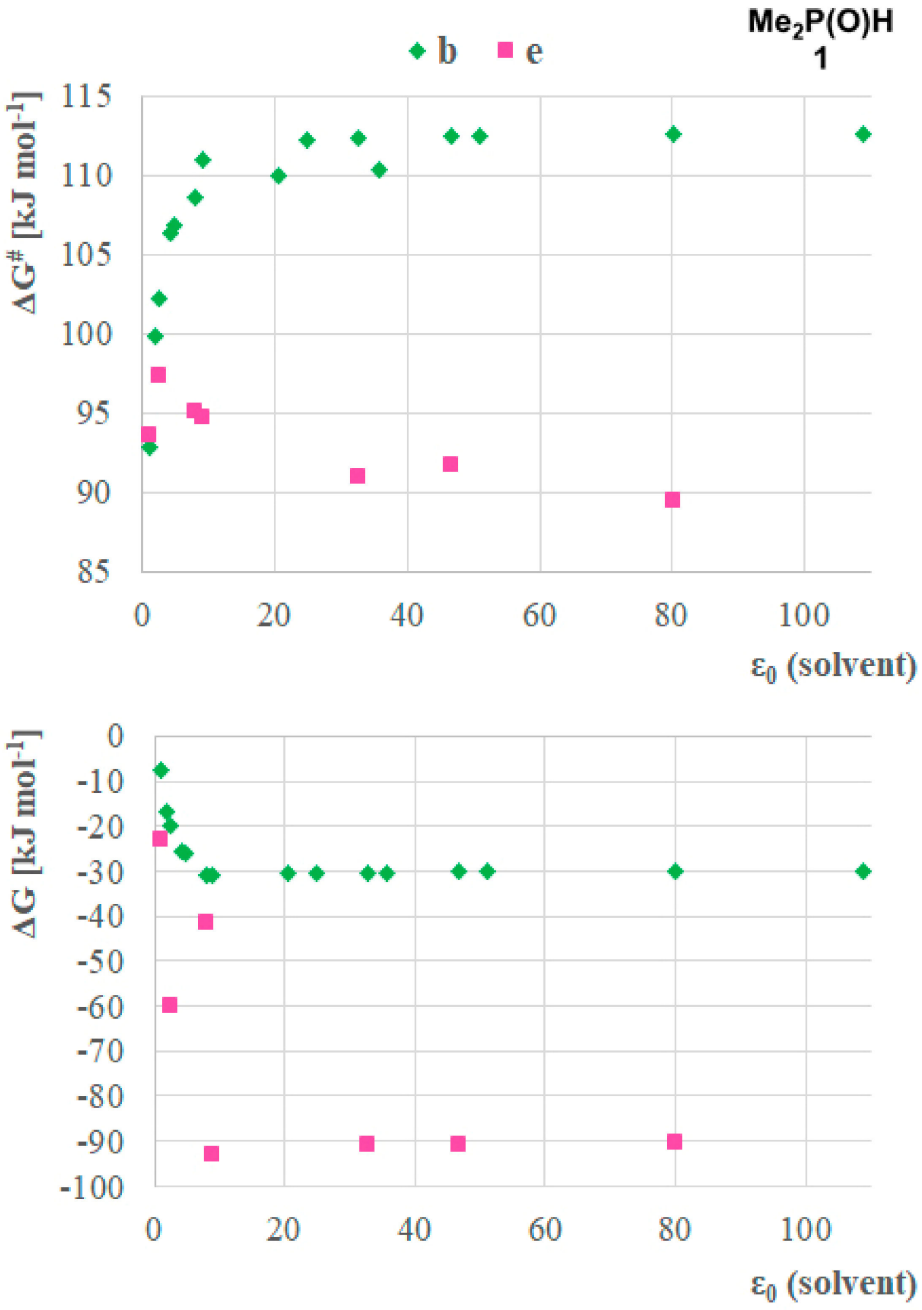
Solvent dependency of the stability (ΔG) of the P(V) product, and the activation barriers (ΔG^#^) for the P(III) → P(V) transformation for the case of dimethyl phosphine oxide (**1**) assuming reaction pathways **b** and **e** calculated by Method **A**.

To further investigate the mechanistic pathways of the tautomerisation, the effect of solvents on the two most advantageous paths (**b** and **e**) was analyzed. Method **A** with the IEFPCM solvent model was applied using a larger set of solvents, including hexane (ε_0_ = 1.88), toluene (ε_0_ = 2.38), diethyl ether (ε_0_ = 4.24), chloroform (ε_0_ = 4.71) tetrahydrofuran (ε_0_ = 7.85), dichloromethane (ε_0_ = 8.93), acetone (ε_0_ = 20.49), ethanol (ε_0_ = 24.85), methanol (ε_0_ = 32.7), acetonitrile (ε_0_ = 35.69), dimethyl sulfoxide (ε_0_ = 46.7), formic acid (ε_0_ = 51.1), water (ε_0_ = 80.1) and formamide (ε_0_ = 108.94) in order to have a set of points on the ε_0_ axis, displaying a higher resolution between the minimal ε_0_ (gas phase) and the maximal ε_0_ (formamide) (Figure 4). The model compound was dimethylphosphine oxide **1**. The analysis of the results obtained showed clear relationships between the stability and the activation of Gibbs free energies in the function of relative permittivity of solvents, indicating the influence of the solvent on the stability of the tautomeric forms and also the energy barrier for the transformation. One can see that the main differences between the ΔGs and ΔG^#^s were found by solvents having ε_0_ < 7.85. The ΔGs and ΔG^#^s were found to be almost the same by higher permittivity values, suggesting the similar ability of those solvents to stabilize the reaction complexes and promote the proton transfer process between the molecules equally. It was shown that similar to the single molecules, the ΔG values decreased with the higher ε_0_ of the solvents. On the contrary, the ΔG^#^ showed different trends for pathways **b** and **e**, and moreover, the solvent dependency of pathway **b** (ΔG^#^s differed in a range of 91.8–117.0 kJ mol^–1^) was higher than that of pathway **e** (ΔG^#^s differed in the range of 89.5–97.3 kJ mol^–1^). These might indicate the influence of a higher entropic factor and the complexity of the formation of **1** + 2H_2_O complex. Otherwise, the difference between the shape of the curves shows that in the case of the two accompanying water molecules the higher ε_0_ results in larger ΔG^#^, while for pathway **b** a decrease in the activation barrier can be seen. This phenomenon might be explained by investigating the interactions of the members of the proton transfer system. The hydrogen atoms of the water molecules might have stronger interactions with the solvent molecules, which might be disadvantageous for the proton transfer system while the larger phosphines are more shielded from the solvent molecules, thus the proton transfer system is not influenced, and the stability of the phosphines in the corresponding solvent determines the height of the activation barrier.

## 3. Conclusions

The prototropic tautomerism of *H*-phosphonates, *H*-phosphinates and secondary phosphine oxides was investigated by quantum chemical computations. It was shown that the stability of the P(III) and P(V) tautomers is highly influenced by the nature of the substituents. The P-reagents with electron donating substituents exist in an equilibrium shifted towards the pentavalent form, while those with electron withdrawing substituents have a strong preference towards the trivalent form. Moreover, a logarithmic relationship was found between this stability and the relative permittivity of the solvent medium. It means that the pentavalent form is more stable in solvents that have higher relative permittivity. This was supported by trends found with physical chemical descriptors, particularly by the corresponding HOMO energies of the molecules and the relative charges on the P atom correlating significantly with the parameters of the logarithmic equation. To investigate the possible mechanistic pathways of the tautomerization, the reaction energetics were mapped and a solvent effect was found, which was different in the presence and in the absence of water. The results suggested that the activation Gibbs free energy of the tautomerization is lower in solvents with higher relative permittivity in the case of anhydrous systems, where it is influenced mostly by the stability of the P-reagents, while it may show the opposite trend in the presence of water. Obviously, these theoretical findings should be evaluated in the future by experiments as well. Setting up the necessary analytics could be our research direction in the future.

## Figures and Tables

**Table 1:**
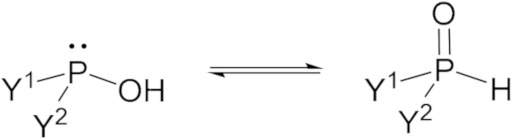
The investigated >P(O)H derivatives (**1**–**20**).

	**1**	**2**	**3**	**4**	**5**	**6**	**7**	**8**	**9**	**10**
**Y^1^**	Me	Et	OMe	OEt	F	CF_3_	CF_3_	CF_3_	CF_3_	CO_2_Me
**Y^2^**	Me	Et	OMe	OEt	F	CF_3_	Me	OMe	Ph	CO_2_Me
	**11**	**12**	**13**	**14**	**15**	**16**	**17**	**18**	**19**	**20**
**Y^1^**	CH_2_NO_2_	Ph	Ph	Ph	Ph	4-F-C_6_H_4_	4-NO_2_-C_6_H_4_	2,4-(NO_2_)_2_-C_6_H_3_	2,4,6-(NO_2_)_3_-C_6_H_2_	C_6_F_5_
**Y^2^**	CH_2_NO_2_	Ph	*t*-Bu	OEt	2-Me-C_6_H_4_	4-F-C_6_H_4_	4-NO_2_-C_6_H_4_	2,4-(NO_2_)_2_-C_6_H_3_	2,4,6-(NO_2_)_3_-C_6_H_2_	C_6_F_5_

**Table 2:** Gibbs free energy values obtained (ΔG) for the tautomeric equilibrium of compounds **1**–**20** calculated by method **A** in the gas phase.

No.	Compound	ΔG [kJ mol^–1^]	No.	Compound	ΔG [kJ mol^–1^]
**1**	Me_2_P(O)H	−7.6	**11**	(CH_2_NO_2_)_2_P(O)H	31.7
**2**	Et_2_P(O)H	−8.9	**12**	Ph_2_P(O)H	−4.0
**3**	(MeO)_2_P(O)H	−15.6	**13**	Ph(*t*-Bu)P(O)H	−17.2
**4**	(EtO)_2_P(O)H	−17.6	**14**	Ph(EtO)P(O)H	−11.3
**5**	F_2_P(O)H	27.3	**15**	Ph(2-MeC_6_H_4_)P(O)H	−7.0
**6**	(CF_3_)_2_P(O)H	39.1	**16**	(4-FC_6_H_4_)_2_P(O)H	−2.7
**7**	CF_3_(Me)P(O)H	16.5	**17**	(4-NO_2_C_6_H_4_)_2_P(O)H	8.4
**8**	CF_3_(MeO)P(O)H	4.9	**18**	[2,4-(NO_2_)_2_C_6_H_3_]_2_P(O)H	25.2
**9**	CF_3_(Ph)P(O)H	16.2	**19**	[2,4,6-(NO_2_)_3_C_6_H_2_]_2_P(O)H	36.1
**10**	(MeO_2_C)_2_P(O)H	31.9	**20**	(C_6_F_5_)_2_P(O)H	15.0

**Table 3:** ΔG^#^ Gibbs free energy values for transition states [kJ mol^–1^], calculated for compounds **1**, **3** and **6** considering reaction pathways **a**–**e** with Method **A** in the gas phase.

Compound	ΔG ^#^				
Pathway	a	b	c	d	e
**1**	230.4	92.8	115.9	135.3	93.6
**3**	224.9	98.2	141.6	143.5	114.9
**6**	231.7	64.7	96.9	n.d.	57.2 *

* the ΔG ^#^ value was computed from the P(V) reaction complex; n.d. not determined due to the lack of convergence.

## Supplementary Materials

The following are available online at https://www.mdpi.com/1420-3049/24/21/3859/s1, Computational protocols, coordinates and energetics of all the computed molecules, physical chemical descriptors, logarithmic parameters, Pearson correlation R^2^s.
