# Supplementary material for: A Mechanistic Study on the Tautomerism of H-Phosphonates, H-Phosphinates and Secondary Phosphine Oxides
Source: Molecules. 2019 Oct 25;24(21):3859. doi: 10.3390/molecules24213859 (PMC6864784; doi:10.3390/molecules24213859)
Supplement: Supplementary file 1 [file molecules-24-03859-s001.pdf]

A Mechanistic Study on the Tautomerism of *H*-Phosphonates, *H*-Phosphinates and Secondary Phosphine Oxides

Daniella Vincze[1], Péter Ábrányi-Balogh[1,2,], Péter Bagi[1], and György Keglevich[1,]

[1] Department of Organic Chemistry and Technology, Budapest University of Technology and Economics, 1521 Budapest, Hungary
[2] Hungarian Academy of Sciences, Research Centre for Natural Sciences, Medicinal Chemistry Research Group, 1117 Budapest, Hungary

Supplementary Material

Table of contents:

Computational methods **3.**

Table S1. Computed energies (E), zero point energies, internal energies (U), enthalpies (H) and Gibbs free energies (G) given in Hartree as well as entropies (S) given in J mol^{-1} K^{-1} [at method A-E for molecules **1-20** in gas phase and also in solvent models]. **3–16**.

Figure S1. ΔG values for the $G_{P(V)}$–$G_{P(III)}$ tautomeric equilibrium of compounds 1–20 calculated by methods A (B3LYP/6-31+G(d,p)), B (B3LYP/6-311++G(3df,3pd)), C (B3LYP/cc-pVTZ), D (B3LYP-D3/6-31+G(d,p)), E (ωB97XD/6-311++G(3df,3pd)) considering the SMD implicit solvent effect of DCM by all methods. **16.**

Table S2. Physical chemical descriptors of selected compounds with the "*a*" and "*b*" constants of the logarithmic equations ($y = a \ln(x) + b$) seen on Fig 3 and the corresponding Pearson correlation R^2. **16-17.**

Figure S2. Stability of the P(V) form in various SMD implicit solvent models. Each point means the energy difference in Gibbs free energy between the P(V) and P(III) forms. **17.**

Table S3. Results of the t- and p-tests together with the confidence level. **17.**

Table S4. Results of correlating the "*a*" and "*b*" constants with different descriptors. **17-18.**

Table S5. Computed dipole moments (method B) in different solvents given in Debye. **18.**

Table S6. Computed energies for the investigations of mechanistic pathways: (E), zero point energies, internal energies (U), enthalpies (H) and Gibbs free energies (G) given in Hartree as

well as entropies (S) given in J mol^{-1} K^{-1} [at method A for molecules **1**, **3** and **6** in gas phase and also in solvent models]. **18–21.**

XYZ coordinates of computed species **21–133.**

Supplementary references **134.**

Computational methods

The B3LYP functional [S1,S2] with the 6-31+G(d,p) [S3] (A), 6-311G++(3df,3pd) (B) [S4,S5] and the cc-pVTZ (C) [S6] basis sets as well as the B3LYP-D3/6-31+G(d,p) (D) [S7] and the ωB97XD/6-311++G(3df,3pd) (D) [S8] methods were used under the Gaussian09 program package [S9]. The geometries of the molecules were optimized in all cases, and frequency calculations were also performed that resulted in the energy values presented in Table S1 and used for the figures of the manuscript. The computations were carried out without using a solvent model, or applying the IEFPCM [S10] and SMD [S11] implicit models taking into account the effect of various solvents. The solution phase Gibbs free energies were obtained by frequency calculations as well. Natural Bond Orbital (NBO) full population analysis was also performed [S12,S13]. The H, G and S values obtained are given at standard conditions, the corrected total energies of the molecules were taken into account. Entropic and thermal corrections are evaluated for isolated molecules using standard rigid rotor harmonic oscillator approximations. (Put another way, the Gibbs free energy is taken as the "sum of electronic and thermal free energies" printed in a Gaussian 09 vibrational frequency calculation). Standard state correction was taken into account, although there was no reaction occurring with the change of the number of molecules. The reaction pathways and energetics were calculated by scanning the corresponding coordinates (atoms) set in the redundant coordinate editor of Gauss View (opt = modredundant keyword). The transition states were optimized with the QST3 or the TS (berny) method. Transition states were identified by having one imaginary frequency in the Hessian matrix, and IRC calculations [S14] were performed in order to prove that the transition states connect two corresponding minima. All the geometries and transition states were optimized and frequency calculations were made to assure that the structures are in a local minimum or in a saddle point, respectively. The reported ΔG values are the differences of the actual sum of Gibbs free energy between the starting structures (single molecules, considered as infinite distance from each other) and the corresponding reaction complexes, transition states, product complexes and single molecules of the latter.

Table S1. Computed energies (E), zero point energies, internal energies (U), enthalpies (H) and Gibbs free energies (G) given in Hartree as well as entropies (S) given in J mol^{-1} K^{-1} [at method **A-E** for molecules **1-20** in gas phase and also in solvent models].

Name	E	ZPE	U	H	G	S
1_(P_III)_method_A.log	-497.0412	-496.9520	-496.9455	-496.9446	-496.9811	76.854
1_(P_III)_method_A_DCM.log	-497.0463	-496.9574	-496.9508	-496.9499	-496.9868	77.572
1_(P_III)_method_A_DCM_smd.log	-497.0485	-496.9597	-496.9531	-496.9522	-496.9890	77.420
1_(P_III)_method_B.log	-497.1270	-497.0381	-497.0317	-497.0307	-497.0671	76.670
1_(P_III)_method_B_DCM_smd.log	-497.1339	-497.0454	-497.0389	-497.0379	-497.0747	77.273
1_(P_III)_method_B_DKM.log	-497.1314	-497.0429	-497.0363	-497.0354	-497.0722	77.543
1_(P_III)_method_B_DMSO.log	-497.1322	-497.0437	-497.0371	-497.0361	-497.0730	77.600
1_(P_III)_method_B_DMSO_smd.log	-497.1320	-497.0434	-497.0369	-497.0359	-497.0726	77.114
1_(P_III)_method_B_MeOH.log	-497.1321	-497.0436	-497.0370	-497.0361	-497.0729	77.605
1_(P_III)_method_B_MeOH_smd.log	-497.1355	-497.0471	-497.0405	-497.0396	-497.0766	78.021
1_(P_III)_method_B_THF.log	-497.1313	-497.0427	-497.0361	-497.0352	-497.0720	77.510
1_(P_III)_method_B_THF_smd.log	-497.1323	-497.0437	-497.0372	-497.0362	-497.0728	76.937

1_(P_III)_method_B_toluene.log	-497.1294	-497.0407	-497.0342	-497.0332	-497.0699	77.080
1_(P_III)_method_B_toluene_smd.log	-497.1306	-497.0417	-497.0352	-497.0343	-497.0707	76.676
1_(P_III)_method_B_water.log	-497.1323	-497.0437	-497.0372	-497.0362	-497.0731	77.593
1_(P_III)_method_B_water_smd.log	-497.1316	-497.0432	-497.0366	-497.0356	-497.0729	78.475
1_(P_III)_method_C_DCM.log	-497.1343	-497.0459	-497.0393	-497.0383	-497.0754	78.010
1_(P_III)_method_C_DCM_smd.log	-497.1364	-497.0481	-497.0415	-497.0405	-497.0775	77.914
1_(P_III)_method_D_DCM.log	-497.0601	-496.9713	-496.9647	-496.9637	-497.0006	77.648
1_(P_III)_method_E_DCM.log	-497.0560	-496.9664	-496.9600	-496.9590	-496.9955	76.879
1_(P_III)_method_E_DCM_smd.log	-497.0583	-496.9688	-496.9624	-496.9614	-496.9978	76.631
1_(P_V)_method_A.log	-497.0441	-496.9553	-496.9494	-496.9485	-496.9840	74.720
1_(P_V)_method_A_DCM.log	-497.0566	-496.9687	-496.9623	-496.9614	-496.9986	78.302
1_(P_V)_method_A_DCM_smd.log	-497.0582	-496.9696	-496.9637	-496.9628	-496.9982	74.461
1_(P_V)_method_B.log	-497.1394	-497.0509	-497.0450	-497.0441	-497.0795	74.541
1_(P_V)_method_B_DCM_smd.log	-497.1524	-497.0641	-497.0582	-497.0572	-497.0927	74.516
1_(P_V)_method_B_DKM.log	-497.1507	-497.0623	-497.0564	-497.0555	-497.0911	74.881
1_(P_V)_method_B_DMSO.log	-497.1527	-497.0643	-497.0584	-497.0574	-497.0929	74.681
1_(P_V)_method_B_DMSO_smd.log	-497.1522	-497.0638	-497.0579	-497.0570	-497.0923	74.220
1_(P_V)_method_B_MeOH.log	-497.1525	-497.0641	-497.0582	-497.0572	-497.0927	74.731
1_(P_V)_method_B_MeOH_smd.log	-497.1583	-497.0698	-497.0640	-497.0630	-497.0983	74.170
1_(P_V)_method_B_THF.log	-497.1503	-497.0619	-497.0560	-497.0550	-497.0906	74.894
1_(P_V)_method_B_THF_smd.log	-497.1509	-497.0624	-497.0566	-497.0556	-497.0909	74.251
1_(P_V)_method_B_toluene.log	-497.1456	-497.0572	-497.0513	-497.0503	-497.0860	75.026
1_(P_V)_method_B_toluene_smd.log	-497.1464	-497.0579	-497.0520	-497.0511	-497.0864	74.284
1_(P_V)_method_B_water.log	-497.1529	-497.0645	-497.0586	-497.0576	-497.0931	74.595
1_(P_V)_method_B_water_smd.log	-497.1547	-497.0662	-497.0604	-497.0595	-497.0948	74.237
1_(P_V)_method_C_DCM.log	-497.1480	-497.0601	-497.0540	-497.0531	-497.0892	75.978
1_(P_V)_method_C_DCM_smd.log	-497.1496	-497.0613	-497.0555	-497.0545	-497.0899	74.458
1_(P_V)_method_D_DCM.log	-497.0710	-496.9824	-496.9764	-496.9755	-497.0113	75.439
1_(P_V)_method_E_DCM.log	-497.0761	-496.9867	-496.9810	-496.9800	-497.0152	73.935
1_(P_V)_method_E_DCM_smd.log	-497.0775	-496.9883	-496.9826	-496.9816	-497.0168	73.912
2_(P_III)_method_A.log	-575.6729	-575.5263	-575.5171	-575.5162	-575.5600	92.261
2_(P_III)_method_A_DCM.log	-575.6775	-575.5314	-575.5221	-575.5211	-575.5652	92.748
2_(P_III)_method_A_DCM_smd.log	-575.6826	-575.5367	-575.5274	-575.5265	-575.5707	93.105
2_(P_III)_method_B.log	-575.7782	-575.6320	-575.6228	-575.6219	-575.6656	92.092
2_(P_III)_method_B_DCM.log	-575.7822	-575.6365	-575.6272	-575.6262	-575.6703	92.727
2_(P_III)_method_B_DCM_smd.log	-575.7876	-575.6419	-575.6328	-575.6318	-575.6753	91.523
2_(P_III)_method_B_DMSO.log	-575.7830	-575.6373	-575.6280	-575.6270	-575.6711	92.841
2_(P_III)_method_B_DMSO_smd.log	-575.7844	-575.6388	-575.6295	-575.6286	-575.6726	92.552
2_(P_III)_method_B_MeOH.log	-575.7829	-575.6372	-575.6279	-575.6269	-575.6710	92.833
2_(P_III)_method_B_MeOH_smd.log	-575.7887	-575.6433	-575.6339	-575.6330	-575.6772	93.042
2_(P_III)_method_B_THF.log	-575.7821	-575.6363	-575.6270	-575.6261	-575.6701	92.694
2_(P_III)_method_B_THF_smd.log	-575.7855	-575.6398	-575.6306	-575.6296	-575.6735	92.361
2_(P_III)_method_B_toluene.log	-575.7804	-575.6344	-575.6251	-575.6242	-575.6681	92.370
2_(P_III)_method_B_toluene_smd.log	-575.7845	-575.6384	-575.6292	-575.6283	-575.6721	92.138
2_(P_III)_method_B_water.log	-575.7830	-575.6373	-575.6280	-575.6271	-575.6712	92.847

2_(P_III)_method_B_water_smd.log	-575.7824	-575.6369	-575.6275	-575.6266	-575.6711	93.668
2_(P_III)_method_D_DCM.log	-575.7020	-575.5556	-575.5464	-575.5454	-575.5893	92.383
2_(P_V)_method_A.log	-575.6760	-575.5300	-575.5213	-575.5204	-575.5634	90.592
2_(P_V)_method_A_DCM.log	-575.6875	-575.5416	-575.5338	-575.5328	-575.5740	86.691
2_(P_V)_method_A_DCM_smd.log	-575.6921	-575.5463	-575.5376	-575.5367	-575.5797	90.487
2_(P_V)_method_B.log	-575.7907	-575.6450	-575.6363	-575.6354	-575.6783	90.300
2_(P_V)_method_B_DCM.log	-575.8010	-575.6554	-575.6467	-575.6457	-575.6895	92.072
2_(P_V)_method_B_DCM_smd.log	-575.8057	-575.6602	-575.6516	-575.6507	-575.6931	89.361
2_(P_V)_method_B_DMSO.log	-575.8029	-575.6572	-575.6486	-575.6476	-575.6908	90.742
2_(P_V)_method_B_DMSO_smd.log	-575.8039	-575.6584	-575.6498	-575.6488	-575.6917	90.245
2_(P_V)_method_B_MeOH.log	-575.8027	-575.6570	-575.6484	-575.6474	-575.6906	90.914
2_(P_V)_method_B_MeOH_smd.log	-575.8111	-575.6655	-575.6569	-575.6560	-575.6989	90.377
2_(P_V)_method_B_THF.log	-575.8005	-575.6550	-575.6462	-575.6453	-575.6891	92.202
2_(P_V)_method_B_THF_smd.log	-575.8036	-575.6581	-575.6495	-575.6485	-575.6914	90.366
2_(P_V)_method_B_toluene.log	-575.7962	-575.6504	-575.6418	-575.6408	-575.6840	90.793
2_(P_V)_method_B_toluene_smd.log	-575.8001	-575.6544	-575.6458	-575.6448	-575.6878	90.407
2_(P_V)_method_B_water.log	-575.8031	-575.6574	-575.6488	-575.6478	-575.6909	90.576
2_(P_V)_method_B_water_smd.log	-575.8052	-575.6596	-575.6510	-575.6500	-575.6929	90.228
2_(P_V)_method_D_DCM.log	-575.7128	-575.5665	-575.5579	-575.5569	-575.6002	91.073
3_(P_III)_method_A.log	-647.5086	-647.4085	-647.4000	-647.3990	-647.4417	89.857
3_(P_III)_method_A_DCM.log	-647.5152	-647.4156	-647.4070	-647.4060	-647.4491	90.641
3_(P_III)_method_A_DCM_smd.log	-647.5169	-647.4175	-647.4088	-647.4078	-647.4509	90.666
3_(P_III)_method_B.log	-647.6584	-647.5584	-647.5499	-647.5489	-647.5916	89.945
3_(P_III)_method_B_DCM.log	-647.6640	-647.5644	-647.5558	-647.5548	-647.5979	90.563
3_(P_III)_method_B_DCM_smd.log	-647.6660	-647.5664	-647.5578	-647.5569	-647.5996	89.948
3_(P_III)_method_B_DMSO.log	-647.6650	-647.5655	-647.5568	-647.5559	-647.5990	90.765
3_(P_III)_method_B_DMSO_smd.log	-647.6650	-647.5657	-647.5569	-647.5560	-647.5995	91.529
3_(P_III)_method_B_MeOH.log	-647.6649	-647.5654	-647.5567	-647.5558	-647.5989	90.740
3_(P_III)_method_B_MeOH_smd.log	-647.6681	-647.5686	-647.5600	-647.5591	-647.6022	90.720
3_(P_III)_method_B_THF.log	-647.6638	-647.5642	-647.5555	-647.5546	-647.5976	90.530
3_(P_III)_method_B_THF_smd.log	-647.6650	-647.5655	-647.5568	-647.5559	-647.5993	91.446
3_(P_III)_method_B_toluene.log	-647.6614	-647.5616	-647.5530	-647.5521	-647.5950	90.278
3_(P_III)_method_B_toluene_smd.log	-647.6625	-647.5628	-647.5541	-647.5532	-647.5963	90.735
3_(P_III)_method_B_water.log	-647.6651	-647.5656	-647.5569	-647.5560	-647.5991	90.789
3_(P_III)_method_B_water_smd.log	-647.6644	-647.5649	-647.5563	-647.5553	-647.5985	90.918
3_(P_III)_method_C_DCM.log	-647.6677	-647.5683	-647.5597	-647.5587	-647.6018	90.700
3_(P_III)_method_C_DCM_smd.log	-647.6694	-647.5702	-647.5615	-647.5606	-647.6039	91.160
3_(P_III)_method_D_DCM.log	-647.5319	-647.4323	-647.4236	-647.4227	-647.4656	90.435
3_(P_III)_method_E_DCM.log	-647.5385	-647.4372	-647.4289	-647.4279	-647.4701	88.685
3_(P_III)_method_E_DCM_smd.log	-647.5402	-647.4390	-647.4307	-647.4298	-647.4718	88.344
3_(P_V)_method_A.log	-647.5143	-647.4141	-647.4059	-647.4049	-647.4477	90.026
3_(P_V)_method_A_DCM.log	-647.5236	-647.4235	-647.4153	-647.4144	-647.4570	89.750
3_(P_V)_method_A_DCM_smd.log	-647.5251	-647.4251	-647.4169	-647.4160	-647.4587	89.878
3_(P_V)_method_B.log	-647.6746	-647.5743	-647.5661	-647.5652	-647.6080	90.061
3_(P_V)_method_B_DCM.log	-647.6830	-647.5829	-647.5747	-647.5738	-647.6166	90.049

3_(P_V)_method_B_DCM_smd.log	-647.6845	-647.5840	-647.5761	-647.5752	-647.6167	87.460
3_(P_V)_method_B_DMSO.log	-647.6844	-647.5843	-647.5762	-647.5752	-647.6179	89.957
3_(P_V)_method_B_DMSO_smd.log	-647.6849	-647.5848	-647.5767	-647.5757	-647.6185	90.033
3_(P_V)_method_B_MeOH.log	-647.6843	-647.5842	-647.5760	-647.5751	-647.6178	89.965
3_(P_V)_method_B_MeOH_smd.log	-647.6875	-647.5876	-647.5794	-647.5785	-647.6215	90.537
3_(P_V)_method_B_THF.log	-647.6827	-647.5826	-647.5744	-647.5734	-647.6162	90.073
3_(P_V)_method_B_THF_smd.log	-647.6841	-647.5840	-647.5758	-647.5749	-647.6177	90.218
3_(P_V)_method_B_toluene.log	-647.6792	-647.5791	-647.5708	-647.5699	-647.6128	90.361
3_(P_V)_method_B_toluene_smd.log	-647.6804	-647.5801	-647.5719	-647.5710	-647.6137	89.910
3_(P_V)_method_B_water.log	-647.6846	-647.5845	-647.5763	-647.5754	-647.6181	89.949
3_(P_V)_method_B_water_smd.log	-647.6840	-647.5840	-647.5758	-647.5748	-647.6178	90.475
3_(P_V)_method_C_DCM.log	-647.6795	-647.5798	-647.5716	-647.5706	-647.6136	90.420
3_(P_V)_method_C_DCM_smd.log	-647.6808	-647.5811	-647.5729	-647.5720	-647.6148	90.012
3_(P_V)_method_D_DCM.log	-647.5408	-647.4406	-647.4324	-647.4315	-647.4742	89.909
3_(P_V)_method_E_DCM.log	-647.5591	-647.4578	-647.4497	-647.4487	-647.4912	89.366
3_(P_V)_method_E_DCM_smd.log	-647.5602	-647.4589	-647.4509	-647.4500	-647.4921	88.697
4_(P_III)_method_A.log	-726.1543	-725.9977	-725.9866	-725.9856	-726.0355	104.851
4_(P_III)_method_A_DCM.log	-726.1609	-726.0047	-725.9936	-725.9926	-726.0426	105.291
4_(P_III)_method_A_DCM_smd.log	-726.1662	-726.0102	-725.9990	-725.9981	-726.0482	105.486
4_(P_III)_method_B.log	-726.3234	-726.1668	-726.1558	-726.1548	-726.2048	105.075
4_(P_III)_method_B_DCM.log	-726.3291	-726.1730	-726.1618	-726.1609	-726.2109	105.288
4_(P_III)_method_B_DCM_smd.log	-726.3347	-726.1786	-726.1676	-726.1667	-726.2160	103.724
4_(P_III)_method_B_DMSO.log	-726.3301	-726.1741	-726.1630	-726.1620	-726.2121	105.411
4_(P_III)_method_B_DMSO_smd.log	-726.3323	-726.1764	-726.1653	-726.1644	-726.2145	105.495
4_(P_III)_method_B_MeOH.log	-726.3300	-726.1740	-726.1629	-726.1619	-726.2120	105.389
4_(P_III)_method_B_MeOH_smd.log	-726.3365	-726.1806	-726.1695	-726.1685	-726.2190	106.235
4_(P_III)_method_B_THF.log	-726.3288	-726.1727	-726.1616	-726.1606	-726.2107	105.278
4_(P_III)_method_B_THF_smd.log	-726.3330	-726.1770	-726.1659	-726.1650	-726.2150	105.313
4_(P_III)_method_B_toluene.log	-726.3264	-726.1701	-726.1590	-726.1581	-726.2081	105.353
4_(P_III)_method_B_toluene_smd.log	-726.3311	-726.1747	-726.1637	-726.1627	-726.2127	105.114
4_(P_III)_method_B_water.log	-726.3302	-726.1742	-726.1631	-726.1621	-726.2122	105.435
4_(P_III)_method_B_water_smd.log	-726.3303	-726.1744	-726.1632	-726.1623	-726.2129	106.443
4_(P_III)_method_D_DCM.log	-726.1864	-726.0302	-726.0189	-726.0180	-726.0688	106.864
4_(P_V)_method_A.log	-726.1608	-726.0038	-725.9932	-725.9922	-726.0422	105.080
4_(P_V)_method_A_DCM.log	-726.1702	-726.0135	-726.0029	-726.0019	-726.0519	105.123
4_(P_V)_method_A_DCM_smd.log	-726.1752	-726.0187	-726.0081	-726.0071	-726.0568	104.576
4_(P_V)_method_B.log	-726.3403	-726.1834	-726.1728	-726.1719	-726.2219	105.229
4_(P_V)_method_B_DCM.log	-726.3489	-726.1923	-726.1817	-726.1807	-726.2306	105.055
4_(P_V)_method_B_DCM_smd.log	-726.3541	-726.1975	-726.1870	-726.1860	-726.2350	103.014
4_(P_V)_method_B_DMSO.log	-726.3504	-726.1938	-726.1832	-726.1822	-726.2321	104.933
4_(P_V)_method_B_DMSO_smd.log	-726.3530	-726.1966	-726.1859	-726.1850	-726.2350	105.278
4_(P_V)_method_B_MeOH.log	-726.3502	-726.1937	-726.1830	-726.1821	-726.2319	104.942
4_(P_V)_method_B_MeOH_smd.log	-726.3569	-726.2005	-726.1899	-726.1889	-726.2388	104.896
4_(P_V)_method_B_THF.log	-726.3486	-726.1920	-726.1813	-726.1804	-726.2303	105.092
4_(P_V)_method_B_THF_smd.log	-726.3530	-726.1964	-726.1858	-726.1849	-726.2347	104.864

4_(P_V)_method_B_toluene.log	-726.3450	-726.1883	-726.1777	-726.1767	-726.2271	106.050
4_(P_V)_method_B_toluene_smd.log	-726.3498	-726.1930	-726.1824	-726.1814	-726.2313	104.830
4_(P_V)_method_B_water.log	-726.3505	-726.1940	-726.1833	-726.1824	-726.2323	104.923
4_(P_V)_method_B_water_smd.log	-726.3508	-726.1944	-726.1837	-726.1828	-726.2326	104.787
4_(P_V)_method_D_DCM.log	-726.1961	-726.0394	-726.0287	-726.0277	-726.0782	106.162
5_(P_III)_method_A.log	-616.9644	-616.9448	-616.9402	-616.9393	-616.9722	69.376
5_(P_III)_method_A_DCM.log	-616.9696	-616.9503	-616.9457	-616.9448	-616.9779	69.619
5_(P_III)_method_A_DCM_smd.log	-616.9707	-616.9518	-616.9472	-616.9462	-616.9794	69.867
5_(P_III)_method_B.log	-617.1057	-617.0860	-617.0815	-617.0805	-617.1134	69.198
5_(P_III)_method_B_DCM.log	-617.1100	-617.0906	-617.0860	-617.0851	-617.1180	69.366
5_(P_III)_method_B_DCM_smd.log	-617.1109	-617.0918	-617.0872	-617.0863	-617.1194	69.689
5_(P_III)_method_C_DCM.log	-617.1174	-617.0980	-617.0934	-617.0925	-617.1255	69.394
5_(P_III)_method_C_DCM_smd.log	-617.1183	-617.0992	-617.0946	-617.0937	-617.1268	69.686
5_(P_III)_method_D_DCM.log	-616.9741	-616.9548	-616.9502	-616.9493	-616.9824	69.648
5_(P_III)_method_E_DCM.log	-617.0048	-616.9848	-616.9803	-616.9794	-617.0122	69.014
5_(P_III)_method_E_DCM_smd.log	-617.0056	-616.9860	-616.9815	-616.9806	-617.0135	69.305
5_(P_V)_method_A.log	-616.9548	-616.9348	-616.9308	-616.9298	-616.9618	67.409
5_(P_V)_method_A_DCM.log	-616.9626	-616.9427	-616.9387	-616.9378	-616.9699	67.533
5_(P_V)_method_A_DCM_smd.log	-616.9638	-616.9440	-616.9399	-616.9390	-616.9711	67.566
5_(P_V)_method_B.log	-617.1086	-617.0882	-617.0842	-617.0833	-617.1152	67.104
5_(P_V)_method_B_DCM.log	-617.1155	-617.0953	-617.0913	-617.0904	-617.1223	67.197
5_(P_V)_method_B_DCM_smd.log	-617.1161	-617.0960	-617.0920	-617.0910	-617.1230	67.230
5_(P_V)_method_C_DCM.log	-617.1156	-617.0957	-617.0917	-617.0907	-617.1227	67.328
5_(P_V)_method_C_DCM_smd.log	-617.1165	-617.0965	-617.0925	-617.0916	-617.1236	67.358
5_(P_V)_method_D_DCM.log	-616.9673	-616.9475	-616.9434	-616.9425	-616.9746	67.533
5_(P_V)_method_E_DCM.log	-617.0122	-616.9917	-616.9878	-616.9868	-617.0187	67.085
5_(P_V)_method_E_DCM_smd.log	-617.0127	-616.9923	-616.9883	-616.9874	-617.0193	67.114
6_(P_III)_method_A.log	-1092.4838	-1092.4404	-1092.4300	-1092.4290	-1092.4778	102.699
6_(P_III)_method_A_DCM.log	-1092.4908	-1092.4478	-1092.4374	-1092.4365	-1092.4854	102.969
6_(P_III)_method_A_DCM_smd.log	-1092.4908	-1092.4483	-1092.4387	-1092.4377	-1092.4838	97.070
6_(P_III)_method_B.log	-1092.7750	-1092.7314	-1092.7210	-1092.7201	-1092.7686	102.021
6_(P_III)_method_B_DCM.log	-1092.7811	-1092.7380	-1092.7276	-1092.7267	-1092.7751	101.847
6_(P_III)_method_B_DCM_smd.log	-1092.7811	-1092.7384	-1092.7288	-1092.7278	-1092.7738	96.625
6_(P_III)_method_B_DMSO.log	-1092.7823	-1092.7392	-1092.7289	-1092.7279	-1092.7763	101.817
6_(P_III)_method_B_DMSO_smd.log	-1092.7790	-1092.7363	-1092.7259	-1092.7249	-1092.7738	102.974
6_(P_III)_method_B_MeOH.log	-1092.7822	-1092.7391	-1092.7287	-1092.7278	-1092.7762	101.822
6_(P_III)_method_B_MeOH_smd.log	-1092.7844	-1092.7416	-1092.7312	-1092.7302	-1092.7795	103.583
6_(P_III)_method_B_THF.log	-1092.7809	-1092.7377	-1092.7273	-1092.7264	-1092.7748	101.849
6_(P_III)_method_B_THF_smd.log	-1092.7791	-1092.7362	-1092.7258	-1092.7249	-1092.7738	103.034
6_(P_III)_method_B_toluene.log	-1092.7783	-1092.7349	-1092.7245	-1092.7236	-1092.7720	101.836

6_(P_III)_method_B_toluene_smd.log	-1092.7765	-1092.7332	-1092.7229	-1092.7219	-1092.7705	102.297
6_(P_III)_method_B_water.log	-1092.7824	-1092.7393	-1092.7290	-1092.7280	-1092.7764	101.813
6_(P_III)_method_B_water_smd.log	-1092.7786	-1092.7358	-1092.7253	-1092.7244	-1092.7736	103.505
6_(P_III)_method_C_DCM.log	-1092.8019	-1092.7588	-1092.7485	-1092.7475	-1092.7962	102.371
6_(P_III)_method_C_DCM_smd.log	-1092.8016	-1092.7590	-1092.7485	-1092.7476	-1092.7971	104.354
6_(P_III)_method_D_DCM.log	-1092.5072	-1092.4641	-1092.4537	-1092.4527	-1092.5037	107.203
6_(P_III)_method_E_DCM.log	-1092.5475	-1092.5031	-1092.4929	-1092.4920	-1092.5399	100.986
6_(P_III)_method_E_DCM_smd.log	-1092.5472	-1092.5031	-1092.4929	-1092.4919	-1092.5406	102.447
6_(P_V)_method_A.log	-1092.4679	-1092.4253	-1092.4152	-1092.4143	-1092.4629	102.349
6_(P_V)_method_A_DCM.log	-1092.4759	-1092.4337	-1092.4235	-1092.4225	-1092.4719	103.851
6_(P_V)_method_A_DCM_smd.log	-1092.4766	-1092.4345	-1092.4243	-1092.4234	-1092.4721	102.544
6_(P_V)_method_B.log	-1092.7677	-1092.7249	-1092.7149	-1092.7140	-1092.7630	103.312
6_(P_V)_method_B_DCM.log	-1092.7748	-1092.7325	-1092.7224	-1092.7214	-1092.7705	103.325
6_(P_V)_method_B_DMSO.log	-1092.7762	-1092.7340	-1092.7239	-1092.7229	-1092.7722	103.785
6_(P_V)_method_B_DMSO_smd.log	-1092.7757	-1092.7334	-1092.7234	-1092.7224	-1092.7708	101.823
6_(P_V)_method_B_MeOH.log	-1092.7761	-1092.7339	-1092.7237	-1092.7228	-1092.7720	103.658
6_(P_V)_method_B_MeOH_smd.log	-1092.7781	-1092.7358	-1092.7257	-1092.7248	-1092.7731	101.654
6_(P_V)_method_B_THF.log	-1092.7745	-1092.7322	-1092.7221	-1092.7211	-1092.7702	103.297
6_(P_V)_method_B_THF_smd.log	-1092.7746	-1092.7322	-1092.7231	-1092.7222	-1092.7675	95.492
6_(P_V)_method_B_toluene.log	-1092.7714	-1092.7289	-1092.7188	-1092.7178	-1092.7671	103.742
6_(P_V)_method_B_toluene_smd.log	-1092.7703	-1092.7278	-1092.7177	-1092.7168	-1092.7657	102.952
6_(P_V)_method_B_water.log	-1092.7764	-1092.7342	-1092.7240	-1092.7231	-1092.7725	104.047
6_(P_V)_method_B_water_smd.log	-1092.7724	-1092.7301	-1092.7200	-1092.7191	-1092.7674	101.548
6_(P_V)_method_C_DCM.log	-1092.7903	-1092.7483	-1092.7381	-1092.7372	-1092.7863	103.398
6_(P_V)_method_C_DCM_smd.log	-1092.7907	-1092.7486	-1092.7395	-1092.7385	-1092.7839	95.430
6_(P_V)_method_D_DCM.log	-1092.4927	-1092.4504	-1092.4403	-1092.4393	-1092.4886	103.679
6_(P_V)_method_E_DCM.log	-1092.5423	-1092.4989	-1092.4889	-1092.4879	-1092.5375	104.300
6_(P_V)_method_E_DCM_smd.log	-1092.5423	-1092.4989	-1092.4890	-1092.4881	-1092.5360	100.913
6_P_V_methodB_DCM_smd.log	-1092.7752	-1092.7328	-1092.7228	-1092.7218	-1092.7708	103.027
7_(P_III)_method_A.log	-794.7693	-794.7026	-794.6943	-794.6934	-794.7358	89.230
7_(P_III)_method_A_DCM.log	-794.7755	-794.7093	-794.7009	-794.6999	-794.7426	89.773
7_(P_III)_method_A_DCM_smd.log	-794.7773	-794.7111	-794.7028	-794.7018	-794.7442	89.175
7_(P_III)_method_B.log	-794.9574	-794.8908	-794.8826	-794.8816	-794.9238	88.805
7_(P_III)_method_B_DCM.log	-794.9628	-794.8966	-794.8883	-794.8874	-794.9297	89.093
7_(P_III)_method_B_DCM_smd.log	-794.9645	-794.8983	-794.8901	-794.8891	-794.9313	88.734
7_(P_III)_method_C_DCM.log	-794.9746	-794.9085	-794.9002	-794.8992	-794.9417	89.402

7_(P_III)_method_C_DCM_smd.log	-794.9762	-794.9102	-794.9019	-794.9009	-794.9432	88.968
7_(P_III)_method_D_DCM.log	-794.7906	-794.7243	-794.7159	-794.7150	-794.7576	89.647
7_(P_III)_method_E_DCM.log	-794.8084	-794.7410	-794.7329	-794.7319	-794.7739	88.252
7_(P_III)_method_E_DCM_smd.log	-794.8100	-794.7427	-794.7346	-794.7336	-794.7755	88.097
7_(P_V)_method_A.log	-794.7621	-794.6963	-794.6884	-794.6874	-794.7295	88.570
7_(P_V)_method_A_DCM.log	-794.7729	-794.7074	-794.6994	-794.6985	-794.7410	89.635
7_(P_V)_method_A_DCM_smd.log	-794.7750	-794.7095	-794.7015	-794.7006	-794.7429	88.966
7_(P_V)_method_B.log	-794.9594	-794.8936	-794.8857	-794.8848	-794.9267	88.232
7_(P_V)_method_B_DCM.log	-794.9691	-794.9037	-794.8957	-794.8948	-794.9370	88.807
7_(P_V)_method_B_DCM_smd.log	-794.9709	-794.9055	-794.8976	-794.8966	-794.9387	88.667
7_(P_V)_method_C_DCM.log	-794.9754	-794.9102	-794.9022	-794.9013	-794.9437	89.351
7_(P_V)_method_C_DCM_smd.log	-794.9772	-794.9120	-794.9040	-794.9031	-794.9452	88.694
7_(P_V)_method_D_DCM.log	-794.7885	-794.7229	-794.7149	-794.7140	-794.7562	88.757
7_(P_V)_method_E_DCM.log	-794.8157	-794.7491	-794.7414	-794.7404	-794.7820	87.564
7_(P_V)_method_E_DCM_smd.log	-794.8172	-794.7508	-794.7430	-794.7421	-794.7841	88.395
8_(P_III)_method_A.log	-869.9969	-869.9250	-869.9156	-869.9147	-869.9601	95.531
8_(P_III)_method_A_DCM.log	-870.0037	-869.9323	-869.9228	-869.9218	-869.9675	96.070
8_(P_III)_method_A_DCM_smd.log	-870.0050	-869.9336	-869.9242	-869.9233	-869.9685	95.057
8_(P_III)_method_B.log	-870.2171	-870.1452	-870.1358	-870.1349	-870.1802	95.321
8_(P_III)_method_B_DCM.log	-870.2230	-870.1516	-870.1421	-870.1412	-870.1868	96.020
8_(P_III)_method_B_DCM_smd.log	-870.2242	-870.1528	-870.1435	-870.1425	-870.1877	95.029
8_(P_III)_method_D_DCM.log	-870.0207	-869.9491	-869.9397	-869.9387	-869.9839	95.113
8_(P_V)_method_A.log	-869.9942	-869.9227	-869.9135	-869.9126	-869.9582	95.979
8_(P_V)_method_A_DCM.log	-870.0028	-869.9315	-869.9223	-869.9214	-869.9671	96.184
8_(P_V)_method_A_DCM_smd.log	-870.0040	-869.9328	-869.9237	-869.9227	-869.9682	95.647
8_(P_V)_method_B.log	-870.2240	-870.1524	-870.1433	-870.1423	-870.1879	95.868
8_(P_V)_method_B_DCM.log	-870.2318	-870.1605	-870.1513	-870.1504	-870.1965	97.144
8_(P_V)_method_B_DCM_smd.log	-870.2327	-870.1614	-870.1523	-870.1514	-870.1967	95.327
8_(P_V)_method_D_DCM.log	-870.0199	-869.9485	-869.9394	-869.9384	-869.9842	96.255
9_(P_III)_method_A.log	-986.5117	-986.3916	-986.3802	-986.3792	-986.4305	107.999
9_(P_III)_method_A_DCM.log	-986.5186	-986.3988	-986.3873	-986.3864	-986.4376	107.878
9_(P_III)_method_A_DCM_smd.log	-986.5254	-986.4061	-986.3945	-986.3936	-986.4451	108.465
9_(P_III)_method_B.log	-986.7485	-986.6286	-986.6171	-986.6162	-986.6673	107.659
9_(P_III)_method_B_DCM.log	-986.7547	-986.6350	-986.6236	-986.6226	-986.6736	107.302
9_(P_III)_method_B_DCM_smd.log	-986.7612	-986.6420	-986.6305	-986.6295	-986.6809	108.161
9_(P_III)_method_C_DCM_smd.log	-986.7776	-986.6584	-986.6468	-986.6459	-986.6975	108.673
9_(P_III)_method_D_DCM.log	-986.5547	-986.4347	-986.4233	-986.4224	-986.4734	107.434
9_(P_III)_method_E_DCM.log	-986.5296	-986.4084	-986.3971	-986.3961	-986.4470	107.085
9_(P_III)_method_E_DCM_smd.log	-986.5362	-986.4154	-986.4040	-986.4030	-986.4542	107.685
9_(P_III)method_C_DCM.log	-986.7711	-986.6515	-986.6400	-986.6391	-986.6902	107.521
9_(P_V)_method_A.log	-986.5051	-986.3856	-986.3746	-986.3737	-986.4244	106.631
9_(P_V)_method_A_DCM.log	-986.5154	-986.3961	-986.3851	-986.3841	-986.4347	106.361
9_(P_V)_method_A_DCM_smd.log	-986.5226	-986.4034	-986.3924	-986.3915	-986.4418	105.894
9_(P_V)_method_B.log	-986.7510	-986.6316	-986.6206	-986.6197	-986.6703	106.405
9_(P_V)_method_B_DCM.log	-986.7603	-986.6410	-986.6301	-986.6291	-986.6796	106.299

9_(P_V)_method_B_DCM_smd.log	-986.7670	-986.6479	-986.6370	-986.6360	-986.6864	105.924
9_(P_V)_method_C_DCM.log	-986.7714	-986.6522	-986.6412	-986.6402	-986.6908	106.325
9_(P_V)_method_C_DCM_smd.log	-986.7781	-986.6590	-986.6481	-986.6471	-986.6975	105.866
9_(P_V)_method_D_DCM.log	-986.5520	-986.4326	-986.4216	-986.4206	-986.4715	107.033
9_(P_V)_method_E_DCM.log	-986.5360	-986.4150	-986.4042	-986.4033	-986.4535	105.610
9_(P_V)_method_E_DCM_smd.log	-986.5426	-986.4219	-986.4110	-986.4101	-986.4604	105.928
10_(P_III)_method_A.log	-874.1503	-874.0308	-874.0182	-874.0172	-874.0724	116.074
10_(P_III)_method_A_DCM.log	-874.1648	-874.0456	-874.0329	-874.0320	-874.0869	115.524
10_(P_III)_method_A_DCM_smd.log	-874.1657	-874.0465	-874.0340	-874.0330	-874.0867	113.038
10_(P_III)_method_B.log	-874.3639	-874.2446	-874.2320	-874.2311	-874.2853	114.036
10_(P_III)_method_B_DCM.log	-874.3770	-874.2579	-874.2453	-874.2443	-874.2983	113.587
10_(P_III)_method_C_DCM.log	-874.3863	-874.2676	-874.2549	-874.2540	-874.3081	113.886
10_(P_III)_method_C_DCM_smd.log	-874.3872	-874.2685	-874.2559	-874.2549	-874.3087	113.184
10_(P_III)_method_D_DCM.log	-874.1910	-874.0717	-874.0600	-874.0590	-874.1103	107.840
10_(P_III)_method_E_DCM.log	-874.1769	-874.0553	-874.0432	-874.0423	-874.0945	109.801
10_(P_III)_method_E_DCM_smd.log	-874.1777	-874.0562	-874.0442	-874.0432	-874.0950	108.991
10_(P_V)_method_A.log	-874.1387	-874.0198	-874.0076	-874.0067	-874.0602	112.626
10_(P_V)_method_A_DCM.log	-874.1536	-874.0350	-874.0229	-874.0219	-874.0753	112.407
10_(P_V)_method_A_DCM_smd.log	-874.1550	-874.0363	-874.0242	-874.0233	-874.0763	111.468
10_(P_V)_method_B.log	-874.3579	-874.2393	-874.2271	-874.2262	-874.2802	113.717
10_(P_V)_method_B_DCM.log	-874.3733	-874.2548	-874.2427	-874.2417	-874.2953	112.681
10_(P_V)_method_B_DCM_smd.log	-874.3743	-874.2557	-874.2437	-874.2428	-874.2960	112.052
10_(P_V)_method_C_DCM.log	-874.3775	-874.2593	-874.2472	-874.2463	-874.2998	112.759
10_(P_V)_method_C_DCM_smd.log	-874.3787	-874.2604	-874.2484	-874.2475	-874.3001	110.844
10_(P_V)_method_D_DCM.log	-874.1803	-874.0618	-874.0495	-874.0485	-874.1042	117.225
10_(P_V)_method_E_DCM.log	-874.1735	-874.0531	-874.0412	-874.0403	-874.0932	111.312
10_(P_V)_method_E_DCM_smd.log	-874.1746	-874.0542	-874.0423	-874.0414	-874.0942	111.101
10_P_III_method_B_DCM_smd.log	-874.3778	-874.2587	-874.2462	-874.2453	-874.2987	112.468
11_(P_III)_method_A.log	-906.0402	-905.9441	-905.9330	-905.9320	-905.9844	110.216
11_(P_III)_method_A_DCM.log	-906.0576	-905.9614	-905.9503	-905.9494	-906.0017	110.223
11_(P_III)_method_A_DCM_smd.log	-906.0637	-905.9674	-905.9564	-905.9555	-906.0070	108.550
11_(P_III)_method_B.log	-906.2652	-906.1691	-906.1580	-906.1571	-906.2093	109.845
11_(P_III)_method_B_DCM.log	-906.2813	-906.1852	-906.1741	-906.1732	-906.2254	109.847
11_(P_III)_method_B_DCM_smd.log	-906.2870	-906.1907	-906.1798	-906.1789	-906.2299	107.455
11_(P_III)_method_D_DCM.log	-906.0833	-905.9870	-905.9758	-905.9749	-906.0271	109.895
11_(P_III)_method_E_DCM.log	-906.0740	-905.9759	-905.9651	-905.9641	-906.0159	108.885
11_(P_III)_method_E_DCM_smd.log	-906.0797	-905.9814	-905.9706	-905.9697	-906.0203	106.499
11_(P_V)_method_A.log	-906.0284	-905.9328	-905.9222	-905.9212	-905.9723	107.490
11_(P_V)_method_A_DCM.log	-906.0509	-905.9551	-905.9445	-905.9435	-905.9953	108.870
11_(P_V)_method_A_DCM_smd.log	-906.0580	-905.9624	-905.9517	-905.9508	-906.0029	109.809
11_(P_V)_method_B.log	-906.2629	-906.1674	-906.1568	-906.1558	-906.2068	107.334
11_(P_V)_method_B_DCM.log	-906.2834	-906.1876	-906.1771	-906.1761	-906.2274	107.902
11_(P_V)_method_B_DCM_smd.log	-906.2900	-906.1944	-906.1838	-906.1828	-906.2347	109.076
11_(P_V)_method_D_DCM.log	-906.0774	-905.9814	-905.9709	-905.9699	-906.0212	108.008
11_(P_V)_method_E_DCM.log	-906.0773	-905.9797	-905.9693	-905.9684	-906.0197	108.009

12_(P_III)_method_A.log	-880.5280	-880.3314	-880.3190	-880.3180	-880.3725	114.609
12_(P_III)_method_A_DCM.log	-880.5347	-880.3384	-880.3258	-880.3249	-880.3796	115.190
12_(P_III)_method_A_DCM_smd.log	-880.5479	-880.3517	-880.3392	-880.3383	-880.3925	114.186
12_(P_III)_method_B.log	-880.7111	-880.5148	-880.5023	-880.5014	-880.5558	114.566
12_(P_III)_method_B_DCM_smd.log	-880.7301	-880.5341	-880.5216	-880.5207	-880.5746	113.376
12_(P_III)_method_B_DKM.log	-880.7172	-880.5211	-880.5086	-880.5076	-880.5623	114.957
12_(P_III)_method_B_DMSO.log	-880.7184	-880.5224	-880.5098	-880.5088	-880.5635	115.050
12_(P_III)_method_B_DMSO_smd.log	-880.7233	-880.5274	-880.5148	-880.5139	-880.5683	114.558
12_(P_III)_method_B_MeOH.log	-880.7182	-880.5222	-880.5096	-880.5087	-880.5634	115.042
12_(P_III)_method_B_MeOH_smd.log	-880.7275	-880.5316	-880.5190	-880.5181	-880.5724	114.445
12_(P_III)_method_B_THF.log	-880.7169	-880.5209	-880.5083	-880.5074	-880.5620	114.924
12_(P_III)_method_B_THF_smd.log	-880.7262	-880.5302	-880.5176	-880.5167	-880.5708	113.943
12_(P_III)_method_B_toluene.log	-880.7143	-880.5181	-880.5056	-880.5047	-880.5593	114.948
12_(P_III)_method_B_toluene_smd.log	-880.7255	-880.5294	-880.5169	-880.5159	-880.5701	113.921
12_(P_III)_method_B_water.log	-880.7185	-880.5225	-880.5099	-880.5090	-880.5636	115.073
12_(P_III)_method_B_water_smd.log	-880.7185	-880.5225	-880.5109	-880.5099	-880.5614	108.280
12_(P_III)_method_C_DCM.log	-880.7297	-880.5335	-880.5209	-880.5200	-880.5749	115.563
12_(P_III)_method_C_DCM_smd.log	-880.7425	-880.5464	-880.5339	-880.5329	-880.5871	113.948
12_(P_III)_method_D_DCM.log	-880.5915	-880.3949	-880.3823	-880.3814	-880.4364	115.793
12_(P_III)_method_E_DCM.log	-880.5011	-880.3028	-880.2904	-880.2895	-880.3436	113.945
12_(P_III)_method_E_DCM_smd.log	-880.5144	-880.3163	-880.3039	-880.3030	-880.3567	113.108
12_(P_V)_method_A.log	-880.5298	-880.3336	-880.3217	-880.3207	-880.3740	112.092
12_(P_V)_method_A_DCM.log	-880.5417	-880.3455	-880.3336	-880.3326	-880.3868	114.062
12_(P_V)_method_A_DCM_smd.log	-880.5545	-880.3586	-880.3466	-880.3456	-880.3991	112.601
12_(P_V)_method_B.log	-880.7220	-880.5261	-880.5142	-880.5132	-880.5664	111.947
12_(P_V)_method_B_DCM_smd.log	-880.7453	-880.5495	-880.5375	-880.5366	-880.5909	114.377
12_(P_V)_method_B_DKM.log	-880.7328	-880.5369	-880.5250	-880.5240	-880.5777	113.057
12_(P_V)_method_B_DMSO.log	-880.7348	-880.5390	-880.5270	-880.5260	-880.5809	115.500
12_(P_V)_method_B_DMSO_smd.log	-880.7398	-880.5441	-880.5321	-880.5312	-880.5850	113.261
12_(P_V)_method_B_MeOH.log	-880.7346	-880.5388	-880.5268	-880.5258	-880.5822	118.672
12_(P_V)_method_B_MeOH_smd.log	-880.7456	-880.5499	-880.5379	-880.5369	-880.5915	114.883
12_(P_V)_method_B_THF.log	-880.7323	-880.5364	-880.5245	-880.5235	-880.5772	113.025
12_(P_V)_method_B_THF_smd.log	-880.7416	-880.5457	-880.5338	-880.5329	-880.5862	112.218
12_(P_V)_method_B_toluene.log	-880.7277	-880.5317	-880.5198	-880.5189	-880.5723	112.342
12_(P_V)_method_B_toluene_smd.log	-880.7390	-880.5431	-880.5312	-880.5303	-880.5836	112.272
12_(P_V)_method_B_water.log	-880.7350	-880.5392	-880.5272	-880.5263	-880.5809	114.935
12_(P_V)_method_B_water_smd.log	-880.7370	-880.5413	-880.5302	-880.5293	-880.5801	107.095
12_(P_V)_method_C_DCM.log	-880.7399	-880.5440	-880.5320	-880.5311	-880.5852	113.820
12_(P_V)_method_C_DCM_smd.log	-880.7523	-880.5564	-880.5445	-880.5436	-880.5971	112.635
12_(P_V)_method_D_DCM.log	-880.5989	-880.4025	-880.3906	-880.3896	-880.4432	112.696
12_(P_V)_method_E_DCM.log	-880.5171	-880.3191	-880.3073	-880.3064	-880.3593	111.255
12_(P_V)_method_E_DCM_smd.log	-880.5297	-880.3317	-880.3199	-880.3190	-880.3714	110.336
13_(P_III)_method_A.log	-803.3401	-803.1631	-803.1515	-803.1506	-803.2018	107.799
13_(P_III)_method_A_DCM.log	-806.7379	-806.5105	-806.4971	-806.4961	-806.5499	113.258
13_(P_III)_method_A_DCM_smd.log	-806.7480	-806.5209	-806.5074	-806.5065	-806.5604	113.485

13_(P_III)_method_B.log	-806.8961	-806.6688	-806.6554	-806.6545	-806.7080	112.705
13_(P_III)_method_B_DCM_smd.log	-806.9106	-806.6842	-806.6707	-806.6698	-806.7239	113.853
13_(P_III)_method_B_DKM.log	-806.9005	-806.6736	-806.6602	-806.6592	-806.7130	113.224
13_(P_III)_method_B_DMSO.log	-806.9013	-806.6745	-806.6611	-806.6601	-806.7141	113.542
13_(P_III)_method_B_DMSO_smd.log	-806.9047	-806.6782	-806.6647	-806.6638	-806.7178	113.780
13_(P_III)_method_B_MeOH.log	-806.9013	-806.6744	-806.6610	-806.6600	-806.7140	113.500
13_(P_III)_method_B_MeOH_smd.log	-806.9096	-806.6832	-806.6697	-806.6687	-806.7228	113.776
13_(P_III)_method_B_THF.log	-806.9003	-806.6734	-806.6600	-806.6590	-806.7128	113.166
13_(P_III)_method_B_THF_smd.log	-806.9073	-806.6806	-806.6672	-806.6662	-806.7202	113.546
13_(P_III)_method_B_toluene.log	-806.8985	-806.6713	-806.6580	-806.6570	-806.7106	112.820
13_(P_III)_method_B_toluene_smd.log	-806.9070	-806.6799	-806.6665	-806.6656	-806.7193	113.159
13_(P_III)_method_B_water.log	-806.9014	-806.6746	-806.6612	-806.6602	-806.7142	113.585
13_(P_III)_method_B_water_smd.log	-806.9003	-806.6738	-806.6603	-806.6593	-806.7134	113.820
13_(P_III)_method_D_DCM.log	-806.7947	-806.5669	-806.5535	-806.5526	-806.6061	112.745
13_(P_V)_method_A.log	-803.3464	-803.1696	-803.1584	-803.1575	-803.2084	107.108
13_(P_V)_method_A_DCM.log	-806.7470	-806.5198	-806.5069	-806.5060	-806.5590	111.630
13_(P_V)_method_A_DCM_smd.log	-806.7569	-806.5301	-806.5171	-806.5162	-806.5693	111.846
13_(P_V)_method_B.log	-806.9086	-806.6819	-806.6690	-806.6680	-806.7214	112.236
13_(P_V)_method_B_DCM_smd.log	-806.9280	-806.7016	-806.6888	-806.6878	-806.7406	111.089
13_(P_V)_method_B_DKM.log	-806.9183	-806.6916	-806.6787	-806.6778	-806.7309	111.684
13_(P_V)_method_B_DMSO.log	-806.9202	-806.6935	-806.6806	-806.6797	-806.7326	111.529
13_(P_V)_method_B_DMSO_smd.log	-806.9236	-806.6973	-806.6844	-806.6834	-806.7366	111.921
13_(P_V)_method_B_MeOH.log	-806.9199	-806.6933	-806.6804	-806.6794	-806.7324	111.519
13_(P_V)_method_B_MeOH_smd.log	-806.9309	-806.7045	-806.6916	-806.6907	-806.7437	111.524
13_(P_V)_method_B_THF.log	-806.9179	-806.6912	-806.6783	-806.6774	-806.7305	111.753
13_(P_V)_method_B_THF_smd.log	-806.9249	-806.6985	-806.6856	-806.6846	-806.7377	111.736
13_(P_V)_method_B_toluene.log	-806.9137	-806.6871	-806.6741	-806.6732	-806.7265	112.223
13_(P_V)_method_B_toluene_smd.log	-806.9223	-806.6957	-806.6828	-806.6819	-806.7351	112.070
13_(P_V)_method_B_water.log	-806.9203	-806.6937	-806.6808	-806.6799	-806.7329	111.547
13_(P_V)_method_B_water_smd.log	-806.9223	-806.6958	-806.6829	-806.6819	-806.7349	111.474
13_(P_V)_method_D_DCM.log	-806.8044	-806.5771	-806.5642	-806.5632	-806.6165	112.110
14_(P_III)_method_A.log	-806.7331	-806.5052	-806.4919	-806.4910	-806.5445	112.731
14_(P_III)_method_A_DCM.log	-803.3465	-803.1698	-803.1582	-803.1572	-803.2087	108.260
14_(P_III)_method_A_DCM_smd.log	-803.3554	-803.1790	-803.1674	-803.1664	-803.2181	108.684
14_(P_III)_method_B.log	-803.5154	-803.3387	-803.3271	-803.3261	-803.3775	108.164
14_(P_III)_method_B_DCM_smd.log	-803.5302	-803.3537	-803.3422	-803.3413	-803.3922	107.263
14_(P_III)_method_B_DKM.log	-803.5211	-803.3447	-803.3330	-803.3321	-803.3837	108.510
14_(P_III)_method_B_DMSO.log	-803.5221	-803.3458	-803.3341	-803.3332	-803.3848	108.717
14_(P_III)_method_B_MeOH.log	-803.5220	-803.3456	-803.3340	-803.3331	-803.3847	108.694
14_(P_III)_method_B_MeOH_smd.log	-803.5297	-803.3536	-803.3419	-803.3409	-803.3927	109.056
14_(P_III)_method_B_THF.log	-803.5209	-803.3444	-803.3328	-803.3319	-803.3834	108.473
14_(P_III)_method_B_THF_smd.log	-803.5274	-803.3511	-803.3395	-803.3385	-803.3907	109.812
14_(P_III)_method_B_toluene.log	-803.5185	-803.3418	-803.3303	-803.3293	-803.3807	108.247
14_(P_III)_method_B_toluene_smd.log	-803.5262	-803.3497	-803.3381	-803.3371	-803.3889	108.921
14_(P_III)_method_B_water.log	-803.5222	-803.3459	-803.3342	-803.3333	-803.3850	108.741

14_(P_III)_method_B_water_smd.log	-803.5222	-803.3460	-803.3343	-803.3334	-803.3852	108.961
14_(P_III)_method_C_DCM.log	-803.5300	-803.3536	-803.3419	-803.3410	-803.3925	108.388
14_(P_III)_method_C_DCM_smd.log	-803.5388	-803.3626	-803.3510	-803.3501	-803.4018	108.823
14_(P_III)_method_D_DCM.log	-803.3882	-803.2113	-803.1997	-803.1988	-803.2508	109.543
14_(P_III)_method_E_DCM.log	-803.3384	-803.1598	-803.1483	-803.1474	-803.1990	108.681
14_(P_III)_method_E_DCM_smd.log	-803.3476	-803.1689	-803.1577	-803.1567	-803.2067	105.165
14_(P_V)_method_A.log	-806.7362	-806.5091	-806.4962	-806.4952	-806.5488	112.873
14_(P_V)_method_A_DCM.log	-803.3563	-803.1798	-803.1686	-803.1676	-803.2189	107.921
14_(P_V)_method_A_DCM_smd.log	-803.3650	-803.1886	-803.1774	-803.1765	-803.2274	107.184
14_(P_V)_method_B.log	-803.5316	-803.3550	-803.3439	-803.3429	-803.3937	106.892
14_(P_V)_method_B_DCM_smd.log	-803.5493	-803.3732	-803.3620	-803.3611	-803.4121	107.383
14_(P_V)_method_B_DKM.log	-803.5408	-803.3644	-803.3532	-803.3523	-803.4032	107.268
14_(P_V)_method_B_DMSO.log	-803.5424	-803.3661	-803.3549	-803.3539	-803.4050	107.524
14_(P_V)_method_B_DMSO_smd.log	-803.5459	-803.3697	-803.3585	-803.3576	-803.4090	108.227
14_(P_V)_method_B_MeOH.log	-803.5422	-803.3659	-803.3547	-803.3537	-803.4048	107.490
14_(P_V)_method_B_MeOH_smd.log	-803.5508	-803.3746	-803.3634	-803.3625	-803.4134	107.225
14_(P_V)_method_B_THF.log	-803.5404	-803.3640	-803.3528	-803.3519	-803.4028	107.214
14_(P_V)_method_B_THF_smd.log	-803.5470	-803.3707	-803.3595	-803.3586	-803.4097	107.654
14_(P_V)_method_B_toluene.log	-803.5366	-803.3600	-803.3489	-803.3479	-803.3988	106.975
14_(P_V)_method_B_toluene_smd.log	-803.5445	-803.3679	-803.3568	-803.3558	-803.4065	106.659
14_(P_V)_method_B_water.log	-803.5426	-803.3662	-803.3550	-803.3541	-803.4052	107.556
14_(P_V)_method_B_water_smd.log	-803.5435	-803.3673	-803.3560	-803.3551	-803.4063	107.662
14_(P_V)_method_C_DCM.log	-803.5435	-803.3672	-803.3560	-803.3551	-803.4061	107.362
14_(P_V)_method_C_DCM_smd.log	-803.5520	-803.3758	-803.3646	-803.3637	-803.4147	107.362
14_(P_V)_method_D_DCM.log	-803.3983	-803.2215	-803.2103	-803.2094	-803.2606	107.825
14_(P_V)_method_E_DCM.log	-803.3589	-803.1804	-803.1695	-803.1685	-803.2189	105.946
14_(P_V)_method_E_DCM_smd.log	-803.3673	-803.1890	-803.1781	-803.1771	-803.2275	106.057
15_(P_III)_method_A.log	-919.8475	-919.6234	-919.6092	-919.6083	-919.6656	120.497
15_(P_III)_method_A_DCM.log	-919.8541	-919.6303	-919.6161	-919.6151	-919.6729	121.516
15_(P_III)_method_A_DCM_smd.log	-919.8676	-919.6441	-919.6299	-919.6289	-919.6867	121.646
15_(P_III)_method_B.log	-920.0400	-919.8162	-919.8021	-919.8011	-919.8586	120.900
15_(P_III)_method_B_DCM_smd.log	-920.0591	-919.8362	-919.8218	-919.8208	-919.8797	123.855
15_(P_III)_method_B_DKM.log	-920.0459	-919.8225	-919.8082	-919.8073	-919.8654	122.279
15_(P_III)_method_B_DMSO.log	-920.0470	-919.8237	-919.8094	-919.8084	-919.8669	123.050
15_(P_III)_method_B_MeOH.log	-920.0469	-919.8235	-919.8093	-919.8083	-919.8668	123.043
15_(P_III)_method_B_THF.log	-920.0456	-919.8222	-919.8080	-919.8070	-919.8651	122.150
15_(P_III)_method_B_toluene.log	-920.0431	-919.8195	-919.8053	-919.8044	-919.8620	121.270
15_(P_III)_method_B_water.log	-920.0471	-919.8238	-919.8095	-919.8085	-919.8669	122.839
15_(P_III)_method_D_DCM.log	-919.9190	-919.6947	-919.6806	-919.6797	-919.7367	119.954
15_(P_V)_method_A.log	-919.8501	-919.6263	-919.6128	-919.6118	-919.6682	118.705
15_(P_V)_method_A_DCM.log	-919.8610	-919.6374	-919.6237	-919.6227	-919.6797	119.843
15_(P_V)_method_A_DCM_smd.log	-919.8740	-919.6504	-919.6377	-919.6368	-919.6902	112.527
15_(P_V)_method_B.log	-920.0518	-919.8283	-919.8148	-919.8138	-919.8701	118.481
15_(P_V)_method_B_DCM_smd.log	-920.0742	-919.8510	-919.8374	-919.8364	-919.8936	120.342
15_(P_V)_method_B_DKM.log	-920.0617	-919.8381	-919.8246	-919.8237	-919.8799	118.413

15_(P_V)_method_B_DMSO.log	-920.0635	-919.8401	-919.8265	-919.8255	-919.8821	118.993
15_(P_V)_method_B_MeOH.log	-920.0633	-919.8399	-919.8263	-919.8253	-919.8819	118.947
15_(P_V)_method_B_THF.log	-920.0612	-919.8377	-919.8242	-919.8232	-919.8795	118.337
15_(P_V)_method_B_toluene.log	-920.0570	-919.8336	-919.8200	-919.8190	-919.8756	119.075
15_(P_V)_method_B_water.log	-920.0637	-919.8403	-919.8267	-919.8257	-919.8823	119.134
15_(P_V)_method_D_DCM.log	-919.9265	-919.7024	-919.6889	-919.6880	-919.7443	118.562
16_(P_III)_method_A.log	-1079.0095	-1078.8294	-1078.8153	-1078.8143	-1078.8732	123.866
16_(P_III)_method_A_DCM.log	-1079.0171	-1078.8374	-1078.8231	-1078.8222	-1078.8819	125.637
16_(P_III)_method_A_DCM_smd.log	-1079.0292	-1078.8499	-1078.8356	-1078.8346	-1078.8940	124.972
16_(P_III)_method_B.log	-1079.2609	-1079.0808	-1079.0667	-1079.0657	-1079.1244	123.543
16_(P_III)_method_B_DCM.log	-1079.2677	-1079.0879	-1079.0737	-1079.0728	-1079.1314	123.429
16_(P_III)_method_B_DCM_smd.log	-1079.2795	-1079.1000	-1079.0858	-1079.0848	-1079.1437	123.974
16_(P_III)_method_D_DCM.log	-1079.0745	-1078.8945	-1078.8803	-1078.8793	-1078.9383	124.080
16_(P_III)_method_E_DCM.log	-1078.9987	-1078.8166	-1078.8026	-1078.8017	-1078.8600	122.633
16_(P_III)_method_E_DCM_smd.log	-1079.0106	-1078.8288	-1078.8148	-1078.8138	-1078.8718	121.940
16_(P_V)_method_A.log	-1079.0104	-1078.8308	-1078.8172	-1078.8162	-1078.8742	122.038
16_(P_V)_method_A_DCM.log	-1079.0231	-1078.8436	-1078.8300	-1078.8290	-1078.8865	120.934
16_(P_V)_method_A_DCM_smd.log	-1079.0352	-1078.8558	-1078.8422	-1078.8413	-1078.8987	120.837
16_(P_V)_method_B.log	-1079.2712	-1079.0915	-1079.0780	-1079.0770	-1079.1347	121.324
16_(P_V)_method_B_DCM.log	-1079.2827	-1079.1030	-1079.0895	-1079.0885	-1079.1461	121.105
16_(P_V)_method_B_DCM_smd.log	-1079.2942	-1079.1147	-1079.1012	-1079.1002	-1079.1576	120.840
16_(P_V)_method_D_DCM.log	-1079.0810	-1078.9012	-1078.8877	-1078.8867	-1078.9440	120.590
16_(P_V)_method_E_DCM.log	-1079.0140	-1078.8323	-1078.8189	-1078.8180	-1078.8751	120.297
16_(P_V)_method_E_DCM_smd.log	-1079.0255	-1078.8438	-1078.8304	-1078.8295	-1078.8865	120.025
17_(P_III)_method_A.log	-1289.5453	-1289.3438	-1289.3263	-1289.3254	-1289.3933	142.943
17_(P_III)_method_A_DCM.log	-1289.5612	-1289.3600	-1289.3425	-1289.3415	-1289.4091	142.235
17_(P_III)_method_A_DCM_smd.log	-1289.5731	-1289.3722	-1289.3546	-1289.3536	-1289.4216	142.990
17_(P_III)_method_B_DCM_smd.log	-1289.8916	-1289.6908	-1289.6732	-1289.6723	-1289.7402	142.830
17_(P_III)_method_D_DCM.log	-1289.6299	-1289.4285	-1289.4109	-1289.4100	-1289.4778	142.770
17_(P_III)_method_E_DCM.log	-1289.5307	-1289.3267	-1289.3093	-1289.3083	-1289.3782	147.056
17_(P_V)_method_A.log	-1289.5417	-1289.3409	-1289.3238	-1289.3229	-1289.3901	141.466
17_(P_V)_method_A_DCM.log	-1289.5617	-1289.3610	-1289.3439	-1289.3430	-1289.4105	142.123
17_(P_V)_method_A_DCM_smd.log	-1289.5744	-1289.3738	-1289.3567	-1289.3557	-1289.4235	142.755
17_(P_V)_method_B_DCM_smd.log	-1289.9015	-1289.7011	-1289.6840	-1289.6831	-1289.7509	142.805
17_(P_V)_method_D_DCM.log	-1289.6310	-1289.4299	-1289.4129	-1289.4119	-1289.4791	141.375

17_(P_V)_method_E_DCM.log	-1289.5409	-1289.3372	-1289.3204	-1289.3194	-1289.3858	139.722
18_(P_III)_method_A.log	-1698.5456	-1698.3398	-1698.3173	-1698.3164	-1698.3943	163.904
18_(P_III)_method_A_DCM.log	-1698.5653	-1698.3602	-1698.3375	-1698.3365	-1698.4155	166.305
18_(P_III)_method_A_DCM_smd.log	-1698.5760	-1698.3707	-1698.3482	-1698.3472	-1698.4257	165.184
18_(P_III)_method_D_DCM.log	-1698.6513	-1698.4456	-1698.4230	-1698.4221	-1698.5005	164.994
18_(P_V)_method_A.log	-1698.5351	-1698.3296	-1698.3075	-1698.3066	-1698.3847	164.308
18_(P_V)_method_A_DCM.log	-1698.5581	-1698.3529	-1698.3308	-1698.3299	-1698.4075	163.382
18_(P_V)_method_A_DCM_smd.log	-1698.5692	-1698.3637	-1698.3417	-1698.3407	-1698.4182	163.068
18_(P_V)_method_D_DCM.log	-1698.6450	-1698.4394	-1698.4174	-1698.4165	-1698.4948	164.761
19_(P_III)_method_A.log	-2107.5097	-2107.3006	-2107.2727	-2107.2717	-2107.3626	191.236
19_(P_III)_method_A_DCM.log	-2107.5329	-2107.3241	-2107.2962	-2107.2952	-2107.3859	190.853
19_(P_III)_method_A_DCM_smd.log	-2107.5435	-2107.3344	-2107.3067	-2107.3057	-2107.3954	188.610
19_(P_III)_method_D_DCM.log	-2107.6388	-2107.4293	-2107.4015	-2107.4006	-2107.4910	190.380
19_(P_V)_method_A.log	-2107.4953	-2107.2867	-2107.2591	-2107.2581	-2107.3488	190.951
19_(P_V)_method_A_DCM.log	-2107.5233	-2107.3145	-2107.2871	-2107.2862	-2107.3757	188.386
19_(P_V)_method_A_DCM_smd.log	-2107.5327	-2107.3241	-2107.2965	-2107.2955	-2107.3872	192.936
19_(P_V)_method_D_DCM.log	-2107.6289	-2107.4197	-2107.3923	-2107.3914	-2107.4815	189.594
20_(P_III)_method_A.log	-1872.8485	-1872.7340	-1872.7125	-1872.7115	-1872.7859	156.451
20_(P_III)_method_A_DCM.log	-1872.8573	-1872.7432	-1872.7216	-1872.7207	-1872.7957	157.771
20_(P_III)_method_A_DCM_smd.log	-1872.8618	-1872.7481	-1872.7264	-1872.7255	-1872.8003	157.487
20_(P_III)_method_B.log	-1873.3779	-1873.2628	-1873.2414	-1873.2405	-1873.3145	155.723
20_(P_III)_method_B_DCM.log	-1873.3858	-1873.2712	-1873.2499	-1873.2489	-1873.3230	155.954
20_(P_III)_method_B_DMSO.log	-1873.3875	-1873.2730	-1873.2516	-1873.2506	-1873.3248	156.197
20_(P_III)_method_B_MeOH.log	-1873.3873	-1873.2728	-1873.2514	-1873.2504	-1873.3246	156.037
20_(P_III)_method_B_THF.log	-1873.3851	-1873.2706	-1873.2492	-1873.2482	-1873.3230	157.300
20_(P_III)_method_B_toluene.log	-1873.3818	-1873.2669	-1873.2456	-1873.2447	-1873.3191	156.638
20_(P_III)_method_B_water.log	-1873.3876	-1873.2732	-1873.2518	-1873.2508	-1873.3254	157.041
20_(P_III)_method_D_DCM.log	-1872.9185	-1872.8042	-1872.7826	-1872.7817	-1872.8567	157.779
20_(P_III)_method_E_DCM.log	-1872.9054	-1872.7884	-1872.7673	-1872.7664	-1872.8400	155.085
20_(P_III)_method_E_DCM_smd.log	-1872.9089	-1872.7919	-1872.7709	-1872.7700	-1872.8434	154.585
20_(P_V)_method_A.log	-1872.8426	-1872.7283	-1872.7073	-1872.7063	-1872.7801	155.334
20_(P_V)_method_A_DCM.log	-1872.8556	-1872.7416	-1872.7206	-1872.7197	-1872.7932	154.738
20_(P_V)_method_A_DCM_smd.log	-1872.8604	-1872.7465	-1872.7264	-1872.7255	-1872.7955	147.367
20_(P_V)_method_B.log	-1873.3820	-1873.2670	-1873.2462	-1873.2453	-1873.3185	154.046

20_(P_V)_method_B_DKM.log	-1873.3935	-1873.2789	-1873.2582	-1873.2572	-1873.3300	153.187
20_(P_V)_method_B_DMSO.log	-1873.3958	-1873.2813	-1873.2605	-1873.2596	-1873.3326	153.558
20_(P_V)_method_B_MeOH.log	-1873.3956	-1873.2811	-1873.2603	-1873.2593	-1873.3323	153.485
20_(P_V)_method_B_THF.log	-1873.3930	-1873.2784	-1873.2576	-1873.2567	-1873.3295	153.172
20_(P_V)_method_B_toluene.log	-1873.3880	-1873.2731	-1873.2524	-1873.2514	-1873.3243	153.411
20_(P_V)_method_B_water.log	-1873.3961	-1873.2816	-1873.2608	-1873.2598	-1873.3328	153.642
20_(P_V)_method_D_DCM.log	-1872.9172	-1872.8030	-1872.7820	-1872.7810	-1872.8557	157.152
20_(P_V)_method_E_DCM.log	-1872.9141	-1872.7970	-1872.7765	-1872.7756	-1872.8478	152.084
20_(P_V)_method_E_DCM_smd.log	-1872.9173	-1872.8003	-1872.7798	-1872.7788	-1872.8511	152.138

Figure S1. ΔG values for the $G_{P(V)}$–$G_{P(III)}$ tautomeric equilibrium of compounds 1–20 calculated by methods A (B3LYP/6-31+G(d,p)), B (B3LYP/6-311++G(3df,3pd)), C (B3LYP/cc-pVTZ), D (B3LYP-D3/6-31+G(d,p)), E (ωB97XD/6-311++G(3df,3pd)) considering the SMD implicit solvent effect of DCM by all methods.

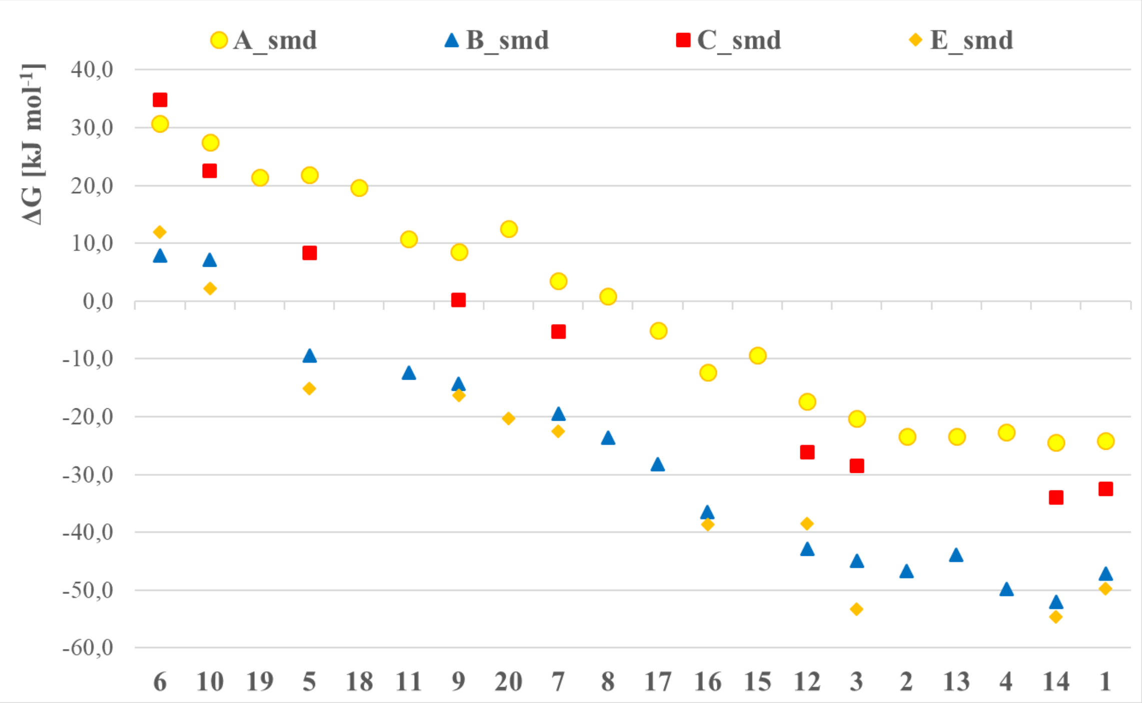

Table S2. Physical chemical descriptors of selected compounds with the "*a*" and "*b*" constants of the logarithmic equations ($y = a \ln(x) + b$) seen on Fig 3 and the corresponding Pearson correlation R^2.

ID	εHOMO (Hartree)	εLUMO (Hartree)	NBO	H (Hartree)	Parr-index (Hartree)	a	b	ε_0 – ΔG R^2
**1**	-0.27743	-0.01783	1.752	7.06389772	1.142231861	-4.186	-37.019	0.8359
**2**	-0.27175	-0.01574	1.763	6.966211307	1.098090877	-3.867	-37.756	0.7797
**3**	-0.30263	-0.01158	2.227	7.919674235	1.153777669	-1.542	-44.255	0.7198

**4**	-0.2668	-0.04533	1.7 91	6.026353729	1.496255798	-1.474	-47.193	0.7302
**6**	-0.3405	-0.04305	0.6 72	8.093822715	1.682208132	-0.903	14.023	0.9615
**12**	-0.26894	-0.05191	1.7 75	5.905538221	1.613370249	-4.352	-29.949	0.8761
**13**	-0.2668	-0.04533	1.7 91	6.026353729	1.496255798	-2.937	-38.065	0.8419
**14**	-0.29763	-0.01066	2.2 36	7.808654579	1.126502358	-2.259	-44.645	0.8518
**20**	-0.29139	-0.09124	1.7 62	5.446221605	2.488010268	-2.160	-11.855	0.8841

Figure S2. Stability of the P(V) form in various SMD implicit solvent models. Each point means the energy difference in Gibbs free energy between the P(V) and P(III) forms.

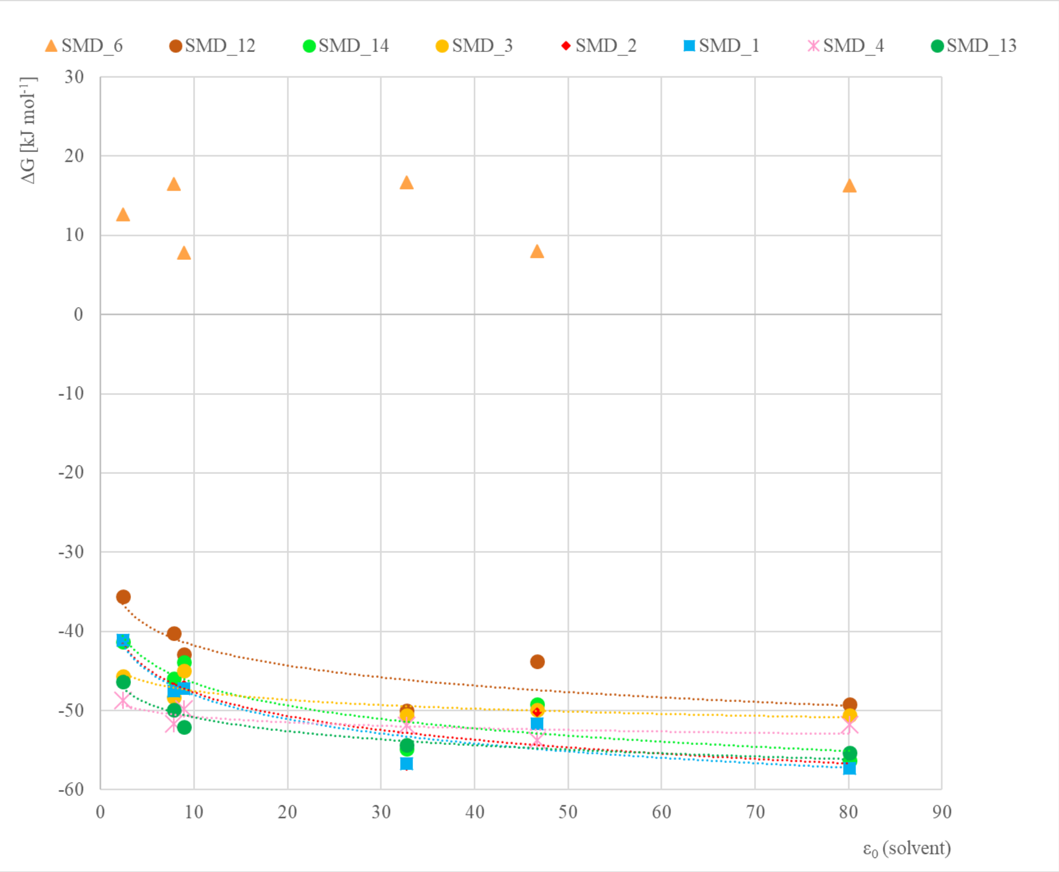

Table S3. Results of the t- and p-tests together with the confidence level.

ID	$\varepsilon_0 - \Delta G$ t-test	$\varepsilon_0 - \Delta G$ p-test	$\varepsilon_0 - \Delta G$ confidence level
**1**	5.046704477	0.003944322	99.6%
**2**	4.20669979861201	0.00843497205629798	99.2%
**3**	3.58390804281948	0.0158094371819169	98.4%
**4**	3.67862013644088	0.0143141687527487	98.6%
**6**	11.1745304093787	0.000100137648179403	100.0%
**12**	5.9460175832979	0.00192226403496411	99.8%
**13**	5.15999548906637	0.00358475100966536	99.6%

**14**	5.36080139838518	0.00303735026541283	99.7%
**20**	6.17581075498995	0.00162169128183819	99.8%

Table S4. Results of correlating the “*a*” and “*b*” constants with different descriptors.

	R^2
a – εHOMO	0.44
a – εLUMO	0.02
a – NBO	0.07
a – H	0.12
a – Parr	0.06
b – εHOMO	0.51
b – εLUMO	0.25
b – NBO	0.76
b – H	0.01
b – Parr	0.36

Table S5. Computed dipole moments (method B) in different solvents given in Debye.

		6		20		15		12		13		3		2		1	
Solvent	ε	III	V	III	V	III	V	III	V	III	V	III	V	III	V	III	V
vacuum	1	2.9297	2.4192	1.9555	2.7112	0.7822	4.1591	0.7352	4.3886	0.1083	4.151	1.3504	2.4321	0.2777	4.1128	0.2609	4.326
DCM	8.93	3.7345	3.2793	2.8575	3.7983	0.9693	5.6772	0.8966	5.9547	0.2355	5.6654	1.7126	3.244	0.503	5.4978	0.4312	5.5757
DMSO	46.7	3.8923	3.49	2.9442	4.0596	0.9858	6.0078	0.8853	6.2752	0.2882	5.9943	1.7877	3.4	0.5602	5.7965	0.4746	5.8171
MeOH	32.7	3.8747	3.4655	2.9335	4.0294	0.9838	5.9707	0.888	6.2308	0.2817	5.957	1.7795	3.3827	0.5536	5.7626	0.4696	5.7895
THF	7.85	3.6991	3.2341	2.5993	3.7433	0.9653	5.6066	0.8958	5.8868	0.2255	5.5934	1.6956	3.2097	0.4909	5.4323	0.4219	5.5215
PhMe	2.38	3.3487	2.8297	2.2821	3.2375	0.8963	4.9205	0.8448	5.2218	0.1526	4.9101	1.5321	2.8556	0.3812	4.8089	0.3391	4.979
H_2O	80.1	3.9087	3.5132	2.9577	4.0877	0.9884	6.0424	0.8824	6.2953	0.2947	6.0296	1.7955	3.4163	0.5665	5.8284	0.4795	5.8423

Table S6. Computed energies for the investigations of mechanistic pathways: (E), zero point energies, internal energies (U), enthalpies (H) and Gibbs free energies (G) given in Hartree as well as entropies (S) given in J mol^{-1} K^{-1} [at method **A** for molecules **1**, **3** and **6** in gas phase and also in solvent models].

Name	E	ZPE	U	H	G	S
1_pathway_a_(P_III).log	-497.0412	-496.9520	-496.9455	-496.9446	-496.9811	76.842
1_pathway_a_(P_TS).log	-496.9486	-496.8643	-496.8582	-496.8572	-496.8932	75.761
1_pathway_a_(P_V).log	-497.0441	-496.9553	-496.9494	-496.9485	-496.9840	74.720
1_pathway_b_(P_III).log	-649.9391	-649.7994	-649.7875	-649.7866	-649.8369	105.919
1_pathway_b_(P_TS).log	-649.9024	-649.7720	-649.7623	-649.7613	-649.8065	95.127
1_pathway_b_(P_V).log	-649.9438	-649.8053	-649.7935	-649.7925	-649.8437	107.647
1_pathway_b_acetone_(P_III).log	-649.9503	-649.8114	-649.7995	-649.7985	-649.8490	106.265
1_pathway_b_acetone_(P_TS).log	-649.9185	-649.7869	-649.7767	-649.7757	-649.8221	97.525
1_pathway_b_acetone_(P_V).log	-649.9605	-649.8234	-649.8111	-649.8101	-649.8645	114.429
1_pathway_b_CHCl3_(P_III).log	-649.9474	-649.8083	-649.7964	-649.7954	-649.8461	106.527
1_pathway_b_CHCl3_(P_TS).log	-649.9141	-649.7830	-649.7730	-649.7720	-649.8178	96.335

1_pathway_b_CHCl3_(P_V).log	-649.9556	-649.8182	-649.8059	-649.8049	-649.8590	113.757
1_pathway_b_DCM_(P_III).log	-649.9486	-649.8099	-649.7978	-649.7969	-649.8476	106.748
1_pathway_b_DCM_(P_TS).log	-649.9159	-649.7845	-649.7744	-649.7734	-649.8194	96.851
1_pathway_b_DCM_(P_V).log	-649.9585	-649.8214	-649.8099	-649.8090	-649.8602	107.878
1_pathway_b_DMSO_(P_III).log	-649.9506	-649.8120	-649.7999	-649.7990	-649.8498	107.009
1_pathway_b_DMSO_(P_TS).log	-649.9190	-649.7871	-649.7770	-649.7760	-649.8221	96.919
1_pathway_b_DMSO_(P_V).log	-649.9613	-649.8245	-649.8129	-649.8120	-649.8636	108.575
1_pathway_b_Et2O_2_(P_III).log	-649.9470	-649.8079	-649.7960	-649.7951	-649.8457	106.598
1_pathway_b_Et2O_2_(P_TS).log	-649.9135	-649.7825	-649.7725	-649.7715	-649.8173	96.256
1_pathway_b_Et2O_2_(P_V).log	-649.9550	-649.8175	-649.8052	-649.8043	-649.8587	114.535
1_pathway_b_EtOH_(P_III).log	-649.9502	-649.8115	-649.7995	-649.7985	-649.8494	107.142
1_pathway_b_EtOH_(P_TS).log	-649.9183	-649.7865	-649.7764	-649.7754	-649.8215	96.953
1_pathway_b_EtOH_(P_V).log	-649.9606	-649.8237	-649.8112	-649.8103	-649.8658	116.747
1_pathway_b_formamide_(P_III).log	-649.9509	-649.8123	-649.8003	-649.7993	-649.8502	106.990
1_pathway_b_formamide_(P_TS).log	-649.9195	-649.7875	-649.7774	-649.7764	-649.8225	96.957
1_pathway_b_formamide_(P_V).log	-649.9618	-649.8250	-649.8125	-649.8115	-649.8664	115.559
1_pathway_b_HCOOH_(P_III).log	-649.9507	-649.8120	-649.8000	-649.7991	-649.8499	107.003
1_pathway_b_HCOOH_(P_TS).log	-649.9191	-649.7872	-649.7770	-649.7761	-649.8222	96.928
1_pathway_b_HCOOH_(P_V).log	-649.9616	-649.8247	-649.8122	-649.8113	-649.8668	116.918
1_pathway_b_hex_(P_III).log	-649.9433	-649.8039	-649.7919	-649.7910	-649.8415	106.305
1_pathway_b_hex_(P_TS).log	-649.9080	-649.7775	-649.7676	-649.7667	-649.8122	95.873
1_pathway_b_hex_(P_V).log	-649.9499	-649.8117	-649.7998	-649.7988	-649.8500	107.647
1_pathway_b_MeCN_2_(P_III).log	-649.9507	-649.8119	-649.7999	-649.7990	-649.8495	106.271
1_pathway_b_MeCN_2_(P_TS).log	-649.9192	-649.7875	-649.7773	-649.7763	-649.8227	97.560
1_pathway_b_MeCN_2_(P_V).log	-649.9613	-649.8244	-649.8119	-649.8109	-649.8663	116.438
1_pathway_b_MeOH_(P_III).log	-649.9504	-649.8117	-649.7997	-649.7988	-649.8496	107.047
1_pathway_b_MeOH_(P_TS).log	-649.9187	-649.7868	-649.7767	-649.7758	-649.8218	96.888
1_pathway_b_MeOH_(P_V).log	-649.9610	-649.8241	-649.8125	-649.8116	-649.8632	108.651
1_pathway_b_THF_(P_III).log	-649.9487	-649.8097	-649.7977	-649.7968	-649.8473	106.344
1_pathway_b_THF_(P_TS).log	-649.9161	-649.7846	-649.7746	-649.7736	-649.8196	96.641
1_pathway_b_THF_(P_V).log	-649.9578	-649.8205	-649.8082	-649.8072	-649.8611	113.291
1_pathway_b_toluene_(P_III).log	-649.9445	-649.8052	-649.7933	-649.7924	-649.8430	106.507
1_pathway_b_toluene_(P_TS).log	-649.9098	-649.7792	-649.7692	-649.7683	-649.8139	95.961
1_pathway_b_toluene_(P_V).log	-649.9518	-649.8137	-649.8018	-649.8008	-649.8521	108.012
1_pathway_b_water_(P_III).log	-649.9508	-649.8122	-649.8002	-649.7992	-649.8501	107.046
1_pathway_b_water_(P_TS).log	-649.9194	-649.7874	-649.7773	-649.7763	-649.8224	96.953
1_pathway_b_water_(P_V).log	-649.9618	-649.8248	-649.8124	-649.8115	-649.8662	115.277
1_pathway_c_(P_III).log	-994.0941	-993.9135	-993.8993	-993.8984	-993.9567	122.689
1_pathway_c_(P_TS).log	-994.0511	-993.8786	-993.8656	-993.8646	-993.9180	112.327
1_pathway_c_(P_V).log	-994.0995	-993.9208	-993.9071	-993.9062	-993.9627	118.898
1_pathway_d_(P_III).log	-1491.1508	-1490.8805	-1490.8584	-1490.8574	-1490.9381	169.786
1_pathway_d_(P_TS).log	-1491.1085	-1490.8430	-1490.8221	-1490.8211	-1490.8946	154.581
1_pathway_d_(P_V).log	-1491.1554	-1490.8859	-1490.8650	-1490.8640	-1490.9398	159.484
1_pathway_e_(P_III).log	-1491.1511	-1490.8795	-1490.8584	-1490.8575	-1490.9324	157.627
1_pathway_e_(P_TS).log	-1491.1168	-1490.8555	-1490.8348	-1490.8338	-1490.9076	155.176
1_pathway_e_(P_V).log	-1491.1546	-1490.8852	-1490.8642	-1490.8633	-1490.9409	163.352

1_pathway_e_DCM_(P_III).log	-1491.1600	-1490.8891	-1490.8671	-1490.8662	-1490.9439	163.631
1_pathway_e_DCM_(P_TS).log	-1491.1348	-1490.8719	-1490.8511	-1490.8502	-1490.9242	155.738
1_pathway_e_DCM_(P_V).log	-1491.1712	-1490.9022	-1490.8803	-1490.8793	-1490.9591	167.902
1_pathway_e_DMSO_(P_III).log	-1491.1617	-1490.8909	-1490.8690	-1490.8680	-1490.9457	163.472
1_pathway_e_DMSO_(P_TS).log	-1491.1383	-1490.8753	-1490.8544	-1490.8535	-1490.9282	157.139
1_pathway_e_DMSO_(P_V).log	-1491.1745	-1490.9057	-1490.8837	-1490.8828	-1490.9637	170.321
1_pathway_e_MeOH_(P_III).log	-1491.1615	-1490.8907	-1490.8688	-1490.8678	-1490.9455	163.441
1_pathway_e_MeOH_(P_TS).log	-1491.1379	-1490.8750	-1490.8541	-1490.8531	-1490.9281	157.807
1_pathway_e_MeOH_(P_V).log	-1491.1741	-1490.9052	-1490.8833	-1490.8824	-1490.9624	168.458
1_pathway_e_THF_(P_III).log	-1491.1597	-1490.8886	-1490.8667	-1490.8658	-1490.9435	163.570
1_pathway_e_THF_(P_TS).log	-1491.1340	-1490.8712	-1490.8504	-1490.8494	-1490.9234	155.600
1_pathway_e_THF_(P_V).log	-1491.1705	-1490.9014	-1490.8795	-1490.8785	-1490.9585	168.380
1_pathway_e_toluene_(P_III).log	-1491.1559	-1490.8845	-1490.8626	-1490.8617	-1490.9396	164.052
1_pathway_e_toluene_(P_TS).log	-1491.1263	-1490.8638	-1490.8432	-1490.8422	-1490.9153	153.909
1_pathway_e_toluene_(P_V).log	-1491.1628	-1490.8937	-1490.8717	-1490.8708	-1490.9512	169.240
1_pathway_e_water_(P_III).log	-1491.1618	-1490.8911	-1490.8691	-1490.8682	-1490.9459	163.498
1_pathway_e_water_(P_TS).log	-1491.1387	-1490.8757	-1490.8548	-1490.8539	-1490.9293	158.671
1_pathway_e_water_(P_V).log	-1491.1748	-1490.9060	-1490.8841	-1490.8831	-1490.9635	169.252
3_pathway_a_(P_III).log	-647.5086	-647.4085	-647.4000	-647.3990	-647.4417	89.858
3_pathway_a_(P_TS).log	-647.4176	-647.3224	-647.3139	-647.3130	-647.3560	90.461
3_pathway_a_(P_V).log	-647.5143	-647.4141	-647.4059	-647.4049	-647.4477	90.037
3_pathway_b_(P_III).log	-800.4059	-800.2557	-800.2415	-800.2405	-800.2975	119.877
3_pathway_b_(P_TS).log	-800.3699	-800.2266	-800.2147	-800.2138	-800.2651	108.124
3_pathway_b_(P_V).log	-800.4117	-800.2617	-800.2476	-800.2467	-800.3035	119.536
3_pathway_c_(P_III).log	-1295.0157	-1294.8144	-1294.7954	-1294.7944	-1294.8658	150.197
3_pathway_c_(P_TS).log	-1294.9763	-1294.7810	-1294.7634	-1294.7625	-1294.8295	141.051
3_pathway_c_(P_V).log	-1295.0353	-1294.8338	-1294.8155	-1294.8145	-1294.8840	146.235
3_pathway_d_(P_III).log	-1942.5539	-1942.2510	-1942.2221	-1942.2212	-1942.3201	208.231
3_pathway_d__(P_TS).log	-1942.5054	-1942.2118	-1942.1847	-1942.1837	-1942.2764	195.114
3_pathway_d__(P_V).log	-1942.5624	-1942.2592	-1942.2306	-1942.2296	-1942.3277	206.416
3_pathway_e_(P_III).log	-1942.5393	-1942.2366	-1942.2075	-1942.2065	-1942.3045	206.153
3_pathway_e_(P_TS).log	-1942.5140	-1942.2175	-1942.1899	-1942.1890	-1942.2814	194.559
3_pathway_e_(P_V).log	-1942.5589	-1942.2559	-1942.2274	-1942.2265	-1942.3225	202.137
6_pathway_a_(P_III).log	-1092.4838	-1092.4404	-1092.4300	-1092.4290	-1092.4778	102.684
6_pathway_a_(P_TS).log	-1092.3746	-1092.3365	-1092.3261	-1092.3251	-1092.3746	104.042
6_pathway_a_(P_V).log	-1092.4679	-1092.4253	-1092.4152	-1092.4143	-1092.4629	102.346
6_pathway_b_(P_III).log	-1245.3643	-1245.2726	-1245.2562	-1245.2553	-1245.3204	137.144
6_pathway_b_(P_TS).log	-1245.3440	-1245.2555	-1245.2412	-1245.2403	-1245.2991	123.644
6_pathway_b_(P_V).log	-1245.3830	-1245.2901	-1245.2735	-1245.2726	-1245.3372	135.988
6_pathway_c_(P_III).log	-2184.9707	-2184.8828	-2184.8598	-2184.8589	-2184.9432	177.541
6_pathway_c_(P_TS).log	-2184.9103	-2184.8309	-2184.8090	-2184.8080	-2184.8888	170.064
6_pathway_c_(P_V).log	-2184.9478	-2184.8615	-2184.8394	-2184.8384	-2184.9189	169.237
6_pathway_e_(P_III).log	-3277.4677	-3277.3349	-3277.2999	-3277.2990	-3277.4158	245.757
6_pathway_e_(P_TS).log	-3277.4237	-3277.3003	-3277.2668	-3277.2659	-3277.3763	232.448
6_pathway_e_(P_V).log	-3277.4503	-3277.3195	-3277.2850	-3277.2841	-3277.3981	240.015
1_pathway_e_DCM_(P_III)_smd.log	-1491.1638	-1490.8929	-1490.8711	-1490.8701	-1490.9475	162.943

1_pathway_e_DCM_(P_TS)_smd.log	-1491.1406	-1490.8780	-1490.8572	-1490.8562	-1490.9306	156.655
1_pathway_e_DCM_(P_V)_smd.log	-1491.1761	-1490.9074	-1490.8855	-1490.8845	-1490.9650	169.460
1_pathway_e_DMSO_(P_III)_smd.log	-1491.1599	-1490.8887	-1490.8670	-1490.8661	-1490.9425	160.732
1_pathway_e_DMSO_(P_TS)_smd.log	-1491.1386	-1490.8725	-1490.8519	-1490.8509	-1490.9265	159.119
1_pathway_e_DMSO_(P_V)_smd.log	-1491.1731	-1490.9046	-1490.8826	-1490.8816	-1490.9645	174.278
1_pathway_e_MeOH_(P_III)_smd.log	-1491.1670	-1490.8967	-1490.8749	-1490.8739	-1490.9510	162.255
1_pathway_e_MeOH_(P_TS)_smd.log	-1491.1466	-1490.8817	-1490.8609	-1490.8599	-1490.9347	157.273
1_pathway_e_MeOH_(P_V)_smd.log	-1491.1888	-1490.9204	-1490.8984	-1490.8975	-1490.9783	170.136
1_pathway_e_THF_(P_III)_smd.log	-1491.1618	-1490.8905	-1490.8687	-1490.8678	-1490.9446	161.606
1_pathway_e_THF_(P_TS)_smd.log	-1491.1374	-1490.8748	-1490.8540	-1490.8530	-1490.9285	158.954
1_pathway_e_THF_(P_V)_smd.log	-1491.1726	-1490.9037	-1490.8818	-1490.8809	-1490.9616	169.981
1_pathway_e_toluene_(P_III)_smd.log	-1491.1587	-1490.8869	-1490.8651	-1490.8642	-1490.9416	162.989
1_pathway_e_toluene_(P_TS)_smd.log	-1491.1295	-1490.8670	-1490.8463	-1490.8454	-1490.9198	156.565
1_pathway_e_toluene_(P_V)_smd.log	-1491.1655	-1490.8960	-1490.8741	-1490.8731	-1490.9543	170.705
1_pathway_e_water_(P_III)_smd.log	-1491.1571	-1490.8866	-1490.8648	-1490.8639	-1490.9412	162.607
1_pathway_e_water_(P_TS)_smd.log	-1491.1370	-1490.8723	-1490.8514	-1490.8505	-1490.9243	155.380
1_pathway_e_water_(P_V)_smd.log	-1491.1791	-1490.9106	-1490.8887	-1490.8878	-1490.9677	168.261

Thermodynamics

1_(P_III)_method_A.log

```
                    Input orientation:
 ---------------------------------------------------------------------
 Center     Atomic      Atomic             Coordinates (Angstroms)
 Number     Number       Type             X           Y           Z
 ---------------------------------------------------------------------
      1         15           0       -0.511783    0.530189   -0.085708
      2          8           0        0.358803    1.260239    1.176608
      3          1           0       -0.245118    1.636851    1.828367
      4          6           0        0.358531    1.330314   -1.514278
      5          1           0        0.075107    0.826291   -2.445133
      6          1           0        0.055333    2.378614   -1.588090
      7          1           0        1.446382    1.281750   -1.393324
      8          6           0        0.359999   -1.106273   -0.105979
      9          1           0        0.058186   -1.693531    0.766000
     10          1           0        0.076144   -1.661760   -1.006932
     11          1           0        1.447758   -0.976289   -0.088807
 ---------------------------------------------------------------------
```

1_(P_III)_method_A_DCM.log

```
                    Input orientation:
 ---------------------------------------------------------------------
 Center     Atomic      Atomic             Coordinates (Angstroms)
 Number     Number       Type             X           Y           Z
 ---------------------------------------------------------------------
      1         15           0       -0.000014    0.095467   -0.554879
      2          8           0       -0.000169    1.488876    0.421943
      3          1           0       -0.000082    2.282910   -0.129260
      4          6           0        1.409856   -0.805523    0.238753
      5          1           0        1.440696   -1.833046   -0.140288
      6          1           0        2.352470   -0.316051   -0.022515
      7          1           0        1.305188   -0.828500    1.329277
      8          6           0       -1.409666   -0.805803    0.238812
      9          1           0       -2.352381   -0.316489   -0.022389
     10          1           0       -1.440345   -1.833318   -0.140264
     11          1           0       -1.304937   -0.828800    1.329329
 ---------------------------------------------------------------------
```

1_(P_III)_method_A_DCM_smd.log

```
                    Input orientation:
 ---------------------------------------------------------------------
 Center     Atomic      Atomic             Coordinates (Angstroms)
 Number     Number       Type             X           Y           Z
 ---------------------------------------------------------------------
      1         15           0       -0.000017    0.094374   -0.548604
      2          8           0       -0.000177    1.490971    0.421541
      3          1           0       -0.000067    2.285141   -0.133802
      4          6           0        1.409444   -0.805305    0.239729
      5          1           0        1.438392   -1.832618   -0.142529
      6          1           0        2.352470   -0.315508   -0.024010
      7          1           0        1.309163   -0.831310    1.331355
      8          6           0       -1.409257   -0.805584    0.239793
      9          1           0       -2.352388   -0.315966   -0.023904
     10          1           0       -1.438024   -1.832900   -0.142466
     11          1           0       -1.308925   -0.831572    1.331415
 ---------------------------------------------------------------------
```

1_(P_III)_method_B.log

```
                    Input orientation:
 ---------------------------------------------------------------------
 Center     Atomic      Atomic             Coordinates (Angstroms)
 Number     Number       Type             X           Y           Z
 ---------------------------------------------------------------------
      1         15           0       -0.000011    0.102236   -0.547108
      2          8           0       -0.000155    1.468936    0.419412
      3          1           0       -0.000105    2.262791   -0.120067
      4          6           0        1.397567   -0.801394    0.238382
      5          1           0        1.420361   -1.825695   -0.137004
      6          1           0        2.337994   -0.321090   -0.028420
      7          1           0        1.300098   -0.818325    1.325044
```

8	6	0	-1.397376	-0.801662	0.238442
9	1	0	-2.337904	-0.321528	-0.028313
10	1	0	-1.419998	-1.825965	-0.136949
11	1	0	-1.299856	-0.818582	1.325101

1_(P_III)_method_B_DCM_smd.log

Input orientation:

Center Number	Atomic Number	Atomic Type	Coordinates (Angstroms) X	Y	Z
1	15	0	-0.000017	0.098016	-0.531952
2	8	0	-0.000161	1.477104	0.416402
3	1	0	-0.000071	2.267725	-0.134382
4	6	0	1.399819	-0.800936	0.241054
5	1	0	1.422306	-1.822958	-0.142433
6	1	0	2.338984	-0.317045	-0.028449
7	1	0	1.309587	-0.829993	1.328895
8	6	0	-1.399637	-0.801207	0.241122
9	1	0	-2.338912	-0.317519	-0.028360
10	1	0	-1.421922	-1.823243	-0.142340
11	1	0	-1.309362	-0.830222	1.328961

1_(P_III)_method_B_DKM.log

Input orientation:

Center Number	Atomic Number	Atomic Type	Coordinates (Angstroms) X	Y	Z
1	15	0	-0.000014	0.099408	-0.538537
2	8	0	-0.000164	1.475630	0.416054
3	1	0	-0.000084	2.265901	-0.131084
4	6	0	1.399542	-0.801334	0.240596
5	1	0	1.421152	-1.823005	-0.141498
6	1	0	2.338674	-0.317928	-0.025291
7	1	0	1.301718	-0.827709	1.327096
8	6	0	-1.399354	-0.801609	0.240658
9	1	0	-2.338589	-0.318373	-0.025178
10	1	0	-1.420792	-1.823278	-0.141449
11	1	0	-1.301474	-0.827981	1.327152

1_(P_III)_method_B_DMSO.log

Input orientation:

Center Number	Atomic Number	Atomic Type	Coordinates (Angstroms) X	Y	Z
1	15	0	-0.000015	0.098808	-0.537181
2	8	0	-0.000167	1.476697	0.415635
3	1	0	-0.000078	2.266474	-0.132596
4	6	0	1.399947	-0.801279	0.240909
5	1	0	1.421070	-1.822596	-0.142016
6	1	0	2.338855	-0.317473	-0.025116
7	1	0	1.302184	-0.829150	1.327409
8	6	0	-1.399760	-0.801554	0.240971
9	1	0	-2.338772	-0.317925	-0.025009
10	1	0	-1.420705	-1.822873	-0.141955
11	1	0	-1.301944	-0.829409	1.327468

1_(P_III)_method_B_DMSO_smd.log

Input orientation:

Center Number	Atomic Number	Atomic Type	Coordinates (Angstroms) X	Y	Z
1	15	0	-0.000015	0.097990	-0.528338
2	8	0	-0.000174	1.482837	0.414273
3	1	0	-0.000073	2.266707	-0.145207
4	6	0	1.398447	-0.800874	0.243024
5	1	0	1.419388	-1.820141	-0.147749
6	1	0	2.337887	-0.315055	-0.021689
7	1	0	1.302934	-0.837193	1.330173
8	6	0	-1.398259	-0.801154	0.243085
9	1	0	-2.337802	-0.315498	-0.021567
10	1	0	-1.419036	-1.820414	-0.147713
11	1	0	-1.302683	-0.837483	1.330227

1_(P_III)_method_B_MeOH.log

Input orientation:

Center Number	Atomic Number	Atomic Type	Coordinates (Angstroms) X	Y	Z
1	15	0	-0.000015	0.098881	-0.537329
2	8	0	-0.000167	1.476582	0.415681
3	1	0	-0.000079	2.266415	-0.132429
4	6	0	1.399898	-0.801286	0.240874
5	1	0	1.421069	-1.822642	-0.141960
6	1	0	2.338834	-0.317531	-0.025134
7	1	0	1.302126	-0.828987	1.327374
8	6	0	-1.399711	-0.801560	0.240936
9	1	0	-2.338752	-0.317983	-0.025026
10	1	0	-1.420705	-1.822919	-0.141900
11	1	0	-1.301885	-0.829249	1.327432

1_(P_III)_method_B_MeOH_smd.log

Input orientation:

Center Number	Atomic Number	Atomic Type	Coordinates (Angstroms) X	Y	Z
1	15	0	-0.000017	0.092154	-0.534072
2	8	0	-0.000167	1.477452	0.412507
3	1	0	-0.000065	2.267868	-0.138613
4	6	0	1.399859	-0.800809	0.242462
5	1	0	1.426067	-1.821010	-0.146093
6	1	0	2.337983	-0.311687	-0.021853
7	1	0	1.302555	-0.834736	1.329678
8	6	0	-1.399677	-0.801083	0.242529
9	1	0	-2.337910	-0.312158	-0.021759
10	1	0	-1.425689	-1.821296	-0.146007
11	1	0	-1.302325	-0.834972	1.329741

1_(P_III)_method_B_THF.log

Input orientation:

Center Number	Atomic Number	Atomic Type	Coordinates (Angstroms) X	Y	Z
1	15	0	-0.000014	0.099533	-0.538851
2	8	0	-0.000164	1.475383	0.416154
3	1	0	-0.000085	2.265766	-0.130733
4	6	0	1.399457	-0.801344	0.240524
5	1	0	1.421178	-1.823097	-0.141373
6	1	0	2.338635	-0.318023	-0.025341
7	1	0	1.301628	-0.827383	1.327026
8	6	0	-1.399270	-0.801619	0.240585
9	1	0	-2.338549	-0.318467	-0.025227
10	1	0	-1.420819	-1.823370	-0.141326
11	1	0	-1.301384	-0.827656	1.327082

1_(P_III)_method_B_THF_smd.log

Input orientation:

Center Number	Atomic Number	Atomic Type	Coordinates (Angstroms) X	Y	Z
1	15	0	-0.000014	0.097978	-0.531435
2	8	0	-0.000163	1.480186	0.414298
3	1	0	-0.000083	2.266212	-0.141141
4	6	0	1.399376	-0.800887	0.242551
5	1	0	1.422843	-1.821033	-0.146209
6	1	0	2.338532	-0.314881	-0.023064
7	1	0	1.306148	-0.834900	1.329970
8	6	0	-1.399190	-0.801158	0.242616
9	1	0	-2.338452	-0.315340	-0.022966
10	1	0	-1.422471	-1.821312	-0.146133
11	1	0	-1.305911	-0.835142	1.330031

1_(P_III)_method_B_toluene.log

Input orientation:

Center Number	Atomic Number	Atomic Type	Coordinates (Angstroms) X	Y	Z
1	15	0	-0.000013	0.100754	-0.542234
2	8	0	-0.000159	1.472776	0.417353
3	1	0	-0.000096	2.264419	-0.126738
4	6	0	1.398626	-0.801413	0.239717
5	1	0	1.421171	-1.824106	-0.139842
6	1	0	2.338293	-0.319128	-0.026208
7	1	0	1.300874	-0.823840	1.326265
8	6	0	-1.398437	-0.801685	0.239777
9	1	0	-2.338204	-0.319567	-0.026094
10	1	0	-1.420813	-1.824376	-0.139798
11	1	0	-1.300629	-0.824113	1.326320

1_(P_III)_method_B_toluene_smd.log

Input orientation:

Center Number	Atomic Number	Atomic Type	Coordinates (Angstroms) X	Y	Z
1	15	0	-0.000013	0.099179	-0.537689
2	8	0	-0.000160	1.475625	0.415918
3	1	0	-0.000094	2.264165	-0.133871
4	6	0	1.398646	-0.800700	0.241131
5	1	0	1.425012	-1.822511	-0.142943
6	1	0	2.338233	-0.316108	-0.024972
7	1	0	1.307023	-0.829680	1.328724
8	6	0	-1.398458	-0.800972	0.241192
9	1	0	-2.338147	-0.316552	-0.024865
10	1	0	-1.424648	-1.822784	-0.142887
11	1	0	-1.306781	-0.829941	1.328781

1_(P_III)_method_B_water.log

Input orientation:

Center Number	Atomic Number	Atomic Type	Coordinates (Angstroms) X	Y	Z
1	15	0	-0.000015	0.098737	-0.537043
2	8	0	-0.000167	1.476804	0.415591
3	1	0	-0.000077	2.266528	-0.132755
4	6	0	1.399994	-0.801273	0.240942
5	1	0	1.421075	-1.822551	-0.142070
6	1	0	2.338874	-0.317414	-0.025097
7	1	0	1.302241	-0.829304	1.327443
8	6	0	-1.399807	-0.801547	0.241005
9	1	0	-2.338792	-0.317868	-0.024991
10	1	0	-1.420710	-1.822829	-0.142007
11	1	0	-1.302002	-0.829561	1.327502

1_(P_III)_method_B_water_smd.log

Input orientation:

Center Number	Atomic Number	Atomic Type	Coordinates (Angstroms) X	Y	Z
1	15	0	-0.000018	0.094141	-0.535747
2	8	0	-0.000168	1.479448	0.413432
3	1	0	-0.000062	2.268329	-0.139389
4	6	0	1.397442	-0.800862	0.242132
5	1	0	1.420297	-1.820688	-0.146181
6	1	0	2.337087	-0.314539	-0.019466
7	1	0	1.295091	-0.834377	1.328469
8	6	0	-1.397260	-0.801135	0.242200
9	1	0	-2.337015	-0.315011	-0.019372
10	1	0	-1.419919	-1.820974	-0.146093
11	1	0	-1.294862	-0.834609	1.328534

1_(P_III)_method_C_DCM.log

Input orientation:

Center Number	Atomic Number	Atomic Type	Coordinates (Angstroms) X	Y	Z
1	15	0	-0.000015	0.099232	-0.543969
2	8	0	-0.000174	1.482210	0.420287

3	1	0	-0.000073	2.270184	-0.131299
4	6	0	1.404468	-0.803379	0.240280
5	1	0	1.426838	-1.825472	-0.140451
6	1	0	2.343310	-0.318905	-0.024653
7	1	0	1.303249	-0.827544	1.326447
8	6	0	-1.404279	-0.803662	0.240337
9	1	0	-2.343221	-0.319339	-0.024520
10	1	0	-1.426494	-1.825742	-0.140437
11	1	0	-1.302994	-0.827857	1.326498

1_(P_III)_method_C_DCM_smd.log

Input orientation:

Center Number	Atomic Number	Atomic Type	Coordinates (Angstroms) X	Y	Z
1	15	0	-0.000001	0.098764	-0.544815
2	8	0	-0.000107	1.481241	0.418362
3	1	0	0.000040	2.272012	-0.133745
4	6	0	1.404344	-0.804831	0.231626
5	1	0	1.426797	-1.825319	-0.156333
6	1	0	2.343379	-0.317809	-0.033222
7	1	0	1.307286	-0.836379	1.318863
8	6	0	-1.404256	-0.804966	0.231621
9	1	0	-2.343337	-0.318034	-0.033233
10	1	0	-1.426608	-1.825457	-0.156336
11	1	0	-1.307201	-0.836501	1.318858

1_(P_III)_method_D_DCM.log

Input orientation:

Center Number	Atomic Number	Atomic Type	Coordinates (Angstroms) X	Y	Z
1	15	0	0.000004	0.099477	-0.560560
2	8	0	-0.000084	1.486980	0.423424
3	1	0	0.000006	2.283054	-0.124354
4	6	0	1.404293	-0.805745	0.233167
5	1	0	1.428838	-1.833738	-0.144368
6	1	0	2.349845	-0.321336	-0.025793
7	1	0	1.294778	-0.826077	1.323063
8	6	0	-1.404208	-0.805865	0.233161
9	1	0	-2.349802	-0.321547	-0.025817
10	1	0	-1.428652	-1.833866	-0.144357
11	1	0	-1.294702	-0.826169	1.323059

1_(P_III)_method_E_DCM.log

Input orientation:

Center Number	Atomic Number	Atomic Type	Coordinates (Angstroms) X	Y	Z
1	15	0	-0.000012	0.097927	-0.535891
2	8	0	-0.000155	1.467682	0.402417
3	1	0	-0.000097	2.251878	-0.146135
4	6	0	1.388713	-0.797752	0.242861
5	1	0	1.418846	-1.814451	-0.152076
6	1	0	2.329123	-0.307060	-0.004095
7	1	0	1.274556	-0.838984	1.327252
8	6	0	-1.388523	-0.798028	0.242918
9	1	0	-2.329029	-0.307478	-0.003953
10	1	0	-1.418514	-1.814707	-0.152077
11	1	0	-1.274294	-0.839304	1.327300

1_(P_III)_method_E_DCM_smd.log

Input orientation:

Center Number	Atomic Number	Atomic Type	Coordinates (Angstroms) X	Y	Z
1	15	0	0.000001	0.103822	-0.535473
2	8	0	-0.000052	1.463508	0.415471
3	1	0	0.000064	2.255621	-0.126152
4	6	0	1.389277	-0.800021	0.225935
5	1	0	1.420015	-1.811029	-0.185971
6	1	0	2.329303	-0.303913	-0.017127
7	1	0	1.280099	-0.858819	1.310824
8	6	0	-1.389237	-0.800077	0.225930
9	1	0	-2.329279	-0.303992	-0.017116
10	1	0	-1.419946	-1.811078	-0.185994
11	1	0	-1.280051	-0.858893	1.310818

1_(P_V)_method_A.log

Input orientation:

Center Number	Atomic Number	Atomic Type	Coordinates (Angstroms) X	Y	Z
1	15	0	-1.097642	0.661894	0.134284
2	1	0	-0.719723	1.196617	-1.128103
3	8	0	-0.563645	1.416828	1.319107
4	6	0	-2.921533	0.604068	0.007978
5	1	0	-3.310751	1.625793	0.001564
6	1	0	-3.240351	0.089581	-0.904558
7	1	0	-3.328889	0.084192	0.880157
8	6	0	-0.544228	-1.076946	0.007624
9	1	0	-0.923310	-1.548939	-0.904835
10	1	0	0.548801	-1.103328	0.000856
11	1	0	-0.898304	-1.634378	0.879866

1_(P_V)_method_A_DCM.log

Input orientation:

Center Number	Atomic Number	Atomic Type	Coordinates (Angstroms) X	Y	Z
1	15	0	0.000000	0.182796	0.278693
2	1	0	0.000006	0.158221	1.694838
3	8	0	-0.000010	1.587469	-0.295373
4	6	0	1.459073	-0.815734	-0.162858
5	1	0	2.364631	-0.291776	0.154172
6	1	0	1.416905	-1.792908	0.327810
7	1	0	1.490954	-0.955669	-1.247203
8	6	0	-1.459068	-0.815744	-0.162854
9	1	0	-1.416842	-1.792964	0.327720
10	1	0	-2.364624	-0.291842	0.154274

11	1	0	-1.491005	-0.955582	-1.247209

1_(P_V)_method_A_DCM_smd.log

Input orientation:

Center Number	Atomic Number	Atomic Type	Coordinates (Angstroms) X	Y	Z
1	15	0	-0.000000	0.181069	0.281974
2	1	0	0.000006	0.151634	1.698309
3	8	0	-0.000010	1.589701	-0.285768
4	6	0	1.456582	-0.814940	-0.164367
5	1	0	2.364335	-0.286745	0.143190
6	1	0	1.419507	-1.789545	0.334021
7	1	0	1.479234	-0.961807	-1.249126
8	6	0	-1.456577	-0.814954	-0.164353
9	1	0	-1.419464	-1.789578	0.333995
10	1	0	-2.364331	-0.286791	0.143255
11	1	0	-1.479262	-0.961778	-1.249118

1_(P_V)_method_B.log

Input orientation:

Center Number	Atomic Number	Atomic Type	Coordinates (Angstroms) X	Y	Z
1	15	0	0.000000	0.191548	0.269670
2	1	0	0.000004	0.137289	1.685161
3	8	0	-0.000008	1.561289	-0.292768
4	6	0	1.446831	-0.813191	-0.160364
5	1	0	2.347052	-0.284932	0.149508
6	1	0	1.411343	-1.785672	0.331224
7	1	0	1.480406	-0.953107	-1.240412
8	6	0	-1.446826	-0.813203	-0.160356
9	1	0	-1.411289	-1.785715	0.331168
10	1	0	-2.347048	-0.284989	0.149591
11	1	0	-1.480445	-0.953049	-1.240411

1_(P_V)_method_B_DCM_smd.log

Input orientation:

Center Number	Atomic Number	Atomic Type	Coordinates (Angstroms) X	Y	Z
1	15	0	0.000000	0.174787	0.275124
2	1	0	0.000006	0.145048	1.686811
3	8	0	-0.000010	1.560071	-0.286056
4	6	0	1.446080	-0.810702	-0.163015
5	1	0	2.348031	-0.283133	0.147156
6	1	0	1.410040	-1.781593	0.332345
7	1	0	1.473042	-0.956363	-1.243443
8	6	0	-1.446075	-0.810714	-0.163004
9	1	0	-1.409990	-1.781633	0.332299
10	1	0	-2.348027	-0.283185	0.147232
11	1	0	-1.473077	-0.956316	-1.243439

1_(P_V)_method_B_DKM.log

Input orientation:

Center Number	Atomic Number	Atomic Type	Coordinates (Angstroms) X	Y	Z
1	15	0	0.000000	0.179192	0.270935
2	1	0	0.000007	0.154189	1.682365
3	8	0	-0.000010	1.561370	-0.295103
4	6	0	1.446940	-0.811734	-0.161905
5	1	0	2.347794	-0.292076	0.160123
6	1	0	1.398446	-1.785165	0.325597
7	1	0	1.483346	-0.950237	-1.241932
8	6	0	-1.446935	-0.811747	-0.161894
9	1	0	-1.398400	-1.785204	0.325553
10	1	0	-2.347789	-0.292127	0.160197
11	1	0	-1.483380	-0.950193	-1.241926

1_(P_V)_method_B_DMSO.log

Input orientation:

Center Number	Atomic Number	Atomic Type	Coordinates (Angstroms) X	Y	Z
1	15	0	0.000000	0.173995	0.273819
2	1	0	0.000006	0.151098	1.684311
3	8	0	-0.000010	1.559807	-0.289987
4	6	0	1.448530	-0.811186	-0.162944
5	1	0	2.348350	-0.286606	0.154015
6	1	0	1.406593	-1.782868	0.328424
7	1	0	1.479745	-0.953628	-1.242585
8	6	0	-1.448525	-0.811199	-0.162932
9	1	0	-1.406546	-1.782907	0.328382
10	1	0	-2.348345	-0.286657	0.154088
11	1	0	-1.479779	-0.953584	-1.242580

1_(P_V)_method_B_DMSO_smd.log

Input orientation:

Center Number	Atomic Number	Atomic Type	Coordinates (Angstroms) X	Y	Z
1	15	0	-0.000000	0.188613	0.274887
2	1	0	0.000003	0.160138	1.686549
3	8	0	-0.000006	1.573779	-0.286413
4	6	0	1.445963	-0.798285	-0.161911
5	1	0	2.347910	-0.269652	0.146341
6	1	0	1.409141	-1.767789	0.336372
7	1	0	1.471841	-0.946677	-1.242053
8	6	0	-1.445959	-0.798294	-0.161904
9	1	0	-1.409120	-1.767805	0.336365
10	1	0	-2.347908	-0.269674	0.146369
11	1	0	-1.471851	-0.946671	-1.242047

1_(P_V)_method_B_MeOH.log

Input orientation:

Center Number	Atomic Number	Atomic Type	Coordinates (Angstroms) X	Y	Z
1	15	0	0.000000	0.174636	0.272939
2	1	0	0.000008	0.152661	1.683548
3	8	0	-0.000012	1.559534	-0.292399
4	6	0	1.448481	-0.811445	-0.162561
5	1	0	2.348151	-0.287997	0.156713
6	1	0	1.404734	-1.783839	0.327174
7	1	0	1.481630	-0.951965	-1.242401
8	6	0	-1.448476	-0.811461	-0.162544
9	1	0	-1.404699	-1.783866	0.327165
10	1	0	-2.348148	-0.288035	0.156764
11	1	0	-1.481650	-0.951955	-1.242387

1_(P_V)_method_B_MeOH_smd.log

Input orientation:

Center Number	Atomic Number	Atomic Type	Coordinates (Angstroms) X	Y	Z
1	15	0	-0.000000	0.157696	0.281711
2	1	0	0.000006	0.151440	1.689694
3	8	0	-0.000010	1.553892	-0.282345
4	6	0	1.448683	-0.810078	-0.164684
5	1	0	2.347604	-0.276047	0.143054
6	1	0	1.417253	-1.779997	0.333115
7	1	0	1.468785	-0.957227	-1.245038
8	6	0	-1.448678	-0.810092	-0.164671
9	1	0	-1.417209	-1.780031	0.333086
10	1	0	-2.347601	-0.276094	0.143120
11	1	0	-1.468814	-0.957196	-1.245031

1_(P_V)_method_B_THF.log

Input orientation:

Center Number	Atomic Number	Atomic Type	Coordinates (Angstroms) X	Y	Z
1	15	0	0.000000	0.180119	0.270523
2	1	0	0.000007	0.154243	1.682153
3	8	0	-0.000010	1.561636	-0.295613
4	6	0	1.446709	-0.811829	-0.161754
5	1	0	2.347716	-0.292669	0.160645
6	1	0	1.397577	-1.785437	0.325379
7	1	0	1.483712	-0.949900	-1.241820
8	6	0	-1.446704	-0.811842	-0.161742
9	1	0	-1.397530	-1.785476	0.325336
10	1	0	-2.347710	-0.292720	0.160719
11	1	0	-1.483746	-0.949856	-1.241815

1_(P_V)_method_B_THF_smd.log

Input orientation:

Center Number	Atomic Number	Atomic Type	Coordinates (Angstroms) X	Y	Z
1	15	0	-0.000000	0.179580	0.273625
2	1	0	0.000004	0.144408	1.685909
3	8	0	-0.000009	1.563711	-0.285068
4	6	0	1.444625	-0.811017	-0.163007
5	1	0	2.347765	-0.285946	0.147542
6	1	0	1.404721	-1.781769	0.332643
7	1	0	1.472286	-0.956956	-1.243426
8	6	0	-1.444620	-0.811030	-0.162998
9	1	0	-1.404668	-1.781810	0.332593
10	1	0	-2.347760	-0.286000	0.147622
11	1	0	-1.472324	-0.956904	-1.243424

1_(P_V)_method_B_toluene.log

Input orientation:

Center Number	Atomic Number	Atomic Type	Coordinates (Angstroms) X	Y	Z
1	15	0	0.000000	0.186139	0.268614
2	1	0	0.000008	0.149024	1.682102
3	8	0	-0.000012	1.561963	-0.296724
4	6	0	1.446363	-0.812645	-0.160755
5	1	0	2.347238	-0.291698	0.159251
6	1	0	1.400034	-1.786578	0.326445
7	1	0	1.484737	-0.949473	-1.240966
8	6	0	-1.446358	-0.812661	-0.160740
9	1	0	-1.399997	-1.786608	0.326430
10	1	0	-2.347234	-0.291740	0.159307
11	1	0	-1.484760	-0.949456	-1.240953

1_(P_V)_method_B_toluene_smd.log

Input orientation:

Center Number	Atomic Number	Atomic Type	Coordinates (Angstroms) X	Y	Z
1	15	0	-0.000000	0.184120	0.272270
2	1	0	0.000006	0.139469	1.685950
3	8	0	-0.000010	1.562377	-0.287010
4	6	0	1.445495	-0.811463	-0.161764
5	1	0	2.347859	-0.283785	0.146379
6	1	0	1.411806	-1.783117	0.332864
7	1	0	1.476667	-0.956455	-1.242106
8	6	0	-1.445490	-0.811476	-0.161751
9	1	0	-1.411760	-1.783152	0.332829
10	1	0	-2.347855	-0.283834	0.146449
11	1	0	-1.476698	-0.956418	-1.242100

1_(P_V)_method_B_water.log

Input orientation:

Center Number	Atomic Number	Atomic Type	Coordinates (Angstroms) X	Y	Z
1	15	0	0.000000	0.173384	0.274111
2	1	0	0.000006	0.150755	1.684509
3	8	0	-0.000010	1.559544	-0.289544
4	6	0	1.448765	-0.811140	-0.163036

5	1	0	2.348408	-0.285945	0.153424
6	1	0	1.407659	-1.782664	0.328698
7	1	0	1.479479	-0.953929	-1.242644
8	6	0	-1.448760	-0.811153	-0.163024
9	1	0	-1.407613	-1.782701	0.328660
10	1	0	-2.348404	-0.285995	0.153496
11	1	0	-1.479511	-0.953889	-1.242638

1_(P_V)_method_B_water_smd.log

Input orientation:

Center Number	Atomic Number	Atomic Type	Coordinates (Angstroms) X	Y	Z
1	15	0	-0.000000	0.158622	0.282555
2	1	0	0.000006	0.153555	1.689374
3	8	0	-0.000010	1.556116	-0.282067
4	6	0	1.447010	-0.810091	-0.164836
5	1	0	2.347100	-0.279293	0.143062
6	1	0	1.411057	-1.779517	0.332573
7	1	0	1.464948	-0.957079	-1.244754
8	6	0	-1.447005	-0.810105	-0.164823
9	1	0	-1.411013	-1.779551	0.332544
10	1	0	-2.347096	-0.279340	0.143129
11	1	0	-1.464977	-0.957048	-1.244746

1_(P_V)_method_C_DCM.log

Input orientation:

Center Number	Atomic Number	Atomic Type	Coordinates (Angstroms) X	Y	Z
1	15	0	0.000000	0.178903	0.272699
2	1	0	0.000007	0.156770	1.687885
3	8	0	-0.000011	1.571902	-0.299729
4	6	0	1.454417	-0.814626	-0.162265
5	1	0	2.353401	-0.292487	0.161088
6	1	0	1.406414	-1.788447	0.324408
7	1	0	1.489620	-0.950065	-1.242675
8	6	0	-1.454412	-0.814639	-0.162254
9	1	0	-1.406366	-1.788488	0.324359
10	1	0	-2.353395	-0.292539	0.161165
11	1	0	-1.489656	-0.950016	-1.242670

1_(P_V)_method_C_DCM_smd.log

Input orientation:

Center Number	Atomic Number	Atomic Type	Coordinates (Angstroms) X	Y	Z
1	15	0	-0.000000	0.184413	0.279871
2	1	0	0.000004	0.151640	1.695102
3	8	0	-0.000008	1.583635	-0.280665
4	6	0	1.452961	-0.803015	-0.164341
5	1	0	2.353507	-0.273511	0.146574
6	1	0	1.416657	-1.774883	0.329479
7	1	0	1.476576	-0.944762	-1.245393
8	6	0	-1.452956	-0.803027	-0.164332
9	1	0	-1.416633	-1.774900	0.329476
10	1	0	-2.353505	-0.273538	0.146601
11	1	0	-1.476584	-0.944762	-1.245385

1_(P_V)_method_D_DCM.log

Input orientation:

Center Number	Atomic Number	Atomic Type	Coordinates (Angstroms) X	Y	Z
1	15	0	0.000000	0.194355	0.278471
2	1	0	0.000005	0.170910	1.694127
3	8	0	-0.000012	1.597458	-0.298012
4	6	0	1.452288	-0.809478	-0.163220
5	1	0	2.361979	-0.294845	0.156008
6	1	0	1.397652	-1.787267	0.324533
7	1	0	1.481562	-0.946264	-1.247834
8	6	0	-1.452278	-0.809495	-0.163214
9	1	0	-1.397592	-1.787315	0.324471
10	1	0	-2.361971	-0.294910	0.156088
11	1	0	-1.481596	-0.946210	-1.247835

1_(P_V)_method_E_DCM.log

Input orientation:

Center Number	Atomic Number	Atomic Type	Coordinates (Angstroms) X	Y	Z
1	15	0	0.000000	0.180812	0.272029
2	1	0	0.000008	0.154247	1.680890
3	8	0	-0.000011	1.556198	-0.292724
4	6	0	1.435571	-0.810213	-0.161699
5	1	0	2.340082	-0.295716	0.157392
6	1	0	1.379300	-1.782426	0.327045
7	1	0	1.465137	-0.949104	-1.241870
8	6	0	-1.435566	-0.810229	-0.161681
9	1	0	-1.379267	-1.782449	0.327044
10	1	0	-2.340078	-0.295752	0.157440
11	1	0	-1.465156	-0.949102	-1.241854

1_(P_V)_method_E_DCM_smd.log

Input orientation:

Center Number	Atomic Number	Atomic Type	Coordinates (Angstroms) X	Y	Z
1	15	0	-0.000000	0.188771	0.276339
2	1	0	0.000001	0.158837	1.685458
3	8	0	-0.000001	1.567216	-0.283763
4	6	0	1.434263	-0.798585	-0.161364
5	1	0	2.340168	-0.276994	0.146462
6	1	0	1.387838	-1.768187	0.335751
7	1	0	1.454674	-0.945185	-1.241893
8	6	0	-1.434263	-0.798586	-0.161361
9	1	0	-1.387835	-1.768189	0.335751
10	1	0	-2.340168	-0.276998	0.146470
11	1	0	-1.454678	-0.945183	-1.241890

2_(P_III)_method_A.log

Input orientation:

Center Number	Atomic Number	Atomic Type	Coordinates (Angstroms) X	Y	Z
1	15	0	-0.551760	0.592865	0.023977
2	8	0	0.433486	1.288935	1.222373
3	1	0	-0.106979	1.688507	1.914464
4	6	0	0.246833	1.358970	-1.474570
5	6	0	0.251938	-1.085677	-0.058937
6	1	0	-0.097864	0.783112	-2.344225
7	1	0	1.333373	1.220595	-1.400809
8	1	0	-0.093756	-1.554716	-0.990189
9	1	0	1.337846	-0.949536	-0.145121
10	6	0	-0.095756	2.843773	-1.649190
11	1	0	0.252815	3.429268	-0.792177
12	1	0	0.382860	3.252646	-2.545927
13	1	0	-1.176238	2.997085	-1.746943
14	6	0	-0.084775	-1.976815	1.143146
15	1	0	0.396592	-2.956655	1.050488
16	1	0	0.264867	-1.523535	2.076449
17	1	0	-1.164537	-2.141185	1.230349

2_(P_III)_method_A_DCM.log

Input orientation:

Center Number	Atomic Number	Atomic Type	Coordinates (Angstroms) X	Y	Z
1	15	0	0.000008	0.148455	-0.507160
2	8	0	-0.000031	1.545406	0.466116
3	1	0	-0.000151	2.339708	-0.083992
4	6	0	1.414269	-0.760843	0.290045
5	6	0	-1.414210	-0.760957	0.289994
6	1	0	1.340797	-1.804491	-0.043135
7	1	0	1.264725	-0.755876	1.377708
8	1	0	-1.340716	-1.804563	-0.043318
9	1	0	-1.264658	-0.756121	1.377656
10	6	0	2.789180	-0.183703	-0.070070
11	1	0	2.879833	0.857555	0.256713
12	1	0	3.588952	-0.753892	0.415087
13	1	0	2.965161	-0.214215	-1.151096
14	6	0	-2.789142	-0.183812	-0.070036
15	1	0	-3.588893	-0.754090	0.415052
16	1	0	-2.879824	0.857400	0.256886
17	1	0	-2.965131	-0.214186	-1.151065

2_(P_III)_method_A_DCM_smd.log

Input orientation:

Center Number	Atomic Number	Atomic Type	Coordinates (Angstroms) X	Y	Z
1	15	0	0.000007	0.153305	-0.495455
2	8	0	-0.000035	1.553223	0.471417
3	1	0	-0.000152	2.347434	-0.083055
4	6	0	1.415190	-0.757262	0.294375
5	6	0	-1.415131	-0.757374	0.294328
6	1	0	1.334405	-1.800192	-0.040617
7	1	0	1.272039	-0.752935	1.383242
8	1	0	-1.334321	-1.800265	-0.040784
9	1	0	-1.271969	-0.753166	1.383195
10	6	0	2.789612	-0.187283	-0.073766
11	1	0	2.891989	0.852927	0.255057
12	1	0	3.589438	-0.764597	0.404595
13	1	0	2.958481	-0.216324	-1.156617
14	6	0	-2.789575	-0.187393	-0.073732
15	1	0	-3.589379	-0.764800	0.404554
16	1	0	-2.891984	0.852769	0.255234
17	1	0	-2.958446	-0.216293	-1.156586

2_(P_III)_method_B.log

Input orientation:

Center Number	Atomic Number	Atomic Type	Coordinates (Angstroms) X	Y	Z
1	15	0	0.000004	0.138223	-0.505723
2	8	0	-0.000019	1.520823	0.441478
3	1	0	-0.000110	2.306928	-0.108831
4	6	0	1.403934	-0.762767	0.290845
5	6	0	-1.403881	-0.762850	0.290834
6	1	0	1.336506	-1.801324	-0.045114
7	1	0	1.253194	-0.761193	1.373607
8	1	0	-1.336413	-1.801389	-0.045173
9	1	0	-1.253137	-0.761318	1.373595
10	6	0	2.771588	-0.177108	-0.063663
11	1	0	2.852519	0.856762	0.272784
12	1	0	3.574168	-0.742589	0.411547
13	1	0	2.943505	-0.193484	-1.140791
14	6	0	-2.771561	-0.177231	-0.063642
15	1	0	-3.574117	-0.742770	0.411539
16	1	0	-2.852535	0.856618	0.272860
17	1	0	-2.943478	-0.193557	-1.140770

2_(P_III)_method_B_DCM.log

Input orientation:

Center Number	Atomic Number	Atomic Type	Coordinates (Angstroms) X	Y	Z
1	15	0	0.000006	0.147637	-0.489255
2	8	0	-0.000019	1.530578	0.457288
3	1	0	-0.000134	2.318835	-0.092212
4	6	0	1.405454	-0.757862	0.294255
5	6	0	-1.405397	-0.757951	0.294233
6	1	0	1.328566	-1.795060	-0.042669
7	1	0	1.262005	-0.758511	1.378037
8	1	0	-1.328475	-1.795126	-0.042758
9	1	0	-1.261943	-0.758663	1.378015
10	6	0	2.774813	-0.181770	-0.070092
11	1	0	2.866446	0.854201	0.257583
12	1	0	3.574641	-0.750626	0.405476
13	1	0	2.942068	-0.207607	-1.147809
14	6	0	-2.774781	-0.181889	-0.070068
15	1	0	-3.574585	-0.750812	0.405462
16	1	0	-2.866455	0.854055	0.257683
17	1	0	-2.942040	-0.207654	-1.147786

2_(P_III)_method_B_DCM_smd.log

Input orientation:

Center Number	Atomic Number	Atomic Type	Coordinates (Angstroms) X	Y	Z
1	15	0	0.000003	0.157323	-0.474495
2	8	0	-0.000014	1.540446	0.469439
3	1	0	-0.000118	2.329996	-0.082301
4	6	0	1.406160	-0.751859	0.299139
5	6	0	-1.406106	-0.751933	0.299139
6	1	0	1.319246	-1.788668	-0.037361
7	1	0	1.271793	-0.751817	1.384438
8	1	0	-1.319147	-1.788734	-0.037377
9	1	0	-1.271733	-0.751899	1.384437
10	6	0	2.775904	-0.187812	-0.076679
11	1	0	2.883699	0.847330	0.250797
12	1	0	3.574731	-0.765067	0.392249
13	1	0	2.935504	-0.214711	-1.156240
14	6	0	-2.775878	-0.187942	-0.076664
15	1	0	-3.574677	-0.765238	0.392260
16	1	0	-2.883717	0.847191	0.250828
17	1	0	-2.935481	-0.214831	-1.156224

2_(P_III)_method_B_DMSO.log

Input orientation:

Center Number	Atomic Number	Atomic Type	Coordinates (Angstroms) X	Y	Z
1	15	0	0.000005	0.148985	-0.486468
2	8	0	-0.000017	1.532169	0.460085
3	1	0	-0.000133	2.320801	-0.089237
4	6	0	1.405740	-0.757095	0.294795
5	6	0	-1.405684	-0.757180	0.294781
6	1	0	1.327234	-1.794003	-0.042342
7	1	0	1.263472	-0.758402	1.378760
8	1	0	-1.327141	-1.794069	-0.042409
9	1	0	-1.263411	-0.758534	1.378744
10	6	0	2.775303	-0.182441	-0.071142
11	1	0	2.868538	0.854020	0.254668
12	1	0	3.574698	-0.751649	0.404675
13	1	0	2.941903	-0.210123	-1.148921
14	6	0	-2.775272	-0.182562	-0.071120
15	1	0	-3.574643	-0.751830	0.404666
16	1	0	-2.868549	0.853876	0.254750
17	1	0	-2.941875	-0.210189	-1.148900

2_(P_III)_method_B_DMSO_smd.log

Input orientation:

Center Number	Atomic Number	Atomic Type	Coordinates (Angstroms) X	Y	Z
1	15	0	0.000004	0.155986	-0.475653
2	8	0	-0.000015	1.547318	0.457995
3	1	0	-0.000125	2.329627	-0.103053
4	6	0	1.404852	-0.751377	0.299111
5	6	0	-1.404797	-0.751455	0.299105
6	1	0	1.317304	-1.786796	-0.041989
7	1	0	1.267452	-0.755682	1.384048
8	1	0	-1.317208	-1.786861	-0.042029
9	1	0	-1.267390	-0.755788	1.384041
10	6	0	2.775610	-0.187614	-0.071931
11	1	0	2.886944	0.844730	0.263328
12	1	0	3.572504	-0.771436	0.392216
13	1	0	2.934729	-0.207010	-1.151761
14	6	0	-2.775582	-0.187740	-0.071911
15	1	0	-3.572449	-0.771617	0.392215
16	1	0	-2.886962	0.844586	0.263392
17	1	0	-2.934703	-0.207098	-1.151741

2_(P_III)_method_B_MeOH.log

Input orientation:

Center Number	Atomic Number	Atomic Type	Coordinates (Angstroms) X	Y	Z
1	15	0	0.000005	0.148836	-0.486774
2	8	0	-0.000017	1.531989	0.459775
3	1	0	-0.000134	2.320582	-0.089564
4	6	0	1.405709	-0.757181	0.294735
5	6	0	-1.405653	-0.757267	0.294719
6	1	0	1.327386	-1.794122	-0.042379
7	1	0	1.263311	-0.758412	1.378679
8	1	0	-1.327294	-1.794188	-0.042450
9	1	0	-1.263249	-0.758548	1.378662
10	6	0	2.775249	-0.182366	-0.071026
11	1	0	2.868303	0.854041	0.254990
12	1	0	3.574692	-0.751532	0.404767
13	1	0	2.941924	-0.209846	-1.148798
14	6	0	-2.775218	-0.182487	-0.071004
15	1	0	-3.574637	-0.751713	0.404757
16	1	0	-2.868314	0.853897	0.255075
17	1	0	-2.941896	-0.209909	-1.148777

2_(P_III)_method_B_MeOH_smd.log

Input orientation:

Center Number	Atomic Number	Atomic Type	Coordinates (Angstroms) X	Y	Z
1	15	0	0.000007	0.138432	-0.488521
2	8	0	-0.000015	1.534218	0.443813
3	1	0	-0.000150	2.319778	-0.113621
4	6	0	1.406489	-0.758909	0.294976
5	6	0	-1.406432	-0.758992	0.294963
6	1	0	1.331712	-1.794911	-0.047668
7	1	0	1.261407	-0.764896	1.378997
8	1	0	-1.331619	-1.794977	-0.047725
9	1	0	-1.261342	-0.765017	1.378983
10	6	0	2.773847	-0.179510	-0.065747
11	1	0	2.869669	0.854514	0.269858
12	1	0	3.573611	-0.753591	0.405699
13	1	0	2.942187	-0.197637	-1.144271
14	6	0	-2.773815	-0.179629	-0.065726
15	1	0	-3.573555	-0.753766	0.405693
16	1	0	-2.869676	0.854374	0.269933

17	1	0	-2.942157	-0.197707	-1.144251

2_(P_III)_method_B_THF.log

Input orientation:

Center Number	Atomic Number	Atomic Type	Coordinates (Angstroms) X	Y	Z
1	15	0	0.000006	0.147326	-0.489895
2	8	0	-0.000019	1.530226	0.456651
3	1	0	-0.000133	2.318391	-0.092897
4	6	0	1.405387	-0.758034	0.294132
5	6	0	-1.405330	-0.758124	0.294110
6	1	0	1.328858	-1.795295	-0.042745
7	1	0	1.261668	-0.758545	1.377874
8	1	0	-1.328767	-1.795361	-0.042835
9	1	0	-1.261606	-0.758699	1.377851
10	6	0	2.774699	-0.181618	-0.069850
11	1	0	2.865972	0.854242	0.258249
12	1	0	3.574626	-0.750399	0.405656
13	1	0	2.942102	-0.207031	-1.147552
14	6	0	-2.774668	-0.181736	-0.069825
15	1	0	-3.574570	-0.750585	0.405641
16	1	0	-2.865982	0.854096	0.258350
17	1	0	-2.942074	-0.207077	-1.147529

2_(P_III)_method_B_THF_smd.log

Input orientation:

Center Number	Atomic Number	Atomic Type	Coordinates (Angstroms) X	Y	Z
1	15	0	0.000004	0.152353	-0.480019
2	8	0	-0.000016	1.542387	0.455444
3	1	0	-0.000124	2.325291	-0.103832
4	6	0	1.405602	-0.753452	0.298478
5	6	0	-1.405547	-0.753534	0.298468
6	1	0	1.322145	-1.789755	-0.041326
7	1	0	1.267498	-0.756415	1.383309
8	1	0	-1.322050	-1.789820	-0.041380
9	1	0	-1.267437	-0.756535	1.383298
10	6	0	2.775406	-0.185628	-0.071213
11	1	0	2.882105	0.846762	0.265055
12	1	0	3.574012	-0.766629	0.393446
13	1	0	2.935329	-0.203612	-1.150872
14	6	0	-2.775377	-0.185752	-0.071191
15	1	0	-3.573957	-0.766813	0.393440
16	1	0	-2.882122	0.846615	0.265131
17	1	0	-2.935302	-0.203687	-1.150850

2_(P_III)_method_B_toluene.log

Input orientation:

Center Number	Atomic Number	Atomic Type	Coordinates (Angstroms) X	Y	Z
1	15	0	0.000005	0.143818	-0.496664
2	8	0	-0.000019	1.526533	0.449924
3	1	0	-0.000124	2.313692	-0.100154
4	6	0	1.404692	-0.759905	0.292800
5	6	0	-1.404637	-0.759994	0.292781
6	1	0	1.331898	-1.797758	-0.043660
7	1	0	1.258051	-0.759286	1.376121
8	1	0	-1.331807	-1.797824	-0.043745
9	1	0	-1.257991	-0.759435	1.376101
10	6	0	2.773453	-0.179904	-0.067214
11	1	0	2.860755	0.854881	0.265054
12	1	0	3.574430	-0.747750	0.407766
13	1	0	2.942533	-0.201009	-1.144730
14	6	0	-2.773423	-0.180024	-0.067190
15	1	0	-3.574376	-0.747936	0.407751
16	1	0	-2.860767	0.854734	0.265153
17	1	0	-2.942505	-0.201058	-1.144707

2_(P_III)_method_B_toluene_smd.log

Input orientation:

Center Number	Atomic Number	Atomic Type	Coordinates (Angstroms) X	Y	Z
1	15	0	0.000004	0.147322	-0.488282
2	8	0	-0.000019	1.534706	0.451403
3	1	0	-0.000120	2.318211	-0.105057
4	6	0	1.405718	-0.756241	0.297054
5	6	0	-1.405663	-0.756327	0.297040
6	1	0	1.328674	-1.793980	-0.040248
7	1	0	1.265792	-0.757203	1.381600
8	1	0	-1.328581	-1.794046	-0.040316
9	1	0	-1.265732	-0.757339	1.381585
10	6	0	2.773943	-0.182795	-0.069719
11	1	0	2.874040	0.849484	0.268185
12	1	0	3.574996	-0.759595	0.395729
13	1	0	2.935932	-0.198497	-1.148953
14	6	0	-2.773914	-0.182918	-0.069696
15	1	0	-3.574941	-0.759781	0.395718
16	1	0	-2.874056	0.849336	0.268273
17	1	0	-2.935905	-0.198561	-1.148931

2_(P_III)_method_B_water.log

Input orientation:

Center Number	Atomic Number	Atomic Type	Coordinates (Angstroms) X	Y	Z
1	15	0	0.000005	0.149125	-0.486180
2	8	0	-0.000017	1.532340	0.460376
3	1	0	-0.000133	2.321007	-0.088930
4	6	0	1.405769	-0.757013	0.294852
5	6	0	-1.405713	-0.757098	0.294839
6	1	0	1.327090	-1.793890	-0.042307
7	1	0	1.263624	-0.758393	1.378836
8	1	0	-1.326997	-1.793956	-0.042370
9	1	0	-1.263563	-0.758521	1.378822
10	6	0	2.775354	-0.182512	-0.071250
11	1	0	2.868759	0.853999	0.254366
12	1	0	3.574704	-0.751760	0.404587
13	1	0	2.941883	-0.210383	-1.149036
14	6	0	-2.775323	-0.182633	-0.071229
15	1	0	-3.574649	-0.751941	0.404580

16	1	0	-2.868771	0.853856	0.254444
17	1	0	-2.941855	-0.210452	-1.149016

2_(P_III)_method_B_water_smd.log

Input orientation:

Center Number	Atomic Number	Atomic Type	Coordinates (Angstroms) X	Y	Z
1	15	0	0.000007	0.140276	-0.493507
2	8	0	-0.000014	1.536447	0.440798
3	1	0	-0.000155	2.320302	-0.118545
4	6	0	1.403936	-0.758532	0.291925
5	6	0	-1.403878	-0.758613	0.291915
6	1	0	1.328209	-1.793557	-0.052033
7	1	0	1.253376	-0.765252	1.374824
8	1	0	-1.328117	-1.793622	-0.052084
9	1	0	-1.253312	-0.765367	1.374812
10	6	0	2.773061	-0.180248	-0.062479
11	1	0	2.866960	0.853603	0.272849
12	1	0	3.569801	-0.754233	0.412976
13	1	0	2.946056	-0.199025	-1.139761
14	6	0	-2.773028	-0.180366	-0.062458
15	1	0	-3.569745	-0.754403	0.412973
16	1	0	-2.866965	0.853466	0.272919
17	1	0	-2.946027	-0.199100	-1.139740

2_(P_III)_method_D_DCM.log

Input orientation:

Center Number	Atomic Number	Atomic Type	Coordinates (Angstroms) X	Y	Z
1	15	0	0.000001	0.117393	-0.527967
2	8	0	-0.000001	1.528799	0.422343
3	1	0	-0.000088	2.312918	-0.141555
4	6	0	1.409927	-0.773291	0.288192
5	6	0	-1.409923	-0.773320	0.288168
6	1	0	1.350820	-1.819780	-0.037730
7	1	0	1.248958	-0.759761	1.373818
8	1	0	-1.350826	-1.819793	-0.037808
9	1	0	-1.248951	-0.759846	1.373794
10	6	0	2.776728	-0.177615	-0.065322
11	1	0	2.842728	0.867767	0.253177
12	1	0	3.582910	-0.728647	0.430351
13	1	0	2.959239	-0.213419	-1.144891
14	6	0	-2.776723	-0.177618	-0.065310
15	1	0	-3.582906	-0.728674	0.430335
16	1	0	-2.842717	0.867746	0.253247
17	1	0	-2.959237	-0.213362	-1.144881

2_(P_V)_method_A.log

Input orientation:

Center Number	Atomic Number	Atomic Type	Coordinates (Angstroms) X	Y	Z
1	15	0	-0.000014	0.244461	0.276519
2	1	0	0.000128	0.257462	1.701744
3	8	0	-0.000155	1.614205	-0.348665
4	6	0	1.464753	-0.793832	-0.113656
5	1	0	1.359214	-1.747788	0.419753
6	1	0	1.420152	-1.014049	-1.187441
7	6	0	-1.464741	-0.793997	-0.113364
8	1	0	-1.358979	-1.747952	0.420001
9	1	0	-1.420341	-1.014186	-1.187164
10	6	0	-2.790581	-0.103889	0.239090
11	1	0	-2.882040	0.851361	-0.284875
12	1	0	-3.639487	-0.734619	-0.043447
13	1	0	-2.864792	0.093208	1.314204
14	6	0	2.790587	-0.103561	0.238505
15	1	0	3.639507	-0.734195	-0.044202
16	1	0	2.881820	0.851691	-0.285495
17	1	0	2.865002	0.093563	1.313600

2_(P_V)_method_A_DCM.log

Input orientation:

Center Number	Atomic Number	Atomic Type	Coordinates (Angstroms) X	Y	Z
1	15	0	0.000000	0.213028	0.203676
2	1	0	-0.000007	0.172286	1.622721
3	8	0	0.000006	1.630693	-0.341641
4	6	0	1.465654	-0.788311	-0.251284
5	1	0	1.350829	-1.770901	0.223044
6	1	0	1.422025	-0.944237	-1.335902
7	6	0	-1.465655	-0.788302	-0.251299
8	1	0	-1.350829	-1.770903	0.223005
9	1	0	-1.422031	-0.944201	-1.335921
10	6	0	-2.796082	-0.135410	0.150511
11	1	0	-2.918939	0.839260	-0.330069
12	1	0	-3.634834	-0.770234	-0.151219
13	1	0	-2.859399	0.009625	1.234195
14	6	0	2.796082	-0.135408	0.150505
15	1	0	3.634832	-0.770239	-0.151213
16	1	0	2.918935	0.839250	-0.330100
17	1	0	2.859403	0.009655	1.234185

2_(P_V)_method_A_DCM_smd.log

Input orientation:

Center Number	Atomic Number	Atomic Type	Coordinates (Angstroms) X	Y	Z
1	15	0	-0.000000	0.223476	0.184195
2	1	0	-0.000001	0.192753	1.603671
3	8	0	0.000000	1.639072	-0.369543
4	6	0	1.464906	-0.781854	-0.260436
5	1	0	1.334642	-1.765406	0.209058
6	1	0	1.432674	-0.931903	-1.346873
7	6	0	-1.464908	-0.781852	-0.260437
8	1	0	-1.334644	-1.765405	0.209055
9	1	0	-1.432676	-0.931900	-1.346874
10	6	0	-2.793919	-0.143688	0.162792
11	1	0	-2.941214	0.827782	-0.319292
12	1	0	-3.631288	-0.790350	-0.120434
13	1	0	-2.838768	0.005594	1.247625
14	6	0	2.793918	-0.143689	0.162791

15	1	0	3.631287	-0.790352	-0.120435
16	1	0	2.941213	0.827780	-0.319296
17	1	0	2.838767	0.005595	1.247624

2_(P_V)_method_B.log

Input orientation:

Center Number	Atomic Number	Atomic Type	Coordinates (Angstroms) X	Y	Z
1	15	0	0.000000	0.203139	0.193149
2	1	0	-0.000003	0.138506	1.611586
3	8	0	0.000002	1.584669	-0.347105
4	6	0	1.456010	-0.798436	-0.246809
5	1	0	1.353069	-1.776597	0.230404
6	1	0	1.415689	-0.957790	-1.326905
7	6	0	-1.456012	-0.798433	-0.246813
8	1	0	-1.353070	-1.776597	0.230391
9	1	0	-1.415692	-0.957778	-1.326911
10	6	0	-2.772730	-0.123420	0.148615
11	1	0	-2.858506	0.857030	-0.316807
12	1	0	-3.623731	-0.727912	-0.164592
13	1	0	-2.840274	0.011787	1.228894
14	6	0	2.772729	-0.123421	0.148612
15	1	0	3.623729	-0.727915	-0.164591
16	1	0	2.858504	0.857026	-0.316817
17	1	0	2.840275	0.011794	1.228891

2_(P_V)_method_B_DCM.log

Input orientation:

Center Number	Atomic Number	Atomic Type	Coordinates (Angstroms) X	Y	Z
1	15	0	0.000000	0.206022	0.194538
2	1	0	-0.000005	0.163073	1.608980
3	8	0	0.000004	1.601338	-0.342510
4	6	0	1.454395	-0.785980	-0.252377
5	1	0	1.339488	-1.764268	0.220165
6	1	0	1.413736	-0.942015	-1.332659
7	6	0	-1.454397	-0.785974	-0.252387
8	1	0	-1.339488	-1.764269	0.220138
9	1	0	-1.413741	-0.941991	-1.332671
10	6	0	-2.778984	-0.132893	0.152752
11	1	0	-2.900809	0.837872	-0.325314
12	1	0	-3.617094	-0.762901	-0.143389
13	1	0	-2.836375	0.012777	1.231952
14	6	0	2.778983	-0.132892	0.152748
15	1	0	3.617093	-0.762906	-0.143385
16	1	0	2.900807	0.837865	-0.325335
17	1	0	2.836377	0.012796	1.231945

2_(P_V)_method_B_DCM_smd.log

Input orientation:

Center Number	Atomic Number	Atomic Type	Coordinates (Angstroms) X	Y	Z
1	15	0	0.000000	0.221143	0.162187
2	1	0	-0.000001	0.198089	1.577227
3	8	0	0.000000	1.610232	-0.393268
4	6	0	1.454385	-0.777491	-0.266032
5	1	0	1.318586	-1.757564	0.198244
6	1	0	1.434095	-0.925862	-1.348509
7	6	0	-1.454387	-0.777490	-0.266033
8	1	0	-1.318588	-1.757563	0.198241
9	1	0	-1.434098	-0.925858	-1.348510
10	6	0	-2.775770	-0.144248	0.172880
11	1	0	-2.930398	0.823846	-0.303043
12	1	0	-3.612541	-0.788561	-0.098730
13	1	0	-2.806671	0.002973	1.253720
14	6	0	2.775769	-0.144249	0.172878
15	1	0	3.612539	-0.788564	-0.098730
16	1	0	2.930397	0.823844	-0.303047
17	1	0	2.806670	0.002974	1.253718

2_(P_V)_method_B_DMSO.log

Input orientation:

Center Number	Atomic Number	Atomic Type	Coordinates (Angstroms) X	Y	Z
1	15	0	0.000000	0.206284	0.190846
2	1	0	-0.000002	0.170975	1.604601
3	8	0	0.000001	1.603001	-0.349380
4	6	0	1.454887	-0.783914	-0.254844
5	1	0	1.336690	-1.762380	0.216195
6	1	0	1.416903	-0.938921	-1.335323
7	6	0	-1.454888	-0.783911	-0.254847
8	1	0	-1.336692	-1.762380	0.216185
9	1	0	-1.416905	-0.938912	-1.335327
10	6	0	-2.779607	-0.134685	0.155954
11	1	0	-2.908988	0.834977	-0.322716
12	1	0	-3.616250	-0.768486	-0.136000
13	1	0	-2.832594	0.011099	1.235312
14	6	0	2.779606	-0.134686	0.155952
15	1	0	3.616248	-0.768489	-0.135999
16	1	0	2.908987	0.834973	-0.322724
17	1	0	2.832595	0.011105	1.235308

2_(P_V)_method_B_DMSO_smd.log

Input orientation:

Center Number	Atomic Number	Atomic Type	Coordinates (Angstroms) X	Y	Z
1	15	0	-0.000000	0.223059	0.171381
2	1	0	-0.000001	0.188750	1.585920
3	8	0	0.000000	1.617557	-0.372384
4	6	0	1.451385	-0.775378	-0.264175
5	1	0	1.315139	-1.753910	0.203085
6	1	0	1.424892	-0.926034	-1.346251
7	6	0	-1.451386	-0.775377	-0.264176
8	1	0	-1.315141	-1.753910	0.203082
9	1	0	-1.424895	-0.926030	-1.346252
10	6	0	-2.775974	-0.145031	0.167245
11	1	0	-2.933690	0.819814	-0.314679
12	1	0	-3.609566	-0.794005	-0.103420
13	1	0	-2.810018	0.007687	1.247338

14	6	0	2.775973	-0.145032	0.167244
15	1	0	3.609564	-0.794007	-0.103420
16	1	0	2.933690	0.819812	-0.314683
17	1	0	2.810017	0.007688	1.247336

2_(P_V)_method_B_MeOH.log

Input orientation:

Center Number	Atomic Number	Atomic Type	Coordinates (Angstroms) X	Y	Z
1	15	0	0.000000	0.206183	0.191458
2	1	0	-0.000002	0.169878	1.605286
3	8	0	0.000002	1.602798	-0.348262
4	6	0	1.454821	-0.784174	-0.254494
5	1	0	1.337104	-1.762610	0.216759
6	1	0	1.416412	-0.939333	-1.334941
7	6	0	-1.454823	-0.784171	-0.254498
8	1	0	-1.337105	-1.762611	0.216747
9	1	0	-1.416415	-0.939321	-1.334947
10	6	0	-2.779551	-0.134436	0.155473
11	1	0	-2.907956	0.835344	-0.323178
12	1	0	-3.616353	-0.767777	-0.137047
13	1	0	-2.833172	0.011376	1.234801
14	6	0	2.779550	-0.134437	0.155471
15	1	0	3.616351	-0.767780	-0.137046
16	1	0	2.907954	0.835339	-0.323188
17	1	0	2.833172	0.011384	1.234797

2_(P_V)_method_B_MeOH_smd.log

Input orientation:

Center Number	Atomic Number	Atomic Type	Coordinates (Angstroms) X	Y	Z
1	15	0	-0.000000	0.189608	0.191543
2	1	0	-0.000001	0.173168	1.602617
3	8	0	0.000000	1.595916	-0.350403
4	6	0	1.457398	-0.784568	-0.258667
5	1	0	1.338443	-1.764304	0.210862
6	1	0	1.420210	-0.935641	-1.340277
7	6	0	-1.457400	-0.784566	-0.258668
8	1	0	-1.338445	-1.764303	0.210860
9	1	0	-1.420212	-0.935638	-1.340278
10	6	0	-2.776511	-0.132365	0.158642
11	1	0	-2.912415	0.835289	-0.324432
12	1	0	-3.615379	-0.768719	-0.124779
13	1	0	-2.821786	0.018788	1.238374
14	6	0	2.776510	-0.132366	0.158641
15	1	0	3.615377	-0.768721	-0.124780
16	1	0	2.912414	0.835287	-0.324435
17	1	0	2.821785	0.018788	1.238372

2_(P_V)_method_B_THF.log

Input orientation:

Center Number	Atomic Number	Atomic Type	Coordinates (Angstroms) X	Y	Z
1	15	0	0.000000	0.206106	0.194834
2	1	0	-0.000005	0.161810	1.609441
3	8	0	0.000004	1.600969	-0.341886
4	6	0	1.454332	-0.786387	-0.252028
5	1	0	1.339897	-1.764660	0.220736
6	1	0	1.413435	-0.942561	-1.332291
7	6	0	-1.454334	-0.786381	-0.252038
8	1	0	-1.339898	-1.764662	0.220708
9	1	0	-1.413440	-0.942536	-1.332304
10	6	0	-2.778805	-0.132594	0.152363
11	1	0	-2.899250	0.838439	-0.325444
12	1	0	-3.617285	-0.761817	-0.144453
13	1	0	-2.836800	0.012944	1.231560
14	6	0	2.778804	-0.132593	0.152358
15	1	0	3.617283	-0.761821	-0.144449
16	1	0	2.899248	0.838432	-0.325466
17	1	0	2.836802	0.012964	1.231553

2_(P_V)_method_B_THF_smd.log

Input orientation:

Center Number	Atomic Number	Atomic Type	Coordinates (Angstroms) X	Y	Z
1	15	0	-0.000000	0.221090	0.170134
2	1	0	-0.000001	0.184755	1.585738
3	8	0	0.000001	1.611705	-0.376187
4	6	0	1.453192	-0.778812	-0.262680
5	1	0	1.321173	-1.758121	0.204585
6	1	0	1.428008	-0.929516	-1.344811
7	6	0	-1.453194	-0.778811	-0.262681
8	1	0	-1.321175	-1.758121	0.204582
9	1	0	-1.428011	-0.929511	-1.344812
10	6	0	-2.775993	-0.142362	0.167085
11	1	0	-2.924191	0.825409	-0.311376
12	1	0	-3.612982	-0.784767	-0.108541
13	1	0	-2.812977	0.007218	1.247497
14	6	0	2.775992	-0.142363	0.167083
15	1	0	3.612980	-0.784769	-0.108541
16	1	0	2.924190	0.825407	-0.311380
17	1	0	2.812977	0.007220	1.247495

2_(P_V)_method_B_toluene.log

Input orientation:

Center Number	Atomic Number	Atomic Type	Coordinates (Angstroms) X	Y	Z
1	15	0	0.000000	0.207536	0.192236
2	1	0	-0.000003	0.154075	1.608625
3	8	0	0.000002	1.596326	-0.345753
4	6	0	1.454533	-0.790328	-0.250606
5	1	0	1.342769	-1.768746	0.223287
6	1	0	1.415164	-0.947059	-1.330952
7	6	0	-1.454535	-0.790324	-0.250611
8	1	0	-1.342770	-1.768746	0.223272
9	1	0	-1.415168	-0.947045	-1.330959
10	6	0	-2.776519	-0.130173	0.151896
11	1	0	-2.885427	0.844108	-0.321614
12	1	0	-3.619583	-0.751303	-0.149447

13	1	0	-2.836749	0.012349	1.231501
14	6	0	2.776518	-0.130173	0.151893
15	1	0	3.619582	-0.751307	-0.149446
16	1	0	2.885425	0.844103	-0.321627
17	1	0	2.836750	0.012359	1.231497

2_(P_V)_method_B_toluene_smd.log

Input orientation:

Center Number	Atomic Number	Atomic Type	Coordinates (Angstroms) X	Y	Z
1	15	0	-0.000000	0.216500	0.174352
2	1	0	-0.000001	0.170690	1.591410
3	8	0	0.000001	1.601933	-0.371914
4	6	0	1.454917	-0.785291	-0.258059
5	1	0	1.332744	-1.765266	0.211081
6	1	0	1.427764	-0.938021	-1.339869
7	6	0	-1.454918	-0.785289	-0.258060
8	1	0	-1.332746	-1.765266	0.211076
9	1	0	-1.427766	-0.938016	-1.339871
10	6	0	-2.774883	-0.136428	0.162792
11	1	0	-2.903678	0.835961	-0.310987
12	1	0	-3.617631	-0.766272	-0.123681
13	1	0	-2.821008	0.008581	1.243403
14	6	0	2.774882	-0.136430	0.162790
15	1	0	3.617629	-0.766275	-0.123681
16	1	0	2.903677	0.835958	-0.310992
17	1	0	2.821008	0.008583	1.243401

2_(P_V)_method_B_water.log

Input orientation:

Center Number	Atomic Number	Atomic Type	Coordinates (Angstroms) X	Y	Z
1	15	0	0.000000	0.206369	0.190308
2	1	0	-0.000001	0.171956	1.603994
3	8	0	0.000001	1.603196	-0.350350
4	6	0	1.454945	-0.783669	-0.255170
5	1	0	1.336305	-1.762159	0.215673
6	1	0	1.417340	-0.938538	-1.335677
7	6	0	-1.454947	-0.783666	-0.255172
8	1	0	-1.336307	-1.762159	0.215666
9	1	0	-1.417342	-0.938531	-1.335680
10	6	0	-2.779660	-0.134913	0.156387
11	1	0	-2.909965	0.834624	-0.322329
12	1	0	-3.616150	-0.769158	-0.135019
13	1	0	-2.832055	0.010875	1.235767
14	6	0	2.779659	-0.134914	0.156385
15	1	0	3.616149	-0.769161	-0.135018
16	1	0	2.909964	0.834620	-0.322336
17	1	0	2.832055	0.010880	1.235764

2_(P_V)_method_B_water_smd.log

Input orientation:

Center Number	Atomic Number	Atomic Type	Coordinates (Angstroms) X	Y	Z
1	15	0	-0.000000	0.191908	0.187140
2	1	0	-0.000001	0.179887	1.597180
3	8	0	0.000000	1.598281	-0.358611
4	6	0	1.456068	-0.783451	-0.260901
5	1	0	1.332311	-1.762469	0.207844
6	1	0	1.420608	-0.933678	-1.342221
7	6	0	-1.456069	-0.783450	-0.260901
8	1	0	-1.332313	-1.762468	0.207842
9	1	0	-1.420610	-0.933675	-1.342222
10	6	0	-2.774751	-0.134408	0.161630
11	1	0	-2.914029	0.832989	-0.320215
12	1	0	-3.613056	-0.771965	-0.118656
13	1	0	-2.814637	0.015769	1.241265
14	6	0	2.774750	-0.134409	0.161629
15	1	0	3.613055	-0.771967	-0.118656
16	1	0	2.914029	0.832987	-0.320218
17	1	0	2.814636	0.015770	1.241264

2_(P_V)_method_D_DCM.log

Input orientation:

Center Number	Atomic Number	Atomic Type	Coordinates (Angstroms) X	Y	Z
1	15	0	-0.000001	0.200515	0.213343
2	1	0	-0.000006	0.150502	1.631399
3	8	0	0.000005	1.620552	-0.323932
4	6	0	1.460742	-0.796284	-0.248731
5	1	0	1.353442	-1.777079	0.230158
6	1	0	1.409514	-0.954602	-1.332310
7	6	0	-1.460744	-0.796276	-0.248744
8	1	0	-1.353443	-1.777081	0.230124
9	1	0	-1.409520	-0.954571	-1.332327
10	6	0	-2.784794	-0.129197	0.144376
11	1	0	-2.892376	0.844353	-0.341463
12	1	0	-3.629827	-0.755475	-0.156250
13	1	0	-2.847420	0.022551	1.227006
14	6	0	2.784793	-0.129197	0.144370
15	1	0	3.629825	-0.755481	-0.156245
16	1	0	2.892373	0.844343	-0.341489
17	1	0	2.847422	0.022574	1.226997

3_(P_III)_method_A.log

Input orientation:

Center Number	Atomic Number	Atomic Type	Coordinates (Angstroms) X	Y	Z
1	15	0	-0.547778	0.661465	-0.225522
2	8	0	0.201621	1.071634	1.227164
3	1	0	-0.278238	1.781153	1.676934
4	6	0	1.847616	1.169379	-1.384386
5	1	0	2.089271	0.139378	-1.656607
6	1	0	2.221337	1.856660	-2.145044
7	1	0	2.289288	1.410685	-0.413581
8	6	0	-0.445759	-1.874716	0.581184
9	1	0	-0.055187	-2.834164	0.236835
10	1	0	-0.104526	-1.685765	1.604127
11	1	0	-1.542925	-1.908064	0.561083

Center Number	Atomic Number	Atomic Type	Coordinates (Angstroms) X	Y	Z
12	8	0	0.062747	-0.878570	-0.313511
13	8	0	0.416142	1.351265	-1.347610

3_(P_III)_method_A_DCM.log

Input orientation:

Center Number	Atomic Number	Atomic Type	Coordinates (Angstroms) X	Y	Z
1	15	0	-0.050093	0.502901	-0.612111
2	8	0	-0.050406	1.440811	0.780699
3	1	0	0.041073	2.382298	0.569317
4	6	0	2.000744	-0.848217	0.557016
5	1	0	1.465197	-1.779900	0.751537
6	1	0	3.041641	-1.060302	0.309860
7	1	0	1.952498	-0.199504	1.435153
8	6	0	-2.316776	-0.643022	0.192114
9	1	0	-2.699960	-1.637539	0.425121
10	1	0	-2.541627	0.038401	1.018134
11	1	0	-2.794970	-0.278845	-0.724870
12	8	0	-0.894856	-0.778800	0.019560
13	8	0	1.438528	-0.180450	-0.597344

3_(P_III)_method_A_DCM_smd.log

Input orientation:

Center Number	Atomic Number	Atomic Type	Coordinates (Angstroms) X	Y	Z
1	15	0	-0.051141	0.497483	-0.607424
2	8	0	-0.050823	1.448664	0.774384
3	1	0	0.028875	2.390395	0.547016
4	6	0	2.004705	-0.852027	0.557669
5	1	0	1.484043	-1.794718	0.743399
6	1	0	3.050208	-1.048978	0.312895
7	1	0	1.948796	-0.213189	1.443375
8	6	0	-2.318895	-0.639969	0.195058
9	1	0	-2.708790	-1.636963	0.410107
10	1	0	-2.547873	0.028247	1.031506
11	1	0	-2.791332	-0.259111	-0.718841
12	8	0	-0.895308	-0.779955	0.029148
13	8	0	1.438530	-0.182047	-0.594106

3_(P_III)_method_B.log

Input orientation:

Center Number	Atomic Number	Atomic Type	Coordinates (Angstroms) X	Y	Z
1	15	0	-0.043098	0.486507	-0.618724
2	8	0	-0.063841	1.438730	0.742609
3	1	0	0.029341	2.368786	0.514433
4	6	0	1.978668	-0.851379	0.559092
5	1	0	1.481832	-1.808089	0.707008
6	1	0	3.031547	-1.010766	0.341821
7	1	0	1.875852	-0.242201	1.455898
8	6	0	-2.287914	-0.634787	0.208033
9	1	0	-2.680081	-1.630367	0.400594
10	1	0	-2.501344	0.010943	1.060403
11	1	0	-2.773095	-0.228076	-0.683156
12	8	0	-0.880852	-0.767222	0.016420
13	8	0	1.423977	-0.174248	-0.580244

3_(P_III)_method_B_DCM.log

Input orientation:

Center Number	Atomic Number	Atomic Type	Coordinates (Angstroms) X	Y	Z
1	15	0	-0.053128	0.485746	-0.598513
2	8	0	-0.046461	1.438055	0.754688
3	1	0	0.044118	2.370480	0.528114
4	6	0	1.994468	-0.849368	0.556965
5	1	0	1.500574	-1.802902	0.730388
6	1	0	3.041612	-1.014258	0.319517
7	1	0	1.913748	-0.225471	1.445107
8	6	0	-2.309296	-0.636535	0.200278
9	1	0	-2.699549	-1.631952	0.393837
10	1	0	-2.543559	0.015741	1.041228
11	1	0	-2.773248	-0.240336	-0.705382
12	8	0	-0.891628	-0.764173	0.041029
13	8	0	1.413343	-0.187195	-0.583068

3_(P_III)_method_B_DCM_smd.log

Input orientation:

Center Number	Atomic Number	Atomic Type	Coordinates (Angstroms) X	Y	Z
1	15	0	-0.052483	0.479723	-0.598286
2	8	0	-0.047285	1.448095	0.741446
3	1	0	0.039104	2.379723	0.498959
4	6	0	1.995278	-0.860468	0.554790
5	1	0	1.563364	-1.853326	0.667664
6	1	0	3.060632	-0.946321	0.354294
7	1	0	1.842949	-0.285883	1.467155
8	6	0	-2.310012	-0.626682	0.209538
9	1	0	-2.705274	-1.618701	0.415117
10	1	0	-2.542308	0.034682	1.044759
11	1	0	-2.775421	-0.239674	-0.700010
12	8	0	-0.891873	-0.763268	0.050099
13	8	0	1.414322	-0.190067	-0.581339

3_(P_III)_method_B_DMSO.log

Input orientation:

Center Number	Atomic Number	Atomic Type	Coordinates (Angstroms) X	Y	Z
1	15	0	-0.055176	0.486255	-0.593359
2	8	0	-0.041639	1.434994	0.760678
3	1	0	0.045113	2.368721	0.536899
4	6	0	1.998733	-0.847684	0.556270
5	1	0	1.500599	-1.796494	0.742878
6	1	0	3.042246	-1.021710	0.309946
7	1	0	1.931522	-0.215517	1.439551

8	6	0	-2.314421	-0.637139	0.196752
9	1	0	-2.704114	-1.632044	0.393450
10	1	0	-2.553657	0.019009	1.033156
11	1	0	-2.773832	-0.245921	-0.713106
12	8	0	-0.894739	-0.763881	0.044952
13	8	0	1.410358	-0.190756	-0.583880

3_(P_III)_method_B_DMSO_smd.log

Input orientation:

Center	Atomic	Atomic	Coordinates (Angstroms)		
Number	Number	Type	X	Y	Z
1	15	0	-0.056989	0.473244	-0.592351
2	8	0	-0.049872	1.449073	0.742959
3	1	0	0.045144	2.377390	0.492587
4	6	0	2.000007	-0.851394	0.558642
5	1	0	1.528851	-1.818810	0.722209
6	1	0	3.052816	-0.993630	0.326886
7	1	0	1.906300	-0.239006	1.454113
8	6	0	-2.314614	-0.633781	0.205513
9	1	0	-2.716640	-1.632612	0.358905
10	1	0	-2.555414	-0.011026	1.067321
11	1	0	-2.766828	-0.201546	-0.690194
12	8	0	-0.893819	-0.768129	0.058362
13	8	0	1.412051	-0.191939	-0.580765

3_(P_III)_method_B_MeOH.log

Input orientation:

Center	Atomic	Atomic	Coordinates (Angstroms)		
Number	Number	Type	X	Y	Z
1	15	0	-0.054919	0.486192	-0.593998
2	8	0	-0.042267	1.435362	0.759926
3	1	0	0.044911	2.368940	0.535814
4	6	0	1.998224	-0.847905	0.556364
5	1	0	1.500612	-1.797312	0.741319
6	1	0	3.042196	-1.020786	0.311136
7	1	0	1.929334	-0.216768	1.440265
8	6	0	-2.313791	-0.637051	0.197187
9	1	0	-2.703560	-1.632007	0.393531
10	1	0	-2.552363	0.018641	1.034150
11	1	0	-2.773781	-0.245225	-0.712147
12	8	0	-0.894348	-0.763937	0.044419
13	8	0	1.410744	-0.190311	-0.583779

3_(P_III)_method_B_MeOH_smd.log

Input orientation:

Center	Atomic	Atomic	Coordinates (Angstroms)		
Number	Number	Type	X	Y	Z
1	15	0	-0.053315	0.488414	-0.599467
2	8	0	-0.043564	1.456709	0.736245
3	1	0	0.032303	2.390565	0.498844
4	6	0	1.993987	-0.865446	0.555730
5	1	0	1.556829	-1.856424	0.662324

6	1	0	3.058510	-0.954728	0.354007
7	1	0	1.843090	-0.292546	1.468982
8	6	0	-2.310405	-0.628845	0.211301
9	1	0	-2.697719	-1.623453	0.418340
10	1	0	-2.548028	0.034445	1.043033
11	1	0	-2.769958	-0.247245	-0.702674
12	8	0	-0.886549	-0.757443	0.058704
13	8	0	1.415812	-0.186171	-0.581182

3_(P_III)_method_B_THF.log

Input orientation:

Center	Atomic	Atomic	Coordinates (Angstroms)		
Number	Number	Type	X	Y	Z
1	15	0	-0.052723	0.485664	-0.599526
2	8	0	-0.047356	1.438638	0.753574
3	1	0	0.044039	2.370792	0.526469
4	6	0	1.993589	-0.849666	0.557081
5	1	0	1.500489	-1.804072	0.727993
6	1	0	3.041411	-1.012853	0.321356
7	1	0	1.910291	-0.227353	1.446125
8	6	0	-2.308270	-0.636440	0.200955
9	1	0	-2.698607	-1.631964	0.393936
10	1	0	-2.541632	0.015111	1.042748
11	1	0	-2.773113	-0.239334	-0.703912
12	8	0	-0.891026	-0.764198	0.040301
13	8	0	1.413900	-0.186492	-0.582912

3_(P_III)_method_B_THF_smd.log

Input orientation:

Center	Atomic	Atomic	Coordinates (Angstroms)		
Number	Number	Type	X	Y	Z
1	15	0	-0.054919	0.476307	-0.597022
2	8	0	-0.051027	1.452242	0.740144
3	1	0	0.048628	2.379715	0.491365
4	6	0	1.995051	-0.853793	0.557756
5	1	0	1.530951	-1.827287	0.705114
6	1	0	3.051787	-0.983191	0.336164
7	1	0	1.884466	-0.252625	1.459025
8	6	0	-2.310192	-0.633510	0.208041
9	1	0	-2.708517	-1.632253	0.371955
10	1	0	-2.549716	-0.003821	1.065385
11	1	0	-2.767983	-0.211024	-0.689770
12	8	0	-0.891238	-0.764673	0.056284
13	8	0	1.413703	-0.188255	-0.580256

3_(P_III)_method_B_toluene.log

Input orientation:

Center	Atomic	Atomic	Coordinates (Angstroms)		
Number	Number	Type	X	Y	Z
1	15	0	-0.048809	0.485558	-0.608394
2	8	0	-0.054800	1.441582	0.746123
3	1	0	0.041846	2.371840	0.516262

Center Number	Atomic Number	Atomic Type	X	Y	Z
4	6	0	1.985927	-0.851452	0.557849
5	1	0	1.496698	-1.810992	0.711009
6	1	0	3.038286	-1.004619	0.334958
7	1	0	1.884968	-0.240192	1.453020
8	6	0	-2.299030	-0.635689	0.205772
9	1	0	-2.689868	-1.631693	0.396793
10	1	0	-2.524317	0.011894	1.053167
11	1	0	-2.772447	-0.233271	-0.692888
12	8	0	-0.886014	-0.764691	0.032211
13	8	0	1.418553	-0.180442	-0.581696

3_(P_III)_method_B_toluene_smd.log

Input orientation:

Center Number	Atomic Number	Atomic Type	Coordinates (Angstroms) X	Y	Z
1	15	0	-0.050696	0.479621	-0.606780
2	8	0	-0.055159	1.453166	0.735547
3	1	0	0.046753	2.379761	0.490142
4	6	0	1.986290	-0.856009	0.557002
5	1	0	1.528977	-1.835630	0.684393
6	1	0	3.047784	-0.972296	0.350752
7	1	0	1.854573	-0.268221	1.464462
8	6	0	-2.300385	-0.632350	0.211910
9	1	0	-2.696555	-1.630059	0.387986
10	1	0	-2.533165	0.004208	1.066393
11	1	0	-2.768941	-0.218398	-0.684862
12	8	0	-0.886148	-0.763210	0.047431
13	8	0	1.417664	-0.182749	-0.580187

3_(P_III)_method_B_water.log

Input orientation:

Center Number	Atomic Number	Atomic Type	Coordinates (Angstroms) X	Y	Z
1	15	0	-0.055428	0.486314	-0.592739
2	8	0	-0.041021	1.434645	0.761403
3	1	0	0.045333	2.368510	0.537939
4	6	0	1.999221	-0.847467	0.556178
5	1	0	1.500587	-1.795697	0.744390
6	1	0	3.042288	-1.022600	0.308795
7	1	0	1.933635	-0.214302	1.438853
8	6	0	-2.315032	-0.637229	0.196334
9	1	0	-2.704650	-1.632090	0.393348
10	1	0	-2.554925	0.019343	1.032206
11	1	0	-2.773874	-0.246583	-0.714028
12	8	0	-0.895118	-0.763822	0.045486
13	8	0	1.409978	-0.191191	-0.583978

3_(P_III)_method_B_water_smd.log

Input orientation:

Center Number	Atomic Number	Atomic Type	Coordinates (Angstroms) X	Y	Z
1	15	0	-0.052594	0.488983	-0.600163
2	8	0	-0.044237	1.451147	0.740553
3	1	0	0.024425	2.385586	0.504332
4	6	0	1.994154	-0.863303	0.556454
5	1	0	1.545082	-1.847122	0.673973
6	1	0	3.055686	-0.967270	0.349242
7	1	0	1.855251	-0.280354	1.464669
8	6	0	-2.309232	-0.629117	0.209489
9	1	0	-2.698133	-1.622482	0.416515
10	1	0	-2.541291	0.033808	1.042379
11	1	0	-2.769903	-0.244926	-0.702137
12	8	0	-0.885683	-0.761352	0.051670
13	8	0	1.417467	-0.185766	-0.582790

3_(P_III)_method_C_DCM.log

Input orientation:

Center Number	Atomic Number	Atomic Type	Coordinates (Angstroms) X	Y	Z
1	15	0	-0.055015	0.490226	-0.598326
2	8	0	-0.038509	1.443744	0.766515
3	1	0	0.041680	2.376925	0.536592
4	6	0	1.998596	-0.849888	0.555047
5	1	0	1.497817	-1.799142	0.733731
6	1	0	3.044099	-1.023679	0.315457
7	1	0	1.924776	-0.221521	1.440989
8	6	0	-2.314930	-0.638902	0.197989
9	1	0	-2.705035	-1.632797	0.400880
10	1	0	-2.554525	0.022373	1.030791
11	1	0	-2.774448	-0.253301	-0.714757
12	8	0	-0.894481	-0.768946	0.047857
13	8	0	1.420970	-0.187262	-0.588578

3_(P_III)_method_C_DCM_smd.log

Input orientation:

Center Number	Atomic Number	Atomic Type	Coordinates (Angstroms) X	Y	Z
1	15	0	-0.049103	0.481045	-0.622512
2	8	0	-0.029605	1.466007	0.717879
3	1	0	0.041578	2.395597	0.460642
4	6	0	2.005550	-0.849179	0.542475
5	1	0	1.528644	-1.813458	0.710342
6	1	0	3.058973	-0.997037	0.315518
7	1	0	1.909299	-0.229511	1.433180
8	6	0	-2.309313	-0.624025	0.198984
9	1	0	-2.706281	-1.616379	0.401464
10	1	0	-2.545619	0.036928	1.033836
11	1	0	-2.769051	-0.234835	-0.712875
12	8	0	-0.888629	-0.763531	0.047424
13	8	0	1.426803	-0.194886	-0.606124

3_(P_III)_method_D_DCM.log

Input orientation:

Center Number	Atomic Number	Atomic Type	Coordinates (Angstroms) X	Y	Z

1	15	0	-0.040190	0.487210	-0.651727
2	8	0	-0.052372	1.440206	0.729846
3	1	0	0.036779	2.379300	0.508437
4	6	0	1.987611	-0.839432	0.557632
5	1	0	1.438267	-1.760102	0.763950
6	1	0	3.029659	-1.066437	0.331132
7	1	0	1.930556	-0.170137	1.419382
8	6	0	-2.293884	-0.625725	0.205385
9	1	0	-2.688961	-1.611604	0.453124
10	1	0	-2.481384	0.064403	1.033073
11	1	0	-2.788861	-0.255595	-0.700140
12	8	0	-0.880851	-0.788225	-0.005203
13	8	0	1.449041	-0.191852	-0.618637

3_(P_III)_method_E_DCM.log

Input orientation:

Center	Atomic	Atomic	Coordinates (Angstroms)		
Number	Number	Type	X	Y	Z
1	15	0	-0.048207	0.471215	-0.607840
2	8	0	-0.048639	1.421318	0.729667
3	1	0	0.006776	2.350992	0.499333
4	6	0	1.968568	-0.844998	0.555127
5	1	0	1.487676	-1.809592	0.706722
6	1	0	3.025920	-0.990439	0.352033
7	1	0	1.850210	-0.233683	1.448960
8	6	0	-2.274553	-0.629056	0.212502
9	1	0	-2.686854	-1.621701	0.372782
10	1	0	-2.484754	-0.004847	1.081472
11	1	0	-2.745174	-0.187532	-0.669498
12	8	0	-0.873010	-0.775037	0.028390
13	8	0	1.413034	-0.188808	-0.585463

3_(P_III)_method_E_DCM_smd.log

Input orientation:

Center	Atomic	Atomic	Coordinates (Angstroms)		
Number	Number	Type	X	Y	Z
1	15	0	-0.046180	0.482197	-0.613936
2	8	0	-0.052665	1.435026	0.719462
3	1	0	-0.004398	2.366178	0.482102
4	6	0	1.975452	-0.835393	0.539794
5	1	0	1.515126	-1.813965	0.669619
6	1	0	3.038376	-0.957011	0.344521
7	1	0	1.839902	-0.245300	1.446192
8	6	0	-2.270242	-0.625004	0.199302
9	1	0	-2.683580	-1.621849	0.336034
10	1	0	-2.484900	-0.022375	1.083098
11	1	0	-2.740306	-0.164048	-0.673801
12	8	0	-0.866946	-0.767467	0.016392
13	8	0	1.418117	-0.169319	-0.594765

3_(P_V)_method_A.log

Input orientation:

Center	Atomic	Atomic	Coordinates (Angstroms)		
Number	Number	Type	X	Y	Z
1	15	0	0.067387	-0.397337	1.613908
2	1	0	-0.169462	-0.990663	2.861308
3	8	0	-0.097656	1.078664	1.556303
4	6	0	-2.152917	-0.705797	0.177884
5	1	0	-2.427778	-1.237321	-0.734044
6	1	0	-2.894165	-0.911054	0.957244
7	6	0	2.260156	-0.643037	0.144559
8	1	0	1.763050	-1.032198	-0.748413
9	1	0	2.356260	0.444002	0.081956
10	1	0	3.244670	-1.099312	0.248243
11	8	0	-0.862775	-1.220512	0.571096
12	8	0	1.522056	-1.017420	1.328586
13	1	0	-2.098341	0.368798	-0.009844

3_(P_V)_method_A_DCM.log

Input orientation:

Center	Atomic	Atomic	Coordinates (Angstroms)		
Number	Number	Type	X	Y	Z
1	15	0	0.008050	0.505126	-0.365825
2	1	0	0.225413	0.966004	-1.668843
3	8	0	0.159620	1.538908	0.701070
4	6	0	2.317970	-0.656867	0.286144
5	1	0	2.676247	-1.670777	0.462158
6	1	0	2.938603	-0.173280	-0.473754
7	6	0	-2.145651	-0.770228	0.546336
8	1	0	-1.593604	-1.637552	0.915474
9	1	0	-2.291942	-0.041863	1.346921
10	1	0	-3.109505	-1.084449	0.148013
11	8	0	0.956238	-0.791800	-0.190402
12	8	0	-1.437340	-0.162331	-0.564717
13	1	0	2.342730	-0.082499	1.214117

3_(P_V)_method_A_DCM_smd.log

Input orientation:

Center	Atomic	Atomic	Coordinates (Angstroms)		
Number	Number	Type	X	Y	Z
1	15	0	0.014562	0.479433	-0.358563
2	1	0	0.258532	0.918280	-1.664794
3	8	0	0.148454	1.531455	0.693500
4	6	0	2.333498	-0.651957	0.281451
5	1	0	2.748750	-1.657956	0.352169
6	1	0	2.898448	-0.072482	-0.455864
7	6	0	-2.168345	-0.753897	0.547492
8	1	0	-1.634374	-1.613362	0.960958
9	1	0	-2.329762	0.001716	1.320310
10	1	0	-3.126284	-1.075143	0.137843
11	8	0	0.957960	-0.817177	-0.146542
12	8	0	-1.429667	-0.189382	-0.567841
13	1	0	2.375059	-0.161135	1.256575

3_(P_V)_method_B.log

Input orientation:

Center	Atomic	Atomic	Coordinates (Angstroms)		
Number	Number	Type	X	Y	Z
1	15	0	0.002037	0.493068	-0.367328
2	1	0	0.175029	1.003405	-1.655520
3	8	0	0.155905	1.463046	0.721138
4	6	0	2.300727	-0.641360	0.285314
5	1	0	2.606689	-1.642521	0.575506
6	1	0	2.995982	-0.251148	-0.458214
7	6	0	-2.121349	-0.776450	0.541802
8	1	0	-1.629045	-1.694378	0.860031
9	1	0	-2.175887	-0.073944	1.371378
10	1	0	-3.119610	-1.001824	0.179552
11	8	0	0.986252	-0.761143	-0.285639
12	8	0	-1.411092	-0.191757	-0.564890
13	1	0	2.281195	0.013393	1.153562

3_(P_V)_method_B_DCM.log

Input orientation:

Center	Atomic	Atomic	Coordinates (Angstroms)		
Number	Number	Type	X	Y	Z
1	15	0	0.009675	0.491111	-0.365915
2	1	0	0.240055	0.931986	-1.668593
3	8	0	0.146641	1.533099	0.665595
4	6	0	2.313935	-0.634999	0.294995
5	1	0	2.660553	-1.635709	0.532052
6	1	0	2.934695	-0.209158	-0.492230
7	6	0	-2.153572	-0.739407	0.540764
8	1	0	-1.633643	-1.611752	0.931169
9	1	0	-2.289847	0.000546	1.326220
10	1	0	-3.116680	-1.033106	0.137104
11	8	0	0.953520	-0.773399	-0.165812
12	8	0	-1.411634	-0.169721	-0.560588
13	1	0	2.351872	-0.006365	1.181098

3_(P_V)_method_B_DCM_smd.log

Input orientation:

Center	Atomic	Atomic	Coordinates (Angstroms)		
Number	Number	Type	X	Y	Z
1	15	0	0.019722	0.451429	-0.340659
2	1	0	0.289959	0.873057	-1.642568
3	8	0	0.122347	1.510103	0.678324
4	6	0	2.344900	-0.630990	0.280285
5	1	0	2.765685	-1.627532	0.379172
6	1	0	2.885379	-0.083204	-0.492219
7	6	0	-2.194459	-0.711278	0.538072
8	1	0	-1.686776	-1.533168	1.040276
9	1	0	-2.401884	0.090078	1.244525
10	1	0	-3.123109	-1.065770	0.101025
11	8	0	0.962825	-0.807909	-0.098626
12	8	0	-1.394058	-0.218975	-0.560667
13	1	0	2.415038	-0.102715	1.228917

3_(P_V)_method_B_DMSO.log

Input orientation:

Center	Atomic	Atomic	Coordinates (Angstroms)		
Number	Number	Type	X	Y	Z
1	15	0	0.010082	0.492287	-0.363957
2	1	0	0.247285	0.928264	-1.666602
3	8	0	0.143652	1.540834	0.663148
4	6	0	2.315390	-0.635689	0.294595
5	1	0	2.662813	-1.636894	0.527645
6	1	0	2.927538	-0.210300	-0.499287
7	6	0	-2.155384	-0.740129	0.540028
8	1	0	-1.632168	-1.609981	0.931118
9	1	0	-2.298089	-0.001559	1.325550
10	1	0	-3.115579	-1.038066	0.132890
11	8	0	0.948914	-0.772460	-0.151157
12	8	0	-1.412381	-0.165085	-0.559030
13	1	0	2.363499	-0.008096	1.180919

3_(P_V)_method_B_DMSO_smd.log

Input orientation:

Center	Atomic	Atomic	Coordinates (Angstroms)		
Number	Number	Type	X	Y	Z
1	15	0	0.019287	0.454289	-0.346156
2	1	0	0.296385	0.859749	-1.651718
3	8	0	0.128305	1.524611	0.660279
4	6	0	2.336476	-0.628649	0.285160
5	1	0	2.765022	-1.623332	0.367854
6	1	0	2.869209	-0.066291	-0.482232
7	6	0	-2.187503	-0.715135	0.539192
8	1	0	-1.684149	-1.558723	1.008475
9	1	0	-2.367532	0.069317	1.271730
10	1	0	-3.130958	-1.042848	0.112902
11	8	0	0.952344	-0.809692	-0.087620
12	8	0	-1.400556	-0.206518	-0.562773
13	1	0	2.409241	-0.113652	1.240764

3_(P_V)_method_B_MeOH.log

Input orientation:

Center	Atomic	Atomic	Coordinates (Angstroms)		
Number	Number	Type	X	Y	Z
1	15	0	0.010048	0.492128	-0.364177
2	1	0	0.246517	0.928584	-1.666847
3	8	0	0.144015	1.540004	0.663359
4	6	0	2.315241	-0.635621	0.294641
5	1	0	2.662697	-1.636794	0.527857
6	1	0	2.928254	-0.209915	-0.498437
7	6	0	-2.155213	-0.740016	0.540127
8	1	0	-1.632308	-1.610050	0.931281
9	1	0	-2.297344	-0.001215	1.325544
10	1	0	-3.115668	-1.037624	0.133325
11	8	0	0.949411	-0.772607	-0.152725
12	8	0	-1.412293	-0.165613	-0.559191
13	1	0	2.362213	-0.008134	1.181102

3_(P_V)_method_B_MeOH_smd.log

Input orientation:

Center Number	Atomic Number	Atomic Type	Coordinates (Angstroms) X	Y	Z
1	15	0	0.015535	0.466467	-0.370502
2	1	0	0.282562	0.875042	-1.674421
3	8	0	0.141044	1.543827	0.635717
4	6	0	2.325502	-0.632168	0.297335
5	1	0	2.728810	-1.634815	0.402144
6	1	0	2.876619	-0.091051	-0.471118
7	6	0	-2.172754	-0.725337	0.546018
8	1	0	-1.651339	-1.579743	0.972814
9	1	0	-2.333925	0.038958	1.303350
10	1	0	-3.123355	-1.040593	0.127437
11	8	0	0.941519	-0.793839	-0.098800
12	8	0	-1.410101	-0.179277	-0.560546
13	1	0	2.385453	-0.104344	1.246431

3_(P_V)_method_B_THF.log

Input orientation:

Center Number	Atomic Number	Atomic Type	Coordinates (Angstroms) X	Y	Z
1	15	0	0.009562	0.491108	-0.366568
2	1	0	0.238036	0.933023	-1.669325
3	8	0	0.147394	1.531629	0.665893
4	6	0	2.313428	-0.634974	0.295165
5	1	0	2.659522	-1.635696	0.533128
6	1	0	2.936559	-0.209097	-0.490240
7	6	0	-2.152918	-0.739277	0.540959
8	1	0	-1.633490	-1.612053	0.931212
9	1	0	-2.287966	0.000874	1.326480
10	1	0	-3.116634	-1.032332	0.138188
11	8	0	0.954599	-0.773269	-0.169480
12	8	0	-1.411524	-0.170567	-0.560868
13	1	0	2.349003	-0.006241	1.181316

3_(P_V)_method_B_THF_smd.log

Input orientation:

Center Number	Atomic Number	Atomic Type	Coordinates (Angstroms) X	Y	Z
1	15	0	0.017204	0.459010	-0.353459
2	1	0	0.277506	0.874984	-1.659757
3	8	0	0.136001	1.518668	0.661097
4	6	0	2.331385	-0.629625	0.287826
5	1	0	2.749940	-1.626382	0.394943
6	1	0	2.888261	-0.082092	-0.473392
7	6	0	-2.180146	-0.717608	0.541139
8	1	0	-1.676173	-1.567293	0.999048
9	1	0	-2.348629	0.062153	1.281527
10	1	0	-3.129346	-1.037522	0.121530
11	8	0	0.957451	-0.805119	-0.118233
12	8	0	-1.401957	-0.205172	-0.563631
13	1	0	2.384074	-0.100876	1.237220

3_(P_V)_method_B_toluene.log

Input orientation:

Center Number	Atomic Number	Atomic Type	Coordinates (Angstroms) X	Y	Z
1	15	0	0.007384	0.491945	-0.372765
2	1	0	0.216223	0.950633	-1.673983
3	8	0	0.152398	1.512557	0.674424
4	6	0	2.307709	-0.632691	0.296953
5	1	0	2.638277	-1.630406	0.569114
6	1	0	2.961393	-0.230224	-0.476469
7	6	0	-2.144445	-0.741169	0.541678
8	1	0	-1.636986	-1.628645	0.915427
9	1	0	-2.250534	-0.006248	1.336852
10	1	0	-3.121021	-1.011161	0.153153
11	8	0	0.966587	-0.770038	-0.211191
12	8	0	-1.410807	-0.178977	-0.565611
13	1	0	2.319393	0.017551	1.168278

3_(P_V)_method_B_toluene_smd.log

Input orientation:

Center Number	Atomic Number	Atomic Type	Coordinates (Angstroms) X	Y	Z
1	15	0	0.012677	0.471155	-0.370063
2	1	0	0.243779	0.901588	-1.677846
3	8	0	0.149963	1.512914	0.656759
4	6	0	2.315926	-0.630882	0.295643
5	1	0	2.709673	-1.631214	0.453727
6	1	0	2.920832	-0.118391	-0.453577
7	6	0	-2.160069	-0.724385	0.544000
8	1	0	-1.657173	-1.594090	0.964806
9	1	0	-2.293752	0.037767	1.309719
10	1	0	-3.126379	-1.018584	0.144775
11	8	0	0.963437	-0.793767	-0.173229
12	8	0	-1.406788	-0.197416	-0.567506
13	1	0	2.333446	-0.071568	1.228650

3_(P_V)_method_B_water.log

Input orientation:

Center Number	Atomic Number	Atomic Type	Coordinates (Angstroms) X	Y	Z
1	15	0	0.010112	0.492441	-0.363747
2	1	0	0.248002	0.927981	-1.666365
3	8	0	0.143301	1.541608	0.662963
4	6	0	2.315528	-0.635750	0.294551
5	1	0	2.662897	-1.636980	0.527505
6	1	0	2.926879	-0.210717	-0.500103
7	6	0	-2.155540	-0.740241	0.539930
8	1	0	-1.632047	-1.609945	0.930929
9	1	0	-2.298751	-0.001905	1.325571

10	1	0	-3.115506	-1.038455	0.132487
11	8	0	0.948450	-0.772313	-0.149691
12	8	0	-1.412465	-0.164588	-0.558882
13	1	0	2.364710	-0.008008	1.180710

3_(P_V)_method_B_water_smd.log

Input orientation:

Center	Atomic	Atomic	Coordinates (Angstroms)		
Number	Number	Type	X	Y	Z
1	15	0	0.015370	0.467293	-0.369364
2	1	0	0.286404	0.876775	-1.671145
3	8	0	0.139946	1.543756	0.639205
4	6	0	2.324060	-0.631974	0.297101
5	1	0	2.724766	-1.633850	0.412771
6	1	0	2.873679	-0.101102	-0.478713
7	6	0	-2.171413	-0.726670	0.544778
8	1	0	-1.648234	-1.582342	0.965487
9	1	0	-2.329277	0.034405	1.305335
10	1	0	-3.122846	-1.039558	0.127792
11	8	0	0.938423	-0.794669	-0.094793
12	8	0	-1.411524	-0.175636	-0.561991
13	1	0	2.386217	-0.093301	1.239397

3_(P_V)_method_C_DCM.log

Input orientation:

Center	Atomic	Atomic	Coordinates (Angstroms)		
Number	Number	Type	X	Y	Z
1	15	0	0.009356	0.489326	-0.370025
2	1	0	0.243999	0.933338	-1.675446
3	8	0	0.140998	1.533217	0.675882
4	6	0	2.319142	-0.634312	0.295998
5	1	0	2.674066	-1.633286	0.529310
6	1	0	2.937367	-0.199542	-0.488836
7	6	0	-2.157769	-0.738379	0.541147
8	1	0	-1.643177	-1.615523	0.928622
9	1	0	-2.279373	0.004813	1.326341
10	1	0	-3.127704	-1.022880	0.146590
11	8	0	0.958913	-0.785654	-0.167386
12	8	0	-1.420732	-0.178917	-0.571463
13	1	0	2.350484	-0.009076	1.185124

3_(P_V)_method_C_DCM_smd.log

Input orientation:

Center	Atomic	Atomic	Coordinates (Angstroms)		
Number	Number	Type	X	Y	Z
1	15	0	0.017545	0.463026	-0.380364
2	1	0	0.274481	0.877756	-1.691585
3	8	0	0.132250	1.531065	0.643587
4	6	0	2.340988	-0.621811	0.268384
5	1	0	2.765807	-1.616992	0.366516
6	1	0	2.890851	-0.063216	-0.490013
7	6	0	-2.167973	-0.734345	0.533151

8	1	0	-1.659340	-1.594163	0.966588
9	1	0	-2.313974	0.036403	1.287963
10	1	0	-3.128298	-1.040488	0.128452
11	8	0	0.965364	-0.807549	-0.136607
12	8	0	-1.409829	-0.211912	-0.584484
13	1	0	2.390662	-0.100896	1.222608

3_(P_V)_method_D_DCM.log

Input orientation:

Center	Atomic	Atomic	Coordinates (Angstroms)		
Number	Number	Type	X	Y	Z
1	15	0	0.007182	0.508744	-0.401580
2	1	0	0.220958	0.967432	-1.705416
3	8	0	0.170294	1.540421	0.665064
4	6	0	2.299168	-0.652080	0.275867
5	1	0	2.663777	-1.664881	0.442875
6	1	0	2.927916	-0.149838	-0.464741
7	6	0	-2.113227	-0.775025	0.535537
8	1	0	-1.539747	-1.636651	0.884293
9	1	0	-2.244824	-0.052127	1.343368
10	1	0	-3.083410	-1.098278	0.161579
11	8	0	0.948446	-0.792113	-0.227534
12	8	0	-1.441391	-0.152798	-0.589594
13	1	0	2.299180	-0.091059	1.211979

3_(P_V)_method_E_DCM.log

Input orientation:

Center	Atomic	Atomic	Coordinates (Angstroms)		
Number	Number	Type	X	Y	Z
1	15	0	0.011390	0.464133	-0.372376
2	1	0	0.261083	0.896820	-1.673240
3	8	0	0.138716	1.505853	0.652031
4	6	0	2.293882	-0.633395	0.295740
5	1	0	2.694679	-1.631547	0.440990
6	1	0	2.886002	-0.110282	-0.455188
7	6	0	-2.139555	-0.719446	0.541319
8	1	0	-1.618856	-1.575810	0.966721
9	1	0	-2.279916	0.048026	1.300258
10	1	0	-3.101764	-1.032504	0.149758
11	8	0	0.944765	-0.794321	-0.154426
12	8	0	-1.402562	-0.191437	-0.569555
13	1	0	2.317706	-0.082965	1.233825

3_(P_V)_method_E_DCM_smd.log

Input orientation:

Center	Atomic	Atomic	Coordinates (Angstroms)		
Number	Number	Type	X	Y	Z
1	15	0	0.016845	0.464433	-0.367376
2	1	0	0.287680	0.887611	-1.667484
3	8	0	0.125296	1.515064	0.650425
4	6	0	2.314896	-0.605367	0.263601
5	1	0	2.754160	-1.595813	0.343150

6	1	0	2.854341	-0.029691	-0.490186
7	6	0	-2.144039	-0.726337	0.522167
8	1	0	-1.628971	-1.581886	0.957759
9	1	0	-2.301212	0.040415	1.279564
10	1	0	-3.100440	-1.043732	0.117093
11	8	0	0.949390	-0.791532	-0.126210
12	8	0	-1.392805	-0.197428	-0.579566
13	1	0	2.372131	-0.098637	1.225574

4_(P_III)_method_A.log

Input orientation:

Center	Atomic	Atomic	Coordinates (Angstroms)		
Number	Number	Type	X	Y	Z
1	15	0	-0.592096	0.650234	-0.223076
2	8	0	0.132109	1.113389	1.228341
3	1	0	-0.360347	1.834668	1.644449
4	6	0	1.823790	1.154513	-1.375810
5	1	0	2.049965	0.107831	-1.599358
6	1	0	2.221674	1.393392	-0.384168
7	6	0	-0.512525	-1.872829	0.649405
8	1	0	-0.134923	-1.670334	1.658411
9	1	0	-1.609192	-1.794489	0.673040
10	8	0	0.026145	-0.888633	-0.253986
11	8	0	0.380725	1.315935	-1.352178
12	6	0	-0.085085	-3.249690	0.171197
13	1	0	1.006233	-3.325506	0.142468
14	1	0	-0.467276	-4.018384	0.851927
15	1	0	-0.471859	-3.449850	-0.832569
16	6	0	2.382199	2.084663	-2.437540
17	1	0	3.471566	1.977490	-2.489283
18	1	0	1.964584	1.847239	-3.420751
19	1	0	2.147855	3.128317	-2.206196

4_(P_III)_method_A_DCM.log

Input orientation:

Center	Atomic	Atomic	Coordinates (Angstroms)		
Number	Number	Type	X	Y	Z
1	15	0	-0.034341	0.928435	-0.574785
2	8	0	0.053275	1.723849	0.904125
3	1	0	0.245690	2.665811	0.781479
4	6	0	1.891962	-0.730342	0.434409
5	1	0	1.257860	-1.618440	0.506740
6	1	0	1.827716	-0.175653	1.375342
7	6	0	-2.421708	-0.064607	0.097647
8	1	0	-2.572677	0.472454	1.040616
9	1	0	-2.781131	0.573285	-0.721022
10	8	0	-1.006500	-0.317544	-0.069691
11	8	0	1.382156	0.109760	-0.640454
12	6	0	-3.154024	-1.394263	0.103321
13	1	0	-2.791798	-2.033557	0.914494
14	1	0	-4.225977	-1.225418	0.251151
15	1	0	-3.014015	-1.921179	-0.845541
16	6	0	3.327170	-1.098978	0.107471
17	1	0	3.729269	-1.743978	0.896263
18	1	0	3.385431	-1.640509	-0.841865
19	1	0	3.956142	-0.205917	0.038781

4_(P_III)_method_A_DCM_smd.log

Input orientation:

Center	Atomic	Atomic	Coordinates (Angstroms)		
Number	Number	Type	X	Y	Z
1	15	0	-0.035876	0.926686	-0.567981
2	8	0	0.050797	1.732368	0.903693
3	1	0	0.239324	2.676431	0.769498
4	6	0	1.896514	-0.729613	0.437452
5	1	0	1.262419	-1.617894	0.511798
6	1	0	1.839636	-0.176659	1.380280
7	6	0	-2.423654	-0.066212	0.100552
8	1	0	-2.579167	0.467253	1.045161
9	1	0	-2.780253	0.573921	-0.717836
10	8	0	-1.005823	-0.317990	-0.061731
11	8	0	1.382996	0.113505	-0.634880
12	6	0	-3.154857	-1.394689	0.099917
13	1	0	-2.797994	-2.037242	0.911972
14	1	0	-4.227728	-1.223701	0.244040
15	1	0	-3.013060	-1.920311	-0.850278
16	6	0	3.328015	-1.101007	0.104419
17	1	0	3.730230	-1.746816	0.893493
18	1	0	3.382329	-1.645018	-0.844626
19	1	0	3.960651	-0.209806	0.033538

4_(P_III)_method_B.log

Input orientation:

Center	Atomic	Atomic	Coordinates (Angstroms)		
Number	Number	Type	X	Y	Z
1	15	0	-0.027011	0.916219	-0.582786
2	8	0	0.042771	1.714141	0.874851
3	1	0	0.230118	2.648777	0.744350
4	6	0	1.863197	-0.742432	0.425744
5	1	0	1.238811	-1.634469	0.466118
6	1	0	1.777133	-0.218752	1.378236
7	6	0	-2.392120	-0.060944	0.104193
8	1	0	-2.531591	0.469524	1.048346
9	1	0	-2.764639	0.580844	-0.701010
10	8	0	-0.992070	-0.306953	-0.085497
11	8	0	1.368022	0.114944	-0.627213
12	6	0	-3.125248	-1.385896	0.112574
13	1	0	-2.752507	-2.025971	0.911825
14	1	0	-4.191630	-1.221454	0.272724
15	1	0	-2.994245	-1.906879	-0.835335
16	6	0	3.303454	-1.090988	0.118245
17	1	0	3.698946	-1.749434	0.893096
18	1	0	3.381574	-1.602748	-0.840549
19	1	0	3.921534	-0.194322	0.080572

4_(P_III)_method_B_DCM.log

Input orientation:

Center	Atomic	Atomic	Coordinates (Angstroms)		
Number	Number	Type	X	Y	Z

1	15	0	-0.037420	0.899827	-0.565302
2	8	0	0.064274	1.712630	0.875147
3	1	0	0.263214	2.645387	0.736151
4	6	0	1.880930	-0.747601	0.431026
5	1	0	1.271711	-1.648421	0.492653
6	1	0	1.800747	-0.209995	1.375453
7	6	0	-2.412989	-0.062426	0.112998
8	1	0	-2.570312	0.464818	1.055331
9	1	0	-2.762385	0.582909	-0.698123
10	8	0	-1.003050	-0.311895	-0.045669
11	8	0	1.354785	0.087966	-0.630452
12	6	0	-3.150411	-1.383871	0.100826
13	1	0	-2.799861	-2.029551	0.905791
14	1	0	-4.218118	-1.211548	0.240236
15	1	0	-3.005939	-1.900858	-0.847490
16	6	0	3.321686	-1.079853	0.111153
17	1	0	3.732506	-1.720143	0.892702
18	1	0	3.397386	-1.608325	-0.838940
19	1	0	3.927743	-0.175841	0.054994

4_(P_III)_method_B_DCM_smd.log

Input orientation:

Center Number	Atomic Number	Atomic Type	Coordinates (Angstroms) X	Y	Z
1	15	0	-0.038307	0.905677	-0.550589
2	8	0	0.052360	1.723848	0.885695
3	1	0	0.233268	2.662012	0.739871
4	6	0	1.892819	-0.729426	0.447254
5	1	0	1.268255	-1.617698	0.535336
6	1	0	1.847708	-0.178816	1.386922
7	6	0	-2.413696	-0.066407	0.110135
8	1	0	-2.579704	0.457045	1.053505
9	1	0	-2.760096	0.579736	-0.701904
10	8	0	-0.999922	-0.310466	-0.039538
11	8	0	1.359544	0.105147	-0.613068
12	6	0	-3.145874	-1.388903	0.090477
13	1	0	-2.800820	-2.037043	0.896996
14	1	0	-4.215146	-1.217564	0.224708
15	1	0	-2.997268	-1.904490	-0.858889
16	6	0	3.316762	-1.096371	0.098808
17	1	0	3.731943	-1.732428	0.882509
18	1	0	3.360896	-1.643750	-0.843400
19	1	0	3.941777	-0.206897	0.013654

4_(P_III)_method_B_DMSO.log

Input orientation:

Center Number	Atomic Number	Atomic Type	Coordinates (Angstroms) X	Y	Z
1	15	0	-0.039016	0.898779	-0.560618
2	8	0	0.066256	1.710860	0.878509
3	1	0	0.264976	2.644130	0.740726
4	6	0	1.886165	-0.743803	0.435391
5	1	0	1.276339	-1.643305	0.508626
6	1	0	1.814363	-0.198485	1.375926
7	6	0	-2.416954	-0.063125	0.112691
8	1	0	-2.577852	0.463551	1.054595
9	1	0	-2.762554	0.582610	-0.699417
10	8	0	-1.005096	-0.312503	-0.040418
11	8	0	1.352827	0.084995	-0.628861
12	6	0	-3.154168	-1.384458	0.096865
13	1	0	-2.807624	-2.030758	0.903155
14	1	0	-4.222215	-1.211407	0.232041
15	1	0	-3.006627	-1.900814	-0.851374
16	6	0	3.323815	-1.081012	0.107517
17	1	0	3.738829	-1.715393	0.891546
18	1	0	3.391958	-1.618081	-0.838388
19	1	0	3.931078	-0.178575	0.039969

4_(P_III)_method_B_DMSO_smd.log

Input orientation:

Center Number	Atomic Number	Atomic Type	Coordinates (Angstroms) X	Y	Z
1	15	0	-0.042542	0.866662	-0.569116
2	8	0	0.089621	1.694515	0.859539
3	1	0	0.315724	2.620143	0.699943
4	6	0	1.874667	-0.810182	0.381683
5	1	0	1.331501	-1.754399	0.357172
6	1	0	1.709271	-0.340056	1.350734
7	6	0	-2.419332	-0.057390	0.143510
8	1	0	-2.560395	0.454593	1.096850
9	1	0	-2.762931	0.609133	-0.652896
10	8	0	-1.011967	-0.329151	-0.030418
11	8	0	1.342222	0.047374	-0.661737
12	6	0	-3.181925	-1.361545	0.114268
13	1	0	-2.841413	-2.029933	0.906107
14	1	0	-4.244738	-1.166240	0.266224
15	1	0	-3.059371	-1.865907	-0.844855
16	6	0	3.348662	-1.023639	0.128117
17	1	0	3.757297	-1.684945	0.894280
18	1	0	3.516080	-1.485927	-0.845504
19	1	0	3.894065	-0.079899	0.164583

4_(P_III)_method_B_MeOH.log

Input orientation:

Center Number	Atomic Number	Atomic Type	Coordinates (Angstroms) X	Y	Z
1	15	0	-0.038834	0.898898	-0.561144
2	8	0	0.066073	1.711075	0.878122
3	1	0	0.264839	2.644281	0.740183
4	6	0	1.885530	-0.744328	0.434827
5	1	0	1.275824	-1.644035	0.506574
6	1	0	1.812647	-0.200003	1.375869
7	6	0	-2.416510	-0.063028	0.112708
8	1	0	-2.577024	0.463775	1.054623
9	1	0	-2.762529	0.582601	-0.699341
10	8	0	-1.004862	-0.312418	-0.040989
11	8	0	1.353045	0.085326	-0.629070
12	6	0	-3.153740	-1.384378	0.097378
13	1	0	-2.806753	-2.030548	0.903570
14	1	0	-4.221752	-1.211403	0.233003
15	1	0	-3.006529	-1.900873	-0.850831
16	6	0	3.323591	-1.080828	0.107954
17	1	0	3.738095	-1.715950	0.891667
18	1	0	3.392720	-1.616800	-0.838492

19	1	0	3.930667	-0.178158	0.041872

4_(P_III)_method_B_MeOH_smd.log

Input orientation:

Center Atomic Atomic Coordinates (Angstroms)

Number	Number	Type	X	Y	Z
1	15	0	-0.031671	0.905819	-0.566268
2	8	0	0.073509	1.689066	0.884921
3	1	0	0.280404	2.625862	0.768850
4	6	0	1.872159	-0.795580	0.395883
5	1	0	1.300219	-1.722510	0.374573
6	1	0	1.728518	-0.317897	1.364539
7	6	0	-2.423677	-0.050081	0.081958
8	1	0	-2.577423	0.524478	0.996737
9	1	0	-2.770930	0.550294	-0.762959
10	8	0	-1.007387	-0.309297	-0.068310
11	8	0	1.355464	0.080726	-0.645552
12	6	0	-3.155903	-1.369957	0.142454
13	1	0	-2.808603	-1.969595	0.984930
14	1	0	-4.224271	-1.188261	0.270177
15	1	0	-3.011744	-1.939806	-0.776196
16	6	0	3.335928	-1.049749	0.124170
17	1	0	3.735821	-1.718862	0.888091
18	1	0	3.477287	-1.520679	-0.849521
19	1	0	3.906798	-0.120763	0.150006

4_(P_III)_method_B_THF.log

Input orientation:

Center Atomic Atomic Coordinates (Angstroms)

Number	Number	Type	X	Y	Z
1	15	0	-0.037061	0.900091	-0.566331
2	8	0	0.063722	1.712958	0.874470
3	1	0	0.262638	2.645627	0.735295
4	6	0	1.879878	-0.748195	0.430237
5	1	0	1.270648	-1.649163	0.489725
6	1	0	1.798188	-0.211998	1.375367
7	6	0	-2.412101	-0.062309	0.113060
8	1	0	-2.568587	0.464952	1.055556
9	1	0	-2.762375	0.583042	-0.697741
10	8	0	-1.002603	-0.311789	-0.046933
11	8	0	1.355235	0.088665	-0.630722
12	6	0	-3.149571	-1.383778	0.101564
13	1	0	-2.798106	-2.029421	0.906143
14	1	0	-4.217195	-1.211636	0.241970
15	1	0	-3.005814	-1.900788	-0.846838
16	6	0	3.321165	-1.079742	0.111877
17	1	0	3.731141	-1.721166	0.892965
18	1	0	3.398196	-1.606621	-0.838979
19	1	0	3.927098	-0.175524	0.057797

4_(P_III)_method_B_THF_smd.log

Input orientation:

Center Atomic Atomic Coordinates (Angstroms)

Number	Number	Type	X	Y	Z
1	15	0	-0.038076	0.866965	-0.585623
2	8	0	0.085561	1.703794	0.840318
3	1	0	0.311809	2.627965	0.677119
4	6	0	1.868959	-0.806477	0.388611
5	1	0	1.318895	-1.746931	0.369449
6	1	0	1.703320	-0.328281	1.353868
7	6	0	-2.415941	-0.055526	0.129645
8	1	0	-2.557913	0.473288	1.073857
9	1	0	-2.763522	0.595223	-0.678368
10	8	0	-1.009676	-0.325410	-0.041337
11	8	0	1.346523	0.046673	-0.661754
12	6	0	-3.173347	-1.363695	0.124849
13	1	0	-2.828009	-2.015840	0.927890
14	1	0	-4.237196	-1.171906	0.274182
15	1	0	-3.048116	-1.884839	-0.824763
16	6	0	3.342627	-1.031270	0.139950
17	1	0	3.746580	-1.690032	0.910795
18	1	0	3.508609	-1.499948	-0.830706
19	1	0	3.893410	-0.090547	0.170503

4_(P_III)_method_B_toluene.log

Input orientation:

Center Atomic Atomic Coordinates (Angstroms)

Number	Number	Type	X	Y	Z
1	15	0	-0.033063	0.904302	-0.575913
2	8	0	0.056329	1.714429	0.870296
3	1	0	0.253297	2.646940	0.732060
4	6	0	1.870906	-0.750229	0.425003
5	1	0	1.258919	-1.650361	0.469383
6	1	0	1.779849	-0.223799	1.375081
7	6	0	-2.403198	-0.061454	0.111994
8	1	0	-2.550808	0.465839	1.056242
9	1	0	-2.763043	0.584088	-0.695158
10	8	0	-0.998236	-0.310690	-0.062374
11	8	0	1.360201	0.097110	-0.631904
12	6	0	-3.140554	-1.383494	0.107585
13	1	0	-2.779500	-2.028636	0.908191
14	1	0	-4.207314	-1.213651	0.258268
15	1	0	-3.004113	-1.900683	-0.841712
16	6	0	3.315010	-1.081100	0.117469
17	1	0	3.717642	-1.731579	0.895130
18	1	0	3.399843	-1.596700	-0.838782
19	1	0	3.922332	-0.177123	0.077623

4_(P_III)_method_B_toluene_smd.log

Input orientation:

Center Atomic Atomic Coordinates (Angstroms)

Number	Number	Type	X	Y	Z
1	15	0	-0.032793	0.880708	-0.591222
2	8	0	0.078021	1.704030	0.846606
3	1	0	0.298550	2.629898	0.694183
4	6	0	1.859690	-0.801989	0.387161
5	1	0	1.299655	-1.736519	0.359977
6	1	0	1.693516	-0.328604	1.355014
7	6	0	-2.408057	-0.053897	0.118805

8	1	0	-2.547049	0.483539	1.058988
9	1	0	-2.765696	0.587635	-0.692864
10	8	0	-1.005280	-0.318665	-0.059661
11	8	0	1.352340	0.062045	-0.658589
12	6	0	-3.158579	-1.366605	0.131249
13	1	0	-2.803270	-2.008470	0.937880
14	1	0	-4.223195	-1.182304	0.284562
15	1	0	-3.033676	-1.896587	-0.813228
16	6	0	3.332453	-1.039260	0.142364
17	1	0	3.729775	-1.707054	0.908901
18	1	0	3.496810	-1.500904	-0.831651
19	1	0	3.891284	-0.103791	0.180008

4_(P_III)_method_B_water.log

Input orientation:

Center Number	Atomic Number	Atomic Type	Coordinates (Angstroms) X	Y	Z
1	15	0	-0.039190	0.898663	-0.560123
2	8	0	0.066421	1.710656	0.878871
3	1	0	0.265092	2.643987	0.741235
4	6	0	1.886776	-0.743283	0.435943
5	1	0	1.276825	-1.642578	0.510629
6	1	0	1.816028	-0.196993	1.375980
7	6	0	-2.417372	-0.063222	0.112685
8	1	0	-2.578629	0.463313	1.054592
9	1	0	-2.762578	0.582634	-0.699461
10	8	0	-1.005318	-0.312590	-0.039878
11	8	0	1.352620	0.084680	-0.628657
12	6	0	-3.154574	-1.384537	0.096362
13	1	0	-2.808448	-2.030980	0.902726
14	1	0	-4.222653	-1.211415	0.231121
15	1	0	-3.006728	-1.900738	-0.851919
16	6	0	3.324022	-1.081199	0.107097
17	1	0	3.739529	-1.714858	0.891436
18	1	0	3.391202	-1.619337	-0.838276
19	1	0	3.931473	-0.178995	0.038120

4_(P_III)_method_B_water_smd.log

Input orientation:

Center Number	Atomic Number	Atomic Type	Coordinates (Angstroms) X	Y	Z
1	15	0	-0.031307	0.908071	-0.565971
2	8	0	0.070763	1.685599	0.889036
3	1	0	0.272036	2.623469	0.773933
4	6	0	1.873530	-0.789647	0.398244
5	1	0	1.296042	-1.712702	0.384132
6	1	0	1.736402	-0.305306	1.364007
7	6	0	-2.421391	-0.051133	0.082439
8	1	0	-2.570481	0.519219	0.999992
9	1	0	-2.769996	0.553356	-0.758257
10	8	0	-1.005423	-0.311723	-0.073944
11	8	0	1.357851	0.084664	-0.645946
12	6	0	-3.155479	-1.369871	0.139344
13	1	0	-2.806278	-1.973679	0.977443
14	1	0	-4.222431	-1.186401	0.271814
15	1	0	-3.015840	-1.935051	-0.782326
16	6	0	3.334463	-1.054202	0.122654
17	1	0	3.732389	-1.720779	0.889068
18	1	0	3.469076	-1.531834	-0.848190
19	1	0	3.910575	-0.128843	0.141010

4_(P_III)_method_D_DCM.log

Input orientation:

Center Number	Atomic Number	Atomic Type	Coordinates (Angstroms) X	Y	Z
1	15	0	-0.018376	0.939885	-0.621226
2	8	0	0.048162	1.707644	0.872243
3	1	0	0.241115	2.651512	0.769638
4	6	0	1.861018	-0.742442	0.397974
5	1	0	1.235212	-1.638721	0.413305
6	1	0	1.740397	-0.214146	1.348097
7	6	0	-2.397652	-0.043597	0.061105
8	1	0	-2.513394	0.537650	0.982225
9	1	0	-2.781995	0.556274	-0.774525
10	8	0	-0.992416	-0.313457	-0.144280
11	8	0	1.395731	0.119283	-0.678726
12	6	0	-3.129223	-1.368679	0.154717
13	1	0	-2.740179	-1.966231	0.984813
14	1	0	-4.196079	-1.192212	0.326369
15	1	0	-3.016072	-1.940910	-0.770964
16	6	0	3.314638	-1.081758	0.133883
17	1	0	3.687184	-1.744031	0.922555
18	1	0	3.426844	-1.593387	-0.826946
19	1	0	3.930805	-0.177522	0.122009

4_(P_V)_method_A.log

Input orientation:

Center Number	Atomic Number	Atomic Type	Coordinates (Angstroms) X	Y	Z
1	15	0	0.004937	0.483206	-0.409215
2	1	0	0.227716	0.901599	-1.729005
3	8	0	0.158674	1.534706	0.630818
4	6	0	2.293672	-0.662314	0.352843
5	1	0	2.939675	-0.196629	-0.401761
6	6	0	-2.177793	-0.729204	0.522681
7	1	0	-1.660910	-1.629705	0.871103
8	1	0	-2.180423	0.013549	1.326643
9	8	0	0.961647	-0.810737	-0.209691
10	8	0	-1.439255	-0.190291	-0.609358
11	1	0	2.239262	0.000628	1.220866
12	6	0	2.802812	-2.041681	0.726365
13	1	0	2.833542	-2.697537	-0.148789
14	1	0	3.815580	-1.962229	1.136021
15	1	0	2.159311	-2.501295	1.482543
16	6	0	-3.584667	-1.045623	0.053180
17	1	0	-4.162968	-1.467116	0.882418
18	1	0	-4.089875	-0.140795	-0.296979
19	1	0	-3.568144	-1.773813	-0.763126

4_(P_V)_method_A_DCM.log

Input orientation:

Center	Atomic	Atomic	Coordinates (Angstroms)		
Number	Number	Type	X	Y	Z
1	15	0	-0.001689	0.929578	-0.299613
2	1	0	0.131670	1.554450	-1.545178
3	8	0	0.113261	1.848520	0.873146
4	6	0	2.438819	-0.093729	0.099021
5	1	0	2.915629	0.579968	-0.621591
6	6	0	-2.033010	-0.608861	0.527027
7	1	0	-1.377058	-1.450989	0.766403
8	1	0	-2.142376	0.023326	1.412766
9	8	0	1.050832	-0.296257	-0.306111
10	8	0	-1.399874	0.179369	-0.529866
11	1	0	2.449031	0.379062	1.084524
12	6	0	3.122346	-1.446466	0.119970
13	1	0	3.096278	-1.914328	-0.868653
14	1	0	4.169199	-1.319578	0.414484
15	1	0	2.640193	-2.115522	0.839022
16	6	0	-3.377687	-1.081198	0.012364
17	1	0	-3.871793	-1.677515	0.786294
18	1	0	-4.022185	-0.232069	-0.233417
19	1	0	-3.258389	-1.703609	-0.879407

4_(P_V)_method_A_DCM_smd.log

Input orientation:

Center	Atomic	Atomic	Coordinates (Angstroms)		
Number	Number	Type	X	Y	Z
1	15	0	0.004854	0.879648	-0.296742
2	1	0	0.183270	1.452130	-1.562105
3	8	0	0.087313	1.846375	0.840352
4	6	0	2.444440	-0.108580	0.150349
5	1	0	2.870582	0.629596	-0.539184
6	6	0	-2.078584	-0.587073	0.541217
7	1	0	-1.450998	-1.427645	0.852796
8	1	0	-2.206201	0.096970	1.385357
9	8	0	1.051845	-0.349813	-0.222558
10	8	0	-1.390934	0.126258	-0.536611
11	1	0	2.466529	0.302398	1.163596
12	6	0	3.185912	-1.426000	0.068069
13	1	0	3.157402	-1.831645	-0.948467
14	1	0	4.233498	-1.268857	0.347914
15	1	0	2.753302	-2.162040	0.753715
16	6	0	-3.413104	-1.061465	0.007189
17	1	0	-3.945213	-1.601586	0.798082
18	1	0	-4.032117	-0.215723	-0.309351
19	1	0	-3.278598	-1.738795	-0.842431

4_(P_V)_method_B.log

Input orientation:

Center	Atomic	Atomic	Coordinates (Angstroms)		
Number	Number	Type	X	Y	Z
1	15	0	-0.009101	0.900419	-0.304370
2	1	0	0.060787	1.605875	-1.508402
3	8	0	0.108882	1.719788	0.906455
4	6	0	2.438968	-0.063558	0.039992
5	1	0	2.975578	0.552860	-0.684565
6	6	0	-1.982169	-0.681043	0.485602
7	1	0	-1.372260	-1.572880	0.635193
8	1	0	-1.995104	-0.104917	1.410632
9	8	0	1.085502	-0.253113	-0.438895
10	8	0	-1.364121	0.122058	-0.551029
11	1	0	2.402947	0.469561	0.988851
12	6	0	3.090669	-1.420637	0.183526
13	1	0	3.101620	-1.950787	-0.768159
14	1	0	4.120262	-1.298998	0.522113
15	1	0	2.559064	-2.029692	0.913899
16	6	0	-3.379961	-1.040107	0.035696
17	1	0	-3.861976	-1.656603	0.795277
18	1	0	-3.980857	-0.143739	-0.112343
19	1	0	-3.355534	-1.600335	-0.898284

4_(P_V)_method_B_DCM.log

Input orientation:

Center	Atomic	Atomic	Coordinates (Angstroms)		
Number	Number	Type	X	Y	Z
1	15	0	-0.002497	0.905330	-0.284220
2	1	0	0.129126	1.543022	-1.518199
3	8	0	0.103757	1.806588	0.876700
4	6	0	2.436356	-0.086494	0.083745
5	1	0	2.907007	0.568984	-0.650557
6	6	0	-2.027446	-0.623054	0.525146
7	1	0	-1.389847	-1.475077	0.758976
8	1	0	-2.127600	-0.001660	1.413983
9	8	0	1.049263	-0.286860	-0.303409
10	8	0	-1.376113	0.162074	-0.514256
11	1	0	2.456716	0.404710	1.054914
12	6	0	3.116882	-1.434795	0.127183
13	1	0	3.081479	-1.921527	-0.846771
14	1	0	4.162081	-1.303651	0.408116
15	1	0	2.642914	-2.085022	0.861712
16	6	0	-3.374572	-1.067589	0.006294
17	1	0	-3.877248	-1.661201	0.770153
18	1	0	-4.002445	-0.209496	-0.230520
19	1	0	-3.264619	-1.680129	-0.887803

4_(P_V)_method_B_DCM_smd.log

Input orientation:

Center	Atomic	Atomic	Coordinates (Angstroms)		
Number	Number	Type	X	Y	Z
1	15	0	0.004495	0.845556	-0.290747
2	1	0	0.186682	1.410795	-1.553636
3	8	0	0.075288	1.812401	0.819467
4	6	0	2.438741	-0.103853	0.154878
5	1	0	2.852588	0.637263	-0.531691
6	6	0	-2.077610	-0.598064	0.541047
7	1	0	-1.473676	-1.447617	0.859419
8	1	0	-2.195529	0.086131	1.380620
9	8	0	1.050405	-0.350072	-0.207199
10	8	0	-1.365916	0.096344	-0.524929
11	1	0	2.464257	0.304092	1.164248
12	6	0	3.191472	-1.408622	0.065083
13	1	0	3.157018	-1.812909	-0.946718
14	1	0	4.235870	-1.240542	0.331886

15	1	0	2.777362	-2.146748	0.752530
16	6	0	-3.414616	-1.044758	0.003560
17	1	0	-3.960095	-1.567977	0.790388
18	1	0	-4.011729	-0.191344	-0.318261
19	1	0	-3.291812	-1.725923	-0.838759

4_(P_V)_method_B_DMSO.log

Input orientation:

Center Number	Atomic Number	Atomic Type	Coordinates (Angstroms) X	Y	Z
1	15	0	-0.001562	0.907394	-0.280427
2	1	0	0.139286	1.536392	-1.517365
3	8	0	0.101060	1.819435	0.874017
4	6	0	2.436901	-0.088612	0.087812
5	1	0	2.897831	0.567511	-0.651664
6	6	0	-2.032254	-0.617057	0.529155
7	1	0	-1.391155	-1.464000	0.770835
8	1	0	-2.142997	0.007277	1.414587
9	8	0	1.043959	-0.288880	-0.282010
10	8	0	-1.377645	0.169025	-0.509016
11	1	0	2.468978	0.400868	1.059531
12	6	0	3.117430	-1.436934	0.121553
13	1	0	3.071729	-1.921653	-0.852976
14	1	0	4.165529	-1.305574	0.391066
15	1	0	2.651892	-2.088348	0.860430
16	6	0	-3.372557	-1.071604	0.001967
17	1	0	-3.876967	-1.664672	0.764991
18	1	0	-4.004030	-0.218445	-0.243138
19	1	0	-3.252232	-1.687970	-0.888161

4_(P_V)_method_B_DMSO_smd.log

Input orientation:

Center Number	Atomic Number	Atomic Type	Coordinates (Angstroms) X	Y	Z
1	15	0	0.005093	0.844990	-0.275040
2	1	0	0.208346	1.404524	-1.537423
3	8	0	0.064873	1.817830	0.830914
4	6	0	2.440290	-0.102132	0.146234
5	1	0	2.835638	0.621074	-0.569708
6	6	0	-2.078209	-0.608414	0.533988
7	1	0	-1.478585	-1.468044	0.832382
8	1	0	-2.188203	0.059270	1.387786
9	8	0	1.042451	-0.357249	-0.172938
10	8	0	-1.367229	0.101878	-0.522687
11	1	0	2.492826	0.330709	1.144209
12	6	0	3.193879	-1.406866	0.071518
13	1	0	3.140620	-1.835362	-0.929583
14	1	0	4.242883	-1.229238	0.313044
15	1	0	2.796139	-2.129196	0.784994
16	6	0	-3.420932	-1.037580	-0.002644
17	1	0	-3.962157	-1.574940	0.777489
18	1	0	-4.017500	-0.175331	-0.301574
19	1	0	-3.307028	-1.701770	-0.859774

4_(P_V)_method_B_MeOH.log

Input orientation:

Center Number	Atomic Number	Atomic Type	Coordinates (Angstroms) X	Y	Z
1	15	0	-0.001662	0.907155	-0.280897
2	1	0	0.138152	1.537091	-1.517529
3	8	0	0.101410	1.818033	0.874242
4	6	0	2.436815	-0.088406	0.087426
5	1	0	2.898849	0.567759	-0.651368
6	6	0	-2.031741	-0.617663	0.528740
7	1	0	-1.391007	-1.465133	0.769631
8	1	0	-2.141355	0.006414	1.414501
9	8	0	1.044540	-0.288690	-0.284434
10	8	0	-1.377484	0.168261	-0.509606
11	1	0	2.467504	0.401144	1.059154
12	6	0	3.117399	-1.436703	0.122120
13	1	0	3.072929	-1.921529	-0.852412
14	1	0	4.165158	-1.305358	0.392992
15	1	0	2.650897	-2.088094	0.860407
16	6	0	-3.372777	-1.071177	0.002465
17	1	0	-3.877002	-1.664253	0.765618
18	1	0	-4.003871	-0.217505	-0.241811
19	1	0	-3.253560	-1.687195	-0.888052

4_(P_V)_method_B_MeOH_smd.log

Input orientation:

Center Number	Atomic Number	Atomic Type	Coordinates (Angstroms) X	Y	Z
1	15	0	0.004197	0.886911	-0.275178
2	1	0	0.179267	1.502319	-1.512537
3	8	0	0.097329	1.816268	0.873711
4	6	0	2.439540	-0.102895	0.116321
5	1	0	2.865557	0.592195	-0.608336
6	6	0	-2.069106	-0.578361	0.551390
7	1	0	-1.425265	-1.399743	0.863343
8	1	0	-2.225261	0.094335	1.393342
9	8	0	1.035317	-0.317997	-0.227086
10	8	0	-1.378630	0.166315	-0.503633
11	1	0	2.483225	0.346409	1.106971
12	6	0	3.142979	-1.435976	0.079864
13	1	0	3.087275	-1.882655	-0.913168
14	1	0	4.194408	-1.291746	0.332944
15	1	0	2.708088	-2.126654	0.803007
16	6	0	-3.375167	-1.080152	-0.009385
17	1	0	-3.904850	-1.636159	0.765633
18	1	0	-4.007530	-0.251806	-0.329929
19	1	0	-3.208178	-1.746456	-0.856090

4_(P_V)_method_B_THF.log

Input orientation:

Center Number	Atomic Number	Atomic Type	Coordinates (Angstroms) X	Y	Z
1	15	0	-0.002719	0.904909	-0.284956
2	1	0	0.126825	1.544675	-1.518188
3	8	0	0.104234	1.803608	0.877515

4	6	0	2.436301	-0.085935	0.082640
5	1	0	2.909110	0.569076	-0.650779
6	6	0	-2.026279	-0.624573	0.524151
7	1	0	-1.389517	-1.477830	0.755963
8	1	0	-2.123904	-0.004034	1.413887
9	8	0	1.050482	-0.286286	-0.308161
10	8	0	-1.375743	0.160503	-0.515437
11	1	0	2.454223	0.405994	1.053497
12	6	0	3.116637	-1.434305	0.128625
13	1	0	3.083328	-1.921815	-0.845014
14	1	0	4.161229	-1.303249	0.411908
15	1	0	2.640811	-2.083952	0.862461
16	6	0	-3.375010	-1.066645	0.007233
17	1	0	-3.877255	-1.660534	0.771186
18	1	0	-4.002032	-0.207352	-0.227442
19	1	0	-3.267526	-1.678103	-0.887903

4_(P_V)_method_B_THF_smd.log

Input orientation:

Center Number	Atomic Number	Atomic Type	Coordinates (Angstroms) X	Y	Z
1	15	0	0.002765	0.853614	-0.285789
2	1	0	0.183156	1.428238	-1.545448
3	8	0	0.076529	1.810588	0.831234
4	6	0	2.437044	-0.100505	0.140283
5	1	0	2.856403	0.623073	-0.561777
6	6	0	-2.067626	-0.609610	0.532910
7	1	0	-1.462474	-1.468307	0.823712
8	1	0	-2.172592	0.054894	1.389958
9	8	0	1.048162	-0.344980	-0.216913
10	8	0	-1.368445	0.106128	-0.525997
11	1	0	2.466348	0.329210	1.140550
12	6	0	3.183253	-1.410877	0.079352
13	1	0	3.150457	-1.835786	-0.924084
14	1	0	4.227553	-1.243444	0.347191
15	1	0	2.761850	-2.132560	0.779631
16	6	0	-3.413443	-1.041405	0.004536
17	1	0	-3.948526	-1.583504	0.785661
18	1	0	-4.013903	-0.179591	-0.287332
19	1	0	-3.303314	-1.701025	-0.856490

4_(P_V)_method_B_toluene.log

Input orientation:

Center Number	Atomic Number	Atomic Type	Coordinates (Angstroms) X	Y	Z
1	15	0	-0.006091	0.900307	-0.295270
2	1	0	0.100076	1.564072	-1.519025
3	8	0	0.104871	1.770017	0.885007
4	6	0	2.436893	-0.076602	0.067006
5	1	0	2.934340	0.565011	-0.662778
6	6	0	-2.007660	-0.652555	0.506073
7	1	0	-1.384540	-1.526347	0.697013
8	1	0	-2.063247	-0.053520	1.414293
9	8	0	1.064770	-0.276965	-0.360957
10	8	0	-1.371174	0.140883	-0.532009
11	1	0	2.430224	0.432041	1.029513
12	6	0	3.111170	-1.426696	0.152967
13	1	0	3.098782	-1.931455	-0.812351
14	1	0	4.149324	-1.296296	0.459909
15	1	0	2.614006	-2.061957	0.885229
16	6	0	-3.381958	-1.050972	0.020453
17	1	0	-3.874856	-1.655837	0.782117
18	1	0	-3.995668	-0.171612	-0.170754
19	1	0	-3.316065	-1.637364	-0.895250

4_(P_V)_method_B_toluene_smd.log

Input orientation:

Center Number	Atomic Number	Atomic Type	Coordinates (Angstroms) X	Y	Z
1	15	0	-0.001503	0.873071	-0.284347
2	1	0	0.135351	1.502626	-1.523759
3	8	0	0.094085	1.774744	0.872820
4	6	0	2.438443	-0.089945	0.099389
5	1	0	2.900240	0.598089	-0.612381
6	6	0	-2.045216	-0.623074	0.525280
7	1	0	-1.432366	-1.486526	0.786148
8	1	0	-2.136143	0.020899	1.399667
9	8	0	1.061402	-0.314227	-0.301721
10	8	0	-1.368510	0.117734	-0.526913
11	1	0	2.443351	0.376500	1.083531
12	6	0	3.154261	-1.419269	0.110754
13	1	0	3.139237	-1.882057	-0.876005
14	1	0	4.194393	-1.269405	0.404009
15	1	0	2.693040	-2.103272	0.823335
16	6	0	-3.398950	-1.047266	0.009483
17	1	0	-3.921561	-1.610287	0.784229
18	1	0	-4.005558	-0.180440	-0.252393
19	1	0	-3.300799	-1.683742	-0.869939

4_(P_V)_method_B_water.log

Input orientation:

Center Number	Atomic Number	Atomic Type	Coordinates (Angstroms) X	Y	Z
1	15	0	-0.001469	0.907622	-0.279969
2	1	0	0.140354	1.535746	-1.517190
3	8	0	0.100722	1.820747	0.873828
4	6	0	2.436990	-0.088800	0.088157
5	1	0	2.896874	0.567255	-0.651989
6	6	0	-2.032733	-0.616500	0.529540
7	1	0	-1.391296	-1.462954	0.771946
8	1	0	-2.144532	0.008061	1.414672
9	8	0	1.043415	-0.289050	-0.279716
10	8	0	-1.377794	0.169744	-0.508456
11	1	0	2.470394	0.400647	1.059850
12	6	0	3.117452	-1.437151	0.121032
13	1	0	3.070569	-1.921800	-0.853475
14	1	0	4.165876	-1.305779	0.389244
15	1	0	2.652827	-2.088557	0.860492
16	6	0	-3.372350	-1.072004	0.001491
17	1	0	-3.876934	-1.665075	0.764385
18	1	0	-4.004177	-0.219320	-0.244375
19	1	0	-3.250990	-1.688681	-0.888281

4_(P_V)_method_B_water_smd.log

Input orientation:

Center	Atomic	Atomic	Coordinates (Angstroms)		
Number	Number	Type	X	Y	Z
1	15	0	0.004307	0.886378	-0.273895
2	1	0	0.184273	1.503937	-1.508393
3	8	0	0.096192	1.813053	0.878353
4	6	0	2.438570	-0.103110	0.113668
5	1	0	2.860884	0.587757	-0.616367
6	6	0	-2.068206	-0.579622	0.548910
7	1	0	-1.423338	-1.401545	0.855492
8	1	0	-2.221624	0.090226	1.393028
9	8	0	1.032781	-0.320872	-0.224139
10	8	0	-1.380087	0.169216	-0.505603
11	1	0	2.484488	0.351580	1.101270
12	6	0	3.142822	-1.435595	0.082623
13	1	0	3.084296	-1.887362	-0.907407
14	1	0	4.194345	-1.288449	0.331385
15	1	0	2.710935	-2.121963	0.810925
16	6	0	-3.375661	-1.079306	-0.009726
17	1	0	-3.902341	-1.637596	0.764975
18	1	0	-4.008539	-0.250052	-0.325247
19	1	0	-3.210902	-1.742524	-0.858664

4_(P_V)_method_D_DCM.log

Input orientation:

Center	Atomic	Atomic	Coordinates (Angstroms)		
Number	Number	Type	X	Y	Z
1	15	0	-0.006338	0.953594	-0.369277
2	1	0	0.108251	1.566825	-1.621631
3	8	0	0.131592	1.880077	0.794387
4	6	0	2.412889	-0.084821	0.070787
5	1	0	2.928196	0.550201	-0.657778
6	6	0	-1.973208	-0.610743	0.507060
7	1	0	-1.277138	-1.427349	0.718826
8	1	0	-2.073841	0.014605	1.398434
9	8	0	1.038396	-0.276039	-0.378078
10	8	0	-1.408711	0.203907	-0.567110
11	1	0	2.396130	0.426701	1.036247
12	6	0	3.062254	-1.449216	0.169424
13	1	0	3.058579	-1.954447	-0.800799
14	1	0	4.100550	-1.335648	0.496884
15	1	0	2.536429	-2.076481	0.895234
16	6	0	-3.314849	-1.129923	0.035992
17	1	0	-3.761847	-1.746454	0.822244
18	1	0	-3.995743	-0.302931	-0.185049
19	1	0	-3.200655	-1.743261	-0.862490

5_(P_III)_method_A.log

Input orientation:

Center	Atomic	Atomic	Coordinates (Angstroms)		
Number	Number	Type	X	Y	Z
1	15	0	-0.066716	0.780932	-0.869973
2	8	0	-0.822613	2.202095	-0.665632
3	1	0	-1.051922	2.445841	0.247521
4	9	0	-1.029452	-0.187439	0.020705
5	9	0	1.096079	0.907956	0.265823

5_(P_III)_method_A_DCM.log

Input orientation:

Center	Atomic	Atomic	Coordinates (Angstroms)		
Number	Number	Type	X	Y	Z
1	15	0	-0.068334	0.784741	-0.873002
2	8	0	-0.821479	2.201690	-0.663449
3	1	0	-1.058855	2.460248	0.245304
4	9	0	-1.026060	-0.195387	0.022027
5	9	0	1.100104	0.898094	0.267564

5_(P_III)_method_A_DCM_smd.log

Input orientation:

Center	Atomic	Atomic	Coordinates (Angstroms)		
Number	Number	Type	X	Y	Z
1	15	0	-0.068863	0.786105	-0.875591
2	8	0	-0.821090	2.201374	-0.666684
3	1	0	-1.057133	2.455765	0.248160
4	9	0	-1.026941	-0.193892	0.023845
5	9	0	1.099404	0.900033	0.268713

5_(P_III)_method_B.log

Input orientation:

Center	Atomic	Atomic	Coordinates (Angstroms)		
Number	Number	Type	X	Y	Z
1	15	0	0.001368	0.000006	0.519923
2	8	0	-1.440025	0.000451	-0.179848
3	1	0	-1.457134	0.000291	-1.147046
4	9	0	0.715145	1.185126	-0.290833
5	9	0	0.714385	-1.185686	-0.290653

5_(P_III)_method_B_DCM.log

Input orientation:

Center	Atomic	Atomic	Coordinates (Angstroms)		
Number	Number	Type	X	Y	Z
1	15	0	-0.006734	-0.000025	-0.515952
2	8	0	-1.439394	0.000869	0.190580
3	1	0	-1.469072	0.001194	1.159115
4	9	0	0.722221	-1.186087	0.289057
5	9	0	0.723596	1.185224	0.289034

5_(P_III)_method_B_DCM_smd.log

Input orientation:

Center Number	Atomic Number	Atomic Type	Coordinates (Angstroms) X	Y	Z
1	15	0	-0.008576	-0.000022	-0.517472
2	8	0	-1.439712	0.000836	0.187674
3	1	0	-1.465355	0.001231	1.160347
4	9	0	0.721449	-1.186034	0.290639
5	9	0	0.722811	1.185164	0.290645

5_(P_III)_method_C_DCM.log

Input orientation:

Center Number	Atomic Number	Atomic Type	Coordinates (Angstroms) X	Y	Z
1	15	0	-0.002083	0.000017	0.524762
2	8	0	-1.446519	0.000446	-0.181235
3	1	0	-1.469449	0.000283	-1.150534
4	9	0	0.726292	1.188880	-0.290808
5	9	0	0.725499	-1.189437	-0.290642

5_(P_III)_method_C_DCM_smd.log

Input orientation:

Center Number	Atomic Number	Atomic Type	Coordinates (Angstroms) X	Y	Z
1	15	0	-0.003740	0.000008	0.526168
2	8	0	-1.446762	0.000467	-0.178503
3	1	0	-1.466494	0.000275	-1.151900
4	9	0	0.725754	1.188959	-0.292194
5	9	0	0.724981	-1.189521	-0.292028

5_(P_III)_method_D_DCM.log

Input orientation:

Center Number	Atomic Number	Atomic Type	Coordinates (Angstroms) X	Y	Z
1	15	0	-0.008381	0.000021	-0.532646
2	8	0	-1.455485	0.000797	0.191247
3	1	0	-1.475427	0.001216	1.165179
4	9	0	0.734215	-1.201458	0.294044
5	9	0	0.735695	1.200599	0.294010

5_(P_III)_method_E_DCM.log

Input orientation:

Center Number	Atomic Number	Atomic Type	Coordinates (Angstroms) X	Y	Z
1	15	0	-0.004236	0.000002	0.515284
2	8	0	-1.431236	0.000471	-0.180714
3	1	0	-1.461034	0.000287	-1.144559
4	9	0	0.715485	1.177939	-0.289323
5	9	0	0.714759	-1.178511	-0.289145

5_(P_III)_method_E_DCM_smd.log

Input orientation:

Center Number	Atomic Number	Atomic Type	Coordinates (Angstroms) X	Y	Z
1	15	0	0.003486	-0.000003	0.517629
2	8	0	1.429114	0.000450	-0.177737
3	1	0	1.456512	0.000564	-1.145574
4	9	0	-0.717621	-1.178575	-0.287888
5	9	0	-0.718378	1.178092	-0.287912

5_(P_V)_method_A.log

Input orientation:

Center Number	Atomic Number	Atomic Type	Coordinates (Angstroms) X	Y	Z
1	8	0	-0.000355	1.895334	-0.421741
2	9	0	-1.073704	0.194612	1.212250
3	9	0	1.303237	0.037141	0.828148
4	15	0	-0.009289	0.521186	0.086339
5	1	0	-0.224091	-0.522439	-0.815064

5_(P_V)_method_A_DCM.log

Input orientation:

Center Number	Atomic Number	Atomic Type	Coordinates (Angstroms) X	Y	Z
1	8	0	0.001510	1.894338	-0.409791
2	9	0	-1.073945	0.203724	1.214553
3	9	0	1.305358	0.046096	0.830070
4	15	0	-0.011205	0.507622	0.080044
5	1	0	-0.225970	-0.527922	-0.824449

5_(P_V)_method_A_DCM_smd.log

Input orientation:

Center Number	Atomic Number	Atomic Type	Coordinates (Angstroms) X	Y	Z
1	8	0	0.002296	1.896354	-0.405749
2	9	0	-1.072887	0.205376	1.216828
3	9	0	1.305288	0.047823	0.832527
4	15	0	-0.012201	0.505692	0.074672
5	1	0	-0.226769	-0.532263	-0.827611

5_(P_V)_method_B.log

Input orientation:

Center Number	Atomic Number	Atomic Type	Coordinates (Angstroms) X	Y	Z
1	8	0	0.907841	1.205082	0.000000
2	9	0	-0.183980	-0.766670	1.189432
3	9	0	-0.183980	-0.766670	-1.189432
4	15	0	-0.162778	0.233673	0.000000
5	1	0	-1.470796	0.702077	0.000000

5_(P_V)_method_B_DCM.log

Input orientation:

Center Number	Atomic Number	Atomic Type	Coordinates (Angstroms) X	Y	Z
1	8	0	0.911702	1.193286	-0.000000
2	9	0	-0.178827	-0.764522	1.190799
3	9	0	-0.178827	-0.764522	-1.190799
4	15	0	-0.176790	0.233306	-0.000000
5	1	0	-1.477557	0.710526	-0.000000

5_(P_V)_method_B_DCM_smd.log

Input orientation:

Center Number	Atomic Number	Atomic Type	Coordinates (Angstroms) X	Y	Z
1	8	0	0.914667	1.190819	0.000000
2	9	0	-0.176756	-0.765675	1.190485
3	9	0	-0.176756	-0.765675	-1.190485
4	15	0	-0.179939	0.236971	0.000000
5	1	0	-1.481870	0.711348	-0.000000

5_(P_V)_method_C_DCM.log

Input orientation:

Center Number	Atomic Number	Atomic Type	Coordinates (Angstroms) X	Y	Z
1	8	0	0.920099	1.201297	-0.000000
2	9	0	-0.175295	-0.770463	1.196206
3	9	0	-0.175295	-0.770463	-1.196206
4	15	0	-0.178646	0.236101	-0.000000
5	1	0	-1.486062	0.709822	0.000000

5_(P_V)_method_C_DCM_smd.log

Input orientation:

Center Number	Atomic Number	Atomic Type	Coordinates (Angstroms) X	Y	Z
1	8	0	0.923272	1.198670	0.000000
2	9	0	-0.173005	-0.771611	1.195907
3	9	0	-0.173005	-0.771611	-1.195907
4	15	0	-0.181960	0.240105	0.000000
5	1	0	-1.490669	0.710608	-0.000000

5_(P_V)_method_D_DCM.log

Input orientation:

Center Number	Atomic Number	Atomic Type	Coordinates (Angstroms) X	Y	Z
1	8	0	0.928547	1.210488	0.000000
2	9	0	-0.179972	-0.780999	1.207659
3	9	0	-0.179972	-0.780999	-1.207659
4	15	0	-0.179972	0.243913	0.000000
5	1	0	-1.489284	0.715383	0.000000

5_(P_V)_method_E_DCM.log

Input orientation:

Center Number	Atomic Number	Atomic Type	Coordinates (Angstroms) X	Y	Z
1	8	0	0.905788	1.190042	0.000000
2	9	0	-0.174519	-0.761573	1.184511
3	9	0	-0.174519	-0.761573	-1.184511
4	15	0	-0.175734	0.231235	0.000000
5	1	0	-1.475990	0.708348	-0.000000

5_(P_V)_method_E_DCM_smd.log

Input orientation:

Center Number	Atomic Number	Atomic Type	Coordinates (Angstroms) X	Y	Z
1	8	0	0.910147	1.186705	0.000000
2	9	0	-0.173135	-0.762155	1.184093
3	9	0	-0.173135	-0.762155	-1.184093
4	15	0	-0.178551	0.234918	0.000000
5	1	0	-1.479140	0.711474	0.000000

6_(P_III)_method_A.log

Input orientation:

Center Number	Atomic Number	Atomic Type	Coordinates (Angstroms) X	Y	Z
1	15	0	0.042734	0.830596	-0.684313
2	8	0	-0.109487	1.950154	0.529314
3	1	0	-0.113099	2.853668	0.184686
4	6	0	1.461368	-0.186094	0.096891
5	6	0	-1.408414	-0.298260	-0.159354
6	9	0	1.622373	-1.345832	-0.580679

7	9	0	1.326463	-0.479490	1.401036
8	9	0	2.602858	0.534052	-0.040481
9	9	0	-1.355952	-1.462308	-0.847245
10	9	0	-1.483998	-0.589870	1.149798
11	9	0	-2.560216	0.331484	-0.501477

6_(P_III)_method_A_DCM.log

Input orientation:

Center	Atomic	Atomic	Coordinates (Angstroms)		
Number	Number	Type	X	Y	Z
1	15	0	0.000044	0.771498	-0.759284
2	8	0	0.000010	1.981019	0.368163
3	1	0	0.000515	2.857759	-0.045785
4	6	0	1.434465	-0.250332	-0.005270
5	6	0	-1.434397	-0.250225	-0.005232
6	9	0	1.477000	-1.485756	-0.558640
7	9	0	1.409569	-0.401163	1.334500
8	9	0	2.598541	0.374963	-0.312909
9	9	0	-1.476832	-1.485683	-0.558624
10	9	0	-1.409522	-0.401064	1.334519
11	9	0	-2.598483	0.374984	-0.312907

6_(P_III)_method_A_DCM_smd.log

Input orientation:

Center	Atomic	Atomic	Coordinates (Angstroms)		
Number	Number	Type	X	Y	Z
1	15	0	0.000044	0.771673	-0.756646
2	8	0	-0.000034	1.979310	0.370987
3	1	0	0.000553	2.856796	-0.050980
4	6	0	1.437094	-0.250433	-0.005455
5	6	0	-1.437026	-0.250377	-0.005491
6	9	0	1.484560	-1.486125	-0.558541
7	9	0	1.415889	-0.402710	1.334989
8	9	0	2.599973	0.378353	-0.313266
9	9	0	-1.484552	-1.485954	-0.558919
10	9	0	-1.415695	-0.403028	1.334891
11	9	0	-2.599896	0.378496	-0.313039

6_(P_III)_method_B.log

Input orientation:

Center	Atomic	Atomic	Coordinates (Angstroms)		
Number	Number	Type	X	Y	Z
1	15	0	0.000014	0.758382	-0.737415
2	8	0	0.000008	1.977311	0.355443
3	1	0	0.000659	2.839921	-0.070095
4	6	0	1.435664	-0.252090	0.004426
5	6	0	-1.435638	-0.251978	0.004497
6	9	0	1.478847	-1.467846	-0.565539
7	9	0	1.411677	-0.413032	1.329203
8	9	0	2.581396	0.388042	-0.302895
9	9	0	-1.478574	-1.467833	-0.565383
10	9	0	-1.411725	-0.412803	1.329270
11	9	0	-2.581417	0.387926	-0.302982

6_(P_III)_method_B_DCM.log

Input orientation:

Center	Atomic	Atomic	Coordinates (Angstroms)		
Number	Number	Type	X	Y	Z
1	15	0	0.000059	0.775343	-0.745740
2	8	0	0.000025	1.973524	0.360669
3	1	0	0.000453	2.846161	-0.049569
4	6	0	1.429003	-0.249702	-0.002754
5	6	0	-1.428917	-0.249604	-0.002731
6	9	0	1.463481	-1.474570	-0.556866
7	9	0	1.408249	-0.401240	1.327927
8	9	0	2.585082	0.370830	-0.311751
9	9	0	-1.463347	-1.474502	-0.556833
10	9	0	-1.408183	-0.401122	1.327943
11	9	0	-2.584993	0.370882	-0.311765

6_(P_III)_method_B_DCM_smd.log

Input orientation:

Center	Atomic	Atomic	Coordinates (Angstroms)		
Number	Number	Type	X	Y	Z
1	15	0	0.000129	0.775577	-0.743299
2	8	0	-0.000140	1.972641	0.362229
3	1	0	0.000289	2.846249	-0.055199
4	6	0	1.430820	-0.249766	-0.002265
5	6	0	-1.430651	-0.249911	-0.002683
6	9	0	1.469037	-1.474654	-0.556366
7	9	0	1.413237	-0.402732	1.328966
8	9	0	2.585936	0.373266	-0.311925
9	9	0	-1.469875	-1.473981	-0.558565
10	9	0	-1.412260	-0.404887	1.328304
11	9	0	-2.585611	0.374198	-0.310667

6_(P_III)_method_B_DMSO.log

Input orientation:

Center	Atomic	Atomic	Coordinates (Angstroms)		
Number	Number	Type	X	Y	Z
1	15	0	0.000057	0.778351	-0.747517
2	8	0	0.000019	1.972542	0.362242
3	1	0	0.000462	2.846736	-0.045631
4	6	0	1.428054	-0.249144	-0.004358
5	6	0	-1.427968	-0.249049	-0.004334
6	9	0	1.460778	-1.475817	-0.555077
7	9	0	1.407800	-0.398725	1.327697
8	9	0	2.585797	0.367708	-0.313575
9	9	0	-1.460645	-1.475751	-0.555042
10	9	0	-1.407733	-0.398607	1.327713
11	9	0	-2.585710	0.367756	-0.313588

6_(P_III)_method_B_DMSO_smd.log

Input orientation:

Center Number	Atomic Number	Atomic Type	Coordinates (Angstroms) X	Y	Z
1	15	0	0.000050	0.778951	-0.746100
2	8	0	0.000022	1.972207	0.366526
3	1	0	0.000488	2.845457	-0.051424
4	6	0	1.429233	-0.249409	-0.004841
5	6	0	-1.429155	-0.249314	-0.004807
6	9	0	1.466240	-1.475806	-0.555323
7	9	0	1.410549	-0.400109	1.327690
8	9	0	2.586463	0.369858	-0.312815
9	9	0	-1.466083	-1.475755	-0.555246
10	9	0	-1.410507	-0.399957	1.327721
11	9	0	-2.586389	0.369878	-0.312852

6_(P_III)_method_B_MeOH.log

Input orientation:

Center Number	Atomic Number	Atomic Type	Coordinates (Angstroms) X	Y	Z
1	15	0	0.000057	0.778014	-0.747321
2	8	0	0.000020	1.972655	0.362050
3	1	0	0.000460	2.846675	-0.046093
4	6	0	1.428151	-0.249207	-0.004173
5	6	0	-1.428066	-0.249112	-0.004149
6	9	0	1.461088	-1.475674	-0.555286
7	9	0	1.407829	-0.399022	1.327723
8	9	0	2.585712	0.368067	-0.313348
9	9	0	-1.460956	-1.475607	-0.555252
10	9	0	-1.407762	-0.398905	1.327739
11	9	0	-2.585624	0.368116	-0.313360

6_(P_III)_method_B_MeOH_smd.log

Input orientation:

Center Number	Atomic Number	Atomic Type	Coordinates (Angstroms) X	Y	Z
1	15	0	0.000063	0.780661	-0.744792
2	8	0	0.000032	1.970619	0.361559
3	1	0	0.000424	2.846797	-0.048309
4	6	0	1.429192	-0.248893	-0.004800
5	6	0	-1.429099	-0.248810	-0.004799
6	9	0	1.467647	-1.476457	-0.554831
7	9	0	1.413136	-0.400467	1.328401
8	9	0	2.587204	0.369642	-0.313703
9	9	0	-1.467571	-1.476348	-0.554939
10	9	0	-1.413020	-0.400498	1.328379
11	9	0	-2.587096	0.369755	-0.313637

6_(P_III)_method_B_THF.log

Input orientation:

Center Number	Atomic Number	Atomic Type	Coordinates (Angstroms) X	Y	Z
1	15	0	0.000059	0.774670	-0.745346
2	8	0	0.000025	1.973733	0.360354
3	1	0	0.000453	2.846020	-0.050407
4	6	0	1.429229	-0.249824	-0.002411
5	6	0	-1.429144	-0.249725	-0.002388
6	9	0	1.464061	-1.474299	-0.557246
7	9	0	1.408379	-0.401768	1.327980
8	9	0	2.584928	0.371511	-0.311386
9	9	0	-1.463925	-1.474230	-0.557213
10	9	0	-1.408314	-0.401649	1.327995
11	9	0	-2.584840	0.371561	-0.311401

6_(P_III)_method_B_THF_smd.log

Input orientation:

Center Number	Atomic Number	Atomic Type	Coordinates (Angstroms) X	Y	Z
1	15	0	0.000048	0.775379	-0.743402
2	8	0	0.000021	1.972981	0.365357
3	1	0	0.000499	2.845062	-0.052229
4	6	0	1.430495	-0.249918	-0.002974
5	6	0	-1.430422	-0.249821	-0.002937
6	9	0	1.466250	-1.474867	-0.556406
7	9	0	1.413105	-0.400995	1.327906
8	9	0	2.585815	0.371911	-0.314181
9	9	0	-1.466082	-1.474819	-0.556320
10	9	0	-1.413071	-0.400833	1.327941
11	9	0	-2.585747	0.371920	-0.314223

6_(P_III)_method_B_toluene.log

Input orientation:

Center Number	Atomic Number	Atomic Type	Coordinates (Angstroms) X	Y	Z
1	15	0	0.000052	0.767780	-0.741728
2	8	0	0.000024	1.975604	0.357596
3	1	0	0.000488	2.844219	-0.058591
4	6	0	1.431683	-0.250979	0.000755
5	6	0	-1.431608	-0.250877	0.000788
6	9	0	1.469918	-1.471620	-0.560906
7	9	0	1.409672	-0.406777	1.328517
8	9	0	2.583382	0.378408	-0.307790
9	9	0	-1.469751	-1.471570	-0.560822
10	9	0	-1.409635	-0.406607	1.328548
11	9	0	-2.583314	0.378420	-0.307837

6_(P_III)_method_B_toluene_smd.log

Input orientation:

Center Number	Atomic Number	Atomic Type	Coordinates (Angstroms) X	Y	Z

1	15	0	0.000042	0.768732	-0.740036
2	8	0	0.000019	1.976139	0.360645
3	1	0	0.000531	2.844382	-0.059589
4	6	0	1.431718	-0.250910	0.000684
5	6	0	-1.431653	-0.250807	0.000732
6	9	0	1.468509	-1.471808	-0.560474
7	9	0	1.412434	-0.406439	1.328553
8	9	0	2.583682	0.377367	-0.310093
9	9	0	-1.468305	-1.471782	-0.560322
10	9	0	-1.412431	-0.406207	1.328606
11	9	0	-2.583634	0.377334	-0.310177

6_(P_III)_method_B_water.log

Input orientation:

Center Number	Atomic Number	Atomic Type	Coordinates (Angstroms) X	Y	Z
1	15	0	0.000057	0.778670	-0.747699
2	8	0	0.000018	1.972435	0.362429
3	1	0	0.000465	2.846793	-0.045190
4	6	0	1.427964	-0.249085	-0.004535
5	6	0	-1.427880	-0.248990	-0.004510
6	9	0	1.460483	-1.475953	-0.554877
7	9	0	1.407781	-0.398439	1.327672
8	9	0	2.585880	0.367365	-0.313797
9	9	0	-1.460350	-1.475888	-0.554841
10	9	0	-1.407715	-0.398322	1.327688
11	9	0	-2.585793	0.367413	-0.313810

6_(P_III)_method_B_water_smd.log

Input orientation:

Center Number	Atomic Number	Atomic Type	Coordinates (Angstroms) X	Y	Z
1	15	0	0.000061	0.785271	-0.746943
2	8	0	0.000034	1.971785	0.364073
3	1	0	0.000428	2.847891	-0.045448
4	6	0	1.425708	-0.248453	-0.006318
5	6	0	-1.425618	-0.248366	-0.006309
6	9	0	1.459410	-1.477267	-0.553472
7	9	0	1.406956	-0.397647	1.327300
8	9	0	2.586848	0.363747	-0.314076
9	9	0	-1.459318	-1.477169	-0.553537
10	9	0	-1.406855	-0.397636	1.327291
11	9	0	-2.586744	0.363844	-0.314031

6_(P_III)_method_C_DCM.log

Input orientation:

Center Number	Atomic Number	Atomic Type	Coordinates (Angstroms) X	Y	Z
1	15	0	0.000062	0.777856	-0.749956
2	8	0	0.000025	1.980970	0.367127
3	1	0	0.000433	2.852011	-0.048112
4	6	0	1.435006	-0.251071	-0.004090
5	6	0	-1.434913	-0.250977	-0.004071
6	9	0	1.468922	-1.479355	-0.556667
7	9	0	1.413539	-0.400378	1.329715
8	9	0	2.594353	0.368211	-0.314251
9	9	0	-1.468800	-1.479286	-0.556629
10	9	0	-1.413464	-0.400257	1.329731
11	9	0	-2.594253	0.368276	-0.314267

6_(P_III)_method_C_DCM_smd.log

Input orientation:

Center Number	Atomic Number	Atomic Type	Coordinates (Angstroms) X	Y	Z
1	15	0	-0.000004	0.774989	-0.747711
2	8	0	-0.000062	1.978086	0.367622
3	1	0	0.000238	2.849672	-0.056151
4	6	0	1.436997	-0.252733	-0.002187
5	6	0	-1.436987	-0.252727	-0.002175
6	9	0	1.474283	-1.482702	-0.551376
7	9	0	1.419052	-0.399767	1.332583
8	9	0	2.595383	0.368214	-0.314745
9	9	0	-1.474143	-1.482757	-0.551250
10	9	0	-1.419106	-0.399631	1.332607
11	9	0	-2.595398	0.368096	-0.314860

6_(P_III)_method_D_DCM.log

Input orientation:

Center Number	Atomic Number	Atomic Type	Coordinates (Angstroms) X	Y	Z
1	15	0	0.000007	0.773424	-0.760613
2	8	0	-0.000058	1.977766	0.371567
3	1	0	0.000258	2.856185	-0.038065
4	6	0	1.427928	-0.249985	-0.005305
5	6	0	-1.427906	-0.249965	-0.005301
6	9	0	1.460027	-1.490278	-0.547212
7	9	0	1.403771	-0.387599	1.335590
8	9	0	2.595783	0.364104	-0.319724
9	9	0	-1.459872	-1.490315	-0.547117
10	9	0	-1.403817	-0.387471	1.335600
11	9	0	-2.595783	0.364001	-0.319842

6_(P_III)_method_E_DCM.log

Input orientation:

Center Number	Atomic Number	Atomic Type	Coordinates (Angstroms) X	Y	Z
1	15	0	0.000051	0.767372	-0.737637
2	8	0	0.000026	1.961824	0.355749
3	1	0	0.000491	2.828750	-0.056410
4	6	0	1.422193	-0.247578	-0.001317
5	6	0	-1.422119	-0.247480	-0.001282
6	9	0	1.460167	-1.462862	-0.556155
7	9	0	1.399286	-0.401542	1.320638
8	9	0	2.568174	0.375846	-0.304809

9	9	0	-1.459995	-1.462818	-0.556058
10	9	0	-1.399255	-0.401363	1.320673
11	9	0	-2.568109	0.375852	-0.304863

6_(P_III)_method_E_DCM_smd.log

Input orientation:

Center Number	Atomic Number	Atomic Type	Coordinates (Angstroms) X	Y	Z
1	15	0	0.000007	0.766426	-0.735479
2	8	0	-0.000047	1.957873	0.359456
3	1	0	0.000258	2.826566	-0.058109
4	6	0	1.422202	-0.250615	-0.001781
5	6	0	-1.422186	-0.250600	-0.001781
6	9	0	1.462707	-1.465822	-0.556806
7	9	0	1.401225	-0.406242	1.320741
8	9	0	2.568664	0.373504	-0.304267
9	9	0	-1.462549	-1.465877	-0.556674
10	9	0	-1.401295	-0.406073	1.320757
11	9	0	-2.568669	0.373390	-0.304426

6_(P_V)_method_A.log

Input orientation:

Center Number	Atomic Number	Atomic Type	Coordinates (Angstroms) X	Y	Z
1	15	0	0.000654	0.857329	-0.384016
2	1	0	0.021288	0.805765	-1.796360
3	8	0	-0.013473	2.184310	0.284168
4	6	0	-1.496039	-0.259738	-0.013929
5	6	0	1.484702	-0.264098	0.035960
6	9	0	2.615689	0.459542	-0.038428
7	9	0	1.578184	-1.282456	-0.847088
8	9	0	1.386651	-0.781560	1.272140
9	9	0	-1.350020	-1.472300	-0.591744
10	9	0	-1.675587	-0.432348	1.302845
11	9	0	-2.601938	0.316597	-0.526810

6_(P_V)_method_A_DCM.log

Input orientation:

Center Number	Atomic Number	Atomic Type	Coordinates (Angstroms) X	Y	Z
1	15	0	-0.001934	0.857787	-0.409008
2	1	0	0.000761	0.838196	-1.816988
3	8	0	-0.010133	2.174549	0.291908
4	6	0	-1.489531	-0.259797	0.003613
5	6	0	1.490786	-0.254141	0.008338
6	9	0	2.620886	0.467077	-0.110937
7	9	0	1.568644	-1.304557	-0.834060
8	9	0	1.419455	-0.724646	1.267333
9	9	0	-1.357185	-1.481776	-0.552096
10	9	0	-1.633155	-0.409656	1.331149
11	9	0	-2.612443	0.304258	-0.483329

6_(P_V)_method_A_DCM_smd.log

Input orientation:

Center Number	Atomic Number	Atomic Type	Coordinates (Angstroms) X	Y	Z
1	15	0	-0.002108	0.852067	-0.409629
2	1	0	0.010089	0.829344	-1.818033
3	8	0	-0.020643	2.164799	0.300348
4	6	0	-1.494627	-0.262433	-0.007654
5	6	0	1.496009	-0.249620	0.018328
6	9	0	2.624201	0.469246	-0.147519
7	9	0	1.564145	-1.328376	-0.789473
8	9	0	1.459711	-0.680589	1.293320
9	9	0	-1.397281	-1.469212	-0.602698
10	9	0	-1.626525	-0.453540	1.317378
11	9	0	-2.616821	0.335609	-0.458445

6_(P_V)_method_B.log

Input orientation:

Center Number	Atomic Number	Atomic Type	Coordinates (Angstroms) X	Y	Z
1	15	0	-0.002280	0.855712	-0.382845
2	1	0	0.020861	0.800948	-1.790901
3	8	0	-0.025024	2.163439	0.276956
4	6	0	-1.486755	-0.263749	-0.013222
5	6	0	1.486961	-0.242379	0.033893
6	9	0	2.608299	0.450723	-0.191133
7	9	0	1.507632	-1.336142	-0.742784
8	9	0	1.473396	-0.628220	1.310683
9	9	0	-1.386657	-1.430906	-0.666999
10	9	0	-1.599902	-0.514644	1.290606
11	9	0	-2.600383	0.352512	-0.428331

6_(P_V)_method_B_DCM.log

Input orientation:

Center Number	Atomic Number	Atomic Type	Coordinates (Angstroms) X	Y	Z
1	15	0	-0.002142	0.852035	-0.401021
2	1	0	0.028685	0.836594	-1.804920
3	8	0	-0.030702	2.145736	0.297227
4	6	0	-1.485615	-0.269645	-0.025722
5	6	0	1.485360	-0.242164	0.034057
6	9	0	2.610570	0.438507	-0.211965
7	9	0	1.499879	-1.360256	-0.703086
8	9	0	1.474601	-0.585193	1.325287
9	9	0	-1.403467	-1.431385	-0.686620
10	9	0	-1.574617	-0.530653	1.281240
11	9	0	-2.606404	0.353719	-0.408552

6_(P_V)_method_B_DMSO.log

Input orientation:

Center Number	Atomic Number	Atomic Type	Coordinates (Angstroms) X	Y	Z
1	15	0	-0.002115	0.850549	-0.404522
2	1	0	0.027575	0.847070	-1.807446
3	8	0	-0.030475	2.139012	0.305713
4	6	0	-1.485738	-0.270370	-0.027354
5	6	0	1.485702	-0.243396	0.030849
6	9	0	2.610989	0.438237	-0.212928
7	9	0	1.503423	-1.362963	-0.702346
8	9	0	1.473161	-0.583200	1.323950
9	9	0	-1.404076	-1.436574	-0.678743
10	9	0	-1.575728	-0.521894	1.282422
11	9	0	-2.606569	0.350822	-0.413669

6_(P_V)_method_B_DMSO_smd.log

Input orientation:

Center Number	Atomic Number	Atomic Type	Coordinates (Angstroms) X	Y	Z
1	15	0	-0.003657	0.848165	-0.385807
2	1	0	0.026833	0.860909	-1.789295
3	8	0	-0.033656	2.119706	0.355922
4	6	0	-1.493079	-0.273850	-0.036817
5	6	0	1.493327	-0.240695	0.029069
6	9	0	2.609689	0.426271	-0.295544
7	9	0	1.480859	-1.391565	-0.653947
8	9	0	1.548041	-0.525508	1.335583
9	9	0	-1.425211	-1.422931	-0.719590
10	9	0	-1.598489	-0.562333	1.265776
11	9	0	-2.608508	0.369124	-0.409428

6_(P_V)_method_B_MeOH.log

Input orientation:

Center Number	Atomic Number	Atomic Type	Coordinates (Angstroms) X	Y	Z
1	15	0	-0.002117	0.850730	-0.404146
2	1	0	0.027754	0.845827	-1.807186
3	8	0	-0.030530	2.139832	0.304664
4	6	0	-1.485711	-0.270305	-0.027187
5	6	0	1.485649	-0.243224	0.031268
6	9	0	2.610952	0.438209	-0.212879
7	9	0	1.502926	-1.362704	-0.702272
8	9	0	1.473325	-0.583264	1.324180
9	9	0	-1.404086	-1.435893	-0.679880
10	9	0	-1.575444	-0.523149	1.282228
11	9	0	-2.606568	0.351234	-0.412865

6_(P_V)_method_B_MeOH_smd.log

Input orientation:

Center Number	Atomic Number	Atomic Type	Coordinates (Angstroms) X	Y	Z
1	15	0	-0.002018	0.839295	-0.389993
2	1	0	0.034202	0.861782	-1.792500
3	8	0	-0.035239	2.114216	0.352037
4	6	0	-1.497710	-0.272717	-0.039902
5	6	0	1.497014	-0.241078	0.036485
6	9	0	2.611018	0.424920	-0.294447
7	9	0	1.482356	-1.395430	-0.640721
8	9	0	1.551350	-0.517718	1.343375
9	9	0	-1.425550	-1.426331	-0.714070
10	9	0	-1.612240	-0.550302	1.262991
11	9	0	-2.607032	0.370657	-0.427330

6_(P_V)_method_B_THF.log

Input orientation:

Center Number	Atomic Number	Atomic Type	Coordinates (Angstroms) X	Y	Z
1	15	0	-0.002143	0.852335	-0.400277
2	1	0	0.028777	0.834322	-1.804377
3	8	0	-0.030684	2.147116	0.295433
4	6	0	-1.485581	-0.269394	-0.025227
5	6	0	1.485295	-0.241988	0.034566
6	9	0	2.610471	0.438766	-0.211244
7	9	0	1.499513	-1.359389	-0.703979
8	9	0	1.474613	-0.586407	1.325211
9	9	0	-1.403100	-1.430542	-0.687479
10	9	0	-1.574684	-0.531697	1.281236
11	9	0	-2.606327	0.354172	-0.407940

6_(P_V)_method_B_THF_smd.log

Input orientation:

Center Number	Atomic Number	Atomic Type	Coordinates (Angstroms) X	Y	Z
1	15	0	-0.002685	0.849615	-0.386247
2	1	0	0.032942	0.842038	-1.790729
3	8	0	-0.035801	2.133453	0.330950
4	6	0	-1.491342	-0.272585	-0.036879
5	6	0	1.490985	-0.239093	0.039019
6	9	0	2.608860	0.427217	-0.279230
7	9	0	1.478146	-1.387310	-0.651141
8	9	0	1.537227	-0.529879	1.342748
9	9	0	-1.416859	-1.422488	-0.720100
10	9	0	-1.599154	-0.560460	1.263831
11	9	0	-2.606168	0.366787	-0.416298

6_(P_V)_method_B_toluene.log

Input orientation:

Center Number	Atomic Number	Atomic Type	Coordinates (Angstroms) X	Y	Z
1	15	0	-0.002222	0.854370	-0.392229
2	1	0	0.028384	0.815938	-1.798148
3	8	0	-0.030007	2.157103	0.283290

Center Number	Atomic Number	Atomic Type	X	Y	Z
4	6	0	-1.485939	-0.267430	-0.021589
5	6	0	1.485486	-0.240809	0.038225
6	9	0	2.609403	0.441149	-0.208995
7	9	0	1.496443	-1.353335	-0.710140
8	9	0	1.478414	-0.594658	1.324833
9	9	0	-1.400052	-1.424025	-0.694003
10	9	0	-1.578883	-0.539260	1.280972
11	9	0	-2.604876	0.358251	-0.406292

6_(P_V)_method_B_toluene_smd.log

Input orientation:

Center Number	Atomic Number	Atomic Type	Coordinates (Angstroms) X	Y	Z
1	15	0	-0.002144	0.854408	-0.392255
2	1	0	0.015145	0.810568	-1.798662
3	8	0	-0.023175	2.156604	0.284958
4	6	0	-1.485596	-0.261228	-0.007852
5	6	0	1.486641	-0.245643	0.025036
6	9	0	2.608449	0.458849	-0.163640
7	9	0	1.529038	-1.321529	-0.774596
8	9	0	1.459033	-0.662295	1.293193
9	9	0	-1.379079	-1.447369	-0.623864
10	9	0	-1.612734	-0.473470	1.302735
11	9	0	-2.599427	0.338398	-0.449129

6_(P_V)_method_B_water.log

Input orientation:

Center Number	Atomic Number	Atomic Type	Coordinates (Angstroms) X	Y	Z
1	15	0	-0.002129	0.850343	-0.404769
2	1	0	0.027586	0.848465	-1.807581
3	8	0	-0.030473	2.138046	0.307068
4	6	0	-1.485850	-0.270519	-0.027819
5	6	0	1.485775	-0.243507	0.030661
6	9	0	2.610960	0.437936	-0.214228
7	9	0	1.503352	-1.363973	-0.700968
8	9	0	1.473812	-0.581649	1.324329
9	9	0	-1.404481	-1.436824	-0.678921
10	9	0	-1.575703	-0.521784	1.282105
11	9	0	-2.606698	0.350762	-0.413952

6_(P_V)_method_B_water_smd.log

Input orientation:

Center Number	Atomic Number	Atomic Type	Coordinates (Angstroms) X	Y	Z
1	15	0	0.003323	0.844284	-0.383716
2	1	0	0.027369	0.880234	-1.785209
3	8	0	-0.011217	2.110997	0.374315
4	6	0	-1.497402	-0.259441	-0.030419
5	6	0	1.493468	-0.256277	0.020413
6	9	0	2.613160	0.400493	-0.308228
7	9	0	1.461421	-1.401988	-0.669927
8	9	0	1.551364	-0.549065	1.323861
9	9	0	-1.440141	-1.405071	-0.719036
10	9	0	-1.598615	-0.552233	1.270440
11	9	0	-2.606580	0.395362	-0.396569

6_(P_V)_method_C_DCM.log

Input orientation:

Center Number	Atomic Number	Atomic Type	Coordinates (Angstroms) X	Y	Z
1	15	0	-0.001461	0.852821	-0.403570
2	1	0	0.030340	0.836829	-1.811458
3	8	0	-0.029747	2.157902	0.298894
4	6	0	-1.494584	-0.270748	-0.026038
5	6	0	1.493758	-0.245284	0.035523
6	9	0	2.620070	0.437547	-0.212916
7	9	0	1.509424	-1.366807	-0.700820
8	9	0	1.484115	-0.586023	1.329808
9	9	0	-1.417622	-1.433785	-0.690165
10	9	0	-1.582214	-0.533468	1.283183
11	9	0	-2.615929	0.358312	-0.406517

6_(P_V)_method_C_DCM_smd.log

Input orientation:

Center Number	Atomic Number	Atomic Type	Coordinates (Angstroms) X	Y	Z
1	15	0	-0.000072	0.849137	-0.402768
2	1	0	0.006732	0.823279	-1.811278
3	8	0	-0.006393	2.153641	0.302919
4	6	0	-1.498690	-0.257011	-0.001737
5	6	0	1.499116	-0.251612	0.011787
6	9	0	2.618955	0.403297	-0.333269
7	9	0	1.465390	-1.409429	-0.665811
8	9	0	1.565782	-0.528478	1.320398
9	9	0	-1.445817	-1.423076	-0.663661
10	9	0	-1.585650	-0.517323	1.309075
11	9	0	-2.616967	0.385599	-0.374151

6_(P_V)_method_D_DCM.log

Input orientation:

Center Number	Atomic Number	Atomic Type	Coordinates (Angstroms) X	Y	Z
1	15	0	-0.000995	0.862631	-0.419951
2	1	0	-0.007408	0.832310	-1.827199
3	8	0	-0.002620	2.182598	0.273952
4	6	0	-1.479809	-0.252508	0.013118
5	6	0	1.482260	-0.254042	0.000958
6	9	0	2.617216	0.460870	-0.102516
7	9	0	1.560300	-1.299391	-0.847099
8	9	0	1.394811	-0.731806	1.256448
9	9	0	-1.330811	-1.489999	-0.502454
10	9	0	-1.628359	-0.360628	1.344015
11	9	0	-2.605176	0.283946	-0.497896

6_(P_V)_method_E_DCM.log

Input orientation:

Center	Atomic	Atomic	Coordinates (Angstroms)		
Number	Number	Type	X	Y	Z
1	15	0	-0.002035	0.845808	-0.405939
2	1	0	0.025920	0.820435	-1.807602
3	8	0	-0.029090	2.136442	0.284963
4	6	0	-1.475715	-0.266817	-0.020892
5	6	0	1.476002	-0.241049	0.032879
6	9	0	2.593964	0.444245	-0.186243
7	9	0	1.502861	-1.340358	-0.716978
8	9	0	1.447386	-0.600947	1.310858
9	9	0	-1.396208	-1.422163	-0.676738
10	9	0	-1.556873	-0.522678	1.279103
11	9	0	-2.590062	0.354377	-0.397488

6_(P_V)_method_E_DCM_smd.log

Input orientation:

Center	Atomic	Atomic	Coordinates (Angstroms)		
Number	Number	Type	X	Y	Z
1	15	0	-0.000523	0.845120	-0.419061
2	1	0	-0.001861	0.799318	-1.820914
3	8	0	-0.002784	2.140724	0.264563
4	6	0	-1.475007	-0.249513	0.010120
5	6	0	1.476690	-0.249938	0.007463
6	9	0	2.597036	0.429928	-0.222965
7	9	0	1.495020	-1.351836	-0.739887
8	9	0	1.464593	-0.610308	1.286851
9	9	0	-1.422130	-1.413366	-0.634436
10	9	0	-1.535714	-0.492399	1.314834
11	9	0	-2.593345	0.376866	-0.348937

6_P_V_methodB_DCM_smd.log

Input orientation:

Center	Atomic	Atomic	Coordinates (Angstroms)		
Number	Number	Type	X	Y	Z
1	15	0	0.000003	0.846621	-0.399619
2	1	0	0.008550	0.821537	-1.804186
3	8	0	-0.007288	2.139912	0.301504
4	6	0	-1.492112	-0.255643	-0.003944
5	6	0	1.492312	-0.248876	0.013666
6	9	0	2.609800	0.402019	-0.336922
7	9	0	1.455708	-1.407739	-0.657010
8	9	0	1.561995	-0.519299	1.320901
9	9	0	-1.441918	-1.415053	-0.672673
10	9	0	-1.576628	-0.524895	1.302694
11	9	0	-2.608217	0.389701	-0.369031

7_(P_III)_method_A.log

Input orientation:

Center	Atomic	Atomic	Coordinates (Angstroms)		
Number	Number	Type	X	Y	Z
1	15	0	-0.069932	0.683431	-0.787702
2	8	0	-0.136711	2.039023	0.196380
3	1	0	0.201642	2.820915	-0.260114
4	6	0	1.393326	-0.202812	0.061948
5	6	0	-1.415562	-0.281255	0.022896
6	9	0	1.538182	-1.448549	-0.469516
7	9	0	1.287001	-0.353755	1.403189
8	9	0	2.549740	0.470747	-0.166752
9	1	0	-1.344829	-0.225511	1.112526
10	1	0	-1.362109	-1.326192	-0.298315
11	1	0	-2.375012	0.131594	-0.300254

7_(P_III)_method_A_DCM.log

Input orientation:

Center	Atomic	Atomic	Coordinates (Angstroms)		
Number	Number	Type	X	Y	Z
1	15	0	-0.073231	0.688046	-0.782812
2	8	0	-0.113689	2.038003	0.208666
3	1	0	0.173632	2.829762	-0.268904
4	6	0	1.393154	-0.202533	0.061427
5	6	0	-1.416793	-0.281655	0.020612
6	9	0	1.533633	-1.458290	-0.449107
7	9	0	1.302362	-0.336542	1.409895
8	9	0	2.558942	0.455935	-0.179888
9	1	0	-1.348368	-0.245095	1.111195
10	1	0	-1.370606	-1.321088	-0.318599
11	1	0	-2.373300	0.141092	-0.298199

7_(P_III)_method_A_DCM_smd.log

Input orientation:

Center	Atomic	Atomic	Coordinates (Angstroms)		
Number	Number	Type	X	Y	Z
1	15	0	-0.081599	0.682330	-0.778829
2	8	0	-0.115937	2.038144	0.203406
3	1	0	0.197416	2.821941	-0.277450
4	6	0	1.392326	-0.201279	0.060758
5	6	0	-1.422679	-0.281961	0.025478
6	9	0	1.552001	-1.453044	-0.454662
7	9	0	1.308230	-0.344019	1.409606
8	9	0	2.552686	0.472067	-0.176646
9	1	0	-1.360092	-0.249886	1.117348
10	1	0	-1.378624	-1.321026	-0.318355
11	1	0	-2.377993	0.144370	-0.296369

7_(P_III)_method_B.log

Input orientation:

Center Atomic Atomic Coordinates (Angstroms)

Number	Number	Type	X	Y	Z
1	15	0	0.918717	-0.126849	-0.642556
2	8	0	1.458422	-1.295952	0.395403
3	1	0	1.572673	-2.138079	-0.054045
4	6	0	-0.866395	0.017394	0.001505
5	6	0	1.584968	1.336255	0.230632
6	9	0	-1.479890	1.061092	-0.600969
7	9	0	-0.988327	0.197595	1.329030
8	9	0	-1.563891	-1.094219	-0.307891
9	1	0	1.420734	1.269562	1.304824
10	1	0	1.108405	2.235408	-0.159805
11	1	0	2.653085	1.397630	0.028222

7_(P_III)_method_B_DCM.log

Input orientation:

Center Number	Atomic Number	Atomic Type	Coordinates (Angstroms) X	Y	Z
1	15	0	0.925708	-0.126062	-0.636056
2	8	0	1.442553	-1.306472	0.396430
3	1	0	1.594436	-2.136944	-0.066817
4	6	0	-0.862155	0.020365	0.005264
5	6	0	1.593703	1.334069	0.235735
6	9	0	-1.479442	1.073444	-0.578136
7	9	0	-0.989642	0.180449	1.338434
8	9	0	-1.571566	-1.083492	-0.316746
9	1	0	1.426133	1.277811	1.310011
10	1	0	1.128203	2.234884	-0.163967
11	1	0	2.662773	1.384438	0.035436

7_(P_III)_method_B_DCM_smd.log

Input orientation:

Center Number	Atomic Number	Atomic Type	Coordinates (Angstroms) X	Y	Z
1	15	0	0.929060	-0.117838	-0.631753
2	8	0	1.444206	-1.304902	0.392829
3	1	0	1.573482	-2.139602	-0.076176
4	6	0	-0.861410	0.019173	0.005413
5	6	0	1.597841	1.336940	0.239412
6	9	0	-1.493061	1.061388	-0.582348
7	9	0	-0.996949	0.182264	1.338227
8	9	0	-1.560654	-1.094599	-0.312545
9	1	0	1.431472	1.289653	1.315097
10	1	0	1.138359	2.239104	-0.166935
11	1	0	2.668358	1.380909	0.038367

7_(P_III)_method_C_DCM.log

Input orientation:

Center Number	Atomic Number	Atomic Type	Coordinates (Angstroms) X	Y	Z
1	15	0	-0.076170	0.691478	-0.773852
2	8	0	-0.113923	2.033810	0.205566
3	1	0	0.172401	2.819132	-0.273904
4	6	0	1.391161	-0.204431	0.063785
5	6	0	-1.413707	-0.278523	0.022335
6	9	0	1.528805	-1.451724	-0.448539
7	9	0	1.301028	-0.339244	1.405598
8	9	0	2.549299	0.453607	-0.177853
9	1	0	-1.344899	-0.245979	1.108448
10	1	0	-1.361489	-1.311548	-0.321152
11	1	0	-2.366771	0.141056	-0.296145

7_(P_III)_method_C_DCM_smd.log

Input orientation:

Center Number	Atomic Number	Atomic Type	Coordinates (Angstroms) X	Y	Z
1	15	0	0.928743	-0.118199	-0.637805
2	8	0	1.451585	-1.306276	0.399208
3	1	0	1.576538	-2.141824	-0.070643
4	6	0	-0.869093	0.015924	0.001537
5	6	0	1.596845	1.344693	0.235095
6	9	0	-1.504950	1.057202	-0.589114
7	9	0	-1.005101	0.182044	1.336723
8	9	0	-1.567714	-1.101940	-0.314371
9	1	0	1.431051	1.295536	1.310827
10	1	0	1.133342	2.244985	-0.170820
11	1	0	2.667132	1.391231	0.032980

7_(P_III)_method_D_DCM.log

Input orientation:

Center Number	Atomic Number	Atomic Type	Coordinates (Angstroms) X	Y	Z
1	15	0	0.926576	-0.127634	-0.653079
2	8	0	1.448388	-1.314011	0.407779
3	1	0	1.594219	-2.152849	-0.052835
4	6	0	-0.860909	0.018902	-0.000168
5	6	0	1.594618	1.340127	0.231835
6	9	0	-1.482904	1.086528	-0.574603
7	9	0	-0.980790	0.172397	1.343697
8	9	0	-1.585130	-1.085198	-0.325897
9	1	0	1.426756	1.271306	1.309784
10	1	0	1.122213	2.246192	-0.159302
11	1	0	2.667666	1.396731	0.032377

7_(P_III)_method_E_DCM.log

Input orientation:

Center Number	Atomic Number	Atomic Type	Coordinates (Angstroms) X	Y	Z
1	15	0	-0.072953	0.693726	-0.762256
2	8	0	-0.106693	2.017828	0.202268
3	1	0	0.171623	2.803182	-0.271988
4	6	0	1.375182	-0.199143	0.065368
5	6	0	-1.396035	-0.274811	0.019893
6	9	0	1.501024	-1.438001	-0.440804

7	9	0	1.283142	-0.327493	1.395933
8	9	0	2.526278	0.447451	-0.177358
9	1	0	-1.329398	-0.242201	1.106192
10	1	0	-1.333655	-1.307485	-0.323175
11	1	0	-2.352781	0.134584	-0.299785

7_(P_III)_method_E_DCM_smd.log

Input orientation:

Center	Atomic	Atomic	Coordinates (Angstroms)		
Number	Number	Type	X	Y	Z
1	15	0	0.919465	-0.120483	-0.628425
2	8	0	1.434822	-1.292005	0.393080
3	1	0	1.568718	-2.124238	-0.070039
4	6	0	-0.859790	0.014101	0.001719
5	6	0	1.572733	1.336768	0.229523
6	9	0	-1.485042	1.051755	-0.580783
7	9	0	-0.990711	0.174075	1.326311
8	9	0	-1.553206	-1.092440	-0.316447
9	1	0	1.406238	1.290001	1.305283
10	1	0	1.101093	2.231545	-0.179410
11	1	0	2.642434	1.394598	0.028740

7_(P_V)_method_A.log

Input orientation:

Center	Atomic	Atomic	Coordinates (Angstroms)		
Number	Number	Type	X	Y	Z
1	15	0	0.053739	0.845627	-0.388516
2	1	0	-0.007249	0.822615	-1.803585
3	8	0	-0.006521	2.190341	0.258193
4	6	0	-1.450559	-0.260644	-0.033847
5	9	0	-1.367925	-1.413951	-0.753524
6	9	0	-1.520433	-0.601026	1.270877
7	9	0	-2.603155	0.347290	-0.371588
8	6	0	1.450785	-0.234746	0.047657
9	1	0	1.387017	-1.190759	-0.479764
10	1	0	2.377725	0.275620	-0.228028
11	1	0	1.450814	-0.404798	1.127458

7_(P_V)_method_A_DCM.log

Input orientation:

Center	Atomic	Atomic	Coordinates (Angstroms)		
Number	Number	Type	X	Y	Z
1	15	0	0.063426	0.825299	-0.399822
2	1	0	0.001980	0.832289	-1.809253
3	8	0	-0.034667	2.169115	0.266215
4	6	0	-1.452193	-0.264445	-0.037539
5	9	0	-1.405447	-1.425084	-0.739821
6	9	0	-1.527754	-0.585652	1.274318
7	9	0	-2.599444	0.366171	-0.371007
8	6	0	1.463020	-0.233391	0.048583
9	1	0	1.411531	-1.184974	-0.487731
10	1	0	2.384725	0.286912	-0.225504
11	1	0	1.459061	-0.410670	1.126897

7_(P_V)_method_A_DCM_smd.log

Input orientation:

Center	Atomic	Atomic	Coordinates (Angstroms)		
Number	Number	Type	X	Y	Z
1	15	0	0.068883	0.817820	-0.397787
2	1	0	-0.001598	0.825657	-1.806991
3	8	0	-0.039698	2.159609	0.273754
4	6	0	-1.452940	-0.264606	-0.037119
5	9	0	-1.422585	-1.430229	-0.733048
6	9	0	-1.546198	-0.581214	1.276270
7	9	0	-2.595261	0.376413	-0.379389
8	6	0	1.470516	-0.232191	0.047150
9	1	0	1.425133	-1.182855	-0.493267
10	1	0	2.386876	0.298442	-0.230434
11	1	0	1.471109	-0.411277	1.126197

7_(P_V)_method_B.log

Input orientation:

Center	Atomic	Atomic	Coordinates (Angstroms)		
Number	Number	Type	X	Y	Z
1	15	0	-0.942654	-0.258974	-0.324589
2	1	0	-0.898471	-0.221829	-1.735139
3	8	0	-1.483057	-1.484492	0.284969
4	6	0	0.896637	0.027811	0.013798
5	9	0	1.338763	1.103247	-0.676330
6	9	0	1.126619	0.258306	1.315108
7	9	0	1.631382	-1.025059	-0.359881
8	6	0	-1.688823	1.309139	0.159038
9	1	0	-1.198402	2.142724	-0.341712
10	1	0	-2.742010	1.284230	-0.117138
11	1	0	-1.610230	1.426100	1.238539

7_(P_V)_method_B_DCM.log

Input orientation:

Center	Atomic	Atomic	Coordinates (Angstroms)		
Number	Number	Type	X	Y	Z
1	15	0	-0.946108	-0.232993	-0.333999
2	1	0	-0.912341	-0.219548	-1.740217
3	8	0	-1.457710	-1.474271	0.290083
4	6	0	0.895765	0.032304	0.010706
5	9	0	1.370736	1.096746	-0.666184
6	9	0	1.123541	0.246129	1.317408
7	9	0	1.614941	-1.038415	-0.359023
8	6	0	-1.698231	1.319126	0.161781
9	1	0	-1.218422	2.153175	-0.348336
10	1	0	-2.752141	1.287326	-0.110542
11	1	0	-1.610229	1.440641	1.239834

7_(P_V)_method_B_DCM_smd.log

Input orientation:

Center Atomic Number	Atomic Number	Atomic Type	Coordinates (Angstroms) X	Y	Z
1	15	0	-0.946978	-0.223235	-0.333036
2	1	0	-0.906177	-0.212382	-1.739198
3	8	0	-1.448807	-1.468445	0.294125
4	6	0	0.897362	0.031598	0.010599
5	9	0	1.391224	1.089883	-0.663334
6	9	0	1.137071	0.237737	1.317855
7	9	0	1.605963	-1.048081	-0.363175
8	6	0	-1.705398	1.321184	0.161657
9	1	0	-1.236091	2.159228	-0.353713
10	1	0	-2.760371	1.275749	-0.110596
11	1	0	-1.617995	1.446983	1.240328

7_(P_V)_method_C_DCM.log

Input orientation:

Center Atomic Number	Atomic Number	Atomic Type	Coordinates (Angstroms) X	Y	Z
1	15	0	0.064791	0.819712	-0.395699
2	1	0	0.008706	0.823193	-1.805153
3	8	0	-0.033814	2.154771	0.264578
4	6	0	-1.453173	-0.267256	-0.038390
5	9	0	-1.413986	-1.414951	-0.748555
6	9	0	-1.525228	-0.596085	1.264927
7	9	0	-2.589890	0.373334	-0.361838
8	6	0	1.462125	-0.229438	0.050388
9	1	0	1.404155	-1.181717	-0.474845
10	1	0	2.376492	0.287992	-0.236378
11	1	0	1.464059	-0.393985	1.126299

7_(P_V)_method_C_DCM_smd.log

Input orientation:

Center Atomic Number	Atomic Number	Atomic Type	Coordinates (Angstroms) X	Y	Z
1	15	0	-0.947325	-0.228402	-0.335651
2	1	0	-0.905099	-0.218754	-1.745632
3	8	0	-1.450106	-1.485258	0.296505
4	6	0	0.903909	0.031909	0.010602
5	9	0	1.398613	1.089213	-0.669165
6	9	0	1.142599	0.244927	1.319668
7	9	0	1.614637	-1.051222	-0.357750
8	6	0	-1.715747	1.319968	0.162213
9	1	0	-1.242618	2.160199	-0.346038
10	1	0	-2.768335	1.272623	-0.118827
11	1	0	-1.635494	1.438254	1.242254

7_(P_V)_method_D_DCM.log

Input orientation:

Center Atomic Number	Atomic Number	Atomic Type	Coordinates (Angstroms) X	Y	Z
1	15	0	-0.949256	-0.240383	-0.344869
2	1	0	-0.910939	-0.230847	-1.754601
3	8	0	-1.468020	-1.500020	0.289021
4	6	0	0.894384	0.032172	0.010512
5	9	0	1.371979	1.106288	-0.667895
6	9	0	1.113888	0.251575	1.327009
7	9	0	1.629516	-1.040240	-0.354822
8	6	0	-1.698200	1.325989	0.162785
9	1	0	-1.219568	2.162367	-0.353732
10	1	0	-2.759765	1.298586	-0.095834
11	1	0	-1.594217	1.444731	1.243936

7_(P_V)_method_E_DCM.log

Input orientation:

Center Atomic Number	Atomic Number	Atomic Type	Coordinates (Angstroms) X	Y	Z
1	15	0	0.062140	0.820809	-0.395839
2	1	0	0.004585	0.823243	-1.799213
3	8	0	-0.032094	2.138003	0.259642
4	6	0	-1.432913	-0.262325	-0.035723
5	9	0	-1.376341	-1.406726	-0.728914
6	9	0	-1.500852	-0.575139	1.260554
7	9	0	-2.566118	0.357781	-0.366057
8	6	0	1.439288	-0.227185	0.045606
9	1	0	1.384302	-1.169863	-0.496803
10	1	0	2.360598	0.290340	-0.215790
11	1	0	1.421642	-0.413367	1.117872

7_(P_V)_method_E_DCM_smd.log

Input orientation:

Center Atomic Number	Atomic Number	Atomic Type	Coordinates (Angstroms) X	Y	Z
1	15	0	-0.937936	-0.228722	-0.334434
2	1	0	-0.899406	-0.220058	-1.738517
3	8	0	-1.441188	-1.464179	0.295901
4	6	0	0.894108	0.027103	0.010105
5	9	0	1.378562	1.085709	-0.652533
6	9	0	1.124129	0.226138	1.311744
7	9	0	1.602964	-1.040847	-0.365595
8	6	0	-1.679995	1.314221	0.159560
9	1	0	-1.213937	2.145274	-0.369700
10	1	0	-2.739909	1.272109	-0.091569
11	1	0	-1.568563	1.445013	1.235385

8_(P_III)_method_A.log

Input orientation:

Center Atomic Number	Atomic Number	Atomic Type	Coordinates (Angstroms) X	Y	Z

1	15	0	-0.285044	0.740885	-0.987262
2	8	0	-0.930825	2.242290	-0.643937
3	1	0	-1.268194	2.672471	-1.441268
4	6	0	1.021754	0.889041	0.415243
5	9	0	1.539222	-0.335516	0.692515
6	9	0	0.574932	1.407220	1.583969
7	9	0	2.044626	1.673790	-0.006389
8	6	0	-1.961903	-0.190445	0.977571
9	1	0	-2.269995	0.841392	1.160875
10	1	0	-2.834572	-0.844469	0.956778
11	1	0	-1.269858	-0.519637	1.756835
12	8	0	-1.337897	-0.315152	-0.321149

8_(P_III)_method_A_DCM.log

Input orientation:

Center Number	Atomic Number	Atomic Type	Coordinates (Angstroms) X	Y	Z
1	15	0	-0.283146	0.748974	-0.988724
2	8	0	-0.914776	2.245076	-0.626602
3	1	0	-1.298169	2.669565	-1.408795
4	6	0	1.033144	0.886112	0.410844
5	9	0	1.553625	-0.339063	0.686172
6	9	0	0.598718	1.403385	1.587346
7	9	0	2.062301	1.670373	-0.007046
8	6	0	-1.974896	-0.188763	0.970231
9	1	0	-2.313109	0.837349	1.126369
10	1	0	-2.830039	-0.864349	0.946696
11	1	0	-1.288330	-0.486243	1.766245
12	8	0	-1.323076	-0.320547	-0.318955

8_(P_III)_method_A_DCM_smd.log

Input orientation:

Center Number	Atomic Number	Atomic Type	Coordinates (Angstroms) X	Y	Z
1	15	0	-0.286200	0.750112	-0.988836
2	8	0	-0.907582	2.251160	-0.635013
3	1	0	-1.300268	2.666765	-1.421625
4	6	0	1.029527	0.891436	0.411816
5	9	0	1.543075	-0.332832	0.705993
6	9	0	0.603655	1.430721	1.581839
7	9	0	2.066936	1.661586	-0.016755
8	6	0	-1.972608	-0.195192	0.973741
9	1	0	-2.290505	0.833521	1.156724
10	1	0	-2.844003	-0.850926	0.939841
11	1	0	-1.289984	-0.529560	1.759114
12	8	0	-1.329796	-0.314920	-0.323058

8_(P_III)_method_B.log

Input orientation:

Center Number	Atomic Number	Atomic Type	Coordinates (Angstroms) X	Y	Z
1	15	0	-0.471221	0.777067	-0.692785
2	8	0	-0.683591	1.629098	0.701171
3	1	0	-1.003188	2.519145	0.527583
4	6	0	1.015681	-0.202846	0.016692
5	9	0	1.257902	-1.283923	-0.752889
6	9	0	0.875856	-0.639292	1.282551
7	9	0	2.116943	0.572392	-0.018582
8	6	0	-2.014262	-1.150863	0.470918
9	1	0	-2.028222	-0.555782	1.381354
10	1	0	-3.012734	-1.519898	0.253853
11	1	0	-1.329176	-1.988655	0.585950
12	8	0	-1.622985	-0.346890	-0.658258

8_(P_III)_method_B_DCM.log

Input orientation:

Center Number	Atomic Number	Atomic Type	Coordinates (Angstroms) X	Y	Z
1	15	0	-0.471203	0.775271	-0.693143
2	8	0	-0.667861	1.626605	0.695268
3	1	0	-1.029159	2.504952	0.532187
4	6	0	1.021108	-0.203465	0.014470
5	9	0	1.265253	-1.291299	-0.747294
6	9	0	0.895013	-0.633538	1.285720
7	9	0	2.128277	0.568734	-0.029865
8	6	0	-2.033390	-1.135092	0.485810
9	1	0	-2.077190	-0.513599	1.376679
10	1	0	-3.022630	-1.516765	0.252368
11	1	0	-1.346025	-1.963954	0.640990
12	8	0	-1.615561	-0.356745	-0.657109

8_(P_III)_method_B_DCM_smd.log

Input orientation:

Center Number	Atomic Number	Atomic Type	Coordinates (Angstroms) X	Y	Z
1	15	0	-0.472310	0.776475	-0.690808
2	8	0	-0.661033	1.633991	0.693268
3	1	0	-1.033638	2.510463	0.525832
4	6	0	1.020314	-0.200886	0.019980
5	9	0	1.260668	-1.299604	-0.728024
6	9	0	0.905922	-0.613104	1.298202
7	9	0	2.130342	0.568675	-0.042245
8	6	0	-2.033582	-1.140827	0.482232
9	1	0	-2.058871	-0.535629	1.385603
10	1	0	-3.032889	-1.502917	0.255798
11	1	0	-1.359312	-1.985287	0.612015
12	8	0	-1.618975	-0.350243	-0.655772

8_(P_III)_method_D_DCM.log

Input orientation:

Center Number	Atomic Number	Atomic Type	Coordinates (Angstroms) X	Y	Z
1	15	0	-0.466046	0.785254	-0.709199
2	8	0	-0.667618	1.627673	0.710805

3	1	0	-1.049065	2.504208	0.553711
4	6	0	1.014026	-0.207084	0.012185
5	9	0	1.250860	-1.310582	-0.744959
6	9	0	0.875388	-0.630320	1.293276
7	9	0	2.138641	0.555080	-0.030784
8	6	0	-2.023435	-1.130490	0.482869
9	1	0	-2.066758	-0.490137	1.365697
10	1	0	-3.012773	-1.529797	0.261038
11	1	0	-1.318469	-1.949053	0.643616
12	8	0	-1.628117	-0.363646	-0.682174

8_(P_V)_method_A.log

Input orientation:

Center Number	Atomic Number	Atomic Type	Coordinates (Angstroms) X	Y	Z
1	15	0	-0.064395	0.131305	0.084179
2	1	0	-0.229989	0.059530	1.478729
3	8	0	-0.056954	1.468109	-0.560545
4	6	0	-1.587765	-1.067869	-1.752785
5	1	0	-0.821688	-1.587943	-2.332808
6	1	0	-2.504379	-1.656228	-1.729422
7	1	0	-1.780745	-0.079981	-2.175802
8	8	0	-1.165063	-0.949100	-0.368199
9	6	0	1.575709	-0.792752	-0.154586
10	9	0	1.580261	-1.959150	0.534627
11	9	0	1.784679	-1.083656	-1.458536
12	9	0	2.616238	-0.055779	0.284325

8_(P_V)_method_A_DCM.log

Input orientation:

Center Number	Atomic Number	Atomic Type	Coordinates (Angstroms) X	Y	Z
1	15	0	-0.064798	0.140474	0.091064
2	1	0	-0.230290	0.095804	1.482601
3	8	0	-0.013070	1.477201	-0.566729
4	6	0	-1.592355	-1.078056	-1.751137
5	1	0	-0.802458	-1.577471	-2.315045
6	1	0	-2.483346	-1.702316	-1.716707
7	1	0	-1.825663	-0.107689	-2.191389
8	8	0	-1.182362	-0.912185	-0.361190
9	6	0	1.568623	-0.797487	-0.157571
10	9	0	1.558887	-1.992395	0.478025
11	9	0	1.800986	-1.032471	-1.469477
12	9	0	2.611754	-0.086921	0.326733

8_(P_V)_method_A_DCM_smd.log

Input orientation:

Center Number	Atomic Number	Atomic Type	Coordinates (Angstroms) X	Y	Z
1	15	0	-0.067884	0.171074	0.088310
2	1	0	-0.202537	0.131991	1.482742
3	8	0	0.006486	1.504395	-0.576180

4	6	0	-1.586362	-1.107992	-1.725866
5	1	0	-0.793247	-1.665271	-2.229581
6	1	0	-2.495017	-1.707400	-1.680380
7	1	0	-1.782195	-0.164683	-2.239087
8	8	0	-1.215923	-0.859760	-0.335507
9	6	0	1.551267	-0.787585	-0.184533
10	9	0	1.488757	-2.046064	0.311950
11	9	0	1.862434	-0.881922	-1.497602
12	9	0	2.580128	-0.160294	0.434912

8_(P_V)_method_B.log

Input orientation:

Center Number	Atomic Number	Atomic Type	Coordinates (Angstroms) X	Y	Z
1	15	0	0.473755	0.716334	-0.411587
2	1	0	0.214631	1.085465	-1.738982
3	8	0	0.788068	1.770111	0.554157
4	6	0	2.326492	-0.993678	0.418379
5	1	0	1.743180	-1.724956	0.973833
6	1	0	3.172930	-1.476397	-0.058973
7	1	0	2.667651	-0.201083	1.079826
8	8	0	1.524613	-0.441094	-0.652293
9	6	0	-1.109716	-0.211816	0.031453
10	9	0	-1.432133	-1.097038	-0.930363
11	9	0	-0.965051	-0.890365	1.182264
12	9	0	-2.140829	0.631662	0.169568

8_(P_V)_method_B_DCM.log

Input orientation:

Center Number	Atomic Number	Atomic Type	Coordinates (Angstroms) X	Y	Z
1	15	0	0.481279	0.719843	-0.433789
2	1	0	0.218070	1.080624	-1.758724
3	8	0	0.769112	1.793970	0.526959
4	6	0	2.325396	-1.011023	0.421319
5	1	0	1.714437	-1.723207	0.969730
6	1	0	3.153322	-1.519043	-0.060016
7	1	0	2.694315	-0.233755	1.084475
8	8	0	1.543235	-0.421728	-0.652099
9	6	0	-1.096775	-0.203787	0.045607
10	9	0	-1.406588	-1.152126	-0.856122
11	9	0	-0.961826	-0.803912	1.240900
12	9	0	-2.139414	0.638701	0.122847

8_(P_V)_method_B_DCM_smd.log

Input orientation:

Center Number	Atomic Number	Atomic Type	Coordinates (Angstroms) X	Y	Z
1	15	0	0.496730	0.747727	-0.426461
2	1	0	0.216382	1.116640	-1.744995
3	8	0	0.765057	1.815830	0.547512
4	6	0	2.301222	-1.038567	0.405737

5	1	0	1.649314	-1.754523	0.901342
6	1	0	3.127216	-1.554685	-0.072558
7	1	0	2.677595	-0.307097	1.116391
8	8	0	1.575947	-0.372913	-0.665153
9	6	0	-1.070698	-0.195162	0.054892
10	9	0	-1.300196	-1.241288	-0.759468
11	9	0	-1.000282	-0.665181	1.311997
12	9	0	-2.143723	0.613776	-0.018151

8_(P_V)_method_D_DCM.log

Input orientation:

Center Number	Atomic Number	Atomic Type	Coordinates (Angstroms) X	Y	Z
1	15	0	0.481993	0.740028	-0.444046
2	1	0	0.216251	1.109227	-1.769629
3	8	0	0.769414	1.818135	0.543881
4	6	0	2.307716	-1.009272	0.423421
5	1	0	1.653696	-1.673791	0.990400
6	1	0	3.112465	-1.575849	-0.040636
7	1	0	2.714413	-0.228022	1.066742
8	8	0	1.566170	-0.412345	-0.680773
9	6	0	-1.080410	-0.216309	0.044111
10	9	0	-1.387504	-1.167904	-0.867420
11	9	0	-0.915676	-0.830025	1.238895
12	9	0	-2.143963	0.610685	0.146140

9_(P_III)_method_A.log

Input orientation:

Center Number	Atomic Number	Atomic Type	Coordinates (Angstroms) X	Y	Z
1	15	0	-0.596267	1.138756	-0.700650
2	8	0	-0.956147	2.276173	0.474740
3	1	0	-1.048679	3.156429	0.087857
4	6	0	1.169870	0.727917	-0.089424
5	9	0	1.657141	-0.333869	-0.784378
6	9	0	1.284749	0.436537	1.224615
7	9	0	1.994174	1.780950	-0.333596
8	6	0	-1.464449	-0.318205	-0.001641
9	6	0	-1.641671	-1.437124	-0.830573
10	6	0	-1.983747	-0.332364	1.301894
11	6	0	-2.306909	-2.568437	-0.352914
12	1	0	-1.261521	-1.427583	-1.849189
13	6	0	-2.660046	-1.460213	1.770297
14	1	0	-1.860303	0.536652	1.939259
15	6	0	-2.818606	-2.579586	0.947267
16	1	0	-2.434466	-3.432601	-0.998124
17	1	0	-3.061038	-1.466211	2.779753
18	1	0	-3.344522	-3.455345	1.316366

9_(P_III)_method_A_DCM.log

Input orientation:

Center Number	Atomic Number	Atomic Type	Coordinates (Angstroms) X	Y	Z

1	15	0	-0.598065	1.143352	-0.702440
2	8	0	-0.935532	2.287154	0.467810
3	1	0	-1.103307	3.152494	0.067843
4	6	0	1.167375	0.729484	-0.086052
5	9	0	1.651774	-0.357313	-0.745784
6	9	0	1.284532	0.471906	1.239940
7	9	0	2.011069	1.763351	-0.353214
8	6	0	-1.460757	-0.316989	-0.000101
9	6	0	-1.646386	-1.431075	-0.835076
10	6	0	-1.972575	-0.337946	1.307001
11	6	0	-2.311580	-2.564090	-0.359127
12	1	0	-1.275784	-1.416947	-1.856962
13	6	0	-2.648815	-1.467184	1.774105
14	1	0	-1.846865	0.526393	1.950320
15	6	0	-2.814961	-2.581953	0.944988
16	1	0	-2.446704	-3.423306	-1.009118
17	1	0	-3.044560	-1.477321	2.785434
18	1	0	-3.341294	-3.458133	1.311995

9_(P_III)_method_A_DCM_smd.log

Input orientation:

Center Number	Atomic Number	Atomic Type	Coordinates (Angstroms) X	Y	Z
1	15	0	-0.588706	1.129484	-0.708996
2	8	0	-0.938971	2.290936	0.438901
3	1	0	-1.114370	3.147043	0.015106
4	6	0	1.172429	0.736773	-0.066929
5	9	0	1.686055	-0.343119	-0.716249
6	9	0	1.277302	0.482738	1.261913
7	9	0	2.007197	1.782395	-0.322444
8	6	0	-1.457001	-0.324545	-0.001863
9	6	0	-1.661580	-1.431226	-0.842523
10	6	0	-1.954040	-0.348505	1.311282
11	6	0	-2.332691	-2.560919	-0.366222
12	1	0	-1.300221	-1.412864	-1.868092
13	6	0	-2.635821	-1.474567	1.778244
14	1	0	-1.813718	0.508907	1.961518
15	6	0	-2.821913	-2.582216	0.943330
16	1	0	-2.483238	-3.414822	-1.020458
17	1	0	-3.020200	-1.487718	2.794313
18	1	0	-3.352949	-3.455900	1.310730

9_(P_III)_method_B.log

Input orientation:

Center Number	Atomic Number	Atomic Type	Coordinates (Angstroms) X	Y	Z
1	15	0	0.861658	0.485766	-1.026557
2	8	0	1.168240	1.990709	-0.417095
3	1	0	1.723788	2.499092	-1.013795
4	6	0	1.791174	-0.505406	0.312511
5	9	0	1.549469	-1.823265	0.154342
6	9	0	1.488304	-0.188913	1.581276
7	9	0	3.120853	-0.319831	0.167996
8	6	0	-0.834709	0.218245	-0.414750
9	6	0	-1.562422	-0.838945	-0.966103
10	6	0	-1.434658	1.035879	0.545232

11	6	0	-2.862333	-1.092183	-0.546479
12	1	0	-1.113661	-1.465278	-1.726885
13	6	0	-2.739306	0.789329	0.951849
14	1	0	-0.882010	1.862262	0.967969
15	6	0	-3.452420	-0.276036	0.411425
16	1	0	-3.415591	-1.916862	-0.974767
17	1	0	-3.198943	1.427462	1.694589
18	1	0	-4.467936	-0.465534	0.732287

9_(P_III)_method_B_DCM.log

Input orientation:

Center Number	Atomic Number	Atomic Type	Coordinates (Angstroms) X	Y	Z
1	15	0	-0.857353	0.484812	1.032284
2	8	0	-1.184788	1.980879	0.425730
3	1	0	-1.681010	2.512613	1.056429
4	6	0	-1.784692	-0.504677	-0.311389
5	9	0	-1.522638	-1.823243	-0.185507
6	9	0	-1.502287	-0.163443	-1.583037
7	9	0	-3.118914	-0.348942	-0.158845
8	6	0	0.837281	0.216999	0.412158
9	6	0	1.563738	-0.846851	0.954063
10	6	0	1.439279	1.043812	-0.539356
11	6	0	2.863955	-1.097598	0.531843
12	1	0	1.116227	-1.479558	1.710157
13	6	0	2.744144	0.799540	-0.948991
14	1	0	0.890891	1.877489	-0.953371
15	6	0	3.455662	-0.272837	-0.418746
16	1	0	3.416355	-1.926236	0.953255
17	1	0	3.205131	1.445240	-1.684156
18	1	0	4.471102	-0.460204	-0.740649

9_(P_III)_method_B_DCM_smd.log

Input orientation:

Center Number	Atomic Number	Atomic Type	Coordinates (Angstroms) X	Y	Z
1	15	0	-0.856248	0.469317	1.030608
2	8	0	-1.191536	1.972573	0.448450
3	1	0	-1.668583	2.497756	1.103821
4	6	0	-1.790948	-0.495121	-0.325764
5	9	0	-1.546691	-1.818905	-0.219736
6	9	0	-1.506476	-0.141729	-1.594647
7	9	0	-3.124762	-0.327035	-0.173015
8	6	0	0.838725	0.209308	0.409399
9	6	0	1.572797	-0.842167	0.965433
10	6	0	1.433373	1.028892	-0.553443
11	6	0	2.875260	-1.087672	0.546267
12	1	0	1.128470	-1.468457	1.729313
13	6	0	2.740197	0.789350	-0.959722
14	1	0	0.879990	1.853222	-0.980458
15	6	0	3.460231	-0.270406	-0.414983
16	1	0	3.434482	-1.906543	0.978627
17	1	0	3.196175	1.429015	-1.703788
18	1	0	4.477627	-0.453602	-0.734491

9_(P_III)_method_C_DCM_smd.log

Input orientation:

Center Number	Atomic Number	Atomic Type	Coordinates (Angstroms) X	Y	Z
1	15	0	-0.862481	0.476619	1.030820
2	8	0	-1.193243	1.986549	0.433069
3	1	0	-1.682306	2.508030	1.083497
4	6	0	-1.800484	-0.498659	-0.325569
5	9	0	-1.554763	-1.824450	-0.211726
6	9	0	-1.513065	-0.151814	-1.598355
7	9	0	-3.137377	-0.331091	-0.176748
8	6	0	0.838713	0.210566	0.409123
9	6	0	1.576488	-0.825389	0.987703
10	6	0	1.425940	1.011531	-0.573279
11	6	0	2.879605	-1.073757	0.571520
12	1	0	1.135629	-1.436508	1.765742
13	6	0	2.733084	0.768074	-0.976420
14	1	0	0.867670	1.823698	-1.017009
15	6	0	3.458904	-0.275903	-0.409138
16	1	0	3.443674	-1.880063	1.020869
17	1	0	3.185072	1.392179	-1.735962
18	1	0	4.476387	-0.461788	-0.726658

9_(P_III)_method_D_DCM.log

Input orientation:

Center Number	Atomic Number	Atomic Type	Coordinates (Angstroms) X	Y	Z
1	15	0	-0.867203	0.484569	1.054873
2	8	0	-1.179689	1.998431	0.422605
3	1	0	-1.695755	2.532514	1.042895
4	6	0	-1.768791	-0.497700	-0.313481
5	9	0	-1.497379	-1.825157	-0.192796
6	9	0	-1.458365	-0.143744	-1.584527
7	9	0	-3.116563	-0.354892	-0.191559
8	6	0	0.834820	0.215010	0.435594
9	6	0	1.566340	-0.854186	0.974887
10	6	0	1.424308	1.045446	-0.528973
11	6	0	2.867571	-1.106242	0.534608
12	1	0	1.123062	-1.489861	1.736667
13	6	0	2.729695	0.797584	-0.956480
14	1	0	0.864027	1.879565	-0.936458
15	6	0	3.450365	-0.279445	-0.429800
16	1	0	3.426951	-1.937674	0.951672
17	1	0	3.183555	1.442904	-1.702289
18	1	0	4.465134	-0.469326	-0.765568

9_(P_III)_method_E_DCM.log

Input orientation:

Center Number	Atomic Number	Atomic Type	Coordinates (Angstroms) X	Y	Z
1	15	0	-0.612053	1.144407	-0.687082
2	8	0	-0.924261	2.273173	0.452542
3	1	0	-1.087719	3.131520	0.058717
4	6	0	1.128384	0.695327	-0.088430

5	9	0	1.579519	-0.382791	-0.747762
6	9	0	1.232853	0.432874	1.218895
7	9	0	1.976508	1.703668	-0.347582
8	6	0	-1.469411	-0.298573	0.005339
9	6	0	-1.636684	-1.407475	-0.820872
10	6	0	-1.974044	-0.327037	1.302943
11	6	0	-2.282071	-2.540610	-0.349931
12	1	0	-1.264711	-1.387922	-1.838106
13	6	0	-2.631964	-1.455031	1.766537
14	1	0	-1.857442	0.533369	1.946956
15	6	0	-2.782529	-2.562925	0.943398
16	1	0	-2.403259	-3.398856	-0.996571
17	1	0	-3.026420	-1.469814	2.773619
18	1	0	-3.297131	-3.441427	1.308952

9_(P_III)_method_E_DCM_smd.log

Input orientation:

Center Number	Atomic Number	Atomic Type	Coordinates (Angstroms) X	Y	Z
1	15	0	-0.873875	0.491528	1.020257
2	8	0	-1.208431	1.967863	0.407690
3	1	0	-1.703363	2.499524	1.037756
4	6	0	-1.766242	-0.508198	-0.318437
5	9	0	-1.506864	-1.817053	-0.174634
6	9	0	-1.457596	-0.181193	-1.579013
7	9	0	-3.095423	-0.352950	-0.197733
8	6	0	0.818115	0.226479	0.417840
9	6	0	1.538771	-0.829350	0.971762
10	6	0	1.415551	1.037800	-0.543961
11	6	0	2.836173	-1.085314	0.554300
12	1	0	1.087639	-1.454000	1.733846
13	6	0	2.717516	0.788673	-0.948240
14	1	0	0.867540	1.865531	-0.973104
15	6	0	3.426454	-0.273956	-0.403530
16	1	0	3.387382	-1.910154	0.985741
17	1	0	3.178964	1.424749	-1.692139
18	1	0	4.443074	-0.464616	-0.721772

9_(P_III)method_C_DCM.log

Input orientation:

Center Number	Atomic Number	Atomic Type	Coordinates (Angstroms) X	Y	Z
1	15	0	-0.604958	1.140887	-0.696666
2	8	0	-0.939478	2.289674	0.450950
3	1	0	-1.106918	3.145090	0.040705
4	6	0	1.159862	0.720803	-0.079834
5	9	0	1.632970	-0.370872	-0.724116
6	9	0	1.270402	0.480102	1.243096
7	9	0	2.004349	1.742725	-0.357114
8	6	0	-1.465981	-0.314286	0.006581
9	6	0	-1.649172	-1.419890	-0.827754
10	6	0	-1.969572	-0.343409	1.308894
11	6	0	-2.305990	-2.551337	-0.357949
12	1	0	-1.283085	-1.398275	-1.846451
13	6	0	-2.637667	-1.470903	1.770248
14	1	0	-1.844353	0.513034	1.955421
15	6	0	-2.802369	-2.576562	0.940800
16	1	0	-2.439876	-3.404865	-1.008278
17	1	0	-3.028057	-1.487652	2.778807
18	1	0	-3.322544	-3.452388	1.304221

9_(P_V)_method_A.log

Input orientation:

Center Number	Atomic Number	Atomic Type	Coordinates (Angstroms) X	Y	Z
1	15	0	-0.087833	1.207502	-0.851957
2	1	0	0.247241	0.782944	-2.161268
3	8	0	-0.230463	2.676177	-0.616340
4	6	0	-1.487670	0.169986	-0.362719
5	6	0	-1.657371	-1.127900	-0.868724
6	6	0	-2.422627	0.706921	0.534741
7	6	0	-2.754595	-1.889869	-0.465726
8	1	0	-0.940887	-1.545763	-1.570728
9	6	0	-3.517991	-0.061486	0.932837
10	1	0	-2.290985	1.719738	0.903101
11	6	0	-3.682225	-1.357388	0.435437
12	1	0	-2.887424	-2.894189	-0.856189
13	1	0	-4.243016	0.352278	1.627026
14	1	0	-4.536503	-1.952112	0.745369
15	6	0	1.408463	0.457039	0.054623
16	9	0	1.282600	0.548168	1.392847
17	9	0	2.549397	1.082420	-0.303105
18	9	0	1.551546	-0.859441	-0.258951

9_(P_V)_method_A_DCM.log

Input orientation:

Center Number	Atomic Number	Atomic Type	Coordinates (Angstroms) X	Y	Z
1	15	0	-0.076404	1.140300	-0.928286
2	1	0	0.315356	0.600650	-2.171659
3	8	0	-0.212498	2.633805	-0.838393
4	6	0	-1.485742	0.147579	-0.399559
5	6	0	-1.727977	-1.110071	-0.975241
6	6	0	-2.346908	0.653230	0.587946
7	6	0	-2.823909	-1.864082	-0.551818
8	1	0	-1.073741	-1.500851	-1.749621
9	6	0	-3.440894	-0.106664	1.003886
10	1	0	-2.166367	1.633165	1.018627
11	6	0	-3.677501	-1.363356	0.436482
12	1	0	-3.014310	-2.835403	-0.997162
13	1	0	-4.108480	0.282501	1.766122
14	1	0	-4.531558	-1.950323	0.760501
15	6	0	1.382547	0.530656	0.133686
16	9	0	1.184386	0.790803	1.444983
17	9	0	2.533171	1.139045	-0.234632
18	9	0	1.570486	-0.805959	0.004413

9_(P_V)_method_A_DCM_smd.log

Input orientation:

Center Atomic Atomic Coordinates
(Angstroms)

Number	Number	Type	X	Y	Z
1	15	0	-0.072715	1.114134	-0.950661
2	1	0	0.344943	0.516887	-2.158356
3	8	0	-0.208118	2.611193	-0.928069
4	6	0	-1.486336	0.136562	-0.403747
5	6	0	-1.757416	-1.101865	-1.007770
6	6	0	-2.320644	0.628169	0.613942
7	6	0	-2.855554	-1.852320	-0.582498
8	1	0	-1.122691	-1.476080	-1.806663
9	6	0	-3.417655	-0.127373	1.029955
10	1	0	-2.117939	1.592298	1.070564
11	6	0	-3.683014	-1.365823	0.434700
12	1	0	-3.067761	-2.809686	-1.048920
13	1	0	-4.065242	0.250710	1.815334
14	1	0	-4.539407	-1.949361	0.760302
15	6	0	1.378570	0.565836	0.155854
16	9	0	1.184769	0.906187	1.450653
17	9	0	2.532631	1.151446	-0.246097
18	9	0	1.573235	-0.775890	0.111752

9_(P_V)_method_B.log

Input orientation:

Center Number	Atomic Number	Atomic Type	Coordinates (Angstroms) X	Y	Z
1	15	0	0.818205	0.714571	-0.688244
2	1	0	1.026247	0.234884	-1.999744
3	8	0	1.223438	2.100651	-0.398459
4	6	0	-0.879599	0.271917	-0.299040
5	6	0	-1.492357	-0.853249	-0.854727
6	6	0	-1.588901	1.094616	0.577913
7	6	0	-2.805719	-1.157719	-0.525396
8	1	0	-0.950548	-1.490461	-1.541258
9	6	0	-2.902971	0.784536	0.902812
10	1	0	-1.109749	1.972842	0.988069
11	6	0	-3.508968	-0.340180	0.353932
12	1	0	-3.280940	-2.028115	-0.956295
13	1	0	-3.453503	1.421631	1.581143
14	1	0	-4.533199	-0.578505	0.607189
15	6	0	1.878920	-0.562077	0.227652
16	9	0	1.733259	-0.467843	1.554730
17	9	0	3.176225	-0.392311	-0.063251
18	9	0	1.542539	-1.818884	-0.137090

9_(P_V)_method_B_DCM.log

Input orientation:

Center Number	Atomic Number	Atomic Type	Coordinates (Angstroms) X	Y	Z
1	15	0	0.803861	0.651782	-0.767865
2	1	0	1.018328	0.038580	-2.015863
3	8	0	1.232853	2.060897	-0.621985
4	6	0	-0.888329	0.254610	-0.338364
5	6	0	-1.550883	-0.804226	-0.964691
6	6	0	-1.543294	1.017000	0.632845
7	6	0	-2.860543	-1.102620	-0.612749
8	1	0	-1.053947	-1.389743	-1.726887
9	6	0	-2.852821	0.713260	0.978411
10	1	0	-1.033240	1.844900	1.105248
11	6	0	-3.509065	-0.345798	0.358073
12	1	0	-3.375253	-1.919127	-1.099462
13	1	0	-3.361178	1.303247	1.728119
14	1	0	-4.530290	-0.577933	0.627670
15	6	0	1.869174	-0.490184	0.308002
16	9	0	1.721523	-0.212631	1.613055
17	9	0	3.170371	-0.347673	0.005140
18	9	0	1.549559	-1.785175	0.125010

9_(P_V)_method_B_DCM_smd.log

Input orientation:

Center Number	Atomic Number	Atomic Type	Coordinates (Angstroms) X	Y	Z
1	15	0	0.799841	0.630251	-0.784602
2	1	0	1.016749	-0.035746	-2.004375
3	8	0	1.232962	2.043522	-0.691991
4	6	0	-0.891897	0.245346	-0.340762
5	6	0	-1.568790	-0.789047	-0.992159
6	6	0	-1.533278	0.988850	0.654296
7	6	0	-2.880217	-1.082864	-0.641537
8	1	0	-1.079639	-1.355885	-1.773725
9	6	0	-2.844801	0.690441	0.996964
10	1	0	-1.012748	1.797130	1.149524
11	6	0	-3.515925	-0.344744	0.351798
12	1	0	-3.406072	-1.881228	-1.147139
13	1	0	-3.343430	1.265998	1.764872
14	1	0	-4.538928	-0.572462	0.620218
15	6	0	1.877414	-0.462621	0.330528
16	9	0	1.758647	-0.125868	1.625552
17	9	0	3.175686	-0.335134	-0.001038
18	9	0	1.561253	-1.766774	0.217282

9_(P_V)_method_C_DCM.log

Input orientation:

Center Number	Atomic Number	Atomic Type	Coordinates (Angstroms) X	Y	Z
1	15	0	-0.084805	1.140538	-0.918540
2	1	0	0.297902	0.608744	-2.167870
3	8	0	-0.216142	2.623654	-0.819854
4	6	0	-1.491145	0.149175	-0.395369
5	6	0	-1.725432	-1.104505	-0.964946
6	6	0	-2.351413	0.650935	0.584297
7	6	0	-2.814759	-1.857119	-0.546101
8	1	0	-1.069983	-1.491439	-1.734015
9	6	0	-3.438431	-0.107729	0.996740
10	1	0	-2.174730	1.628296	1.011339
11	6	0	-3.668082	-1.359686	0.433865
12	1	0	-3.000503	-2.826060	-0.987797
13	1	0	-4.106710	0.278308	1.753648
14	1	0	-4.517602	-1.946421	0.755433
15	6	0	1.381372	0.522875	0.128525
16	9	0	1.203436	0.801751	1.432217
17	9	0	2.525555	1.113615	-0.263641
18	9	0	1.551128	-0.809909	0.012342

9_(P_V)_method_C_DCM_smd.log

Input orientation:

Center Number	Atomic Number	Atomic Type	Coordinates (Angstroms) X	Y	Z
1	15	0	0.801961	0.630109	-0.789497
2	1	0	1.022085	-0.041439	-2.009928
3	8	0	1.232262	2.057249	-0.703273
4	6	0	-0.895512	0.240066	-0.340147
5	6	0	-1.569785	-0.794571	-0.992654
6	6	0	-1.536225	0.984650	0.653646
7	6	0	-2.881559	-1.088515	-0.642751
8	1	0	-1.079366	-1.361255	-1.773625
9	6	0	-2.847943	0.685185	0.995498
10	1	0	-1.016176	1.793391	1.148737
11	6	0	-3.518129	-0.350400	0.350029
12	1	0	-3.407003	-1.887197	-1.148254
13	1	0	-3.347622	1.260365	1.762995
14	1	0	-4.541192	-0.578436	0.617782
15	6	0	1.890432	-0.455888	0.335227
16	9	0	1.788108	-0.095427	1.627908
17	9	0	3.187857	-0.341284	-0.013870
18	9	0	1.564674	-1.762195	0.247899

9_(P_V)_method_D_DCM.log

Input orientation:

Center Number	Atomic Number	Atomic Type	Coordinates (Angstroms) X	Y	Z
1	15	0	0.812107	0.663867	-0.780668
2	1	0	1.036202	0.058519	-2.034563
3	8	0	1.247632	2.091470	-0.614631
4	6	0	-0.885990	0.260997	-0.347358
5	6	0	-1.545243	-0.807976	-0.972780
6	6	0	-1.539297	1.028194	0.629165
7	6	0	-2.857080	-1.114356	-0.609921
8	1	0	-1.044968	-1.395464	-1.736952
9	6	0	-2.851421	0.715698	0.984019
10	1	0	-1.026656	1.861978	1.097603
11	6	0	-3.507538	-0.354297	0.367070
12	1	0	-3.371662	-1.938661	-1.092500
13	1	0	-3.361349	1.306721	1.737617
14	1	0	-4.529512	-0.592789	0.644124
15	6	0	1.854988	-0.494082	0.305871
16	9	0	1.695127	-0.210048	1.617548
17	9	0	3.171174	-0.378294	0.018263
18	9	0	1.510311	-1.792313	0.121800

9_(P_V)_method_E_DCM.log

Input orientation:

Center Number	Atomic Number	Atomic Type	Coordinates (Angstroms) X	Y	Z
1	15	0	-0.087869	1.146837	-0.915175
2	1	0	0.288485	0.625126	-2.164157
3	8	0	-0.208346	2.610864	-0.798802
4	6	0	-1.485913	0.164942	-0.402567
5	6	0	-1.702685	-1.095460	-0.953475
6	6	0	-2.350017	0.668375	0.566885
7	6	0	-2.782726	-1.852195	-0.530589
8	1	0	-1.037370	-1.484916	-1.713520
9	6	0	-3.429181	-0.092802	0.983378
10	1	0	-2.182269	1.652907	0.982516
11	6	0	-3.642995	-1.350612	0.436195
12	1	0	-2.955601	-2.829689	-0.958492
13	1	0	-4.105071	0.296123	1.732141
14	1	0	-4.490142	-1.940037	0.759805
15	6	0	1.342633	0.497556	0.124523
16	9	0	1.119899	0.687916	1.426003
17	9	0	2.478517	1.122451	-0.191864
18	9	0	1.530310	-0.812363	-0.072531

9_(P_V)_method_E_DCM_smd.log

Input orientation:

Center Number	Atomic Number	Atomic Type	Coordinates (Angstroms) X	Y	Z
1	15	0	0.816380	0.662341	-0.766407
2	1	0	1.039595	0.055198	-2.013093
3	8	0	1.250316	2.061489	-0.600971
4	6	0	-0.871224	0.266529	-0.343966
5	6	0	-1.509963	-0.812431	-0.950151
6	6	0	-1.535734	1.036777	0.607545
7	6	0	-2.814791	-1.120061	-0.601450
8	1	0	-0.994376	-1.407606	-1.693808
9	6	0	-2.841143	0.724505	0.949085
10	1	0	-1.036899	1.878331	1.070109
11	6	0	-3.477710	-0.352108	0.346074
12	1	0	-3.315181	-1.955352	-1.072110
13	1	0	-3.362678	1.322488	1.684125
14	1	0	-4.499499	-0.589533	0.611495
15	6	0	1.847919	-0.490524	0.310145
16	9	0	1.660681	-0.245124	1.609139
17	9	0	3.150164	-0.336676	0.052964
18	9	0	1.543211	-1.775387	0.090393

10_(P_III)_method_A.log

Input orientation:

Center Number	Atomic Number	Atomic Type	Coordinates (Angstroms) X	Y	Z
1	15	0	0.001584	0.804555	0.452335
2	8	0	1.438840	0.873418	1.269764
3	1	0	1.347797	1.366600	2.094886
4	6	0	0.639821	1.358809	-1.243986
5	6	0	-0.025909	-1.062974	0.130411
6	8	0	0.688665	-1.859099	0.691885
7	8	0	1.715787	1.876448	-1.428429
8	8	0	-0.311661	1.193978	-2.179706
9	8	0	-1.018284	-1.377577	-0.720632
10	6	0	-1.203561	-2.786956	-0.988477
11	1	0	-2.045250	-2.839133	-1.677595
12	1	0	-0.302844	-3.205934	-1.442604
13	1	0	-1.425472	-3.320409	-0.061548
14	6	0	0.017755	1.656472	-3.510118

15	1	0	0.884522	1.113866	-3.893989
16	1	0	-0.866216	1.450306	-4.111896
17	1	0	0.236603	2.726445	-3.493360

10_(P_III)_method_A_DCM.log

Input orientation:

Center	Atomic	Atomic	Coordinates (Angstroms)		
Number	Number	Type	X	Y	Z
1	15	0	-0.010403	0.809185	0.457385
2	8	0	1.420128	0.890828	1.289434
3	1	0	1.309284	1.387277	2.112986
4	6	0	0.642672	1.345475	-1.241582
5	6	0	-0.012198	-1.058118	0.119924
6	8	0	0.751733	-1.844822	0.637404
7	8	0	1.746690	1.809475	-1.431669
8	8	0	-0.316902	1.227159	-2.164937
9	8	0	-1.028557	-1.381877	-0.686026
10	6	0	-1.202591	-2.792441	-0.988208
11	1	0	-2.065247	-2.836934	-1.649925
12	1	0	-0.311590	-3.179476	-1.485492
13	1	0	-1.389426	-3.348703	-0.068022
14	6	0	0.011782	1.661381	-3.512038
15	1	0	0.850123	1.078408	-3.897131
16	1	0	-0.887541	1.478855	-4.096785
17	1	0	0.264219	2.723146	-3.508379

10_(P_III)_method_A_DCM_smd.log

Input orientation:

Center	Atomic	Atomic	Coordinates (Angstroms)		
Number	Number	Type	X	Y	Z
1	15	0	-0.015569	0.811988	0.459280
2	8	0	1.404833	0.905145	1.304485
3	1	0	1.280342	1.405731	2.127270
4	6	0	0.646233	1.338064	-1.239226
5	6	0	-0.003320	-1.054097	0.116629
6	8	0	0.782467	-1.835469	0.608927
7	8	0	1.761805	1.770957	-1.435385
8	8	0	-0.322295	1.244439	-2.154734
9	8	0	-1.034812	-1.380465	-0.667205
10	6	0	-1.197842	-2.788694	-0.990299
11	1	0	-2.080121	-2.833501	-1.627443
12	1	0	-0.318271	-3.154595	-1.524434
13	1	0	-1.352739	-3.366418	-0.076559
14	6	0	0.008661	1.656218	-3.509374
15	1	0	0.821484	1.040205	-3.900066
16	1	0	-0.902849	1.499022	-4.084532
17	1	0	0.294170	2.710288	-3.520393

10_(P_III)_method_B.log

Input orientation:

Center	Atomic	Atomic	Coordinates (Angstroms)		
Number	Number	Type	X	Y	Z
1	15	0	-0.000116	1.081072	-0.771752
2	8	0	-0.000263	2.429335	0.148574
3	1	0	-0.000468	3.224298	-0.390531
4	6	0	1.421676	0.170894	0.071484
5	6	0	-1.421647	0.170514	0.071521
6	8	0	-2.184840	0.681326	0.841728
7	8	0	2.184799	0.681964	0.841590
8	8	0	1.517801	-1.081637	-0.393991
9	8	0	-1.517439	-1.082032	-0.393983
10	6	0	-2.617024	-1.864678	0.111233
11	1	0	-2.531309	-2.831329	-0.373233
12	1	0	-2.542071	-1.967381	1.191711
13	1	0	-3.563285	-1.390187	-0.139886
14	6	0	2.617686	-1.863926	0.111128
15	1	0	2.542863	-1.966655	1.191612
16	1	0	2.532243	-2.830604	-0.373332
17	1	0	3.563770	-1.389126	-0.140075

10_(P_III)_method_B_DCM.log

Input orientation:

Center	Atomic	Atomic	Coordinates (Angstroms)		
Number	Number	Type	X	Y	Z
1	15	0	0.000262	1.078183	-0.789416
2	8	0	0.000658	2.441011	0.110203
3	1	0	0.000977	3.226291	-0.446747
4	6	0	1.408888	0.168431	0.080238
5	6	0	-1.409558	0.169604	0.079535
6	8	0	-2.136125	0.673759	0.894990
7	8	0	2.136002	0.672305	0.895378
8	8	0	1.535085	-1.064053	-0.408152
9	8	0	-1.535831	-1.063157	-0.408140
10	6	0	-2.617599	-1.867106	0.118605
11	1	0	-2.544373	-2.819285	-0.393947
12	1	0	-2.497288	-1.997502	1.191087
13	1	0	-3.571390	-1.389476	-0.090924
14	6	0	2.616750	-1.868399	0.118203
15	1	0	2.496323	-1.999444	1.190592
16	1	0	2.543509	-2.820257	-0.394943
17	1	0	3.570594	-1.390706	-0.090939

10_(P_III)_method_C_DCM.log

Input orientation:

Center	Atomic	Atomic	Coordinates (Angstroms)		
Number	Number	Type	X	Y	Z
1	15	0	-0.005488	0.808104	0.457900
2	8	0	1.408931	0.893440	1.291469
3	1	0	1.293870	1.388877	2.109634
4	6	0	0.645640	1.337389	-1.242142
5	6	0	-0.009248	-1.056259	0.114316
6	8	0	0.755969	-1.837075	0.618655
7	8	0	1.747144	1.784046	-1.433881
8	8	0	-0.316481	1.229519	-2.158643
9	8	0	-1.028961	-1.376451	-0.682581
10	6	0	-1.201534	-2.782231	-0.989854
11	1	0	-2.065174	-2.828790	-1.643419
12	1	0	-0.316889	-3.165001	-1.492796
13	1	0	-1.379397	-3.342812	-0.075389

14	6	0	0.012391	1.655736	-3.504324
15	1	0	0.840817	1.066507	-3.889740
16	1	0	-0.885386	1.483258	-4.086924
17	1	0	0.275974	2.710556	-3.505341

10_(P_III)_method_C_DCM_smd.log

Input orientation:

Center Number	Atomic Number	Atomic Type	Coordinates (Angstroms) X	Y	Z
1	15	0	-0.000147	1.084492	-0.800380
2	8	0	-0.000423	2.458520	0.099802
3	1	0	-0.000307	3.239437	-0.469521
4	6	0	1.408420	0.172504	0.080825
5	6	0	-1.408393	0.172037	0.080837
6	8	0	-2.119205	0.669576	0.915720
7	8	0	2.118890	0.670167	0.915927
8	8	0	1.543844	-1.053225	-0.422935
9	8	0	-1.543282	-1.053826	-0.422748
10	6	0	-2.613146	-1.870863	0.116906
11	1	0	-2.549289	-2.814266	-0.415578
12	1	0	-2.465799	-2.023779	1.184074
13	1	0	-3.574558	-1.394465	-0.063436
14	6	0	2.613822	-1.870017	0.116876
15	1	0	2.466453	-2.022824	1.184055
16	1	0	2.550152	-2.813493	-0.415499
17	1	0	3.575158	-1.393471	-0.063485

10_(P_III)_method_D_DCM.log

Input orientation:

Center Number	Atomic Number	Atomic Type	Coordinates (Angstroms) X	Y	Z
1	15	0	0.000096	1.094457	-0.817115
2	8	0	0.000490	2.462045	0.117295
3	1	0	0.000289	3.254670	-0.437877
4	6	0	1.402465	0.177583	0.063919
5	6	0	-1.401907	0.177716	0.064659
6	8	0	-2.122675	0.673280	0.904201
7	8	0	2.125242	0.673970	0.901246
8	8	0	1.523848	-1.058100	-0.429699
9	8	0	-1.525503	-1.056867	-0.431147
10	6	0	-2.586935	-1.877509	0.122933
11	1	0	-2.511788	-2.829558	-0.398036
12	1	0	-2.433493	-2.007680	1.195436
13	1	0	-3.554017	-1.407160	-0.062201
14	6	0	2.585543	-1.878696	0.123954
15	1	0	2.433991	-2.006659	1.196991
16	1	0	2.508512	-2.831668	-0.395045
17	1	0	3.552723	-1.409627	-0.063893

10_(P_III)_method_E_DCM.log

Input orientation:

Center Number	Atomic Number	Atomic Type	Coordinates (Angstroms) X	Y	Z
1	15	0	-0.006306	0.810658	0.464689
2	8	0	1.389310	0.900902	1.284520
3	1	0	1.282311	1.391385	2.101095
4	6	0	0.626098	1.324084	-1.225583
5	6	0	-0.004161	-1.031188	0.106672
6	8	0	0.776780	-1.809605	0.578095
7	8	0	1.728787	1.746683	-1.434183
8	8	0	-0.338665	1.223212	-2.127025
9	8	0	-1.026443	-1.348937	-0.672742
10	6	0	-1.170638	-2.734419	-1.013785
11	1	0	-2.042468	-2.787741	-1.655840
12	1	0	-0.286361	-3.083885	-1.542091
13	1	0	-1.319713	-3.327873	-0.114569
14	6	0	-0.009463	1.606988	-3.469048
15	1	0	0.803065	0.989960	-3.846211
16	1	0	-0.910877	1.443834	-4.048702
17	1	0	0.280918	2.654756	-3.498351

10_(P_III)_method_E_DCM_smd.log

Input orientation:

Center Number	Atomic Number	Atomic Type	Coordinates (Angstroms) X	Y	Z
1	15	0	-0.000140	1.092487	-0.788932
2	8	0	-0.000325	2.443413	0.104648
3	1	0	-0.000537	3.228108	-0.451144
4	6	0	1.385950	0.173104	0.077942
5	6	0	-1.385800	0.172612	0.078113
6	8	0	-2.088410	0.649799	0.924757
7	8	0	2.088346	0.650438	0.924681
8	8	0	1.516714	-1.040341	-0.433788
9	8	0	-1.516368	-1.040789	-0.433764
10	6	0	-2.555210	-1.865221	0.114163
11	1	0	-2.492537	-2.807512	-0.420428
12	1	0	-2.389500	-2.022578	1.178486
13	1	0	-3.527216	-1.401944	-0.045197
14	6	0	2.555659	-1.864554	0.114271
15	1	0	2.390017	-2.021698	1.178636
16	1	0	2.493054	-2.806967	-0.420114
17	1	0	3.527612	-1.401217	-0.045237

10_(P_V)_method_A.log

Input orientation:

Center Number	Atomic Number	Atomic Type	Coordinates (Angstroms) X	Y	Z
1	8	0	-0.222667	2.503795	0.406713
2	6	0	1.502969	0.205609	0.957290
3	6	0	-1.387721	-0.055073	1.075950
4	8	0	1.656821	-0.992436	0.847350
5	8	0	-2.219480	-0.649350	0.430081
6	8	0	2.340834	1.064008	1.527225
7	8	0	-1.300734	-0.016163	2.409090
8	6	0	3.558942	0.503222	2.081396
9	1	0	4.097572	1.352549	2.497179
10	1	0	3.312648	-0.223849	2.857729
11	1	0	4.138215	0.019094	1.292663
12	6	0	-2.311458	-0.755605	3.141239

13	1	0	-2.081808	-0.587170	4.191792
14	1	0	-3.303311	-0.374138	2.891326
15	1	0	-2.248528	-1.816493	2.891065
16	15	0	-0.057914	1.023998	0.278060
17	1	0	-0.082715	0.477730	-1.024720

10_(P_V)_method_A_DCM.log

Input orientation:

Center Number	Atomic Number	Atomic Type	Coordinates (Angstroms) X	Y	Z
1	8	0	-0.028416	1.955502	-0.676426
2	6	0	1.565566	0.075340	0.865656
3	6	0	-1.356279	0.398866	1.297124
4	8	0	2.004346	-1.051501	0.778455
5	8	0	-2.222324	1.225551	1.467405
6	8	0	2.082408	1.081355	1.558930
7	8	0	-1.198397	-0.744040	1.957310
8	6	0	3.315628	0.815668	2.289031
9	1	0	3.559731	1.755090	2.779485
10	1	0	3.145496	0.023734	3.019689
11	1	0	4.099382	0.524307	1.588514
12	6	0	-2.174955	-1.053587	2.995339
13	1	0	-1.860402	-2.012388	3.400805
14	1	0	-2.152915	-0.276968	3.760855
15	1	0	-3.169101	-1.122301	2.551751
16	15	0	-0.014555	0.603706	-0.025309
17	1	0	-0.203550	-0.518602	-0.857184

10_(P_V)_method_A_DCM_smd.log

Input orientation:

Center Number	Atomic Number	Atomic Type	Coordinates (Angstroms) X	Y	Z
1	8	0	-0.049441	2.056664	-0.630717
2	6	0	1.539114	0.099780	0.816782
3	6	0	-1.383494	0.379146	1.241010
4	8	0	2.005705	-1.005520	0.637825
5	8	0	-2.374240	1.071606	1.300639
6	8	0	2.022105	1.050050	1.605891
7	8	0	-1.085642	-0.658025	2.016756
8	6	0	3.247580	0.743667	2.335314
9	1	0	3.467822	1.643999	2.906138
10	1	0	3.078699	-0.106645	2.998971
11	1	0	4.050926	0.525110	1.629150
12	6	0	-2.056635	-1.024221	3.043445
13	1	0	-1.618925	-1.881530	3.552000
14	1	0	-2.193423	-0.189838	3.733673
15	1	0	-3.004485	-1.292536	2.573222
16	15	0	-0.034138	0.669839	-0.055506
17	1	0	-0.219866	-0.401818	-0.953162

10_(P_V)_method_B.log

Input orientation:

Center Number	Atomic Number	Atomic Type	Coordinates (Angstroms) X	Y	Z
1	8	0	-0.316569	2.511406	0.105753
2	6	0	-1.353752	-0.126332	0.539040
3	6	0	1.464042	0.318733	-0.210787
4	8	0	-1.774330	-0.689806	1.511863
5	8	0	2.136694	0.824403	-1.059303
6	8	0	-1.774200	-0.294683	-0.711548
7	8	0	1.623724	-0.907604	0.299158
8	6	0	-2.863109	-1.224486	-0.906249
9	1	0	-3.063709	-1.212859	-1.971508
10	1	0	-2.568595	-2.217974	-0.575959
11	1	0	-3.734536	-0.897035	-0.344347
12	6	0	2.719732	-1.688051	-0.229628
13	1	0	2.686133	-2.631894	0.302766
14	1	0	2.586107	-1.839105	-1.298229
15	1	0	3.661311	-1.174495	-0.050253
16	15	0	0.002666	1.171994	0.621723
17	1	0	0.327623	1.056185	1.987429

10_(P_V)_method_B_DCM.log

Input orientation:

Center Number	Atomic Number	Atomic Type	Coordinates (Angstroms) X	Y	Z
1	8	0	0.230699	-2.379102	-0.219411
2	6	0	1.353012	0.206452	0.382726
3	6	0	-1.514362	-0.160028	-0.216419
4	8	0	1.552810	0.964189	1.295267
5	8	0	-2.157676	-0.571754	-1.141750
6	8	0	2.011360	0.148719	-0.760423
7	8	0	-1.726465	0.951068	0.470566
8	6	0	3.103763	1.088470	-0.938343
9	1	0	3.507548	0.872731	-1.919978
10	1	0	2.724255	2.105393	-0.888130
11	1	0	3.852005	0.929394	-0.166692
12	6	0	-2.854195	1.773524	0.068752
13	1	0	-2.845536	2.619294	0.745395
14	1	0	-2.722783	2.096853	-0.960216
15	1	0	-3.774682	1.204975	0.168461
16	15	0	-0.012602	-1.085357	0.451900
17	1	0	-0.243238	-1.103898	1.837257

10_(P_V)_method_B_DCM_smd.log

Input orientation:

Center Number	Atomic Number	Atomic Type	Coordinates (Angstroms) X	Y	Z
1	8	0	0.241632	-2.431053	-0.222279
2	6	0	1.344960	0.144803	0.437454
3	6	0	-1.511095	-0.215687	-0.189503
4	8	0	1.625738	0.801032	1.406181
5	8	0	-2.248103	-0.685315	-1.012332
6	8	0	1.907799	0.211912	-0.755667
7	8	0	-1.610102	0.974262	0.381931
8	6	0	2.985370	1.171909	-0.923501
9	1	0	3.306713	1.062263	-1.953293
10	1	0	2.614823	2.177662	-0.739686
11	1	0	3.797376	0.937443	-0.239355

12	6	0	-2.730412	1.811103	-0.014777
13	1	0	-2.624589	2.722507	0.563058
14	1	0	-2.673752	2.020889	-1.080233
15	1	0	-3.665639	1.309973	0.223091
16	15	0	-0.015141	-1.151692	0.472829
17	1	0	-0.261663	-1.201088	1.855047

10_(P_V)_method_C_DCM.log

Input orientation:

Center Number	Atomic Number	Atomic Type	Coordinates (Angstroms) X	Y	Z
1	8	0	-0.025512	1.916105	-0.621334
2	6	0	1.580581	0.093214	0.960249
3	6	0	-1.343193	0.389401	1.375648
4	8	0	1.942166	-1.053320	1.022135
5	8	0	-2.076818	1.290400	1.678764
6	8	0	2.180791	1.148490	1.482608
7	8	0	-1.333465	-0.837899	1.873988
8	6	0	3.421156	0.908967	2.203048
9	1	0	3.746064	1.885926	2.540162
10	1	0	3.233528	0.248061	3.045092
11	1	0	4.152690	0.465208	1.533057
12	6	0	-2.310697	-1.139791	2.908692
13	1	0	-2.128621	-2.173246	3.178005
14	1	0	-2.156587	-0.482020	3.759861
15	1	0	-3.313978	-1.011684	2.511369
16	15	0	-0.001397	0.587505	0.052322
17	1	0	-0.175044	-0.555589	-0.752235

10_(P_V)_method_C_DCM_smd.log

Input orientation:

Center Number	Atomic Number	Atomic Type	Coordinates (Angstroms) X	Y	Z
1	8	0	0.295749	-2.374603	-0.015218
2	6	0	1.349126	0.300058	0.369797
3	6	0	-1.491269	-0.180714	-0.257001
4	8	0	1.419967	1.227116	1.135712
5	8	0	-1.961499	-0.567492	-1.293144
6	8	0	2.136560	0.057012	-0.661840
7	8	0	-1.907443	0.839118	0.477515
8	6	0	3.212494	1.006473	-0.905716
9	1	0	3.728239	0.630894	-1.782707
10	1	0	2.796545	1.993525	-1.094442
11	1	0	3.879954	1.034410	-0.047470
12	6	0	-3.047059	1.593756	-0.024264
13	1	0	-3.214660	2.375709	0.708443
14	1	0	-2.807484	2.019277	-0.996070
15	1	0	-3.914134	0.941616	-0.099545
16	15	0	0.008116	-1.021354	0.539186
17	1	0	-0.257477	-0.921529	1.918636

10_(P_V)_method_D_DCM.log

Input orientation:

Center Number	Atomic Number	Atomic Type	Coordinates (Angstroms) X	Y	Z
1	8	0	0.240960	-2.496771	-0.211498
2	6	0	1.340644	0.099785	0.477893
3	6	0	-1.502021	-0.250503	-0.173719
4	8	0	1.681274	0.699737	1.474532
5	8	0	-2.285978	-0.733057	-0.957673
6	8	0	1.826435	0.248116	-0.747617
7	8	0	-1.531645	0.976048	0.337659
8	6	0	2.885044	1.233404	-0.917661
9	1	0	3.133859	1.199673	-1.975525
10	1	0	2.515393	2.218931	-0.631490
11	1	0	3.742873	0.956716	-0.303552
12	6	0	-2.631943	1.838148	-0.072963
13	1	0	-2.464536	2.776814	0.449519
14	1	0	-2.600692	1.978050	-1.154048
15	1	0	-3.578642	1.385736	0.224603
16	15	0	-0.018102	-1.203327	0.502679
17	1	0	-0.269011	-1.266576	1.887825

10_(P_V)_method_E_DCM.log

Input orientation:

Center Number	Atomic Number	Atomic Type	Coordinates (Angstroms) X	Y	Z
1	8	0	-0.028326	1.936627	-0.647296
2	6	0	1.540252	0.094425	0.885318
3	6	0	-1.330428	0.390873	1.301068
4	8	0	1.947520	-1.032894	0.847955
5	8	0	-2.160679	1.224257	1.517568
6	8	0	2.073915	1.103581	1.536219
7	8	0	-1.195212	-0.768822	1.910673
8	6	0	3.273801	0.828029	2.280891
9	1	0	3.572121	1.775882	2.712539
10	1	0	3.062626	0.097393	3.058021
11	1	0	4.043526	0.448588	1.613400
12	6	0	-2.136345	-1.068038	2.957181
13	1	0	-1.846619	-2.039507	3.339495
14	1	0	-2.070730	-0.311788	3.735535
15	1	0	-3.144138	-1.096881	2.550522
16	15	0	-0.013936	0.609948	-0.012769
17	1	0	-0.195686	-0.511944	-0.834890

10_(P_V)_method_E_DCM_smd.log

Input orientation:

Center Number	Atomic Number	Atomic Type	Coordinates (Angstroms) X	Y	Z
1	8	0	0.259113	-2.445008	-0.190357
2	6	0	1.321156	0.133466	0.456751
3	6	0	-1.486994	-0.241666	-0.184501
4	8	0	1.591899	0.795555	1.419718
5	8	0	-2.218988	-0.710933	-1.006543
6	8	0	1.874009	0.212628	-0.732919
7	8	0	-1.578598	0.951621	0.365662
8	6	0	2.913639	1.194800	-0.899443
9	1	0	3.271063	1.069105	-1.915700

10	1	0	2.503292	2.192653	-0.757392
11	1	0	3.714814	1.012845	-0.186296
12	6	0	-2.668091	1.786974	-0.068796
13	1	0	-2.562541	2.714076	0.484027
14	1	0	-2.588744	1.968352	-1.138754
15	1	0	-3.617483	1.308002	0.161361
16	15	0	-0.012508	-1.172478	0.496456
17	1	0	-0.272449	-1.214553	1.874292

10_P_III_method_B_DCM_smd.log

Input orientation:

Center Number	Atomic Number	Atomic Type	Coordinates (Angstroms) X	Y	Z
1	15	0	-0.000110	1.083900	-0.795194
2	8	0	-0.000270	2.451740	0.094374
3	1	0	-0.000561	3.234694	-0.471056
4	6	0	1.403395	0.173492	0.081026
5	6	0	-1.403255	0.173041	0.081142
6	8	0	-2.115546	0.668543	0.914454
7	8	0	2.115624	0.669217	0.914257
8	8	0	1.538940	-1.050943	-0.422170
9	8	0	-1.538544	-1.051395	-0.422120
10	6	0	-2.607038	-1.867724	0.115089
11	1	0	-2.543539	-2.810963	-0.417255
12	1	0	-2.461103	-2.021430	1.182166
13	1	0	-3.568411	-1.391674	-0.065281
14	6	0	2.607670	-1.866988	0.115005
15	1	0	2.461846	-2.020674	1.182100
16	1	0	2.544360	-2.810271	-0.417284
17	1	0	3.568918	-1.390720	-0.065455

11_(P_III)_method_A.log

Input orientation:

Center Number	Atomic Number	Atomic Type	Coordinates (Angstroms) X	Y	Z
1	15	0	-0.325571	0.732573	0.212335
2	8	0	0.909273	0.404342	1.287126
3	1	0	0.892546	1.011007	2.040740
4	6	0	0.674601	1.438413	-1.232343
5	6	0	-0.367088	-0.992295	-0.532521
6	1	0	0.246094	1.219819	-2.211023
7	1	0	1.708305	1.085614	-1.170790
8	1	0	-1.014236	-0.988671	-1.414051
9	1	0	0.622051	-1.383946	-0.772678
10	8	0	-0.375520	-2.998641	0.666411
11	8	0	0.529824	3.606416	-2.108681
12	7	0	-0.991332	-1.964496	0.434495
13	7	0	0.736732	2.933015	-1.105705
14	8	0	-2.079799	-1.653724	0.913217
15	8	0	0.996057	3.377183	0.013825

11_(P_III)_method_A_DCM.log

Input orientation:

Center Number	Atomic Number	Atomic Type	Coordinates (Angstroms) X	Y	Z
1	15	0	-0.196016	0.696900	0.310249
2	8	0	1.282503	0.484251	1.050199
3	1	0	1.261040	0.759871	1.978837
4	6	0	0.421724	1.493694	-1.289617
5	6	0	-0.352368	-1.007298	-0.499766
6	1	0	-0.387187	1.492097	-2.022631
7	1	0	1.321044	1.013968	-1.675099
8	1	0	-1.044657	-0.947484	-1.340933
9	1	0	0.619433	-1.402385	-0.795271
10	8	0	-0.192893	-2.764728	1.034126
11	8	0	1.948815	3.247196	-1.040434
12	7	0	-0.948912	-1.971879	0.473880
13	7	0	0.761318	2.927800	-1.033768
14	8	0	-2.160734	-1.895368	0.678706
15	8	0	-0.171174	3.699976	-0.808121

11_(P_III)_method_A_DCM_smd.log

Input orientation:

Center Number	Atomic Number	Atomic Type	Coordinates (Angstroms) X	Y	Z
1	15	0	-0.197666	0.693707	0.311057
2	8	0	1.275090	0.494550	1.069412
3	1	0	1.229549	0.773498	1.999696
4	6	0	0.416545	1.490862	-1.283891
5	6	0	-0.341785	-1.005217	-0.503552
6	1	0	-0.402306	1.491470	-2.007442
7	1	0	1.305402	1.001502	-1.682294
8	1	0	-1.025040	-0.932837	-1.352245
9	1	0	0.633720	-1.395131	-0.795370
10	8	0	-0.198084	-2.764239	1.030236
11	8	0	1.954789	3.243191	-1.099260
12	7	0	-0.947426	-1.972523	0.457469
13	7	0	0.769644	2.919961	-1.028810
14	8	0	-2.163195	-1.903753	0.646504
15	8	0	-0.147300	3.691567	-0.741155

11_(P_III)_method_B.log

Input orientation:

Center Number	Atomic Number	Atomic Type	Coordinates (Angstroms) X	Y	Z
1	15	0	0.000875	0.016455	-0.486302
2	8	0	0.160039	1.650363	-0.370159
3	1	0	-0.172308	2.096275	-1.155742
4	6	0	1.357021	-0.402728	0.727530
5	6	0	-1.356803	-0.291616	0.776196
6	1	0	1.296117	-1.462447	0.966672
7	1	0	1.327742	0.211848	1.622294
8	1	0	-1.270793	-1.251696	1.275709
9	1	0	-1.376559	0.522254	1.499786
10	8	0	-2.881454	0.648641	-0.691779
11	8	0	3.442109	0.623734	0.614125
12	7	0	-2.690725	-0.269914	0.090526
13	7	0	2.705699	-0.193687	0.097031

14	8	0	-3.481560	-1.153975	0.352762
15	8	0	2.958249	-0.862060	-0.889936

11_(P_III)_method_B_DCM.log

Input orientation:

Center Number	Atomic Number	Atomic Type	Coordinates (Angstroms) X	Y	Z
1	15	0	-0.005656	0.064441	0.477586
2	8	0	0.061206	-1.575235	0.543709
3	1	0	0.050608	-1.899661	1.451124
4	6	0	1.348617	0.355294	-0.790224
5	6	0	-1.363769	0.253847	-0.807093
6	1	0	1.279688	1.380869	-1.143133
7	1	0	1.311456	-0.359904	-1.605369
8	1	0	-1.291911	1.238319	-1.259945
9	1	0	-1.326718	-0.540815	-1.545158
10	8	0	-3.305108	-0.872428	-0.197930
11	8	0	3.325795	-0.795414	-0.369322
12	7	0	-2.698572	0.184442	-0.141525
13	7	0	2.684341	0.214880	-0.136125
14	8	0	-3.087096	1.183534	0.441829
15	8	0	3.039262	1.112236	0.611400

11_(P_III)_method_B_DCM_smd.log

Input orientation:

Center Number	Atomic Number	Atomic Type	Coordinates (Angstroms) X	Y	Z
1	15	0	-0.007196	0.070244	0.477850
2	8	0	0.066720	-1.569303	0.572241
3	1	0	0.054285	-1.870690	1.491023
4	6	0	1.342083	0.357478	-0.789021
5	6	0	-1.359386	0.244982	-0.809915
6	1	0	1.269497	1.386855	-1.133405
7	1	0	1.295108	-0.347586	-1.612920
8	1	0	-1.277449	1.228320	-1.266361
9	1	0	-1.316917	-0.551452	-1.546502
10	8	0	-3.316220	-0.865466	-0.219526
11	8	0	3.346624	-0.771197	-0.439896
12	7	0	-2.695058	0.183126	-0.150826
13	7	0	2.679482	0.206619	-0.144681
14	8	0	-3.076873	1.179745	0.443204
15	8	0	3.017444	1.062729	0.658558

11_(P_III)_method_D_DCM.log

Input orientation:

Center Number	Atomic Number	Atomic Type	Coordinates (Angstroms) X	Y	Z
1	15	0	-0.006503	0.140823	0.484368
2	8	0	0.058231	-1.520929	0.586608
3	1	0	0.048027	-1.822174	1.507024
4	6	0	1.354439	0.373183	-0.802758
5	6	0	-1.367761	0.275737	-0.818097
6	1	0	1.331409	1.402190	-1.165098
7	1	0	1.286343	-0.348102	-1.616102
8	1	0	-1.339695	1.267003	-1.272778
9	1	0	-1.295239	-0.520379	-1.558287
10	8	0	-3.289157	-0.926925	-0.257189
11	8	0	3.311396	-0.842719	-0.418674
12	7	0	-2.695675	0.145171	-0.150119
13	7	0	2.680362	0.176276	-0.145198
14	8	0	-3.101434	1.110050	0.498573
15	8	0	3.047400	1.035201	0.657551

11_(P_III)_method_E_DCM.log

Input orientation:

Center Number	Atomic Number	Atomic Type	Coordinates (Angstroms) X	Y	Z
1	15	0	-0.006358	-0.071873	-0.478070
2	8	0	0.056017	1.556598	-0.514567
3	1	0	0.047755	1.895220	-1.412187
4	6	0	1.335055	-0.381275	0.775510
5	6	0	-1.349667	-0.289453	0.793801
6	1	0	1.280459	-1.417747	1.100282
7	1	0	1.284571	0.309042	1.611977
8	1	0	-1.287047	-1.288840	1.216004
9	1	0	-1.300925	0.479343	1.558972
10	8	0	-3.256834	0.880174	0.216398
11	8	0	3.284507	0.802720	0.399211
12	7	0	-2.676027	-0.180231	0.136097
13	7	0	2.661501	-0.200826	0.131745
14	8	0	-3.083075	-1.151111	-0.465138
15	8	0	3.027716	-1.060297	-0.641322

11_(P_III)_method_E_DCM_smd.log

Input orientation:

Center Number	Atomic Number	Atomic Type	Coordinates (Angstroms) X	Y	Z
1	15	0	-0.007287	-0.072343	-0.479026
2	8	0	0.061866	1.556908	-0.531866
3	1	0	0.053532	1.879588	-1.438737
4	6	0	1.330207	-0.387843	0.770937
5	6	0	-1.345877	-0.286448	0.793245
6	1	0	1.265160	-1.427030	1.088234
7	1	0	1.276969	0.293368	1.615065
8	1	0	-1.269815	-1.286936	1.213599
9	1	0	-1.296805	0.479232	1.562143
10	8	0	-3.281848	0.854676	0.250908
11	8	0	3.313110	0.759748	0.456607
12	7	0	-2.673815	-0.188430	0.140892
13	7	0	2.658678	-0.207943	0.134495
14	8	0	-3.062121	-1.150913	-0.487409
15	8	0	3.002584	-1.031335	-0.687808

11_(P_V)_method_A.log

Input orientation:

Center Number	Atomic Number	Atomic Type	Coordinates (Angstroms) X	Y	Z
1	15	0	-0.000032	0.137474	0.193046
2	1	0	0.000009	-0.044619	1.588931
3	8	0	-0.000075	1.536557	-0.322360
4	6	0	1.421285	-0.918156	-0.385877
5	1	0	1.339292	-1.930946	0.018980
6	1	0	1.477307	-0.933100	-1.474545
7	6	0	-1.421324	-0.918222	-0.385824
8	1	0	-1.339244	-1.931024	0.018983
9	1	0	-1.477418	-0.933131	-1.474488
10	7	0	-2.725522	-0.367394	0.127670
11	7	0	2.725463	-0.367216	0.127523
12	8	0	-3.597424	-0.119895	-0.693055
13	8	0	-2.809873	-0.221253	1.345678
14	8	0	3.597388	-0.119888	-0.693227
15	8	0	2.810169	-0.221858	1.345595

11_(P_V)_method_A_DCM.log

Input orientation:

Center Number	Atomic Number	Atomic Type	Coordinates (Angstroms) X	Y	Z
1	15	0	0.001686	0.077743	0.260813
2	1	0	-0.038327	-0.144175	1.647694
3	8	0	0.034955	1.510886	-0.179880
4	6	0	1.423884	-0.963200	-0.330271
5	1	0	1.401981	-1.941551	0.157331
6	1	0	1.418440	-1.067212	-1.414357
7	6	0	-1.422494	-0.912119	-0.404605
8	1	0	-1.375253	-1.941384	-0.039532
9	1	0	-1.439677	-0.884306	-1.493480
10	7	0	-2.723174	-0.344351	0.086844
11	7	0	2.722343	-0.318945	0.060233
12	8	0	-3.559140	-0.022387	-0.749069
13	8	0	-2.861554	-0.245475	1.305802
14	8	0	3.538557	-0.091710	-0.824731
15	8	0	2.877775	-0.064485	1.254237

11_(P_V)_method_A_DCM_smd.log

Input orientation:

Center Number	Atomic Number	Atomic Type	Coordinates (Angstroms) X	Y	Z
1	15	0	0.000013	0.061876	0.281217
2	1	0	0.000038	-0.161302	1.669574
3	8	0	0.000037	1.499663	-0.151641
4	6	0	1.421435	-0.952428	-0.351592
5	1	0	1.398261	-1.946470	0.104449
6	1	0	1.395764	-1.015142	-1.439401
7	6	0	-1.421443	-0.952381	-0.351567
8	1	0	-1.398224	-1.946465	0.104388
9	1	0	-1.395845	-1.015025	-1.439381
10	7	0	-2.717310	-0.316300	0.046568
11	7	0	2.717300	-0.316265	0.046408
12	8	0	-3.479611	0.045859	-0.843847
13	8	0	-2.932654	-0.192151	1.253060
14	8	0	3.479353	0.046216	-0.844088
15	8	0	2.932887	-0.192356	1.252881

11_(P_V)_method_B.log

Input orientation:

Center Number	Atomic Number	Atomic Type	Coordinates (Angstroms) X	Y	Z
1	15	0	0.001669	0.180082	0.230819
2	1	0	0.009848	1.522972	-0.177380
3	8	0	-0.008340	-0.093804	1.674498
4	6	0	-1.406569	-0.548478	-0.714224
5	1	0	-1.331976	-0.288869	-1.769125
6	1	0	-1.449501	-1.624457	-0.576013
7	6	0	1.415264	-0.560284	-0.696923
8	1	0	1.355467	-0.300564	-1.752735
9	1	0	1.447787	-1.636504	-0.557765
10	7	0	2.719091	0.003876	-0.199042
11	7	0	-2.711688	0.026050	-0.231820
12	8	0	3.548602	-0.780307	0.207180
13	8	0	2.833835	1.215954	-0.257017
14	8	0	-3.552576	-0.751581	0.163465
15	8	0	-2.815681	1.239078	-0.290073

11_(P_V)_method_B_DCM.log

Input orientation:

Center Number	Atomic Number	Atomic Type	Coordinates (Angstroms) X	Y	Z
1	15	0	0.000966	-0.244347	-0.144119
2	1	0	0.046294	-1.485334	0.505678
3	8	0	-0.039708	-0.278670	-1.620316
4	6	0	-1.405831	0.632472	0.659709
5	1	0	-1.369958	0.478677	1.736666
6	1	0	-1.401365	1.688959	0.414768
7	6	0	1.413552	0.691224	0.577189
8	1	0	1.372866	0.649569	1.663919
9	1	0	1.414963	1.717147	0.224568
10	7	0	2.714632	0.062714	0.171650
11	7	0	-2.705804	0.054791	0.183105
12	8	0	3.510384	0.750889	-0.434834
13	8	0	2.879697	-1.104702	0.485039
14	8	0	-3.491453	0.802390	-0.363104
15	8	0	-2.878778	-1.136600	0.380093

11_(P_V)_method_B_DCM_smd.log

Input orientation:

Center Number	Atomic Number	Atomic Type	Coordinates (Angstroms) X	Y	Z
1	15	0	-0.005340	0.207494	0.211772
2	1	0	-0.049629	1.584236	-0.049279
3	8	0	0.010273	-0.184539	1.637827
4	6	0	-1.404124	-0.476915	-0.772707
5	1	0	-1.345567	-0.104371	-1.794034

6	1	0	-1.388155	-1.561819	-0.741649
7	6	0	1.407003	-0.409441	-0.791525
8	1	0	1.370149	0.035011	-1.785902
9	1	0	1.394914	-1.493368	-0.852006
10	7	0	2.709019	0.010555	-0.182222
11	7	0	-2.707951	-0.013085	-0.204432
12	8	0	3.549837	-0.845722	0.010342
13	8	0	2.841212	1.197169	0.072556
14	8	0	-3.423610	-0.840939	0.326772
15	8	0	-2.962496	1.177120	-0.303161

11_(P_V)_method_D_DCM.log

Input orientation:

Center	Atomic	Atomic	Coordinates (Angstroms)		
Number	Number	Type	X	Y	Z
1	15	0	0.001095	-0.257871	-0.128168
2	1	0	0.053055	-1.495106	0.534227
3	8	0	-0.044171	-0.307869	-1.625733
4	6	0	-1.413334	0.635351	0.671138
5	1	0	-1.393367	0.480780	1.752908
6	1	0	-1.403949	1.696031	0.425879
7	6	0	1.420297	0.701484	0.577920
8	1	0	1.389580	0.678150	1.669959
9	1	0	1.420605	1.725170	0.206645
10	7	0	2.713903	0.063424	0.168609
11	7	0	-2.704700	0.059266	0.174449
12	8	0	3.528809	0.753443	-0.431590
13	8	0	2.865425	-1.121266	0.466128
14	8	0	-3.516209	0.822461	-0.334505
15	8	0	-2.856582	-1.154269	0.312144

11_(P_V)_method_E_DCM.log

Input orientation:

Center	Atomic	Atomic	Coordinates (Angstroms)		
Number	Number	Type	X	Y	Z
1	15	0	-0.000075	0.252198	0.161600
2	1	0	0.027106	1.568411	-0.310704
3	8	0	-0.031901	0.076599	1.621991
4	6	0	-1.392961	-0.519682	-0.741845
5	1	0	-1.365621	-0.221826	-1.789262
6	1	0	-1.375695	-1.600502	-0.645335
7	6	0	1.399901	-0.566423	-0.685225
8	1	0	1.378148	-0.343620	-1.751257
9	1	0	1.382095	-1.638891	-0.518124
10	7	0	2.694402	-0.039174	-0.167769
11	7	0	-2.684521	-0.025529	-0.187862
12	8	0	3.536326	-0.838127	0.160781
13	8	0	2.813020	1.166679	-0.123913
14	8	0	-3.496090	-0.844264	0.167823
15	8	0	-2.828902	1.177315	-0.137055

12_(P_III)_method_A.log

Input orientation:

Center	Atomic	Atomic	Coordinates (Angstroms)		
Number	Number	Type	X	Y	Z
1	15	0	-0.317482	0.219188	-1.404237
2	8	0	0.882453	0.712840	-2.491906
3	1	0	0.517330	1.353121	-3.115843
4	6	0	0.294805	0.959579	0.177313
5	6	0	1.629711	0.852849	0.603922
6	6	0	-0.615638	1.679641	0.964205
7	6	0	2.040816	1.455785	1.792175
8	1	0	2.344889	0.299768	0.001916
9	6	0	-0.207377	2.273705	2.163924
10	1	0	-1.647850	1.778531	0.636508
11	6	0	1.121345	2.163569	2.576713
12	1	0	3.075780	1.371200	2.111960
13	1	0	1.443355	2.627845	3.504632
14	6	0	0.241936	-1.527882	-1.196406
15	6	0	-0.271139	-2.288990	-0.131952
16	6	0	1.087478	-2.148293	-2.127506
17	6	0	0.064739	-3.636155	0.005470
18	1	0	-0.929546	-1.827864	0.600657
19	6	0	1.424292	-3.498807	-1.986927
20	1	0	1.488299	-1.568882	-2.952437
21	6	0	0.914968	-4.246229	-0.923104
22	1	0	-0.335631	-4.209111	0.837208
23	1	0	2.086801	-3.964552	-2.711479
24	1	0	1.176152	-5.295236	-0.816848
25	1	0	-0.923276	2.824549	2.767468

12_(P_III)_method_A_DCM.log

Input orientation:

Center	Atomic	Atomic	Coordinates (Angstroms)		
Number	Number	Type	X	Y	Z
1	15	0	-0.009515	1.368198	-0.722948
2	8	0	0.115099	2.450506	0.571772
3	1	0	-0.064877	3.350677	0.266910
4	6	0	-1.433307	0.317706	-0.184859
5	6	0	-1.505457	-0.286593	1.083011
6	6	0	-2.500369	0.148835	-1.080320
7	6	0	-2.623379	-1.039113	1.444826
8	1	0	-0.686725	-0.167960	1.787133
9	6	0	-3.617038	-0.616611	-0.723472
10	1	0	-2.459921	0.617284	-2.060591
11	6	0	-3.679757	-1.208510	0.539877
12	1	0	-2.671086	-1.498735	2.428032
13	1	0	-4.546480	-1.799419	0.821876
14	6	0	1.411501	0.270871	-0.289347
15	6	0	1.511496	-0.995765	-0.892441
16	6	0	2.454583	0.709781	0.540216
17	6	0	2.621278	-1.809830	-0.657981
18	1	0	0.717505	-1.355687	-1.542531
19	6	0	3.565415	-0.108327	0.776193
20	1	0	2.390788	1.686493	1.008280
21	6	0	3.652899	-1.368411	0.178919
22	1	0	2.679952	-2.788128	-1.126643
23	1	0	4.361121	0.241933	1.427910
24	1	0	4.516237	-2.001637	0.361569
25	1	0	-4.434027	-0.744062	-1.427797

12_(P_III)_method_A_DCM_smd.log

Input orientation:

Center Number	Atomic Number	Atomic Type	Coordinates (Angstroms) X	Y	Z
1	15	0	-0.012700	1.369144	-0.708096
2	8	0	0.106539	2.450447	0.585108
3	1	0	-0.020238	3.357851	0.267026
4	6	0	-1.439655	0.322088	-0.172380
5	6	0	-1.528466	-0.255590	1.106943
6	6	0	-2.488364	0.124246	-1.083706
7	6	0	-2.645863	-1.011722	1.464144
8	1	0	-0.724755	-0.114362	1.824458
9	6	0	-3.603736	-0.645064	-0.730434
10	1	0	-2.435108	0.572295	-2.073079
11	6	0	-3.683755	-1.211249	0.543950
12	1	0	-2.706976	-1.450536	2.456455
13	1	0	-4.550016	-1.805040	0.822765
14	6	0	1.410912	0.269283	-0.286372
15	6	0	1.515853	-0.986864	-0.910763
16	6	0	2.450673	0.698125	0.552771
17	6	0	2.627847	-1.801257	-0.686733
18	1	0	0.724516	-1.336421	-1.570065
19	6	0	3.563809	-0.120419	0.777672
20	1	0	2.385283	1.667302	1.036920
21	6	0	3.656457	-1.370380	0.159763
22	1	0	2.691138	-2.771407	-1.172217
23	1	0	4.357394	0.222119	1.436615
24	1	0	4.521845	-2.003854	0.333739
25	1	0	-4.406697	-0.795235	-1.446892

12_(P_III)_method_B.log

Input orientation:

Center Number	Atomic Number	Atomic Type	Coordinates (Angstroms) X	Y	Z
1	15	0	-0.007684	1.382828	-0.698078
2	8	0	0.124817	2.438160	0.587804
3	1	0	-0.088978	3.330939	0.305863
4	6	0	-1.421297	0.331685	-0.166259
5	6	0	-1.523964	-0.214174	1.117323
6	6	0	-2.446022	0.096849	-1.083168
7	6	0	-2.627583	-0.976071	1.471112
8	1	0	-0.737840	-0.039002	1.839633
9	6	0	-3.548453	-0.679189	-0.734722
10	1	0	-2.381633	0.525810	-2.075436
11	6	0	-3.639877	-1.213666	0.543179
12	1	0	-2.699415	-1.391018	2.467845
13	1	0	-4.497087	-1.812950	0.820060
14	6	0	1.401348	0.280740	-0.288976
15	6	0	1.497371	-0.962891	-0.922373
16	6	0	2.435366	0.684775	0.556380
17	6	0	2.592196	-1.786992	-0.704614
18	1	0	0.707510	-1.295712	-1.584555
19	6	0	3.532138	-0.143542	0.775693
20	1	0	2.375686	1.643705	1.050440
21	6	0	3.614864	-1.379322	0.147289
22	1	0	2.647415	-2.748243	-1.198137
23	1	0	4.322126	0.180418	1.440533
24	1	0	4.468528	-2.021407	0.317877
25	1	0	-4.333594	-0.858229	-1.457120

12_(P_III)_method_B_DCM_smd.log

Input orientation:

Center Number	Atomic Number	Atomic Type	Coordinates (Angstroms) X	Y	Z
1	15	0	-0.013095	1.363708	-0.689780
2	8	0	0.092075	2.436497	0.578281
3	1	0	-0.013898	3.340803	0.260270
4	6	0	-1.431624	0.318182	-0.165083
5	6	0	-1.525347	-0.261863	1.105185
6	6	0	-2.472267	0.124247	-1.075020
7	6	0	-2.637906	-1.015131	1.454175
8	1	0	-0.727415	-0.124679	1.823410
9	6	0	-3.583852	-0.641461	-0.730931
10	1	0	-2.414361	0.575161	-2.057885
11	6	0	-3.667287	-1.209602	0.534058
12	1	0	-2.702293	-1.456835	2.440067
13	1	0	-4.531075	-1.801949	0.806243
14	6	0	1.403880	0.271087	-0.276281
15	6	0	1.501210	-0.983207	-0.890412
16	6	0	2.448648	0.699157	0.545103
17	6	0	2.607067	-1.794736	-0.673646
18	1	0	0.706233	-1.333373	-1.537630
19	6	0	3.556429	-0.116034	0.763272
20	1	0	2.394017	1.667620	1.021960
21	6	0	3.639616	-1.363486	0.155992
22	1	0	2.663069	-2.764166	-1.151477
23	1	0	4.353986	0.227189	1.409625
24	1	0	4.501262	-1.995606	0.325697
25	1	0	-4.381135	-0.788025	-1.447600

12_(P_III)_method_B_DKM.log

Input orientation:

Center Number	Atomic Number	Atomic Type	Coordinates (Angstroms) X	Y	Z
1	15	0	-0.009711	1.373852	-0.692000
2	8	0	0.105780	2.436928	0.585552
3	1	0	-0.056706	3.336889	0.287485
4	6	0	-1.425516	0.322183	-0.170326
5	6	0	-1.516284	-0.261799	1.098135
6	6	0	-2.467669	0.128186	-1.078159
7	6	0	-2.625842	-1.018548	1.447419
8	1	0	-0.716832	-0.123898	1.814128
9	6	0	-3.576483	-0.641776	-0.734738
10	1	0	-2.412871	0.582096	-2.059619
11	6	0	-3.656499	-1.212975	0.528811
12	1	0	-2.687750	-1.462512	2.432110
13	1	0	-4.517932	-1.807995	0.801241
14	6	0	1.402927	0.277189	-0.277573
15	6	0	1.490282	-0.982903	-0.880566
16	6	0	2.453311	0.705246	0.536312
17	6	0	2.591847	-1.799331	-0.661939
18	1	0	0.690049	-1.335088	-1.519717
19	6	0	3.556612	-0.114921	0.757258
20	1	0	2.404001	1.677668	1.004760
21	6	0	3.629904	-1.367678	0.160219
22	1	0	2.639820	-2.773058	-1.131041

23	1	0	4.358281	0.228228	1.397943
24	1	0	4.487736	-2.003787	0.331919
25	1	0	-4.374517	-0.788698	-1.450020

12_(P_III)_method_B_DMSO.log

Input orientation:

Center Number	Atomic Number	Atomic Type	Coordinates (Angstroms) X	Y	Z
1	15	0	-0.011005	1.370604	-0.694112
2	8	0	0.096942	2.437133	0.580787
3	1	0	-0.035193	3.339649	0.274787
4	6	0	-1.426868	0.319646	-0.172175
5	6	0	-1.513719	-0.271298	1.093519
6	6	0	-2.473809	0.133671	-1.076370
7	6	0	-2.624272	-1.026353	1.444053
8	1	0	-0.710614	-0.140823	1.806903
9	6	0	-3.583676	-0.634410	-0.731821
10	1	0	-2.422149	0.591964	-2.055896
11	6	0	-3.659893	-1.212255	0.529107
12	1	0	-2.683002	-1.475593	2.426509
13	1	0	-4.521978	-1.805942	0.802208
14	6	0	1.402440	0.275666	-0.276565
15	6	0	1.491852	-0.985469	-0.877483
16	6	0	2.452457	0.706928	0.536319
17	6	0	2.594825	-1.799899	-0.657290
18	1	0	0.692633	-1.339903	-1.516679
19	6	0	3.557232	-0.111092	0.758807
20	1	0	2.402544	1.680529	1.002372
21	6	0	3.632347	-1.365072	0.164162
22	1	0	2.644416	-2.774317	-1.124744
23	1	0	4.358536	0.234649	1.398515
24	1	0	4.491167	-1.999499	0.336983
25	1	0	-4.385277	-0.775015	-1.444305

12_(P_III)_method_B_DMSO_smd.log

Input orientation:

Center Number	Atomic Number	Atomic Type	Coordinates (Angstroms) X	Y	Z
1	15	0	-0.011186	1.355659	-0.705183
2	8	0	0.091848	2.447962	0.547990
3	1	0	-0.041073	3.343759	0.217359
4	6	0	-1.427946	0.311735	-0.174184
5	6	0	-1.502761	-0.298395	1.083259
6	6	0	-2.489156	0.151153	-1.066968
7	6	0	-2.617104	-1.046845	1.437012
8	1	0	-0.688984	-0.190791	1.788750
9	6	0	-3.602722	-0.609343	-0.718217
10	1	0	-2.446095	0.622850	-2.040773
11	6	0	-3.667692	-1.206470	0.534445
12	1	0	-2.666446	-1.511972	2.412986
13	1	0	-4.532663	-1.795344	0.810491
14	6	0	1.406027	0.271319	-0.276240
15	6	0	1.502959	-0.989400	-0.877431
16	6	0	2.449708	0.706715	0.542516
17	6	0	2.607524	-1.799850	-0.650477
18	1	0	0.709450	-1.344895	-1.523579
19	6	0	3.556085	-0.107706	0.771063
20	1	0	2.397066	1.680170	1.009174
21	6	0	3.638871	-1.361464	0.176959
22	1	0	2.663707	-2.773808	-1.119003
23	1	0	4.352957	0.241374	1.415112
24	1	0	4.499396	-1.992938	0.354442
25	1	0	-4.415832	-0.729975	-1.421908

12_(P_III)_method_B_MeOH.log

Input orientation:

Center Number	Atomic Number	Atomic Type	Coordinates (Angstroms) X	Y	Z
1	15	0	-0.010830	1.370998	-0.693818
2	8	0	0.098075	2.437108	0.581390
3	1	0	-0.038147	3.339323	0.276374
4	6	0	-1.426689	0.319940	-0.171950
5	6	0	-1.514015	-0.270206	1.094062
6	6	0	-2.473061	0.133048	-1.076592
7	6	0	-2.624461	-1.025458	1.444422
8	1	0	-0.711344	-0.138879	1.807768
9	6	0	-3.582817	-0.635249	-0.732200
10	1	0	-2.421021	0.590843	-2.056338
11	6	0	-3.659496	-1.212333	0.529029
12	1	0	-2.683577	-1.474095	2.427135
13	1	0	-4.521514	-1.806171	0.802033
14	6	0	1.402524	0.275861	-0.276658
15	6	0	1.491634	-0.985186	-0.877759
16	6	0	2.452640	0.706764	0.536264
17	6	0	2.594436	-1.799864	-0.657770
18	1	0	0.692251	-1.339375	-1.516883
19	6	0	3.557236	-0.111523	0.758554
20	1	0	2.402847	1.680250	1.002555
21	6	0	3.632076	-1.365393	0.163687
22	1	0	2.643788	-2.774227	-1.125370
23	1	0	4.358623	0.233926	1.398319
24	1	0	4.490773	-2.000029	0.336364
25	1	0	-4.383996	-0.776574	-1.445025

12_(P_III)_method_B_MeOH_smd.log

Input orientation:

Center Number	Atomic Number	Atomic Type	Coordinates (Angstroms) X	Y	Z
1	15	0	-0.012005	1.383832	-0.667597
2	8	0	0.088921	2.429597	0.624167
3	1	0	-0.031251	3.341021	0.333200
4	6	0	-1.428741	0.326801	-0.162546
5	6	0	-1.536073	-0.238457	1.113302
6	6	0	-2.451542	0.109316	-1.086965
7	6	0	-2.644848	-1.001134	1.453648
8	1	0	-0.751891	-0.082580	1.843184
9	6	0	-3.558611	-0.666751	-0.751278
10	1	0	-2.384305	0.547987	-2.074900
11	6	0	-3.656468	-1.219744	0.519432
12	1	0	-2.720534	-1.430376	2.444443
13	1	0	-4.517644	-1.818986	0.785375
14	6	0	1.402695	0.282068	-0.269799
15	6	0	1.477647	-0.979618	-0.871779
16	6	0	2.466081	0.708979	0.528245

17	6	0	2.579511	-1.799428	-0.665113
18	1	0	0.668745	-1.329388	-1.501732
19	6	0	3.569183	-0.114934	0.737106
20	1	0	2.433752	1.684032	0.994061
21	6	0	3.629660	-1.370036	0.142943
22	1	0	2.617750	-2.774857	-1.132749
23	1	0	4.380976	0.227763	1.365981
24	1	0	4.487339	-2.009491	0.305896
25	1	0	-4.342413	-0.832118	-1.478931

12_(P_III)_method_B_THF.log

Input orientation:

Center Number	Atomic Number	Atomic Type	Coordinates (Angstroms) X	Y	Z
1	15	0	-0.009496	1.374528	-0.691655
2	8	0	0.107412	2.436888	0.586477
3	1	0	-0.060203	3.336353	0.289936
4	6	0	-1.425278	0.322746	-0.169943
5	6	0	-1.516862	-0.259644	1.099150
6	6	0	-2.466397	0.126911	-1.078523
7	6	0	-2.626175	-1.016769	1.448241
8	1	0	-0.718200	-0.120059	1.815678
9	6	0	-3.574946	-0.643476	-0.735271
10	1	0	-2.410948	0.579802	-2.060430
11	6	0	-3.655752	-1.213152	0.528878
12	1	0	-2.688743	-1.459521	2.433443
13	1	0	-4.517014	-1.808476	0.801215
14	6	0	1.402958	0.277481	-0.277854
15	6	0	1.490040	-0.982276	-0.881498
16	6	0	2.453262	0.704771	0.536488
17	6	0	2.591310	-1.799109	-0.663165
18	1	0	0.689719	-1.333901	-1.520845
19	6	0	3.556258	-0.115830	0.757137
20	1	0	2.403953	1.676863	1.005596
21	6	0	3.629326	-1.368216	0.159382
22	1	0	2.639074	-2.772602	-1.132783
23	1	0	4.357888	0.226703	1.398208
24	1	0	4.486957	-2.004663	0.330866
25	1	0	-4.372205	-0.791852	-1.451132

12_(P_III)_method_B_THF_smd.log

Input orientation:

Center Number	Atomic Number	Atomic Type	Coordinates (Angstroms) X	Y	Z
1	15	0	-0.009999	1.358992	-0.700319
2	8	0	0.102619	2.444582	0.558478
3	1	0	-0.058945	3.338992	0.239093
4	6	0	-1.427940	0.315282	-0.169670
5	6	0	-1.511988	-0.279882	1.094152
6	6	0	-2.479707	0.138842	-1.070405
7	6	0	-2.625464	-1.030145	1.445768
8	1	0	-0.705481	-0.158179	1.805509
9	6	0	-3.592322	-0.623928	-0.723834
10	1	0	-2.429409	0.600502	-2.048732
11	6	0	-3.666062	-1.206597	0.534940
12	1	0	-2.682120	-1.483459	2.426883
13	1	0	-4.530525	-1.796883	0.809620
14	6	0	1.406622	0.271626	-0.277230
15	6	0	1.503295	-0.985628	-0.885130
16	6	0	2.449999	0.701985	0.544410
17	6	0	2.607408	-1.797554	-0.662674
18	1	0	0.709069	-1.337531	-1.532315
19	6	0	3.555838	-0.114023	0.768615
20	1	0	2.395934	1.672581	1.016680
21	6	0	3.638534	-1.364147	0.167311
22	1	0	2.663283	-2.769009	-1.136369
23	1	0	4.352517	0.230973	1.415093
24	1	0	4.498871	-1.996811	0.341422
25	1	0	-4.398093	-0.757082	-1.433706

12_(P_III)_method_B_toluene.log

Input orientation:

Center Number	Atomic Number	Atomic Type	Coordinates (Angstroms) X	Y	Z
1	15	0	-0.008249	1.380696	-0.690271
2	8	0	0.118969	2.436689	0.593463
3	1	0	-0.080163	3.332716	0.308210
4	6	0	-1.423437	0.328300	-0.166566
5	6	0	-1.522872	-0.235816	1.109773
6	6	0	-2.454100	0.111836	-1.081886
7	6	0	-2.629333	-0.996540	1.458638
8	1	0	-0.732251	-0.077646	1.831177
9	6	0	-3.559531	-0.662692	-0.738625
10	1	0	-2.392501	0.552726	-2.068990
11	6	0	-3.647935	-1.214805	0.532407
12	1	0	-2.698277	-1.425352	2.449629
13	1	0	-4.507142	-1.813085	0.805042
14	6	0	1.402376	0.279904	-0.281720
15	6	0	1.489907	-0.974102	-0.896358
16	6	0	2.448718	0.697429	0.542221
17	6	0	2.588207	-1.794846	-0.680318
18	1	0	0.691387	-1.318149	-1.542079
19	6	0	3.548732	-0.127324	0.760385
20	1	0	2.397186	1.664571	1.021047
21	6	0	3.622675	-1.373682	0.151112
22	1	0	2.636462	-2.764079	-1.158686
23	1	0	4.347647	0.207407	1.408989
24	1	0	4.478371	-2.013288	0.320749
25	1	0	-4.348909	-0.827370	-1.459751

12_(P_III)_method_B_toluene_smd.log

Input orientation:

Center Number	Atomic Number	Atomic Type	Coordinates (Angstroms) X	Y	Z
1	15	0	-0.008783	1.362334	-0.700520
2	8	0	0.113474	2.441053	0.564862
3	1	0	-0.077419	3.332562	0.257996
4	6	0	-1.426470	0.318437	-0.166586
5	6	0	-1.519282	-0.257445	1.105027
6	6	0	-2.468863	0.122724	-1.073624
7	6	0	-2.631215	-1.008587	1.457317
8	1	0	-0.719998	-0.117912	1.820922
9	6	0	-3.579833	-0.641519	-0.726414
10	1	0	-2.411534	0.571802	-2.057435

11	6	0	-3.661742	-1.205253	0.539848
12	1	0	-2.694926	-1.446714	2.444795
13	1	0	-4.525337	-1.796225	0.815493
14	6	0	1.406806	0.272175	-0.281738
15	6	0	1.507076	-0.978250	-0.901882
16	6	0	2.444701	0.693315	0.550901
17	6	0	2.609204	-1.792457	-0.681704
18	1	0	0.715996	-1.323364	-1.556426
19	6	0	3.548683	-0.125042	0.772612
20	1	0	2.385367	1.658365	1.033413
21	6	0	3.635050	-1.368049	0.158512
22	1	0	2.667763	-2.758885	-1.165022
23	1	0	4.341381	0.212607	1.427645
24	1	0	4.494326	-2.002479	0.330820
25	1	0	-4.378487	-0.789695	-1.441221

12_(P_III)_method_B_water.log

Input orientation:

Center	Atomic	Atomic	Coordinates (Angstroms)		
Number	Number	Type	X	Y	Z
1	15	0	-0.011180	1.370216	-0.694414
2	8	0	0.095822	2.437158	0.580183
3	1	0	-0.032213	3.339963	0.273218
4	6	0	-1.427047	0.319361	-0.172396
5	6	0	-1.513431	-0.272352	1.092991
6	6	0	-2.474544	0.134271	-1.076151
7	6	0	-2.624084	-1.027215	1.443702
8	1	0	-0.709902	-0.142699	1.806058
9	6	0	-3.584514	-0.633601	-0.731439
10	1	0	-2.423256	0.593043	-2.055465
11	6	0	-3.660276	-1.212180	0.529197
12	1	0	-2.682434	-1.477037	2.425911
13	1	0	-4.522423	-1.805721	0.802399
14	6	0	1.402352	0.275472	-0.276485
15	6	0	1.492076	-0.985737	-0.877245
16	6	0	2.452253	0.707076	0.536390
17	6	0	2.595215	-1.799922	-0.656844
18	1	0	0.693030	-1.340403	-1.516531
19	6	0	3.557202	-0.110681	0.759077
20	1	0	2.402206	1.680782	1.002225
21	6	0	3.632603	-1.364757	0.164629
22	1	0	2.645054	-2.774386	-1.124171
23	1	0	4.358412	0.235338	1.398749
24	1	0	4.491543	-1.998977	0.337594
25	1	0	-4.386527	-0.773514	-1.443588

12_(P_III)_method_B_water_smd.log

Input orientation:

Center	Atomic	Atomic	Coordinates (Angstroms)		
Number	Number	Type	X	Y	Z
1	15	0	-0.011103	1.403600	-0.633296
2	8	0	0.090753	2.419498	0.683653
3	1	0	-0.040092	3.335444	0.413131
4	6	0	-1.428116	0.335268	-0.155921
5	6	0	-1.547632	-0.237425	1.115404
6	6	0	-2.435963	0.113838	-1.095391
7	6	0	-2.654051	-1.011515	1.436848
8	1	0	-0.774497	-0.078470	1.855822
9	6	0	-3.540370	-0.673945	-0.778795
10	1	0	-2.359221	0.558093	-2.079672
11	6	0	-3.650745	-1.234181	0.487694
12	1	0	-2.739434	-1.446343	2.423933
13	1	0	-4.509851	-1.841972	0.738696
14	6	0	1.400342	0.291272	-0.253890
15	6	0	1.441004	-0.990320	-0.815112
16	6	0	2.497388	0.733755	0.488709
17	6	0	2.542998	-1.813774	-0.622646
18	1	0	0.605034	-1.352677	-1.400007
19	6	0	3.599541	-0.094020	0.684814
20	1	0	2.492135	1.724195	0.921375
21	6	0	3.626127	-1.368906	0.131285
22	1	0	2.554433	-2.804613	-1.057120
23	1	0	4.436874	0.260721	1.271296
24	1	0	4.482710	-2.011505	0.284407
25	1	0	-4.312326	-0.842517	-1.517625

12_(P_III)_method_C_DCM.log

Input orientation:

Center	Atomic	Atomic	Coordinates (Angstroms)		
Number	Number	Type	X	Y	Z
1	15	0	-0.009622	1.375470	-0.693243
2	8	0	0.108986	2.441519	0.595383
3	1	0	-0.060427	3.339984	0.294625
4	6	0	-1.430969	0.321764	-0.168377
5	6	0	-1.525132	-0.252579	1.103762
6	6	0	-2.467621	0.120261	-1.080364
7	6	0	-2.635029	-1.009213	1.452835
8	1	0	-0.729174	-0.107514	1.822209
9	6	0	-3.576418	-0.649438	-0.735855
10	1	0	-2.409325	0.567501	-2.064673
11	6	0	-3.661300	-1.211972	0.531251
12	1	0	-2.700758	-1.446359	2.440329
13	1	0	-4.522845	-1.806540	0.804046
14	6	0	1.408082	0.274693	-0.278501
15	6	0	1.496297	-0.981948	-0.887583
16	6	0	2.455590	0.701023	0.539390
17	6	0	2.598611	-1.798088	-0.670755
18	1	0	0.697848	-1.331361	-1.530456
19	6	0	3.559273	-0.119463	0.757928
20	1	0	2.404724	1.671407	1.011868
21	6	0	3.634763	-1.369343	0.155198
22	1	0	2.648709	-2.769486	-1.144421
23	1	0	4.359793	0.221292	1.401293
24	1	0	4.493046	-2.005230	0.325140
25	1	0	-4.371163	-0.802880	-1.453438

12_(P_III)_method_C_DCM_smd.log

Input orientation:

Center	Atomic	Atomic	Coordinates (Angstroms)		
Number	Number	Type	X	Y	Z
1	15	0	-0.007876	1.371603	-0.693027
2	8	0	0.107226	2.442635	0.589538
3	1	0	0.001656	3.347393	0.270836
4	6	0	-1.432997	0.326135	-0.163111

5	6	0	-1.529956	-0.239818	1.112685
6	6	0	-2.468256	0.121376	-1.076282
7	6	0	-2.642288	-0.992412	1.464066
8	1	0	-0.735462	-0.092673	1.832707
9	6	0	-3.579173	-0.643904	-0.728544
10	1	0	-2.407318	0.561742	-2.063770
11	6	0	-3.667341	-1.198856	0.541934
12	1	0	-2.710459	-1.423312	2.454531
13	1	0	-4.530842	-1.790305	0.816867
14	6	0	1.411533	0.268125	-0.288231
15	6	0	1.509175	-0.973141	-0.927122
16	6	0	2.449792	0.678447	0.549453
17	6	0	2.612058	-1.791041	-0.718940
18	1	0	0.718224	-1.307143	-1.587737
19	6	0	3.554357	-0.143772	0.758401
20	1	0	2.393590	1.637601	1.044621
21	6	0	3.639310	-1.378841	0.126724
22	1	0	2.670352	-2.750482	-1.216253
23	1	0	4.348363	0.184512	1.416757
24	1	0	4.498614	-2.015936	0.289130
25	1	0	-4.373318	-0.799810	-1.446836

12_(P_III)_method_D_DCM.log

Input orientation:

Center	Atomic	Atomic	Coordinates (Angstroms)		
Number	Number	Type	X	Y	Z
1	15	0	-0.008121	1.396909	-0.751034
2	8	0	0.103053	2.468722	0.553556
3	1	0	-0.044250	3.374129	0.247730
4	6	0	-1.410579	0.327160	-0.215770
5	6	0	-1.389355	-0.407756	0.981679
6	6	0	-2.549317	0.270669	-1.031748
7	6	0	-2.490437	-1.176893	1.356310
8	1	0	-0.508455	-0.380040	1.615785
9	6	0	-3.650659	-0.509184	-0.662570
10	1	0	-2.573911	0.835793	-1.959706
11	6	0	-3.621895	-1.231023	0.532352
12	1	0	-2.467155	-1.739618	2.284821
13	1	0	-4.475545	-1.836026	0.822707
14	6	0	1.399551	0.294955	-0.312995
15	6	0	1.490190	-0.969228	-0.919819
16	6	0	2.425086	0.713744	0.545219
17	6	0	2.580156	-1.801347	-0.663776
18	1	0	0.701396	-1.311500	-1.584859
19	6	0	3.516313	-0.122889	0.803019
20	1	0	2.359284	1.686998	1.019256
21	6	0	3.597786	-1.380116	0.200058
22	1	0	2.634415	-2.778619	-1.134161
23	1	0	4.300628	0.209405	1.476883
24	1	0	4.445557	-2.028217	0.400285
25	1	0	-4.525426	-0.550456	-1.304408

12_(P_III)_method_E_DCM.log

Input orientation:

Center	Atomic	Atomic	Coordinates (Angstroms)		
Number	Number	Type	X	Y	Z
1	15	0	-0.009872	1.452919	-0.562773
2	8	0	0.107617	2.405174	0.781523
3	1	0	-0.052395	3.322210	0.556229
4	6	0	-1.417260	0.368501	-0.138800
5	6	0	-1.546099	-0.242525	1.109043
6	6	0	-2.393310	0.147330	-1.104267
7	6	0	-2.632044	-1.055271	1.382576
8	1	0	-0.789635	-0.082881	1.867090
9	6	0	-3.478160	-0.679429	-0.836778
10	1	0	-2.305538	0.623669	-2.073141
11	6	0	-3.598038	-1.277783	0.406400
12	1	0	-2.726459	-1.524280	2.352912
13	1	0	-4.443553	-1.918124	0.620460
14	6	0	1.379430	0.316359	-0.220100
15	6	0	1.338405	-1.005377	-0.662464
16	6	0	2.541826	0.785137	0.387283
17	6	0	2.430894	-1.841288	-0.493484
18	1	0	0.442391	-1.392795	-1.131485
19	6	0	3.633584	-0.053698	0.561839
20	1	0	2.591926	1.808093	0.735721
21	6	0	3.582001	-1.367681	0.121124
22	1	0	2.379551	-2.866530	-0.835322
23	1	0	4.526097	0.322896	1.043877
24	1	0	4.432884	-2.021739	0.257511
25	1	0	-4.228305	-0.849390	-1.597380

12_(P_III)_method_E_DCM_smd.log

Input orientation:

Center	Atomic	Atomic	Coordinates (Angstroms)		
Number	Number	Type	X	Y	Z
1	15	0	0.016043	1.381673	-0.730561
2	8	0	0.161937	2.451430	0.515503
3	1	0	0.099662	3.353074	0.190836
4	6	0	-1.402736	0.369474	-0.180854
5	6	0	-1.489611	-0.171015	1.103113
6	6	0	-2.435944	0.140817	-1.083411
7	6	0	-2.593210	-0.919320	1.474447
8	1	0	-0.689226	-0.006858	1.814328
9	6	0	-3.538723	-0.621919	-0.716935
10	1	0	-2.379719	0.560830	-2.080507
11	6	0	-3.617951	-1.148715	0.561698
12	1	0	-2.655560	-1.332406	2.472839
13	1	0	-4.477115	-1.739294	0.852816
14	6	0	1.389573	0.247941	-0.321027
15	6	0	1.396844	-1.035586	-0.868457
16	6	0	2.475963	0.659230	0.444441
17	6	0	2.461647	-1.892330	-0.644260
18	1	0	0.559779	-1.373876	-1.468396
19	6	0	3.542771	-0.201522	0.671296
20	1	0	2.486308	1.652039	0.873821
21	6	0	3.539378	-1.476978	0.128280
22	1	0	2.450494	-2.886554	-1.071683
23	1	0	4.377764	0.128945	1.275765
24	1	0	4.370565	-2.146967	0.305611
25	1	0	-4.333782	-0.798541	-1.429465

12_(P_V)_method_A.log

Input orientation:

Center Atomic Atomic Coordinates
(Angstroms)

Number	Number	Type	X	Y	Z
1	15	0	-0.037383	0.297073	-0.095160
2	1	0	0.090013	0.401555	1.316013
3	8	0	-0.141136	1.600117	-0.838164
4	6	0	-1.485845	-0.802770	-0.229089
5	6	0	-1.976744	-1.493508	0.887570
6	6	0	-2.125804	-0.946930	-1.469763
7	6	0	-3.084815	-2.336444	0.762999
8	1	0	-1.505152	-1.368153	1.859510
9	6	0	-3.234049	-1.785956	-1.591075
10	1	0	-1.763994	-0.390768	-2.329765
11	6	0	-3.711671	-2.484159	-0.476677
12	1	0	-3.461990	-2.866949	1.632444
13	1	0	-3.728706	-1.891617	-2.552157
14	1	0	-4.576148	-3.134647	-0.573313
15	6	0	1.432373	-0.676766	-0.583335
16	6	0	1.671059	-1.974688	-0.106860
17	6	0	2.356409	-0.071569	-1.445957
18	6	0	2.826620	-2.658661	-0.487993
19	1	0	0.955500	-2.458756	0.552803
20	6	0	3.510300	-0.761166	-1.829195
21	1	0	2.157818	0.931869	-1.810549
22	6	0	3.746688	-2.051930	-1.349795
23	1	0	3.006987	-3.663817	-0.117971
24	1	0	4.222588	-0.289890	-2.500220
25	1	0	4.644277	-2.586659	-1.647161

12_(P_V)_method_A_DCM.log

Input orientation:

Center Number	Atomic Number	Atomic Type	Coordinates (Angstroms) X	Y	Z
1	15	0	-0.033468	1.161376	-0.797652
2	1	0	0.020949	0.963361	-2.198106
3	8	0	-0.127724	2.613648	-0.378765
4	6	0	1.466386	0.284762	-0.256264
5	6	0	2.342494	-0.265412	-1.203585
6	6	0	1.770873	0.184443	1.112183
7	6	0	3.506753	-0.919160	-0.787986
8	1	0	2.122352	-0.184909	-2.264761
9	6	0	2.935130	-0.465077	1.523273
10	1	0	1.102371	0.611966	1.854352
11	6	0	3.802581	-1.018879	0.573755
12	1	0	4.180348	-1.343770	-1.526167
13	1	0	3.166364	-0.540516	2.581486
14	1	0	4.707718	-1.524579	0.896775
15	6	0	-1.470445	0.175844	-0.254597
16	6	0	-1.537414	-1.208585	-0.479928
17	6	0	-2.537982	0.831083	0.374513
18	6	0	-2.663705	-1.928419	-0.077057
19	1	0	-0.714020	-1.728765	-0.962198
20	6	0	-3.662329	0.105964	0.781350
21	1	0	-2.481796	1.901739	0.544411
22	6	0	-3.726194	-1.271622	0.554795
23	1	0	-2.711212	-2.999022	-0.252286
24	1	0	-4.485437	0.617001	1.271812
25	1	0	-4.600196	-1.834220	0.869613

12_(P_V)_method_A_DCM_smd.log

Input orientation:

Center Number	Atomic Number	Atomic Type	Coordinates (Angstroms) X	Y	Z
1	15	0	-0.017599	1.117867	-0.869259
2	1	0	0.062245	0.841162	-2.253948
3	8	0	-0.107943	2.588260	-0.516204
4	6	0	1.472895	0.271301	-0.252325
5	6	0	2.357782	-0.337455	-1.154775
6	6	0	1.759946	0.251661	1.123255
7	6	0	3.514760	-0.968629	-0.686613
8	1	0	2.148869	-0.318265	-2.221443
9	6	0	2.917164	-0.375976	1.586509
10	1	0	1.083721	0.725708	1.829779
11	6	0	3.794052	-0.988335	0.682502
12	1	0	4.195931	-1.438655	-1.390227
13	1	0	3.135300	-0.388611	2.650586
14	1	0	4.693707	-1.476825	1.046295
15	6	0	-1.473177	0.164371	-0.317803
16	6	0	-1.672757	-1.159371	-0.741299
17	6	0	-2.409257	0.774209	0.528477
18	6	0	-2.798120	-1.867279	-0.314548
19	1	0	-0.954719	-1.638462	-1.402383
20	6	0	-3.533397	0.060869	0.956957
21	1	0	-2.259012	1.802544	0.843628
22	6	0	-3.728010	-1.257997	0.536621
23	1	0	-2.950689	-2.890529	-0.645939
24	1	0	-4.256145	0.536944	1.613561
25	1	0	-4.603150	-1.810258	0.867560

12_(P_V)_method_B.log

Input orientation:

Center Number	Atomic Number	Atomic Type	Coordinates (Angstroms) X	Y	Z
1	15	0	-0.032058	1.222340	-0.683069
2	1	0	0.038052	1.112406	-2.092345
3	8	0	-0.148411	2.600287	-0.151481
4	6	0	1.465424	0.325749	-0.195595
5	6	0	2.174355	-0.451988	-1.110760
6	6	0	1.923393	0.427573	1.120115
7	6	0	3.318569	-1.135397	-0.713766
8	1	0	1.842934	-0.517269	-2.139667
9	6	0	3.067199	-0.252378	1.514284
10	1	0	1.390096	1.051075	1.825207
11	6	0	3.763191	-1.037107	0.599074
12	1	0	3.865319	-1.733929	-1.429789
13	1	0	3.419620	-0.167366	2.533301
14	1	0	4.655954	-1.564064	0.908014
15	6	0	-1.456360	0.178665	-0.255405
16	6	0	-1.535563	-1.167924	-0.616175
17	6	0	-2.507691	0.767740	0.446407
18	6	0	-2.656341	-1.915325	-0.281159
19	1	0	-0.721840	-1.639426	-1.152346
20	6	0	-3.627721	0.015770	0.784109
21	1	0	-2.434631	1.811312	0.720257
22	6	0	-3.702995	-1.323244	0.420171
23	1	0	-2.713012	-2.958200	-0.562584
24	1	0	-4.440230	0.476463	1.329614
25	1	0	-4.574856	-1.907511	0.682554

12_(P_V)_method_B_DCM_smd.log

Input orientation:

Center Number	Atomic Number	Atomic Type	Coordinates (Angstroms) X	Y	Z
1	15	0	-0.024291	1.099932	-0.874384
2	1	0	0.050096	0.805645	-2.251061
3	8	0	-0.111125	2.552339	-0.546500
4	6	0	1.461302	0.268726	-0.260052
5	6	0	2.350864	-0.324956	-1.156722
6	6	0	1.743782	0.244348	1.109528
7	6	0	3.507352	-0.942669	-0.691145
8	1	0	2.144336	-0.302466	-2.219196
9	6	0	2.899630	-0.370050	1.571279
10	1	0	1.063221	0.705576	1.813692
11	6	0	3.780810	-0.965963	0.671462
12	1	0	4.192850	-1.400320	-1.391810
13	1	0	3.113815	-0.386625	2.631551
14	1	0	4.680494	-1.445333	1.034085
15	6	0	-1.467606	0.156051	-0.314864
16	6	0	-1.660578	-1.168944	-0.714642
17	6	0	-2.403676	0.770051	0.517017
18	6	0	-2.777566	-1.871407	-0.281659
19	1	0	-0.942934	-1.652946	-1.365279
20	6	0	-3.519528	0.062550	0.952796
21	1	0	-2.258600	1.799047	0.816542
22	6	0	-3.706577	-1.256379	0.554438
23	1	0	-2.925205	-2.896019	-0.596184
24	1	0	-4.242217	0.543098	1.598777
25	1	0	-4.576252	-1.805036	0.891297

12_(P_V)_method_B_DKM.log

Input orientation:

Center Number	Atomic Number	Atomic Type	Coordinates (Angstroms) X	Y	Z
1	15	0	-0.029990	1.149688	-0.804815
2	1	0	0.032175	0.933773	-2.197465
3	8	0	-0.119703	2.583022	-0.404306
4	6	0	1.458126	0.285268	-0.250159
5	6	0	2.316238	-0.308681	-1.176521
6	6	0	1.771666	0.232080	1.111870
7	6	0	3.471485	-0.954861	-0.748174
8	1	0	2.088198	-0.265572	-2.233631
9	6	0	2.926282	-0.409745	1.536607
10	1	0	1.116719	0.693994	1.838975
11	6	0	3.775893	-1.005157	0.606805
12	1	0	4.132336	-1.412192	-1.471652
13	1	0	3.164680	-0.447066	2.590725
14	1	0	4.675017	-1.505182	0.940493
15	6	0	-1.462798	0.174402	-0.274826
16	6	0	-1.565300	-1.187242	-0.572287
17	6	0	-2.487737	0.806124	0.429206
18	6	0	-2.682249	-1.906382	-0.168879
19	1	0	-0.775590	-1.690135	-1.115671
20	6	0	-3.603549	0.082048	0.836547
21	1	0	-2.406497	1.860520	0.653237
22	6	0	-3.701277	-1.272044	0.537615
23	1	0	-2.757596	-2.959671	-0.402114
24	1	0	-4.394634	0.575879	1.384153
25	1	0	-4.569501	-1.834617	0.853231

12_(P_V)_method_B_DMSO.log

Input orientation:

Center Number	Atomic Number	Atomic Type	Coordinates (Angstroms) X	Y	Z
1	15	0	-0.031392	1.101375	-0.880346
2	1	0	0.028168	0.822961	-2.261085
3	8	0	-0.112322	2.554610	-0.547806
4	6	0	1.454564	0.262684	-0.282995
5	6	0	2.380055	-0.259707	-1.187946
6	6	0	1.703071	0.165273	1.090140
7	6	0	3.539275	-0.875815	-0.727345
8	1	0	2.199374	-0.187439	-2.252465
9	6	0	2.861511	-0.446935	1.547156
10	1	0	0.992990	0.565315	1.802096
11	6	0	3.779488	-0.969058	0.638383
12	1	0	4.251535	-1.279031	-1.433968
13	1	0	3.048684	-0.520224	2.609642
14	1	0	4.680619	-1.447699	0.996978
15	6	0	-1.466343	0.159555	-0.302386
16	6	0	-1.615236	-1.194304	-0.616393
17	6	0	-2.441598	0.802944	0.459399
18	6	0	-2.728169	-1.894305	-0.170317
19	1	0	-0.866831	-1.705431	-1.208605
20	6	0	-3.553316	0.097766	0.909105
21	1	0	-2.326879	1.851435	0.696011
22	6	0	-3.696813	-1.248820	0.594822
23	1	0	-2.840374	-2.940984	-0.417777
24	1	0	-4.305570	0.600428	1.501529
25	1	0	-4.562093	-1.796344	0.943138

12_(P_V)_method_B_DMSO_smd.log

Input orientation:

Center Number	Atomic Number	Atomic Type	Coordinates (Angstroms) X	Y	Z
1	15	0	-0.031075	1.062246	-0.944337
2	1	0	0.029338	0.705889	-2.306180
3	8	0	-0.102940	2.530122	-0.685698
4	6	0	1.450065	0.245580	-0.300793
5	6	0	2.416496	-0.237850	-1.184517
6	6	0	1.658385	0.133372	1.077630
7	6	0	3.578879	-0.827008	-0.697260
8	1	0	2.262438	-0.155124	-2.252952
9	6	0	2.819730	-0.453374	1.561269
10	1	0	0.913745	0.500167	1.772818
11	6	0	3.780237	-0.934507	0.674067
12	1	0	4.323664	-1.200216	-1.387541
13	1	0	2.975880	-0.539236	2.628385
14	1	0	4.683718	-1.393219	1.053700
15	6	0	-1.469192	0.149147	-0.325761
16	6	0	-1.669398	-1.187508	-0.680401
17	6	0	-2.389073	0.788692	0.504910
18	6	0	-2.777796	-1.875980	-0.205406
19	1	0	-0.963303	-1.690787	-1.329093
20	6	0	-3.496490	0.095200	0.983162
21	1	0	-2.238666	1.825758	0.772183

22	6	0	-3.691043	-1.235155	0.628769
23	1	0	-2.930723	-2.909733	-0.485411
24	1	0	-4.206488	0.594821	1.628782
25	1	0	-4.553992	-1.773044	0.998641

12_(P_V)_method_B_MeOH.log

Input orientation:

Center	Atomic	Atomic	Coordinates (Angstroms)		
Number	Number	Type	X	Y	Z
1	15	0	-0.031047	1.122480	-0.847210
2	1	0	0.031901	0.871358	-2.233155
3	8	0	-0.116937	2.567767	-0.484192
4	6	0	1.456511	0.275366	-0.266055
5	6	0	2.344194	-0.296077	-1.179013
6	6	0	1.741746	0.215422	1.102185
7	6	0	3.501447	-0.925332	-0.731189
8	1	0	2.136524	-0.250170	-2.240089
9	6	0	2.898396	-0.409594	1.546218
10	1	0	1.062512	0.656460	1.820033
11	6	0	3.778036	-0.981760	0.629583
12	1	0	4.184744	-1.365926	-1.444075
13	1	0	3.114274	-0.453334	2.604898
14	1	0	4.678122	-1.469641	0.978240
15	6	0	-1.465612	0.165503	-0.292670
16	6	0	-1.599707	-1.187877	-0.615292
17	6	0	-2.456636	0.797621	0.458060
18	6	0	-2.713727	-1.898476	-0.189123
19	1	0	-0.838506	-1.690469	-1.198293
20	6	0	-3.569489	0.081935	0.887929
21	1	0	-2.353231	1.845806	0.701224
22	6	0	-3.698298	-1.264108	0.564845
23	1	0	-2.814215	-2.944810	-0.443107
24	1	0	-4.334131	0.576002	1.471668
25	1	0	-4.564474	-1.819896	0.897544

12_(P_V)_method_B_MeOH_smd.log

Input orientation:

Center	Atomic	Atomic	Coordinates (Angstroms)		
Number	Number	Type	X	Y	Z
1	15	0	-0.028852	1.087412	-0.886142
2	1	0	0.036417	0.815236	-2.264410
3	8	0	-0.109123	2.548777	-0.553506
4	6	0	1.457503	0.267449	-0.273704
5	6	0	2.355925	-0.310711	-1.171746
6	6	0	1.728562	0.231635	1.097947
7	6	0	3.513899	-0.923032	-0.703242
8	1	0	2.154801	-0.282364	-2.235025
9	6	0	2.886757	-0.376713	1.561085
10	1	0	1.037187	0.676820	1.802060
11	6	0	3.778843	-0.955657	0.660916
12	1	0	4.207232	-1.369816	-1.403331
13	1	0	3.093631	-0.402444	2.622736
14	1	0	4.680297	-1.430780	1.025201
15	6	0	-1.469783	0.157324	-0.310786
16	6	0	-1.644956	-1.177830	-0.685215
17	6	0	-2.415745	0.777599	0.505177
18	6	0	-2.756103	-1.883692	-0.244085
19	1	0	-0.917145	-1.666478	-1.320943
20	6	0	-3.525938	0.065780	0.948187
21	1	0	-2.286610	1.812344	0.791634
22	6	0	-3.696411	-1.262468	0.574579
23	1	0	-2.889526	-2.916283	-0.538281
24	1	0	-4.256906	0.550449	1.581747
25	1	0	-4.561556	-1.814305	0.918112

12_(P_V)_method_B_THF.log

Input orientation:

Center	Atomic	Atomic	Coordinates (Angstroms)		
Number	Number	Type	X	Y	Z
1	15	0	-0.028782	1.154292	-0.797597
2	1	0	0.033492	0.944129	-2.191330
3	8	0	-0.118463	2.585414	-0.390770
4	6	0	1.458760	0.285960	-0.247166
5	6	0	2.314020	-0.308427	-1.175813
6	6	0	1.774576	0.230057	1.114168
7	6	0	3.468481	-0.957975	-0.750476
8	1	0	2.084513	-0.262828	-2.232524
9	6	0	2.928371	-0.415127	1.535968
10	1	0	1.122092	0.692907	1.842870
11	6	0	3.775001	-1.011145	0.603887
12	1	0	4.127165	-1.415533	-1.475803
13	1	0	3.168575	-0.454386	2.589613
14	1	0	4.673573	-1.513689	0.935295
15	6	0	-1.461962	0.176669	-0.272284
16	6	0	-1.560974	-1.185788	-0.567007
17	6	0	-2.491316	0.808489	0.425180
18	6	0	-2.678902	-1.905644	-0.167628
19	1	0	-0.767527	-1.688872	-1.104750
20	6	0	-3.608110	0.083720	0.828517
21	1	0	-2.412306	1.863486	0.647201
22	6	0	-3.702407	-1.271157	0.532170
23	1	0	-2.751461	-2.959645	-0.398566
24	1	0	-4.402634	0.577632	1.371064
25	1	0	-4.571380	-1.834288	0.844749

12_(P_V)_method_B_THF_smd.log

Input orientation:

Center	Atomic	Atomic	Coordinates (Angstroms)		
Number	Number	Type	X	Y	Z
1	15	0	-0.035960	1.076338	-0.905229
2	1	0	0.015681	0.754662	-2.277634
3	8	0	-0.110795	2.535937	-0.613901
4	6	0	1.451652	0.243535	-0.297605
5	6	0	2.413778	-0.210101	-1.201354
6	6	0	1.673198	0.097089	1.075326
7	6	0	3.583670	-0.804994	-0.740160
8	1	0	2.251472	-0.097994	-2.265954
9	6	0	2.841777	-0.495275	1.533391
10	1	0	0.933749	0.443899	1.785926
11	6	0	3.797240	-0.947244	0.625925
12	1	0	4.325319	-1.154030	-1.446244
13	1	0	3.008211	-0.606673	2.596550
14	1	0	4.707027	-1.409355	0.985836
15	6	0	-1.470827	0.149227	-0.296543

16	6	0	-1.612241	-1.215838	-0.559385
17	6	0	-2.456244	0.817664	0.429382
18	6	0	-2.727339	-1.902974	-0.098849
19	1	0	-0.853207	-1.744584	-1.122238
20	6	0	-3.570485	0.125841	0.893940
21	1	0	-2.348197	1.875538	0.625908
22	6	0	-3.706539	-1.232244	0.630134
23	1	0	-2.832928	-2.959479	-0.306447
24	1	0	-4.331323	0.648528	1.458244
25	1	0	-4.574295	-1.769224	0.989946

12_(P_V)_method_B_toluene.log

Input orientation:

Center Number	Atomic Number	Atomic Type	Coordinates (Angstroms) X	Y	Z
1	15	0	-0.033783	1.178807	-0.761807
2	1	0	0.024639	0.993589	-2.161332
3	8	0	-0.132445	2.595493	-0.322936
4	6	0	1.458516	0.298456	-0.238930
5	6	0	2.283873	-0.320970	-1.177509
6	6	0	1.805286	0.257187	1.114815
7	6	0	3.437605	-0.982881	-0.769886
8	1	0	2.032678	-0.283391	-2.229826
9	6	0	2.957885	-0.400615	1.519509
10	1	0	1.178154	0.745308	1.849087
11	6	0	3.773526	-1.023131	0.577668
12	1	0	4.073529	-1.459065	-1.503654
13	1	0	3.222460	-0.427321	2.567779
14	1	0	4.671983	-1.534604	0.895716
15	6	0	-1.459878	0.178327	-0.254745
16	6	0	-1.529919	-1.191581	-0.519471
17	6	0	-2.517975	0.809755	0.398589
18	6	0	-2.648228	-1.919540	-0.136365
19	1	0	-0.711601	-1.694673	-1.018624
20	6	0	-3.635237	0.077211	0.785505
21	1	0	-2.456703	1.870332	0.599083
22	6	0	-3.701170	-1.285038	0.517597
23	1	0	-2.697816	-2.979995	-0.343061
24	1	0	-4.452349	0.570831	1.293846
25	1	0	-4.570633	-1.854239	0.817917

12_(P_V)_method_B_toluene_smd.log

Input orientation:

Center Number	Atomic Number	Atomic Type	Coordinates (Angstroms) X	Y	Z
1	15	0	-0.030975	1.150055	-0.805414
2	1	0	0.034816	0.914772	-2.196819
3	8	0	-0.126368	2.580866	-0.414703
4	6	0	1.459488	0.284956	-0.247955
5	6	0	2.319142	-0.306972	-1.173461
6	6	0	1.774341	0.232849	1.113165
7	6	0	3.474957	-0.952226	-0.746065
8	1	0	2.091546	-0.260724	-2.231032
9	6	0	2.929184	-0.408216	1.537561
10	1	0	1.119696	0.697055	1.839258
11	6	0	3.779076	-1.003221	0.608490
12	1	0	4.137189	-1.407637	-1.470089
13	1	0	3.168222	-0.444137	2.591946
14	1	0	4.679052	-1.502431	0.942300
15	6	0	-1.463557	0.169379	-0.276042
16	6	0	-1.573179	-1.190180	-0.577845
17	6	0	-2.483462	0.803239	0.432721
18	6	0	-2.691853	-1.905922	-0.173996
19	1	0	-0.785678	-1.693838	-1.124437
20	6	0	-3.600865	0.082537	0.840726
21	1	0	-2.395329	1.857317	0.657520
22	6	0	-3.705601	-1.269681	0.537601
23	1	0	-2.772984	-2.958438	-0.410502
24	1	0	-4.388639	0.578268	1.392074
25	1	0	-4.575821	-1.829419	0.853960

12_(P_V)_method_B_water.log

Input orientation:

Center Number	Atomic Number	Atomic Type	Coordinates (Angstroms) X	Y	Z
1	15	0	-0.030834	1.124888	-0.842546
2	1	0	0.033082	0.878780	-2.229076
3	8	0	-0.117396	2.569331	-0.474527
4	6	0	1.456961	0.277467	-0.262754
5	6	0	2.336681	-0.305591	-1.176074
6	6	0	1.749700	0.227622	1.104322
7	6	0	3.493607	-0.936430	-0.729574
8	1	0	2.123252	-0.267249	-2.236291
9	6	0	2.906042	-0.399080	1.546933
10	1	0	1.076701	0.677754	1.822383
11	6	0	3.777794	-0.982850	0.630064
12	1	0	4.170815	-1.385999	-1.442636
13	1	0	3.127847	-0.435028	2.604672
14	1	0	4.677691	-1.471824	0.977625
15	6	0	-1.465559	0.166065	-0.292035
16	6	0	-1.599119	-1.186119	-0.619685
17	6	0	-2.457061	0.795319	0.460501
18	6	0	-2.713372	-1.898390	-0.196849
19	1	0	-0.837498	-1.686270	-1.204174
20	6	0	-3.570260	0.078016	0.886772
21	1	0	-2.354106	1.842590	0.707717
22	6	0	-3.698655	-1.266865	0.558615
23	1	0	-2.813457	-2.943789	-0.454733
24	1	0	-4.335438	0.569829	1.471685
25	1	0	-4.565022	-1.823925	0.888629

12_(P_V)_method_B_water_smd.log

Input orientation:

Center Number	Atomic Number	Atomic Type	Coordinates (Angstroms) X	Y	Z
1	15	0	-0.028426	1.091720	-0.887454
2	1	0	0.039059	0.815312	-2.263873
3	8	0	-0.109097	2.554660	-0.556748
4	6	0	1.455839	0.271237	-0.272807
5	6	0	2.350914	-0.311579	-1.170979
6	6	0	1.726700	0.235356	1.098776
7	6	0	3.506537	-0.928172	-0.702430
8	1	0	2.147964	-0.283423	-2.233567
9	6	0	2.882638	-0.377169	1.561883

10	1	0	1.036477	0.682649	1.802146
11	6	0	3.771919	-0.960408	0.661652
12	1	0	4.197241	-1.378740	-1.402010
13	1	0	3.089690	-0.403396	2.623044
14	1	0	4.671127	-1.438842	1.025850
15	6	0	-1.467920	0.161854	-0.310802
16	6	0	-1.639506	-1.173075	-0.687318
17	6	0	-2.414464	0.778577	0.506860
18	6	0	-2.748368	-1.882543	-0.246521
19	1	0	-0.910150	-1.657423	-1.324039
20	6	0	-3.522421	0.063139	0.949528
21	1	0	-2.287712	1.812797	0.794964
22	6	0	-3.689636	-1.264929	0.573787
23	1	0	-2.879316	-2.914581	-0.542124
24	1	0	-4.253917	0.544515	1.584233
25	1	0	-4.552774	-1.819285	0.916914

12_(P_V)_method_C_DCM.log

Input orientation:

Center	Atomic	Atomic	Coordinates (Angstroms)		
Number	Number	Type	X	Y	Z
1	15	0	-0.008006	1.306999	-0.481753
2	1	0	0.089498	1.432396	-1.887253
3	8	0	-0.101140	2.615008	0.253012
4	6	0	1.466851	0.315980	-0.109683
5	6	0	2.401211	0.042603	-1.109037
6	6	0	1.690561	-0.145637	1.190928
7	6	0	3.546615	-0.689893	-0.813608
8	1	0	2.240039	0.401147	-2.117391
9	6	0	2.835263	-0.873677	1.483110
10	1	0	0.973297	0.063098	1.973815
11	6	0	3.763048	-1.147362	0.480637
12	1	0	4.267583	-0.898781	-1.591987
13	1	0	3.004724	-1.229269	2.490170
14	1	0	4.653804	-1.715933	0.710823
15	6	0	-1.465014	0.246834	-0.232383
16	6	0	-1.583910	-0.985217	-0.880184
17	6	0	-2.484003	0.687614	0.610810
18	6	0	-2.713747	-1.767839	-0.683304
19	1	0	-0.798807	-1.337739	-1.536949
20	6	0	-3.612669	-0.101245	0.809353
21	1	0	-2.389094	1.644498	1.104793
22	6	0	-3.727850	-1.326792	0.163441
23	1	0	-2.803487	-2.720309	-1.187464
24	1	0	-4.400268	0.242504	1.465914
25	1	0	-4.606206	-1.938868	0.317250

12_(P_V)_method_C_DCM_smd.log

Input orientation:

Center	Atomic	Atomic	Coordinates (Angstroms)		
Number	Number	Type	X	Y	Z
1	15	0	-0.006997	1.294954	-0.506483
2	1	0	0.096022	1.393162	-1.912736
3	8	0	-0.099749	2.614537	0.208564
4	6	0	1.468320	0.311976	-0.110473
5	6	0	2.402203	0.020814	-1.105234
6	6	0	1.693521	-0.122149	1.199127
7	6	0	3.550068	-0.702252	-0.795505
8	1	0	2.237800	0.359690	-2.120261
9	6	0	2.840717	-0.841199	1.505220
10	1	0	0.974947	0.100465	1.977417
11	6	0	3.768874	-1.132626	0.507907
12	1	0	4.271637	-0.924555	-1.570244
13	1	0	3.011656	-1.175803	2.519698
14	1	0	4.662000	-1.693653	0.749345
15	6	0	-1.468753	0.241530	-0.247163
16	6	0	-1.619393	-0.964171	-0.936397
17	6	0	-2.456434	0.658879	0.643917
18	6	0	-2.749044	-1.745533	-0.731511
19	1	0	-0.858336	-1.294620	-1.632201
20	6	0	-3.585219	-0.128341	0.849670
21	1	0	-2.340283	1.597080	1.169178
22	6	0	-3.731706	-1.328666	0.163463
23	1	0	-2.863778	-2.677938	-1.268049
24	1	0	-4.349160	0.197768	1.542871
25	1	0	-4.610622	-1.939227	0.322941

12_(P_V)_method_D_DCM.log

Input orientation:

Center	Atomic	Atomic	Coordinates (Angstroms)		
Number	Number	Type	X	Y	Z
1	15	0	-0.020014	1.322573	-0.568109
2	1	0	0.056999	1.399797	-1.978527
3	8	0	-0.116443	2.671059	0.113616
4	6	0	1.447269	0.331671	-0.176784
5	6	0	2.455465	0.154693	-1.134171
6	6	0	1.590997	-0.232827	1.100909
7	6	0	3.597965	-0.585775	-0.819022
8	1	0	2.350906	0.589493	-2.124195
9	6	0	2.733688	-0.967920	1.413323
10	1	0	0.809853	-0.104659	1.844493
11	6	0	3.737020	-1.145213	0.453402
12	1	0	4.375211	-0.723682	-1.563936
13	1	0	2.841782	-1.405078	2.400891
14	1	0	4.624451	-1.720454	0.698418
15	6	0	-1.447795	0.250068	-0.229690
16	6	0	-1.465154	-1.087043	-0.654469
17	6	0	-2.548830	0.782587	0.452412
18	6	0	-2.581066	-1.884050	-0.398200
19	1	0	-0.609186	-1.507538	-1.174601
20	6	0	-3.662478	-0.020773	0.712506
21	1	0	-2.523407	1.816796	0.780100
22	6	0	-3.679598	-1.351202	0.285970
23	1	0	-2.592785	-2.918773	-0.725995
24	1	0	-4.513319	0.391699	1.245663
25	1	0	-4.545279	-1.974544	0.487658

12_(P_V)_method_E_DCM.log

Input orientation:

Center	Atomic	Atomic	Coordinates (Angstroms)		
Number	Number	Type	X	Y	Z
1	15	0	-0.036183	1.161622	-0.870183
2	1	0	0.006428	0.911526	-2.255678
3	8	0	-0.114489	2.598513	-0.508380

4	6	0	1.432147	0.289309	-0.303532
5	6	0	2.357444	-0.206659	-1.215389
6	6	0	1.649063	0.119217	1.063094
7	6	0	3.492134	-0.867944	-0.766477
8	1	0	2.195485	-0.078885	-2.278359
9	6	0	2.782712	-0.536922	1.509071
10	1	0	0.928697	0.495659	1.778753
11	6	0	3.703971	-1.031378	0.593378
12	1	0	4.209202	-1.252211	-1.478914
13	1	0	2.948976	-0.667967	2.569454
14	1	0	4.589116	-1.545203	0.943656
15	6	0	-1.441976	0.197116	-0.283876
16	6	0	-1.498203	-1.178979	-0.493467
17	6	0	-2.478062	0.839423	0.383683
18	6	0	-2.586842	-1.904769	-0.041382
19	1	0	-0.689303	-1.686882	-1.004918
20	6	0	-3.565634	0.108580	0.841486
21	1	0	-2.425726	1.907916	0.544487
22	6	0	-3.620186	-1.260178	0.627931
23	1	0	-2.629602	-2.972807	-0.205196
24	1	0	-4.368640	0.609478	1.364931
25	1	0	-4.468133	-1.829323	0.984791

12_(P_V)_method_E_DCM_smd.log

Input orientation:

Center Number	Atomic Number	Atomic Type	Coordinates (Angstroms) X	Y	Z
1	15	0	-0.011014	1.350856	-0.443258
2	1	0	0.098302	1.524466	-1.835977
3	8	0	-0.113975	2.613421	0.330474
4	6	0	1.439049	0.342091	-0.094705
5	6	0	2.420673	0.165705	-1.064165
6	6	0	1.590616	-0.247469	1.159471
7	6	0	3.547680	-0.594381	-0.783138
8	1	0	2.306445	0.621739	-2.040073
9	6	0	2.716883	-1.002104	1.438845
10	1	0	0.825491	-0.120515	1.915897
11	6	0	3.695114	-1.175987	0.466695
12	1	0	4.309351	-0.729123	-1.539596
13	1	0	2.833260	-1.458746	2.412618
14	1	0	4.574793	-1.766607	0.687041
15	6	0	-1.439891	0.267407	-0.242067
16	6	0	-1.506367	-0.951344	-0.913784
17	6	0	-2.481401	0.656867	0.591776
18	6	0	-2.609680	-1.772252	-0.752390
19	1	0	-0.695259	-1.262293	-1.561558
20	6	0	-3.584391	-0.170169	0.755893
21	1	0	-2.424639	1.605359	1.109628
22	6	0	-3.648396	-1.381742	0.084566
23	1	0	-2.660180	-2.718476	-1.274454
24	1	0	-4.391912	0.133595	1.408790
25	1	0	-4.508607	-2.025877	0.212384

13_(P_III)_method_A.log

Input orientation:

Center Number	Atomic Number	Atomic Type	Coordinates (Angstroms) X	Y	Z
1	15	0	0.066153	1.403746	-0.418200
2	8	0	-1.346661	0.563628	-0.629791
3	6	0	-1.671983	-0.652199	0.084424
4	1	0	-1.574422	-0.470294	1.160419
5	1	0	-0.958276	-1.434604	-0.198102
6	6	0	-3.092353	-1.048640	-0.278523
7	1	0	-3.185718	-1.218391	-1.355624
8	1	0	-3.366534	-1.972758	0.242748
9	1	0	-3.800628	-0.265576	0.008878
10	6	0	1.335441	0.062634	-0.537454
11	6	0	1.476304	-0.590822	-1.773832
12	6	0	2.213255	-0.263476	0.506764
13	6	0	2.457642	-1.566808	-1.955779
14	1	0	0.812331	-0.339618	-2.598190
15	6	0	3.199080	-1.238083	0.322890
16	1	0	2.115332	0.241533	1.461890
17	6	0	3.322703	-1.891666	-0.905759
18	1	0	2.551228	-2.068365	-2.914891
19	1	0	3.870795	-1.485551	1.140361
20	1	0	4.091085	-2.646493	-1.047164
21	8	0	0.170166	1.594133	1.254237
22	1	0	-0.139642	2.473262	1.508623

13_(P_III)_method_A_DCM.log

Input orientation:

Center Number	Atomic Number	Atomic Type	Coordinates (Angstroms) X	Y	Z
1	15	0	0.838024	-0.398978	-1.006154
2	6	0	-0.893333	-0.156844	-0.399265
3	6	0	-1.607172	0.972522	-0.836950
4	6	0	-1.545466	-1.087935	0.425856
5	6	0	-2.928912	1.184064	-0.433513
6	1	0	-1.134598	1.689241	-1.504642
7	6	0	-2.871061	-0.883269	0.819900
8	1	0	-1.013737	-1.976074	0.751675
9	6	0	-3.564649	0.255440	0.396807
10	1	0	-3.464193	2.064406	-0.778045
11	1	0	-3.362093	-1.613369	1.457575
12	1	0	-4.594950	0.412603	0.702862
13	8	0	1.092109	-2.007368	-0.540354
14	1	0	1.262819	-2.553558	-1.319280
15	6	0	1.889666	0.456913	0.329031
16	6	0	3.359244	0.128927	-0.007748
17	1	0	4.022512	0.640086	0.701222
18	1	0	3.556665	-0.945336	0.063144
19	1	0	3.627969	0.462337	-1.016681
20	6	0	1.561467	-0.021053	1.752768
21	1	0	2.241801	0.459688	2.467864
22	1	0	0.537792	0.237020	2.040791
23	1	0	1.683555	-1.104186	1.850578
24	6	0	1.662748	1.976055	0.209918
25	1	0	1.878564	2.342950	-0.800501
26	1	0	0.636588	2.258450	0.465570
27	1	0	2.332619	2.500560	0.902564

13_(P_III)_method_A_DCM_smd.log

Input orientation:

Center Atomic Atomic Coordinates (Angstroms)

Number	Number	Type	X	Y	Z
1	15	0	0.833641	-0.397183	-0.996648
2	6	0	-0.899440	-0.160005	-0.392666
3	6	0	-1.608785	0.977695	-0.816952
4	6	0	-1.558068	-1.103408	0.413440
5	6	0	-2.932790	1.184656	-0.418204
6	1	0	-1.129537	1.704519	-1.468977
7	6	0	-2.885963	-0.902441	0.803024
8	1	0	-1.031480	-1.998400	0.729590
9	6	0	-3.575247	0.244020	0.393760
10	1	0	-3.464639	2.071686	-0.751925
11	1	0	-3.381827	-1.642141	1.426328
12	1	0	-4.607471	0.398155	0.696194
13	8	0	1.086537	-2.006638	-0.538704
14	1	0	1.284673	-2.543671	-1.320639
15	6	0	1.892362	0.461813	0.330510
16	6	0	3.357734	0.118925	-0.007075
17	1	0	4.027245	0.635261	0.692800
18	1	0	3.551092	-0.955635	0.077341
19	1	0	3.626262	0.436749	-1.021644
20	6	0	1.563306	-0.000204	1.758485
21	1	0	2.251355	0.479625	2.467446
22	1	0	0.543828	0.272248	2.049652
23	1	0	1.674723	-1.083771	1.869359
24	6	0	1.678943	1.981193	0.198137
25	1	0	1.888315	2.336734	-0.818161
26	1	0	0.658256	2.277490	0.461202
27	1	0	2.360956	2.506019	0.879320

13_(P_III)_method_B.log

Input orientation:

Center Number	Atomic Number	Atomic Type	Coordinates (Angstroms) X	Y	Z
1	15	0	-0.836063	-0.395604	-1.001212
2	6	0	0.884585	-0.159386	-0.397404
3	6	0	1.595337	0.961957	-0.836360
4	6	0	1.534289	-1.080592	0.428171
5	6	0	2.907808	1.177579	-0.433294
6	1	0	1.123792	1.667747	-1.509284
7	6	0	2.851123	-0.872807	0.821481
8	1	0	1.006684	-1.965251	0.755653
9	6	0	3.539278	0.259154	0.398041
10	1	0	3.440845	2.053052	-0.779875
11	1	0	3.340870	-1.596450	1.460008
12	1	0	4.564317	0.418945	0.704844
13	8	0	-1.100053	-1.983376	-0.550037
14	1	0	-1.253062	-2.526967	-1.326132
15	6	0	-1.875986	0.453860	0.330210
16	6	0	-3.341425	0.128302	-0.005883
17	1	0	-3.538613	-0.940664	0.071016
18	1	0	-4.003282	0.642667	0.695007
19	1	0	-3.604435	0.453150	-1.013972
20	6	0	-1.550841	-0.023557	1.749634
21	1	0	-0.533622	0.237080	2.039741
22	1	0	-2.231942	0.450058	2.462109
23	1	0	-1.668075	-1.102678	1.844850
24	6	0	-1.649507	1.968023	0.212557
25	1	0	-0.627952	2.247536	0.469215
26	1	0	-1.861132	2.331376	-0.794977
27	1	0	-2.317852	2.492002	0.899890

13_(P_III)_method_B_DCM_smd.log

Input orientation:

Center Number	Atomic Number	Atomic Type	Coordinates (Angstroms) X	Y	Z
1	15	0	-0.832851	-0.399371	-0.985624
2	6	0	0.891986	-0.162720	-0.390954
3	6	0	1.599536	0.958007	-0.839116
4	6	0	1.547755	-1.080413	0.434968
5	6	0	2.915749	1.175008	-0.446211
6	1	0	1.122679	1.664000	-1.508154
7	6	0	2.867991	-0.870631	0.819289
8	1	0	1.026308	-1.964544	0.774218
9	6	0	3.553786	0.259798	0.385303
10	1	0	3.445718	2.049744	-0.800078
11	1	0	3.361381	-1.591352	1.458754
12	1	0	4.581232	0.421067	0.684261
13	8	0	-1.092402	-1.988443	-0.554753
14	1	0	-1.273691	-2.521210	-1.337038
15	6	0	-1.880595	0.452667	0.335708
16	6	0	-3.343154	0.131280	-0.014900
17	1	0	-3.547371	-0.937789	0.050817
18	1	0	-4.008748	0.641806	0.685498
19	1	0	-3.599082	0.464950	-1.022427
20	6	0	-1.570478	-0.024770	1.757868
21	1	0	-0.556702	0.236302	2.061258
22	1	0	-2.259098	0.450939	2.461886
23	1	0	-1.690970	-1.103614	1.858253
24	6	0	-1.651321	1.966269	0.221451
25	1	0	-0.634771	2.247946	0.496292
26	1	0	-1.845247	2.330558	-0.789542
27	1	0	-2.332554	2.489670	0.896970

13_(P_III)_method_B_DKM.log

Input orientation:

Center Number	Atomic Number	Atomic Type	Coordinates (Angstroms) X	Y	Z
1	15	0	-0.835281	-0.403359	-0.993761
2	6	0	0.886760	-0.163601	-0.393257
3	6	0	1.591539	0.965625	-0.823559
4	6	0	1.543484	-1.089616	0.421977
5	6	0	2.905645	1.181936	-0.424059
6	1	0	1.115211	1.679148	-1.484646
7	6	0	2.861881	-0.881129	0.812321
8	1	0	1.021928	-1.978945	0.746095
9	6	0	3.544751	0.257402	0.395930
10	1	0	3.433312	2.063269	-0.763679
11	1	0	3.356198	-1.608717	1.442680
12	1	0	4.570451	0.418080	0.699807
13	8	0	-1.096196	-1.989603	-0.549144
14	1	0	-1.268122	-2.530722	-1.324673
15	6	0	-1.877458	0.453844	0.329543
16	6	0	-3.342883	0.127521	-0.006890
17	1	0	-3.542030	-0.940934	0.072467
18	1	0	-4.003409	0.645278	0.692407
19	1	0	-3.605064	0.450297	-1.015835
20	6	0	-1.554463	-0.015151	1.752382
21	1	0	-0.535794	0.242643	2.040265

22	1	0	-2.233300	0.468103	2.460103
23	1	0	-1.677541	-1.092844	1.856830
24	6	0	-1.651237	1.967737	0.204746
25	1	0	-0.630897	2.249763	0.463290
26	1	0	-1.859602	2.326234	-0.805051
27	1	0	-2.322801	2.492896	0.887709

13_(P_III)_method_B_DMSO.log

Input orientation:

Center Number	Atomic Number	Atomic Type	Coordinates (Angstroms) X	Y	Z
1	15	0	-0.834603	-0.405323	-0.992125
2	6	0	0.887537	-0.165392	-0.392174
3	6	0	1.590292	0.966775	-0.818628
4	6	0	1.546557	-1.093430	0.419123
5	6	0	2.904605	1.183500	-0.419547
6	1	0	1.112410	1.682829	-1.475802
7	6	0	2.865150	-0.884500	0.809120
8	1	0	1.027111	-1.984842	0.740870
9	6	0	3.546044	0.256703	0.396317
10	1	0	3.430388	2.067126	-0.756035
11	1	0	3.361030	-1.613700	1.436350
12	1	0	4.571753	0.417801	0.699883
13	8	0	-1.093967	-1.991509	-0.548525
14	1	0	-1.280945	-2.529829	-1.322783
15	6	0	-1.877249	0.454238	0.329009
16	6	0	-3.342470	0.126279	-0.006899
17	1	0	-3.541102	-0.942212	0.073534
18	1	0	-4.003007	0.644559	0.691920
19	1	0	-3.604998	0.447756	-1.016177
20	6	0	-1.554065	-0.011449	1.752877
21	1	0	-0.535560	0.247674	2.040292
22	1	0	-2.233314	0.473005	2.459288
23	1	0	-1.676492	-1.089003	1.859912
24	6	0	-1.652327	1.968127	0.201200
25	1	0	-0.632445	2.251835	0.459714
26	1	0	-1.860291	2.324415	-0.809436
27	1	0	-2.324958	2.493722	0.882721

13_(P_III)_method_B_DMSO_smd.log

Input orientation:

Center Number	Atomic Number	Atomic Type	Coordinates (Angstroms) X	Y	Z
1	15	0	-0.832759	-0.404737	-0.986808
2	6	0	0.890174	-0.167673	-0.389778
3	6	0	1.590363	0.967119	-0.813854
4	6	0	1.551245	-1.097927	0.417423
5	6	0	2.905502	1.183047	-0.416951
6	1	0	1.109128	1.685906	-1.465871
7	6	0	2.870655	-0.888923	0.805301
8	1	0	1.035360	-1.991645	0.739332
9	6	0	3.549678	0.254085	0.394481
10	1	0	3.429839	2.068806	-0.751452
11	1	0	3.368409	-1.619969	1.429559
12	1	0	4.576331	0.415078	0.696403
13	8	0	-1.089467	-1.995262	-0.556794
14	1	0	-1.294653	-2.520752	-1.337466
15	6	0	-1.877643	0.454110	0.330805
16	6	0	-3.340945	0.123458	-0.005719
17	1	0	-3.544062	-0.944079	0.083614
18	1	0	-4.002889	0.648580	0.687295
19	1	0	-3.602703	0.436176	-1.018558
20	6	0	-1.555207	-0.005168	1.755740
21	1	0	-0.538844	0.260015	2.047114
22	1	0	-2.238275	0.480037	2.458702
23	1	0	-1.674590	-1.082760	1.870990
24	6	0	-1.656143	1.967292	0.198424
25	1	0	-0.640305	2.257446	0.467128
26	1	0	-1.854574	2.319877	-0.815946
27	1	0	-2.338542	2.493019	0.870885

13_(P_III)_method_B_MeOH.log

Input orientation:

Center Number	Atomic Number	Atomic Type	Coordinates (Angstroms) X	Y	Z
1	15	0	-0.834692	-0.405053	-0.992321
2	6	0	0.887437	-0.165161	-0.392313
3	6	0	1.590473	0.966588	-0.819347
4	6	0	1.546152	-1.092887	0.419567
5	6	0	2.904767	1.183272	-0.420226
6	1	0	1.112800	1.682281	-1.477072
7	6	0	2.864724	-0.884007	0.809594
8	1	0	1.026431	-1.984003	0.741697
9	6	0	3.545892	0.256817	0.396241
10	1	0	3.430803	2.066573	-0.757182
11	1	0	3.360392	-1.612959	1.437284
12	1	0	4.571603	0.417865	0.699837
13	8	0	-1.094268	-1.991263	-0.548687
14	1	0	-1.279199	-2.529924	-1.323173
15	6	0	-1.877284	0.454168	0.329095
16	6	0	-3.342537	0.126467	-0.006905
17	1	0	-3.541274	-0.942017	0.073364
18	1	0	-4.003062	0.644687	0.691981
19	1	0	-3.604999	0.448142	-1.016136
20	6	0	-1.554161	-0.011980	1.752828
21	1	0	-0.535634	0.246942	2.040329
22	1	0	-2.233358	0.472322	2.459405
23	1	0	-1.676698	-1.089553	1.859513
24	6	0	-1.652169	1.968061	0.201723
25	1	0	-0.632225	2.251528	0.460264
26	1	0	-1.860164	2.324666	-0.808798
27	1	0	-2.324666	2.493585	0.883437

13_(P_III)_method_B_MeOH_smd.log

Input orientation:

Center Number	Atomic Number	Atomic Type	Coordinates (Angstroms) X	Y	Z
1	15	0	-0.831585	-0.392814	-0.991813
2	6	0	0.891501	-0.164232	-0.391083
3	6	0	1.599839	0.958374	-0.833636
4	6	0	1.545250	-1.083300	0.435078
5	6	0	2.915106	1.174443	-0.436988
6	1	0	1.124766	1.667349	-1.500985
7	6	0	2.864861	-0.874766	0.822389

8	1	0	1.025840	-1.968263	0.775681
9	6	0	3.551657	0.256711	0.392800
10	1	0	3.445402	2.050995	-0.786421
11	1	0	3.356639	-1.597092	1.461545
12	1	0	4.578535	0.417192	0.694709
13	8	0	-1.093250	-1.988591	-0.582617
14	1	0	-1.297927	-2.507638	-1.368408
15	6	0	-1.878149	0.451716	0.334398
16	6	0	-3.341248	0.122198	-0.005740
17	1	0	-3.545229	-0.945958	0.077157
18	1	0	-4.004450	0.642785	0.689609
19	1	0	-3.602407	0.440527	-1.017048
20	6	0	-1.557243	-0.016200	1.757190
21	1	0	-0.541312	0.247103	2.051719
22	1	0	-2.241056	0.465110	2.462249
23	1	0	-1.677452	-1.094376	1.868989
24	6	0	-1.657118	1.966079	0.212668
25	1	0	-0.640396	2.254148	0.480945
26	1	0	-1.857821	2.326045	-0.798747
27	1	0	-2.337669	2.487611	0.890358

13_(P_III)_method_B_THF.log

Input orientation:

Center Number	Atomic Number	Atomic Type	Coordinates (Angstroms) X	Y	Z
1	15	0	-0.835382	-0.402929	-0.994129
2	6	0	0.886608	-0.163281	-0.393495
3	6	0	1.591791	0.965349	-0.824581
4	6	0	1.542871	-1.088854	0.422574
5	6	0	2.905840	1.181621	-0.424958
6	1	0	1.115774	1.678320	-1.486501
7	6	0	2.861210	-0.880423	0.813015
8	1	0	1.020907	-1.977781	0.747158
9	6	0	3.544464	0.257581	0.395902
10	1	0	3.433876	2.062494	-0.765216
11	1	0	3.355217	-1.607661	1.444028
12	1	0	4.570150	0.418200	0.699876
13	8	0	-1.096526	-1.989233	-0.549328
14	1	0	-1.266170	-2.530707	-1.325052
15	6	0	-1.877450	0.453762	0.329641
16	6	0	-3.342909	0.127736	-0.006887
17	1	0	-3.542126	-0.940723	0.072216
18	1	0	-4.003450	0.645337	0.692531
19	1	0	-3.605040	0.450796	-1.015756
20	6	0	-1.554481	-0.015897	1.752266
21	1	0	-0.535815	0.241728	2.040287
22	1	0	-2.233302	0.467007	2.460265
23	1	0	-1.677575	-1.093638	1.856151
24	6	0	-1.651003	1.967662	0.205457
25	1	0	-0.630582	2.249371	0.464030
26	1	0	-1.859431	2.326588	-0.804181
27	1	0	-2.322382	2.492731	0.888687

13_(P_III)_method_B_THF_smd.log

Input orientation:

Center Number	Atomic Number	Atomic Type	Coordinates (Angstroms) X	Y	Z
1	15	0	-0.833014	-0.399381	-0.987602
2	6	0	0.890444	-0.163858	-0.390224
3	6	0	1.595205	0.964637	-0.822797
4	6	0	1.548045	-1.091079	0.423082
5	6	0	2.910775	1.178890	-0.426896
6	1	0	1.116925	1.678890	-1.482008
7	6	0	2.867878	-0.884131	0.809751
8	1	0	1.027989	-1.980654	0.749713
9	6	0	3.550987	0.253533	0.391407
10	1	0	3.438821	2.059864	-0.768086
11	1	0	3.363024	-1.612787	1.438834
12	1	0	4.578146	0.412849	0.692440
13	8	0	-1.093432	-1.989597	-0.555889
14	1	0	-1.270715	-2.521008	-1.338643
15	6	0	-1.879675	0.454447	0.332930
16	6	0	-3.342590	0.124679	-0.007516
17	1	0	-3.544700	-0.943286	0.077797
18	1	0	-4.006923	0.646602	0.685713
19	1	0	-3.602441	0.440520	-1.019817
20	6	0	-1.559572	-0.010836	1.756738
21	1	0	-0.544093	0.253867	2.051145
22	1	0	-2.244201	0.469768	2.461436
23	1	0	-1.677769	-1.089072	1.866135
24	6	0	-1.657507	1.968194	0.206329
25	1	0	-0.641298	2.256412	0.475623
26	1	0	-1.856724	2.324690	-0.806539
27	1	0	-2.338501	2.493002	0.880944

13_(P_III)_method_B_toluene.log

Input orientation:

Center Number	Atomic Number	Atomic Type	Coordinates (Angstroms) X	Y	Z
1	15	0	-0.835964	-0.399161	-0.997266
2	6	0	0.885494	-0.160917	-0.395431
3	6	0	1.593948	0.963064	-0.832349
4	6	0	1.538230	-1.083328	0.426727
5	6	0	2.907405	1.179068	-0.431495
6	1	0	1.120400	1.671579	-1.500905
7	6	0	2.855961	-0.875252	0.818169
8	1	0	1.013066	-1.969356	0.754280
9	6	0	3.542139	0.258822	0.396008
10	1	0	3.438336	2.056444	-0.776428
11	1	0	3.347596	-1.599928	1.454034
12	1	0	4.567648	0.418981	0.700993
13	8	0	-1.099207	-1.986055	-0.550416
14	1	0	-1.253032	-2.529935	-1.327111
15	6	0	-1.877140	0.453412	0.330347
16	6	0	-3.342742	0.129042	-0.006767
17	1	0	-3.541955	-0.939533	0.070699
18	1	0	-4.003745	0.645324	0.693374
19	1	0	-3.604560	0.453767	-1.015196
20	6	0	-1.554213	-0.021273	1.751282
21	1	0	-0.536024	0.236369	2.040833
22	1	0	-2.233796	0.457651	2.461481
23	1	0	-1.675927	-1.099592	1.850374
24	6	0	-1.649650	1.967467	0.210819
25	1	0	-0.628766	2.247222	0.469776
26	1	0	-1.858499	2.329477	-0.797704
27	1	0	-2.319918	2.491795	0.895870

13_(P_III)_method_B_toluene_smd.log

Input orientation:

Center	Atomic	Atomic	Coordinates (Angstroms)		
Number	Number	Type	X	Y	Z
1	15	0	-0.833684	-0.393544	-0.991241
2	6	0	0.889568	-0.159280	-0.392361
3	6	0	1.599981	0.960418	-0.836965
4	6	0	1.541933	-1.080077	0.431911
5	6	0	2.915165	1.174140	-0.440959
6	1	0	1.125993	1.666840	-1.507640
7	6	0	2.861357	-0.874012	0.818354
8	1	0	1.016442	-1.963768	0.765582
9	6	0	3.549471	0.255942	0.388912
10	1	0	3.447881	2.048365	-0.791754
11	1	0	3.352749	-1.597563	1.456027
12	1	0	4.576607	0.414412	0.690064
13	8	0	-1.096856	-1.984049	-0.556049
14	1	0	-1.250012	-2.520344	-1.339047
15	6	0	-1.880371	0.453617	0.335042
16	6	0	-3.343136	0.127575	-0.009840
17	1	0	-3.545463	-0.940731	0.068647
18	1	0	-4.010042	0.644758	0.684543
19	1	0	-3.601106	0.449099	-1.020718
20	6	0	-1.563196	-0.021665	1.756261
21	1	0	-0.548414	0.240338	2.054842
22	1	0	-2.248427	0.452924	2.464558
23	1	0	-1.681374	-1.100565	1.857003
24	6	0	-1.654897	1.967564	0.217808
25	1	0	-0.637995	2.251774	0.488534
26	1	0	-1.853622	2.330997	-0.792683
27	1	0	-2.333468	2.491993	0.895166

13_(P_III)_method_B_water.log

Input orientation:

Center	Atomic	Atomic	Coordinates (Angstroms)		
Number	Number	Type	X	Y	Z
1	15	0	-0.834513	-0.405588	-0.991937
2	6	0	0.887635	-0.165620	-0.392040
3	6	0	1.590111	0.966963	-0.817914
4	6	0	1.546956	-1.093970	0.418676
5	6	0	2.904441	1.183727	-0.418867
6	1	0	1.112022	1.683372	-1.474543
7	6	0	2.865568	-0.884989	0.808648
8	1	0	1.027781	-1.985677	0.740035
9	6	0	3.546189	0.256592	0.396398
10	1	0	3.429974	2.067676	-0.754886
11	1	0	3.361657	-1.614437	1.435421
12	1	0	4.571895	0.417739	0.699939
13	8	0	-1.093663	-1.991753	-0.548358
14	1	0	-1.282701	-2.529730	-1.322378
15	6	0	-1.877210	0.454309	0.328922
16	6	0	-3.342400	0.126091	-0.006892
17	1	0	-3.540924	-0.942410	0.073705
18	1	0	-4.002952	0.644428	0.691861
19	1	0	-3.604995	0.447368	-1.016216
20	6	0	-1.553963	-0.010920	1.752922
21	1	0	-0.535481	0.248406	2.040251
22	1	0	-2.233264	0.473677	2.459172
23	1	0	-1.676272	-1.088457	1.860299
24	6	0	-1.652484	1.968193	0.200678
25	1	0	-0.632663	2.252141	0.459168
26	1	0	-1.860415	2.324167	-0.810073
27	1	0	-2.325247	2.493861	0.882005

13_(P_III)_method_B_water_smd.log

Input orientation:

Center	Atomic	Atomic	Coordinates (Angstroms)		
Number	Number	Type	X	Y	Z
1	15	0	-0.832633	-0.399393	-0.996177
2	6	0	0.888912	-0.168136	-0.393249
3	6	0	1.592902	0.960918	-0.826018
4	6	0	1.544196	-1.089256	0.429144
5	6	0	2.906324	1.179804	-0.425107
6	1	0	1.115346	1.673141	-1.487508
7	6	0	2.862050	-0.877780	0.820763
8	1	0	1.026954	-1.977317	0.763881
9	6	0	3.545094	0.259115	0.399714
10	1	0	3.433178	2.060930	-0.766796
11	1	0	3.355047	-1.601564	1.456572
12	1	0	4.570215	0.421872	0.704774
13	8	0	-1.093034	-1.994678	-0.580785
14	1	0	-1.316167	-2.510604	-1.363221
15	6	0	-1.874042	0.451000	0.329721
16	6	0	-3.338875	0.122630	-0.001961
17	1	0	-3.542567	-0.945066	0.082936
18	1	0	-3.996025	0.644733	0.697383
19	1	0	-3.604568	0.441112	-1.011578
20	6	0	-1.547281	-0.012727	1.752282
21	1	0	-0.529698	0.250314	2.040120
22	1	0	-2.227838	0.472249	2.457312
23	1	0	-1.668297	-1.090185	1.867049
24	6	0	-1.652364	1.964605	0.203283
25	1	0	-0.633702	2.251444	0.464141
26	1	0	-1.859560	2.321715	-0.807358
27	1	0	-2.328483	2.486281	0.884682

13_(P_III)_method_D_DCM.log

Input orientation:

Center	Atomic	Atomic	Coordinates (Angstroms)		
Number	Number	Type	X	Y	Z
1	15	0	0.856175	-0.427188	-1.027723
2	6	0	-0.866524	-0.170375	-0.425217
3	6	0	-1.567065	0.966878	-0.858733
4	6	0	-1.512296	-1.082002	0.423614
5	6	0	-2.874349	1.205528	-0.427434
6	1	0	-1.093059	1.668303	-1.540683
7	6	0	-2.823772	-0.850272	0.845370
8	1	0	-0.983156	-1.971381	0.748491
9	6	0	-3.506146	0.296416	0.426514
10	1	0	-3.401685	2.092170	-0.766738
11	1	0	-3.312392	-1.563617	1.502852
12	1	0	-4.525317	0.475545	0.755490
13	8	0	1.109661	-2.023639	-0.525847
14	1	0	1.289327	-2.585104	-1.291306
15	6	0	1.865688	0.450853	0.312609
16	6	0	3.343633	0.145953	0.007702

17	1	0	3.984192	0.665066	0.730921
18	1	0	3.551969	-0.925786	0.080867
19	1	0	3.625413	0.484836	-0.995436
20	6	0	1.510875	-0.029925	1.725708
21	1	0	2.171247	0.456052	2.454883
22	1	0	0.478702	0.219594	1.986862
23	1	0	1.638599	-1.112036	1.821120
24	6	0	1.610314	1.961377	0.183856
25	1	0	1.824041	2.325710	-0.827533
26	1	0	0.576630	2.219861	0.431569
27	1	0	2.265109	2.501605	0.878012

13_(P_V)_method_A.log

Input orientation:

Center Number	Atomic Number	Atomic Type	Coordinates (Angstroms) X	Y	Z
1	15	0	0.088930	1.087743	-0.002665
2	1	0	0.138583	2.107753	-0.974019
3	8	0	0.154984	1.534196	1.421594
4	8	0	-1.302904	0.414736	-0.514477
5	6	0	-1.923858	-0.644401	0.261213
6	1	0	-2.074507	-0.286978	1.284638
7	1	0	-1.248358	-1.508457	0.288436
8	6	0	-3.241013	-1.000448	-0.402096
9	1	0	-3.081194	-1.350625	-1.426374
10	1	0	-3.735453	-1.797645	0.163429
11	1	0	-3.906087	-0.132413	-0.431300
12	6	0	1.405276	-0.089639	-0.437655
13	6	0	1.455988	-0.689944	-1.706019
14	6	0	2.402429	-0.371897	0.506935
15	6	0	2.494432	-1.566925	-2.023759
16	1	0	0.683603	-0.477465	-2.440590
17	6	0	3.438374	-1.253189	0.186526
18	1	0	2.353119	0.099989	1.483558
19	6	0	3.485426	-1.848372	-1.077031
20	1	0	2.530877	-2.030522	-3.005294
21	1	0	4.208204	-1.472027	0.920714
22	1	0	4.293166	-2.530821	-1.325769

13_(P_V)_method_A_DCM.log

Input orientation:

Center Number	Atomic Number	Atomic Type	Coordinates (Angstroms) X	Y	Z
1	15	0	0.800753	-0.524184	-0.800337
2	1	0	0.943147	0.177752	-2.024515
3	8	0	1.103381	-2.008026	-0.888534
4	6	0	-0.932114	-0.166444	-0.354908
5	6	0	-1.619558	0.914894	-0.927996
6	6	0	-1.597136	-1.004250	0.554841
7	6	0	-2.950255	1.165196	-0.582531
8	1	0	-1.125329	1.559460	-1.650001
9	6	0	-2.927168	-0.751773	0.898770
10	1	0	-1.079816	-1.858526	0.980923
11	6	0	-3.603554	0.333911	0.332669
12	1	0	-3.476839	2.001154	-1.033125
13	1	0	-3.435885	-1.404431	1.601929
14	1	0	-4.639116	0.526384	0.597249
15	6	0	1.947853	0.414489	0.332525
16	6	0	3.380193	0.193544	-0.200620
17	1	0	4.090238	0.727580	0.441589
18	1	0	3.651120	-0.866016	-0.199971
19	1	0	3.499654	0.579240	-1.219565
20	6	0	1.823364	-0.146083	1.762868
21	1	0	2.533066	0.373156	2.417374
22	1	0	0.819952	0.005355	2.173890
23	1	0	2.054261	-1.215191	1.797475
24	6	0	1.602477	1.915644	0.309134
25	1	0	1.656703	2.334408	-0.702096
26	1	0	0.604644	2.114321	0.711630
27	1	0	2.325906	2.458896	0.928194

13_(P_V)_method_A_DCM_smd.log

Input orientation:

Center Number	Atomic Number	Atomic Type	Coordinates (Angstroms) X	Y	Z
1	15	0	0.800052	-0.505236	-0.806695
2	1	0	0.935358	0.224848	-2.013944
3	8	0	1.104313	-1.987076	-0.932647
4	6	0	-0.935450	-0.160578	-0.353338
5	6	0	-1.633465	0.903880	-0.945214
6	6	0	-1.592754	-0.988049	0.571839
7	6	0	-2.966828	1.148329	-0.604227
8	1	0	-1.142248	1.538970	-1.677891
9	6	0	-2.925577	-0.741861	0.910849
10	1	0	-1.068192	-1.828332	1.017262
11	6	0	-3.612727	0.327187	0.325458
12	1	0	-3.500963	1.971854	-1.069739
13	1	0	-3.428129	-1.386634	1.626328
14	1	0	-4.650562	0.514378	0.586778
15	6	0	1.951366	0.412718	0.338659
16	6	0	3.379413	0.187357	-0.201207
17	1	0	4.095152	0.720670	0.436197
18	1	0	3.649788	-0.872941	-0.200980
19	1	0	3.492810	0.570483	-1.222407
20	6	0	1.827673	-0.154319	1.765020
21	1	0	2.546212	0.353852	2.419681
22	1	0	0.827347	0.005036	2.181254
23	1	0	2.048081	-1.226439	1.795435
24	6	0	1.615728	1.915283	0.323740
25	1	0	1.663345	2.335633	-0.687569
26	1	0	0.622168	2.119053	0.735415
27	1	0	2.348029	2.452393	0.938804

13_(P_V)_method_B.log

Input orientation:

Center Number	Atomic Number	Atomic Type	Coordinates (Angstroms) X	Y	Z
1	15	0	0.799924	-0.556961	-0.750689
2	1	0	0.946969	0.094943	-2.001673
3	8	0	1.101167	-2.009956	-0.747614
4	6	0	-0.926255	-0.179099	-0.331393
5	6	0	-1.577289	0.959228	-0.810473
6	6	0	-1.626595	-1.080792	0.472341
7	6	0	-2.903742	1.202649	-0.476226

8	1	0	-1.055817	1.653368	-1.456895
9	6	0	-2.952186	-0.834956	0.808183
10	1	0	-1.131430	-1.980607	0.811057
11	6	0	-3.590201	0.307479	0.337313
12	1	0	-3.402838	2.083745	-0.856464
13	1	0	-3.489416	-1.539010	1.429192
14	1	0	-4.624061	0.495105	0.594612
15	6	0	1.936196	0.437217	0.323698
16	6	0	3.359249	0.167555	-0.199792
17	1	0	4.082272	0.700123	0.421606
18	1	0	3.596272	-0.894659	-0.172304
19	1	0	3.484013	0.517580	-1.226384
20	6	0	1.807190	-0.059723	1.772028
21	1	0	2.526177	0.466168	2.403809
22	1	0	0.811425	0.126764	2.175538
23	1	0	2.011818	-1.127472	1.841707
24	6	0	1.617423	1.935227	0.235161
25	1	0	1.658781	2.301081	-0.792573
26	1	0	0.634448	2.168068	0.643079
27	1	0	2.356449	2.497395	0.810016

13_(P_V)_method_B_DCM_smd.log

Input orientation:

Center Number	Atomic Number	Atomic Type	Coordinates (Angstroms) X	Y	Z
1	15	0	0.796682	-0.501488	-0.793458
2	1	0	0.931669	0.219571	-2.000890
3	8	0	1.098199	-1.960531	-0.908111
4	6	0	-0.928986	-0.161676	-0.346477
5	6	0	-1.617749	0.918025	-0.903844
6	6	0	-1.591300	-1.010124	0.544981
7	6	0	-2.945492	1.154214	-0.565303
8	1	0	-1.122093	1.571074	-1.610624
9	6	0	-2.918069	-0.772210	0.883106
10	1	0	-1.072367	-1.861234	0.964994
11	6	0	-3.595296	0.310639	0.329986
12	1	0	-3.472813	1.989784	-1.005990
13	1	0	-3.424481	-1.434441	1.572711
14	1	0	-4.629822	0.491889	0.590229
15	6	0	1.939522	0.418370	0.333514
16	6	0	3.363470	0.168798	-0.195706
17	1	0	4.079203	0.708917	0.427744
18	1	0	3.623082	-0.888958	-0.171029
19	1	0	3.481553	0.525153	-1.221130
20	6	0	1.805835	-0.122175	1.764633
21	1	0	2.533424	0.376616	2.408668
22	1	0	0.814193	0.065009	2.177731
23	1	0	2.000520	-1.193994	1.810331
24	6	0	1.624736	1.920226	0.293504
25	1	0	1.683808	2.322036	-0.719555
26	1	0	0.635821	2.141654	0.694390
27	1	0	2.356692	2.455314	0.902458

13_(P_V)_method_B_DKM.log

Input orientation:

Center Number	Atomic Number	Atomic Type	Coordinates (Angstroms) X	Y	Z
1	15	0	0.798384	-0.521243	-0.787477
2	1	0	0.942102	0.176579	-2.008492
3	8	0	1.098612	-1.981814	-0.870775
4	6	0	-0.924726	-0.166953	-0.350636
5	6	0	-1.605623	0.916994	-0.909193
6	6	0	-1.591777	-1.008303	0.543632
7	6	0	-2.930406	1.164144	-0.568471
8	1	0	-1.108917	1.565191	-1.619385
9	6	0	-2.915431	-0.758986	0.884444
10	1	0	-1.078369	-1.863692	0.960792
11	6	0	-3.584505	0.328211	0.330645
12	1	0	-3.452312	2.002297	-1.009611
13	1	0	-3.425626	-1.415235	1.576251
14	1	0	-4.616417	0.518708	0.592853
15	6	0	1.935335	0.414991	0.331166
16	6	0	3.362285	0.186706	-0.201663
17	1	0	4.071894	0.722237	0.431720
18	1	0	3.628003	-0.869229	-0.193460
19	1	0	3.477989	0.561614	-1.220053
20	6	0	1.813148	-0.134556	1.761174
21	1	0	2.524546	0.382921	2.407464
22	1	0	0.815804	0.023554	2.171859
23	1	0	2.037484	-1.200075	1.800560
24	6	0	1.598245	1.913014	0.300077
25	1	0	1.650647	2.322259	-0.709975
26	1	0	0.606611	2.116667	0.702417
27	1	0	2.322964	2.454457	0.910997

13_(P_V)_method_B_DMSO.log

Input orientation:

Center Number	Atomic Number	Atomic Type	Coordinates (Angstroms) X	Y	Z
1	15	0	0.798010	-0.513680	-0.792646
2	1	0	0.939343	0.190697	-2.009024
3	8	0	1.099343	-1.975258	-0.889962
4	6	0	-0.924924	-0.165436	-0.352318
5	6	0	-1.610446	0.909857	-0.922174
6	6	0	-1.587235	-0.997824	0.554271
7	6	0	-2.935342	1.156858	-0.581297
8	1	0	-1.116884	1.551588	-1.640248
9	6	0	-2.911091	-0.748869	0.894629
10	1	0	-1.070797	-1.845148	0.984076
11	6	0	-3.584961	0.329442	0.328912
12	1	0	-3.460413	1.988580	-1.030636
13	1	0	-3.417312	-1.397632	1.596291
14	1	0	-4.616749	0.519987	0.591418
15	6	0	1.935744	0.412643	0.332070
16	6	0	3.363030	0.188398	-0.201779
17	1	0	4.071108	0.723264	0.433692
18	1	0	3.631526	-0.866964	-0.196034
19	1	0	3.478166	0.566913	-1.218788
20	6	0	1.813586	-0.144543	1.759142
21	1	0	2.526232	0.368809	2.407161
22	1	0	0.816932	0.012738	2.171591
23	1	0	2.036947	-1.210526	1.793437
24	6	0	1.597770	1.910878	0.308786
25	1	0	1.652517	2.325954	-0.698649
26	1	0	0.605019	2.111539	0.709816
27	1	0	2.320824	2.448194	0.925124

13_(P_V)_method_B_DMSO_smd.log

Input orientation:

Center Number	Atomic Number	Atomic Type	Coordinates (Angstroms) X	Y	Z
1	15	0	0.799776	-0.506537	-0.799809
2	1	0	0.936482	0.227514	-1.998224
3	8	0	1.097371	-1.965670	-0.930835
4	6	0	-0.924762	-0.161291	-0.352367
5	6	0	-1.617732	0.895233	-0.947431
6	6	0	-1.581366	-0.974173	0.575893
7	6	0	-2.944182	1.143076	-0.611641
8	1	0	-1.125423	1.521572	-1.680294
9	6	0	-2.906718	-0.724754	0.911150
10	1	0	-1.060149	-1.806085	1.030135
11	6	0	-3.588444	0.334743	0.319251
12	1	0	-3.474466	1.961063	-1.081008
13	1	0	-3.408175	-1.358664	1.630459
14	1	0	-4.621654	0.525326	0.578251
15	6	0	1.936583	0.404659	0.338667
16	6	0	3.360848	0.195226	-0.204769
17	1	0	4.069255	0.735989	0.426470
18	1	0	3.642297	-0.857443	-0.203338
19	1	0	3.464088	0.575862	-1.222957
20	6	0	1.824878	-0.165615	1.759370
21	1	0	2.539343	0.344899	2.409016
22	1	0	0.829311	-0.016842	2.178682
23	1	0	2.053240	-1.231459	1.785771
24	6	0	1.592580	1.900488	0.331561
25	1	0	1.627785	2.321024	-0.675035
26	1	0	0.605256	2.094811	0.750235
27	1	0	2.323919	2.437506	0.939658

13_(P_V)_method_B_MeOH.log

Input orientation:

Center Number	Atomic Number	Atomic Type	Coordinates (Angstroms) X	Y	Z
1	15	0	0.798050	-0.514445	-0.792162
2	1	0	0.939662	0.189349	-2.008975
3	8	0	1.099233	-1.975907	-0.888098
4	6	0	-0.924888	-0.165558	-0.352159
5	6	0	-1.609949	0.910688	-0.920728
6	6	0	-1.587662	-0.998980	0.553086
7	6	0	-2.934830	1.157656	-0.579822
8	1	0	-1.116087	1.553158	-1.637948
9	6	0	-2.911489	-0.750027	0.893550
10	1	0	-1.071530	-1.847220	0.981433
11	6	0	-3.584884	0.329260	0.329181
12	1	0	-3.459600	1.990072	-1.028242
13	1	0	-3.418108	-1.399656	1.594128
14	1	0	-4.616687	0.519756	0.591682
15	6	0	1.935693	0.412891	0.331961
16	6	0	3.362962	0.188113	-0.201680
17	1	0	4.071192	0.723093	0.433546
18	1	0	3.631120	-0.867318	-0.195553
19	1	0	3.478228	0.566126	-1.218873
20	6	0	1.813417	-0.143396	1.759371
21	1	0	2.525885	0.370423	2.407236
22	1	0	0.816659	0.014011	2.171543
23	1	0	2.036867	-1.209330	1.794290
24	6	0	1.597887	1.911122	0.307741
25	1	0	1.652455	2.325516	-0.699997
26	1	0	0.605240	2.112168	0.708844
27	1	0	2.321105	2.448894	0.923507

13_(P_V)_method_B_MeOH_smd.log

Input orientation:

Center Number	Atomic Number	Atomic Type	Coordinates (Angstroms) X	Y	Z
1	15	0	0.793729	-0.488893	-0.793053
2	1	0	0.938079	0.207628	-2.009125
3	8	0	1.095574	-1.958994	-0.899533
4	6	0	-0.929711	-0.159388	-0.352173
5	6	0	-1.621543	0.904829	-0.935011
6	6	0	-1.584419	-0.987003	0.564333
7	6	0	-2.948294	1.145684	-0.596952
8	1	0	-1.129248	1.541377	-1.658962
9	6	0	-2.910736	-0.744025	0.899740
10	1	0	-1.062295	-1.823586	1.009179
11	6	0	-3.592289	0.322966	0.321617
12	1	0	-3.479288	1.969437	-1.055362
13	1	0	-3.412775	-1.388867	1.608861
14	1	0	-4.626230	0.508565	0.581691
15	6	0	1.939234	0.413472	0.337233
16	6	0	3.361393	0.184425	-0.205467
17	1	0	4.074147	0.720826	0.424324
18	1	0	3.632556	-0.871149	-0.197029
19	1	0	3.470005	0.558616	-1.225352
20	6	0	1.817939	-0.151765	1.759822
21	1	0	2.539058	0.351829	2.407159
22	1	0	0.824169	0.011883	2.177698
23	1	0	2.032650	-1.220524	1.789962
24	6	0	1.609125	1.912528	0.321870
25	1	0	1.656156	2.329683	-0.685622
26	1	0	0.621007	2.117844	0.733443
27	1	0	2.341948	2.443059	0.933568

13_(P_V)_method_B_THF.log

Input orientation:

Center Number	Atomic Number	Atomic Type	Coordinates (Angstroms) X	Y	Z
1	15	0	0.798472	-0.523138	-0.786049
2	1	0	0.942623	0.172865	-2.008303
3	8	0	1.098581	-1.983471	-0.865780
4	6	0	-0.924722	-0.167487	-0.350109
5	6	0	-1.604357	0.918590	-0.906000
6	6	0	-1.593090	-1.010916	0.541104
7	6	0	-2.929125	1.165900	-0.565451
8	1	0	-1.106736	1.568307	-1.614188
9	6	0	-2.916723	-0.761408	0.881853
10	1	0	-1.080521	-1.868196	0.955383
11	6	0	-3.584482	0.327974	0.330881
12	1	0	-3.450121	2.005673	-1.004609
13	1	0	-3.428002	-1.419374	1.571235
14	1	0	-4.616425	0.518570	0.592935
15	6	0	1.935265	0.415599	0.330964
16	6	0	3.362087	0.186467	-0.201807

17	1	0	4.072099	0.722079	0.431096
18	1	0	3.627195	-0.869597	-0.193189
19	1	0	3.477805	0.560693	-1.220469
20	6	0	1.813284	-0.132185	1.761659
21	1	0	2.524474	0.386255	2.407447
22	1	0	0.815834	0.026081	2.172082
23	1	0	2.037869	-1.197591	1.802168
24	6	0	1.598282	1.913540	0.298086
25	1	0	1.649918	2.321405	-0.712588
26	1	0	0.606977	2.117846	0.700917
27	1	0	2.323480	2.455978	0.907593

13_(P_V)_method_B_THF_smd.log

Input orientation:

Center Number	Atomic Number	Atomic Type	Coordinates (Angstroms) X	Y	Z
1	15	0	0.799145	-0.513658	-0.791479
2	1	0	0.936614	0.207493	-1.999359
3	8	0	1.099637	-1.971614	-0.900788
4	6	0	-0.926190	-0.164140	-0.348821
5	6	0	-1.613025	0.908118	-0.922378
6	6	0	-1.589758	-0.995978	0.556974
7	6	0	-2.939479	1.153633	-0.585898
8	1	0	-1.117112	1.548276	-1.640756
9	6	0	-2.915132	-0.748703	0.893403
10	1	0	-1.073172	-1.842368	0.989138
11	6	0	-3.590115	0.327008	0.324209
12	1	0	-3.465326	1.983392	-1.039281
13	1	0	-3.422294	-1.398196	1.594514
14	1	0	-4.623675	0.515577	0.583200
15	6	0	1.937178	0.412253	0.335818
16	6	0	3.360976	0.186633	-0.203235
17	1	0	4.074111	0.724371	0.425340
18	1	0	3.631451	-0.868642	-0.196092
19	1	0	3.470256	0.559931	-1.223599
20	6	0	1.819231	-0.143217	1.762298
21	1	0	2.536307	0.367081	2.409243
22	1	0	0.824258	0.017185	2.178846
23	1	0	2.038788	-1.210496	1.797867
24	6	0	1.602612	1.909992	0.312441
25	1	0	1.645417	2.321475	-0.697675
26	1	0	0.615044	2.115229	0.725141
27	1	0	2.334193	2.449825	0.917789

13_(P_V)_method_B_toluene.log

Input orientation:

Center Number	Atomic Number	Atomic Type	Coordinates (Angstroms) X	Y	Z
1	15	0	0.799420	-0.543541	-0.768284
2	1	0	0.945772	0.131873	-2.004192
3	8	0	1.100268	-2.000866	-0.809480
4	6	0	-0.924988	-0.175253	-0.341860
5	6	0	-1.589045	0.934327	-0.868873
6	6	0	-1.609912	-1.040533	0.514224
7	6	0	-2.914018	1.184491	-0.532547
8	1	0	-1.078655	1.600141	-1.552818
9	6	0	-2.933900	-0.788082	0.851531
10	1	0	-1.107792	-1.917730	0.898553
11	6	0	-3.585487	0.325309	0.331043
12	1	0	-3.423249	2.042609	-0.949783
13	1	0	-3.458728	-1.463686	1.513242
14	1	0	-4.617978	0.517890	0.589828
15	6	0	1.934892	0.422848	0.328959
16	6	0	3.359831	0.184907	-0.204885
17	1	0	4.074763	0.720096	0.423176
18	1	0	3.618142	-0.872671	-0.193391
19	1	0	3.474883	0.553483	-1.225890
20	6	0	1.817170	-0.106090	1.766993
21	1	0	2.528839	0.420658	2.405896
22	1	0	0.819920	0.055784	2.177029
23	1	0	2.041950	-1.170845	1.818179
24	6	0	1.598223	1.919621	0.276451
25	1	0	1.636944	2.311308	-0.741475
26	1	0	0.612012	2.130950	0.688121
27	1	0	2.330666	2.473459	0.867116

13_(P_V)_method_B_toluene_smd.log

Input orientation:

Center Number	Atomic Number	Atomic Type	Coordinates (Angstroms) X	Y	Z
1	15	0	0.798149	-0.532211	-0.771203
2	1	0	0.939358	0.159651	-1.998263
3	8	0	1.100075	-1.988525	-0.833361
4	6	0	-0.928354	-0.171062	-0.340067
5	6	0	-1.599122	0.931876	-0.872468
6	6	0	-1.609253	-1.036414	0.519241
7	6	0	-2.925846	1.175696	-0.538247
8	1	0	-1.091597	1.597216	-1.559189
9	6	0	-2.934977	-0.790454	0.854176
10	1	0	-1.103425	-1.909137	0.909384
11	6	0	-3.592992	0.316488	0.328324
12	1	0	-3.439806	2.029127	-0.960012
13	1	0	-3.456244	-1.466687	1.518509
14	1	0	-4.627214	0.503844	0.585138
15	6	0	1.937748	0.424293	0.331725
16	6	0	3.359691	0.176503	-0.203279
17	1	0	4.080356	0.712576	0.418214
18	1	0	3.616018	-0.881853	-0.185776
19	1	0	3.475074	0.536485	-1.227838
20	6	0	1.816997	-0.103561	1.768694
21	1	0	2.538332	0.410899	2.407638
22	1	0	0.824013	0.072226	2.184005
23	1	0	2.026562	-1.171883	1.821366
24	6	0	1.612989	1.922944	0.278814
25	1	0	1.655530	2.315327	-0.739083
26	1	0	0.628750	2.144337	0.690852
27	1	0	2.349131	2.472758	0.869567

13_(P_V)_method_B_water.log

Input orientation:

Center Number	Atomic Number	Atomic Type	Coordinates (Angstroms) X	Y	Z
1	15	0	0.797971	-0.512962	-0.793093
2	1	0	0.939038	0.191951	-2.009069

3	8	0	1.099450	-1.974650	-0.891692
4	6	0	-0.924961	-0.165323	-0.352468
5	6	0	-1.610923	0.909044	-0.923583
6	6	0	-1.586834	-0.996699	0.555420
7	6	0	-2.935833	1.156085	-0.582742
8	1	0	-1.117646	1.550047	-1.642487
9	6	0	-2.910718	-0.747735	0.895668
10	1	0	-1.070101	-1.843131	0.986639
11	6	0	-3.585039	0.329629	0.328637
12	1	0	-3.461190	1.987134	-1.032977
13	1	0	-3.416560	-1.395649	1.598380
14	1	0	-4.616812	0.520231	0.591144
15	6	0	1.935794	0.412407	0.332177
16	6	0	3.363090	0.188695	-0.201894
17	1	0	4.071031	0.723424	0.433823
18	1	0	3.631909	-0.866600	-0.196563
19	1	0	3.478090	0.567734	-1.218712
20	6	0	1.813777	-0.145664	1.758919
21	1	0	2.526602	0.367234	2.407081
22	1	0	0.817229	0.011482	2.171653
23	1	0	2.037057	-1.211693	1.792593
24	6	0	1.597642	1.910641	0.309820
25	1	0	1.652550	2.326393	-0.697314
26	1	0	0.604791	2.110916	0.710790
27	1	0	2.320536	2.447516	0.926711

13_(P_V)_method_B_water_smd.log

Input orientation:

Center Number	Atomic Number	Atomic Type	Coordinates (Angstroms) X	Y	Z
1	15	0	0.795446	-0.491642	-0.795963
2	1	0	0.941184	0.209987	-2.007951
3	8	0	1.098327	-1.962552	-0.905732
4	6	0	-0.926768	-0.161701	-0.354609
5	6	0	-1.616822	0.900471	-0.942935
6	6	0	-1.580514	-0.981309	0.569551
7	6	0	-2.942240	1.146520	-0.603665
8	1	0	-1.122738	1.530976	-1.670562
9	6	0	-2.905574	-0.733288	0.905924
10	1	0	-1.058097	-1.814101	1.020375
11	6	0	-3.585979	0.331124	0.321608
12	1	0	-3.472122	1.968428	-1.065613
13	1	0	-3.407196	-1.371282	1.620843
14	1	0	-4.618484	0.520725	0.582675
15	6	0	1.936304	0.408963	0.337857
16	6	0	3.359644	0.191746	-0.205305
17	1	0	4.067123	0.730750	0.427508
18	1	0	3.637281	-0.861870	-0.200350
19	1	0	3.464215	0.571126	-1.223268
20	6	0	1.818161	-0.161900	1.758006
21	1	0	2.532423	0.348401	2.407025
22	1	0	0.821528	-0.010671	2.172944
23	1	0	2.045064	-1.227888	1.784177
24	6	0	1.596353	1.905729	0.326703
25	1	0	1.634555	2.322524	-0.681014
26	1	0	0.609147	2.102562	0.743683
27	1	0	2.329718	2.438632	0.934950

13_(P_V)_method_D_DCM.log

Input orientation:

Center Number	Atomic Number	Atomic Type	Coordinates (Angstroms) X	Y	Z
1	15	0	0.822063	-0.529287	-0.826489
2	1	0	0.955874	0.190700	-2.039863
3	8	0	1.156626	-2.004521	-0.926356
4	6	0	-0.903634	-0.199360	-0.363893
5	6	0	-1.598304	0.895074	-0.898588
6	6	0	-1.542317	-1.048207	0.552209
7	6	0	-2.915180	1.147346	-0.508449
8	1	0	-1.117568	1.548730	-1.620820
9	6	0	-2.858779	-0.793783	0.940530
10	1	0	-1.012680	-1.908125	0.949755
11	6	0	-3.544436	0.305042	0.413080
12	1	0	-3.449824	1.994207	-0.927043
13	1	0	-3.349702	-1.453150	1.649428
14	1	0	-4.569170	0.500112	0.713789
15	6	0	1.917706	0.419189	0.331534
16	6	0	3.363744	0.241490	-0.168750
17	1	0	4.044940	0.786048	0.494629
18	1	0	3.658342	-0.811354	-0.172927
19	1	0	3.492249	0.641039	-1.180793
20	6	0	1.765815	-0.163434	1.746940
21	1	0	2.450602	0.355751	2.426632
22	1	0	0.749100	-0.031579	2.129624
23	1	0	2.009609	-1.229587	1.768023
24	6	0	1.527779	1.906311	0.315194
25	1	0	1.585438	2.332614	-0.692198
26	1	0	0.517306	2.066355	0.701100
27	1	0	2.223525	2.465240	0.950818

14_(P_III)_method_A.log

Input orientation:

Center Number	Atomic Number	Atomic Type	Coordinates (Angstroms) X	Y	Z
1	15	0	0.057994	0.998379	-0.584209
2	6	0	-1.550878	0.088047	-0.659286
3	6	0	-2.107271	-0.191624	-1.918861
4	6	0	-2.269791	-0.274377	0.491153
5	6	0	-3.335284	-0.849353	-2.027875
6	1	0	-1.585669	0.116133	-2.822401
7	6	0	-3.503537	-0.921034	0.382401
8	1	0	-1.861295	-0.038700	1.468681
9	6	0	-4.036624	-1.216336	-0.875919
10	1	0	-3.749619	-1.061102	-3.009693
11	1	0	-4.049272	-1.194476	1.281504
12	1	0	-4.996539	-1.718193	-0.958354
13	8	0	0.080034	1.415016	1.059860
14	1	0	0.071634	2.375192	1.156728
15	6	0	1.342719	-0.405359	-0.523015
16	6	0	2.702976	0.273172	-0.259826
17	1	0	2.718276	0.770699	0.714925
18	1	0	3.500119	-0.481088	-0.264255
19	1	0	2.941522	1.015924	-1.029785
20	6	0	1.053481	-1.437583	0.578741
21	1	0	0.117967	-1.974643	0.395349
22	1	0	1.863432	-2.178385	0.612162
23	1	0	0.990989	-0.964052	1.563227
24	6	0	1.366146	-1.087565	-1.903764
25	1	0	0.424873	-1.602012	-2.121881

26	1	0	1.561679	-0.370267	-2.709856
27	1	0	2.166098	-1.838236	-1.928857

14_(P_III)_method_A_DCM.log

Input orientation:

Center Number	Atomic Number	Atomic Type	Coordinates (Angstroms) X	Y	Z
1	15	0	-0.868792	1.071694	-0.857981
2	8	0	-1.945939	-0.155434	-0.552242
3	6	0	-2.007393	-0.874424	0.707327
4	1	0	-2.112773	-0.151616	1.523014
5	1	0	-1.069541	-1.422258	0.848866
6	6	0	-3.192930	-1.821369	0.659575
7	1	0	-3.088929	-2.537364	-0.161793
8	1	0	-3.255237	-2.381864	1.598706
9	1	0	-4.128965	-1.270427	0.524827
10	6	0	0.732891	0.334179	-0.300670
11	6	0	1.211634	-0.787462	-1.000803
12	6	0	1.528865	0.894074	0.710202
13	6	0	2.447533	-1.353124	-0.680059
14	1	0	0.615274	-1.226830	-1.797321
15	6	0	2.769190	0.330344	1.028312
16	1	0	1.173643	1.764911	1.250768
17	6	0	3.229959	-0.793382	0.336260
18	1	0	2.801306	-2.224058	-1.224249
19	1	0	3.374516	0.770295	1.815943
20	1	0	4.194126	-1.228433	0.583116
21	8	0	-1.059934	2.111574	0.448243
22	1	0	-1.645464	2.845339	0.212205

14_(P_III)_method_A_DCM_smd.log

Input orientation:

Center Number	Atomic Number	Atomic Type	Coordinates (Angstroms) X	Y	Z
1	15	0	-0.870362	1.047125	-0.877838
2	8	0	-1.949575	-0.169473	-0.547972
3	6	0	-2.008501	-0.874804	0.720846
4	1	0	-2.098612	-0.143611	1.531246
5	1	0	-1.077141	-1.434526	0.859808
6	6	0	-3.205045	-1.806186	0.693738
7	1	0	-3.118605	-2.533399	-0.120770
8	1	0	-3.263192	-2.356034	1.640219
9	1	0	-4.137052	-1.245821	0.563912
10	6	0	0.736648	0.323826	-0.314791
11	6	0	1.243991	-0.768874	-1.040430
12	6	0	1.505074	0.862079	0.728946
13	6	0	2.480870	-1.328889	-0.712256
14	1	0	0.671183	-1.188996	-1.864557
15	6	0	2.745956	0.303619	1.054791
16	1	0	1.131155	1.713469	1.288232
17	6	0	3.235060	-0.792257	0.337488
18	1	0	2.857403	-2.177193	-1.277259
19	1	0	3.329932	0.726717	1.867841
20	1	0	4.200020	-1.223064	0.590345
21	8	0	-1.059361	2.115763	0.403453
22	1	0	-1.646804	2.844893	0.147253

14_(P_III)_method_B.log

Input orientation:

Center Number	Atomic Number	Atomic Type	Coordinates (Angstroms) X	Y	Z
1	15	0	-0.872825	1.050114	-0.855866
2	8	0	-1.924126	-0.156565	-0.535083
3	6	0	-1.996114	-0.864090	0.718991
4	1	0	-2.108910	-0.141022	1.528119
5	1	0	-1.061894	-1.406274	0.875066
6	6	0	-3.174980	-1.812551	0.667230
7	1	0	-3.061178	-2.528397	-0.146476
8	1	0	-3.248471	-2.365769	1.604712
9	1	0	-4.105760	-1.266611	0.515971
10	6	0	0.728040	0.329537	-0.300256
11	6	0	1.213413	-0.775319	-1.007364
12	6	0	1.512801	0.873984	0.716999
13	6	0	2.442077	-1.338619	-0.689814
14	1	0	0.623928	-1.200800	-1.810874
15	6	0	2.746351	0.313336	1.032817
16	1	0	1.153075	1.733084	1.264516
17	6	0	3.212170	-0.793222	0.332908
18	1	0	2.801409	-2.196974	-1.241712
19	1	0	3.343575	0.742502	1.826612
20	1	0	4.172427	-1.226679	0.578781
21	8	0	-1.066211	2.092747	0.424762
22	1	0	-1.625757	2.831954	0.172209

14_(P_III)_method_B_DCM_smd.log

Input orientation:

Center Number	Atomic Number	Atomic Type	Coordinates (Angstroms) X	Y	Z
1	15	0	-0.868814	1.003807	-0.884693
2	8	0	-1.920354	-0.197934	-0.544428
3	6	0	-1.992111	-0.890356	0.725006
4	1	0	-2.038744	-0.154943	1.528860
5	1	0	-1.086354	-1.484729	0.853323
6	6	0	-3.221822	-1.769229	0.725821
7	1	0	-3.177142	-2.507146	-0.076134
8	1	0	-3.289737	-2.301727	1.676174
9	1	0	-4.127906	-1.175111	0.601796
10	6	0	0.739933	0.311900	-0.318027
11	6	0	1.274243	-0.745181	-1.063079
12	6	0	1.481048	0.826450	0.747506
13	6	0	2.508732	-1.292370	-0.736077
14	1	0	0.722942	-1.145182	-1.906085
15	6	0	2.719807	0.281750	1.073459
16	1	0	1.089666	1.651930	1.324865
17	6	0	3.234746	-0.778249	0.334868
18	1	0	2.906229	-2.113110	-1.318583
19	1	0	3.282845	0.687563	1.903945
20	1	0	4.199132	-1.198760	0.588027
21	8	0	-1.075141	2.082341	0.352975
22	1	0	-1.658158	2.802653	0.082728

14_(P_III)_method_B_DKM.log

Input orientation:

Center Number	Atomic Number	Atomic Type	Coordinates (Angstroms) X	Y	Z
1	15	0	-0.867971	1.057953	-0.839469
2	8	0	-1.924223	-0.152007	-0.538440
3	6	0	-2.003921	-0.872933	0.712856
4	1	0	-2.116807	-0.155687	1.526220
5	1	0	-1.072353	-1.419534	0.864401
6	6	0	-3.185451	-1.816367	0.651851
7	1	0	-3.072920	-2.529115	-0.164970
8	1	0	-3.258834	-2.374625	1.585965
9	1	0	-4.115555	-1.267148	0.507234
10	6	0	0.731680	0.332944	-0.290179
11	6	0	1.205987	-0.783298	-0.988066
12	6	0	1.530170	0.888988	0.710779
13	6	0	2.437024	-1.346084	-0.676472
14	1	0	0.607599	-1.220087	-1.778628
15	6	0	2.766335	0.329183	1.020472
16	1	0	1.182058	1.756945	1.251756
17	6	0	3.220912	-0.788918	0.330237
18	1	0	2.786714	-2.213558	-1.220000
19	1	0	3.373543	0.767552	1.801409
20	1	0	4.182346	-1.222216	0.571264
21	8	0	-1.066539	2.092852	0.438164
22	1	0	-1.636755	2.829527	0.195864

14_(P_III)_method_B_DMSO.log

Input orientation:

Center Number	Atomic Number	Atomic Type	Coordinates (Angstroms) X	Y	Z
1	15	0	-0.866738	1.060377	-0.833812
2	8	0	-1.923736	-0.150644	-0.538600
3	6	0	-2.007188	-0.874633	0.711722
4	1	0	-2.122964	-0.158845	1.525793
5	1	0	-1.076003	-1.421280	0.864545
6	6	0	-3.188212	-1.818143	0.645557
7	1	0	-3.072870	-2.530365	-0.171398
8	1	0	-3.263925	-2.377340	1.578831
9	1	0	-4.118186	-1.269005	0.499456
10	6	0	0.732711	0.333934	-0.286637
11	6	0	1.202885	-0.786460	-0.980935
12	6	0	1.536287	0.894253	0.708050
13	6	0	2.434786	-1.348958	-0.671821
14	1	0	0.600798	-1.227279	-1.766388
15	6	0	2.773377	0.334758	1.015215
16	1	0	1.192038	1.765359	1.246454
17	6	0	3.223799	-0.787420	0.328630
18	1	0	2.780912	-2.219786	-1.212186
19	1	0	3.384350	0.776442	1.791287
20	1	0	4.185726	-1.220606	0.567716
21	8	0	-1.066062	2.091885	0.444841
22	1	0	-1.638747	2.828119	0.205924

14_(P_III)_method_B_MeOH.log

Input orientation:

Center Number	Atomic Number	Atomic Type	Coordinates (Angstroms) X	Y	Z
1	15	0	-0.866878	1.060078	-0.834482
2	8	0	-1.923792	-0.150825	-0.538599
3	6	0	-2.006793	-0.874466	0.711839
4	1	0	-2.122160	-0.158509	1.525837
5	1	0	-1.075582	-1.421154	0.864466
6	6	0	-3.187924	-1.817910	0.646318
7	1	0	-3.072985	-2.530183	-0.170642
8	1	0	-3.263338	-2.377012	1.579684
9	1	0	-4.117897	-1.268719	0.500453
10	6	0	0.732595	0.333811	-0.287054
11	6	0	1.203270	-0.786086	-0.981782
12	6	0	1.535561	0.893629	0.708383
13	6	0	2.435073	-1.348608	-0.672372
14	1	0	0.601629	-1.226432	-1.767848
15	6	0	2.772549	0.334111	1.015848
16	1	0	1.190849	1.764355	1.247101
17	6	0	3.223475	-0.787580	0.328825
18	1	0	2.781630	-2.219034	-1.213117
19	1	0	3.383072	0.775407	1.792500
20	1	0	4.185348	-1.220770	0.568141
21	8	0	-1.066136	2.091986	0.444036
22	1	0	-1.638526	2.828278	0.204712

14_(P_III)_method_B_MeOH_smd.log

Input orientation:

Center Number	Atomic Number	Atomic Type	Coordinates (Angstroms) X	Y	Z
1	15	0	-0.862597	1.067680	-0.821317
2	8	0	-1.923056	-0.145833	-0.534956
3	6	0	-2.013999	-0.880438	0.713872
4	1	0	-2.138623	-0.169794	1.531252
5	1	0	-1.083241	-1.426882	0.868640
6	6	0	-3.191366	-1.823560	0.629966
7	1	0	-3.068157	-2.532060	-0.190338
8	1	0	-3.272193	-2.388897	1.560052
9	1	0	-4.122887	-1.275852	0.481387
10	6	0	0.735444	0.338248	-0.277528
11	6	0	1.194051	-0.791972	-0.964066
12	6	0	1.551565	0.907207	0.702121
13	6	0	2.426684	-1.355712	-0.660396
14	1	0	0.582853	-1.239644	-1.738908
15	6	0	2.789349	0.345703	1.003369
16	1	0	1.222634	1.787790	1.235613
17	6	0	3.228111	-0.786383	0.325765
18	1	0	2.763632	-2.234557	-1.194645
19	1	0	3.409957	0.794612	1.768261
20	1	0	4.190905	-1.221321	0.560634
21	8	0	-1.070505	2.094252	0.456092
22	1	0	-1.645522	2.831779	0.217377

14_(P_III)_method_B_THF.log

Input orientation:

Center Atomic Atomic Coordinates (Angstroms)

Number Number Type X Y Z

1	15	0	-0.868237	1.057480	-0.840621
2	8	0	-1.924320	-0.152248	-0.538368
3	6	0	-2.003275	-0.872504	0.713128
4	1	0	-2.115726	-0.154946	1.526316
5	1	0	-1.071584	-1.418993	0.864524
6	6	0	-3.184803	-1.816052	0.653096
7	1	0	-3.072679	-2.528938	-0.163646
8	1	0	-3.257774	-2.374075	1.587403
9	1	0	-4.114967	-1.266914	0.508628
10	6	0	0.731456	0.332753	-0.290907
11	6	0	1.206582	-0.782654	-0.989512
12	6	0	1.528925	0.887915	0.711311
13	6	0	2.437432	-1.345519	-0.677419
14	1	0	0.608926	-1.218614	-1.781094
15	6	0	2.764888	0.328020	1.021517
16	1	0	1.180038	1.755245	1.252791
17	6	0	3.220287	-0.789258	0.330551
18	1	0	2.787830	-2.212320	-1.221581
19	1	0	3.371340	0.765704	1.803435
20	1	0	4.181611	-1.222599	0.571971
21	8	0	-1.066599	2.093062	0.436851
22	1	0	-1.636309	2.829820	0.193872

14_(P_III)_method_B_THF_smd.log

Input orientation:

Center Number	Atomic Number	Atomic Type	Coordinates (Angstroms) X	Y	Z
1	15	0	-0.872063	1.020927	-0.885172
2	8	0	-1.931321	-0.170034	-0.534796
3	6	0	-1.994016	-0.863428	0.733906
4	1	0	-2.083278	-0.129535	1.535767
5	1	0	-1.066604	-1.419462	0.878675
6	6	0	-3.185798	-1.793160	0.718074
7	1	0	-3.099385	-2.526812	-0.084243
8	1	0	-3.243914	-2.329491	1.666887
9	1	0	-4.114394	-1.237776	0.582036
10	6	0	0.732254	0.318487	-0.319434
11	6	0	1.256544	-0.744563	-1.062886
12	6	0	1.478143	0.827313	0.745314
13	6	0	2.484778	-1.304158	-0.733872
14	1	0	0.702585	-1.139849	-1.906408
15	6	0	2.710718	0.270285	1.073130
16	1	0	1.095203	1.658032	1.320676
17	6	0	3.214874	-0.796439	0.337122
18	1	0	2.874460	-2.129855	-1.314710
19	1	0	3.277215	0.671643	1.903492
20	1	0	4.174337	-1.227077	0.592166
21	8	0	-1.064237	2.109019	0.349369
22	1	0	-1.643062	2.830296	0.077151

14_(P_III)_method_B_toluene.log

Input orientation:

Center Number	Atomic Number	Atomic Type	Coordinates (Angstroms) X	Y	Z
1	15	0	-0.870624	1.053702	-0.849803
2	8	0	-1.924831	-0.154104	-0.537245
3	6	0	-1.998474	-0.868110	0.715844
4	1	0	-2.109397	-0.147746	1.527157
5	1	0	-1.065362	-1.412600	0.867839
6	6	0	-3.178895	-1.813929	0.662356
7	1	0	-3.067822	-2.528318	-0.153099
8	1	0	-3.249940	-2.369517	1.598445
9	1	0	-4.109728	-1.266412	0.516686
10	6	0	0.729464	0.331137	-0.296663
11	6	0	1.210854	-0.777794	-1.000698
12	6	0	1.519042	0.879444	0.715143
13	6	0	2.440218	-1.341271	-0.684877
14	1	0	0.618642	-1.207215	-1.799999
15	6	0	2.753374	0.318790	1.029206
16	1	0	1.163953	1.741890	1.260377
17	6	0	3.215085	-0.792059	0.332666
18	1	0	2.796063	-2.202862	-1.233885
19	1	0	3.354014	0.751090	1.818650
20	1	0	4.175558	-1.225764	0.577086
21	8	0	-1.066567	2.094166	0.427697
22	1	0	-1.631585	2.831847	0.179362

14_(P_III)_method_B_toluene_smd.log

Input orientation:

Center Number	Atomic Number	Atomic Type	Coordinates (Angstroms) X	Y	Z
1	15	0	-0.872068	1.027992	-0.876079
2	8	0	-1.928861	-0.167731	-0.533016
3	6	0	-1.997669	-0.862982	0.731450
4	1	0	-2.097031	-0.131444	1.534678
5	1	0	-1.068351	-1.414156	0.884442
6	6	0	-3.184733	-1.799175	0.702338
7	1	0	-3.087211	-2.528794	-0.102070
8	1	0	-3.251386	-2.339421	1.648384
9	1	0	-4.114066	-1.247865	0.556893
10	6	0	0.731829	0.320545	-0.313105
11	6	0	1.245132	-0.754289	-1.046680
12	6	0	1.489634	0.840711	0.737175
13	6	0	2.473970	-1.313894	-0.721986
14	1	0	0.680166	-1.158520	-1.878482
15	6	0	2.722935	0.283642	1.060476
16	1	0	1.112925	1.680014	1.303937
17	6	0	3.216006	-0.794357	0.334496
18	1	0	2.855175	-2.148806	-1.295065
19	1	0	3.298998	0.693990	1.879673
20	1	0	4.176341	-1.224896	0.585996
21	8	0	-1.065490	2.102224	0.373839
22	1	0	-1.633205	2.831574	0.104951

14_(P_III)_method_B_water.log

Input orientation:

Center Number	Atomic Number	Atomic Type	Coordinates (Angstroms) X	Y	Z
1	15	0	-0.866606	1.060666	-0.833170
2	8	0	-1.923684	-0.150464	-0.538596
3	6	0	-2.007568	-0.874783	0.711615
4	1	0	-2.123750	-0.159158	1.525753
5	1	0	-1.076401	-1.421382	0.864634

6	6	0	-3.188478	-1.818371	0.644825
7	1	0	-3.072732	-2.530547	-0.172121
8	1	0	-3.264481	-2.377656	1.578013
9	1	0	-4.118456	-1.269295	0.498484
10	6	0	0.732821	0.334053	-0.286239
11	6	0	1.202513	-0.786817	-0.980123
12	6	0	1.536980	0.894850	0.707728
13	6	0	2.434505	-1.349296	-0.671292
14	1	0	0.599996	-1.228088	-1.764988
15	6	0	2.774167	0.335375	1.014608
16	1	0	1.193178	1.766322	1.245829
17	6	0	3.224105	-0.787271	0.328443
18	1	0	2.780216	-2.220509	-1.211293
19	1	0	3.385573	0.777428	1.790124
20	1	0	4.186082	-1.220456	0.567310
21	8	0	-1.065986	2.091793	0.445616
22	1	0	-1.638954	2.827971	0.207087

14_(P_III)_method_B_water_smd.log

Input orientation:

Center Number	Atomic Number	Atomic Type	Coordinates (Angstroms) X	Y	Z
1	15	0	-0.862726	1.076151	-0.815283
2	8	0	-1.923265	-0.140902	-0.539534
3	6	0	-2.009083	-0.883990	0.705139
4	1	0	-2.129481	-0.178488	1.526978
5	1	0	-1.077891	-1.431236	0.851121
6	6	0	-3.187043	-1.825982	0.621212
7	1	0	-3.068124	-2.528997	-0.203795
8	1	0	-3.262606	-2.396696	1.547845
9	1	0	-4.118667	-1.277097	0.481389
10	6	0	0.733056	0.341532	-0.273297
11	6	0	1.183558	-0.797262	-0.950856
12	6	0	1.556537	0.916855	0.696361
13	6	0	2.415739	-1.362463	-0.648361
14	1	0	0.566342	-1.250495	-1.717020
15	6	0	2.793735	0.353664	0.996656
16	1	0	1.233662	1.803459	1.222844
17	6	0	3.224687	-0.786515	0.327739
18	1	0	2.746161	-2.247626	-1.175315
19	1	0	3.419694	0.807330	1.753705
20	1	0	4.186656	-1.222667	0.561676
21	8	0	-1.073044	2.092873	0.470329
22	1	0	-1.644857	2.832917	0.232714

14_(P_III)_method_C_DCM.log

Input orientation:

Center Number	Atomic Number	Atomic Type	Coordinates (Angstroms) X	Y	Z
1	15	0	-0.865261	1.065374	-0.846525
2	8	0	-1.932246	-0.151390	-0.548178
3	6	0	-2.006621	-0.874625	0.703546
4	1	0	-2.115924	-0.159316	1.519491
5	1	0	-1.075123	-1.423031	0.850841
6	6	0	-3.189320	-1.816677	0.644797
7	1	0	-3.080700	-2.527781	-0.174312
8	1	0	-3.260021	-2.377387	1.577969
9	1	0	-4.119637	-1.266104	0.504841
10	6	0	0.737106	0.334620	-0.292955
11	6	0	1.212088	-0.778310	-0.994881
12	6	0	1.531325	0.887981	0.712550
13	6	0	2.443149	-1.340907	-0.682532
14	1	0	0.616030	-1.211772	-1.788978
15	6	0	2.767293	0.327438	1.022051
16	1	0	1.181327	1.753623	1.255996
17	6	0	3.224319	-0.787434	0.328226
18	1	0	2.794993	-2.205821	-1.228722
19	1	0	3.372717	0.762891	1.805977
20	1	0	4.185655	-1.220730	0.569366
21	8	0	-1.068393	2.100363	0.443307
22	1	0	-1.645074	2.832227	0.199138

14_(P_III)_method_C_DCM_smd.log

Input orientation:

Center Number	Atomic Number	Atomic Type	Coordinates (Angstroms) X	Y	Z
1	15	0	-0.865884	1.037383	-0.873246
2	8	0	-1.938473	-0.163342	-0.542032
3	6	0	-2.011733	-0.865076	0.723564
4	1	0	-2.104051	-0.135931	1.529697
5	1	0	-1.087845	-1.426355	0.871388
6	6	0	-3.207696	-1.789057	0.691022
7	1	0	-3.117945	-2.516909	-0.116602
8	1	0	-3.276300	-2.332797	1.635196
9	1	0	-4.132560	-1.227720	0.551679
10	6	0	0.739375	0.318109	-0.311152
11	6	0	1.234532	-0.775630	-1.029305
12	6	0	1.512223	0.855952	0.719332
13	6	0	2.464567	-1.335995	-0.707887
14	1	0	0.656532	-1.194814	-1.844550
15	6	0	2.746839	0.297304	1.038112
16	1	0	1.149335	1.709150	1.274538
17	6	0	3.223875	-0.799277	0.328379
18	1	0	2.832732	-2.185955	-1.267373
19	1	0	3.335909	0.720675	1.841367
20	1	0	4.184570	-1.230952	0.576684
21	8	0	-1.061398	2.109756	0.385189
22	1	0	-1.637627	2.837361	0.117663

14_(P_III)_method_D_DCM.log

Input orientation:

Center Number	Atomic Number	Atomic Type	Coordinates (Angstroms) X	Y	Z
1	15	0	-0.875604	1.101707	-0.870181
2	8	0	-1.942537	-0.133047	-0.569351
3	6	0	-1.957222	-0.860648	0.685281
4	1	0	-2.046381	-0.143956	1.507882
5	1	0	-1.008422	-1.395820	0.794349
6	6	0	-3.130312	-1.821120	0.662592
7	1	0	-3.037997	-2.527252	-0.168397
8	1	0	-3.160204	-2.390410	1.597703
9	1	0	-4.075609	-1.280114	0.557026
10	6	0	0.713702	0.350626	-0.314854
11	6	0	1.162896	-0.796025	-0.991361

12	6	0	1.516826	0.909434	0.688679
13	6	0	2.381435	-1.386744	-0.654412
14	1	0	0.553451	-1.235649	-1.777084
15	6	0	2.739947	0.319976	1.023077
16	1	0	1.177962	1.796908	1.211444
17	6	0	3.173594	-0.827874	0.354500
18	1	0	2.713996	-2.277756	-1.178300
19	1	0	3.352783	0.757584	1.805624
20	1	0	4.124079	-1.283670	0.614809
21	8	0	-1.063153	2.124507	0.449144
22	1	0	-1.647740	2.861830	0.223342

14_(P_III)_method_E_DCM.log

Input orientation:

Center Number	Atomic Number	Atomic Type	Coordinates (Angstroms) X	Y	Z
1	15	0	-0.872815	1.172273	-0.722364
2	8	0	-1.908087	-0.057655	-0.505625
3	6	0	-1.969343	-0.841456	0.692153
4	1	0	-2.275803	-0.197429	1.517604
5	1	0	-0.976139	-1.235746	0.916732
6	6	0	-2.958085	-1.963305	0.482806
7	1	0	-2.638448	-2.613183	-0.331282
8	1	0	-3.033416	-2.560432	1.391724
9	1	0	-3.946334	-1.568821	0.248284
10	6	0	0.717624	0.401597	-0.245857
11	6	0	1.132305	-0.714893	-0.972119
12	6	0	1.556724	0.914193	0.738265
13	6	0	2.349705	-1.319535	-0.706957
14	1	0	0.492141	-1.122786	-1.745855
15	6	0	2.780367	0.312658	1.001451
16	1	0	1.249128	1.782610	1.304337
17	6	0	3.177718	-0.804094	0.281885
18	1	0	2.654952	-2.190188	-1.271787
19	1	0	3.423550	0.717716	1.771562
20	1	0	4.131325	-1.271717	0.487634
21	8	0	-1.056781	2.093680	0.623629
22	1	0	-1.611772	2.854422	0.445638

14_(P_III)_method_E_DCM_smd.log

Input orientation:

Center Number	Atomic Number	Atomic Type	Coordinates (Angstroms) X	Y	Z
1	15	0	-0.899752	1.175648	-0.766606
2	8	0	-1.980188	0.006274	-0.473440
3	6	0	-2.086751	-0.686197	0.777956
4	1	0	-2.676683	-0.070810	1.459089
5	1	0	-1.094320	-0.820346	1.213893
6	6	0	-2.749861	-2.020639	0.541990
7	1	0	-2.141307	-2.646350	-0.111782
8	1	0	-2.879049	-2.538739	1.493268
9	1	0	-3.731656	-1.889653	0.085870
10	6	0	0.667160	0.366397	-0.275115
11	6	0	1.007904	-0.822338	-0.921028
12	6	0	1.564195	0.920747	0.632943
13	6	0	2.210173	-1.455004	-0.650264
14	1	0	0.324317	-1.262802	-1.637766

15	6	0	2.771970	0.289898	0.901884
16	1	0	1.315897	1.845052	1.137008
17	6	0	3.096124	-0.897605	0.262993
18	1	0	2.458270	-2.380398	-1.153514
19	1	0	3.459958	0.728276	1.613308
20	1	0	4.037896	-1.388039	0.472269
21	8	0	-1.021838	2.178370	0.525337
22	1	0	-1.544589	2.955204	0.307933

14_(P_V)_method_A.log

Input orientation:

Center Number	Atomic Number	Atomic Type	Coordinates (Angstroms) X	Y	Z
1	15	0	0.023087	0.809326	-0.296254
2	1	0	0.018036	1.733003	-1.379653
3	8	0	0.043944	1.418470	1.081074
4	6	0	1.504052	-0.198028	-0.662961
5	6	0	1.987475	-0.369839	-1.968845
6	6	0	2.191559	-0.784459	0.410220
7	6	0	3.131442	-1.136786	-2.201253
8	1	0	1.482032	0.102179	-2.807513
9	6	0	3.334229	-1.552699	0.175649
10	1	0	1.838009	-0.615005	1.422928
11	6	0	3.802641	-1.732426	-1.129096
12	1	0	3.502634	-1.261548	-3.214386
13	1	0	3.863597	-2.001899	1.010931
14	1	0	4.694715	-2.325285	-1.309792
15	6	0	-1.515252	-0.192337	-0.646240
16	6	0	-2.707570	0.768678	-0.448603
17	1	0	-3.646450	0.223098	-0.602807
18	1	0	-2.715072	1.191845	0.559591
19	1	0	-2.684620	1.597024	-1.166744
20	6	0	-1.594261	-1.347124	0.371070
21	1	0	-2.527733	-1.903484	0.222611
22	1	0	-0.764039	-2.051275	0.250746
23	1	0	-1.582257	-0.971572	1.398659
24	6	0	-1.506350	-0.736919	-2.085568
25	1	0	-1.402184	0.063972	-2.827329
26	1	0	-0.702517	-1.461913	-2.245643
27	1	0	-2.456825	-1.245402	-2.287775

14_(P_V)_method_A_DCM.log

Input orientation:

Center Number	Atomic Number	Atomic Type	Coordinates (Angstroms) X	Y	Z
1	15	0	-0.806854	1.058827	-0.670554
2	1	0	-0.858155	1.169711	-2.071146
3	8	0	-1.043586	2.340409	0.077339
4	8	0	-1.940988	-0.095629	-0.525248
5	6	0	-2.321225	-0.608686	0.785633
6	1	0	-2.719334	0.218587	1.380656
7	1	0	-1.428423	-1.003757	1.282856
8	6	0	-3.360807	-1.693161	0.581777
9	1	0	-2.956394	-2.515891	-0.015481
10	1	0	-3.667269	-2.090672	1.554869
11	1	0	-4.246067	-1.294214	0.077666
12	6	0	0.781507	0.281281	-0.262728

13	6	0	1.154305	-0.936731	-0.855685
14	6	0	1.657020	0.920202	0.627497
15	6	0	2.390712	-1.510479	-0.554178
16	1	0	0.482893	-1.437640	-1.547823
17	6	0	2.892423	0.340674	0.929626
18	1	0	1.365776	1.863510	1.078416
19	6	0	3.259165	-0.872167	0.338858
20	1	0	2.676200	-2.451949	-1.013599
21	1	0	3.566927	0.836449	1.621167
22	1	0	4.220753	-1.320023	0.571742

14_(P_V)_method_A_DCM_smd.log

Input orientation:

Center Number	Atomic Number	Atomic Type	Coordinates (Angstroms) X	Y	Z
1	15	0	-0.807464	1.051778	-0.688439
2	1	0	-0.854631	1.143277	-2.090412
3	8	0	-1.041896	2.343425	0.043771
4	8	0	-1.946536	-0.096162	-0.526330
5	6	0	-2.334262	-0.583683	0.793557
6	1	0	-2.758592	0.250311	1.361075
7	1	0	-1.441502	-0.947588	1.314382
8	6	0	-3.348075	-1.692918	0.604866
9	1	0	-2.919813	-2.525153	0.036610
10	1	0	-3.659649	-2.069045	1.585669
11	1	0	-4.235776	-1.326667	0.078881
12	6	0	0.782659	0.276746	-0.273703
13	6	0	1.171435	-0.924441	-0.890566
14	6	0	1.641402	0.898460	0.644643
15	6	0	2.405785	-1.499983	-0.582732
16	1	0	0.514880	-1.410452	-1.607839
17	6	0	2.875025	0.317404	0.952459
18	1	0	1.341822	1.830752	1.113597
19	6	0	3.256890	-0.879897	0.339660
20	1	0	2.703437	-2.428745	-1.061014
21	1	0	3.536407	0.800540	1.665976
22	1	0	4.217028	-1.329307	0.577549

14_(P_V)_method_B.log

Input orientation:

Center Number	Atomic Number	Atomic Type	Coordinates (Angstroms) X	Y	Z
1	15	0	-0.805103	1.009881	-0.631920
2	1	0	-0.845655	1.133568	-2.030534
3	8	0	-1.055362	2.243714	0.132208
4	8	0	-1.891470	-0.160638	-0.509926
5	6	0	-2.337827	-0.621863	0.787415
6	1	0	-2.710454	0.233797	1.350155
7	1	0	-1.485653	-1.045112	1.323835
8	6	0	-3.418355	-1.658580	0.576555
9	1	0	-3.038525	-2.508328	0.010215
10	1	0	-3.774278	-2.019555	1.542171
11	1	0	-4.262271	-1.231315	0.036397
12	6	0	0.796600	0.260015	-0.258555
13	6	0	1.189764	-0.942080	-0.853065
14	6	0	1.663028	0.908717	0.622106
15	6	0	2.433630	-1.488759	-0.567662

16	1	0	0.522709	-1.452505	-1.536138
17	6	0	2.906455	0.357554	0.909448
18	1	0	1.350773	1.840267	1.073662
19	6	0	3.291641	-0.838479	0.314671
20	1	0	2.733878	-2.419230	-1.030247
21	1	0	3.574340	0.862643	1.594034
22	1	0	4.260712	-1.265061	0.536836

14_(P_V)_method_B_DCM_smd.log

Input orientation:

Center Number	Atomic Number	Atomic Type	Coordinates (Angstroms) X	Y	Z
1	15	0	-0.808471	1.053578	-0.691881
2	1	0	-0.844142	1.126731	-2.091113
3	8	0	-1.028965	2.339953	0.008361
4	8	0	-1.937855	-0.062071	-0.514674
5	6	0	-2.324367	-0.556189	0.797394
6	1	0	-2.781086	0.263234	1.351790
7	1	0	-1.429635	-0.882531	1.329773
8	6	0	-3.292791	-1.698992	0.609762
9	1	0	-2.831455	-2.518173	0.057693
10	1	0	-3.600491	-2.074619	1.586744
11	1	0	-4.183686	-1.371798	0.073215
12	6	0	0.769847	0.280586	-0.277936
13	6	0	1.156320	-0.911115	-0.899095
14	6	0	1.621390	0.882257	0.649965
15	6	0	2.377771	-1.494939	-0.589769
16	1	0	0.505421	-1.383372	-1.624155
17	6	0	2.842566	0.293825	0.960674
18	1	0	1.325355	1.808098	1.123576
19	6	0	3.220168	-0.893048	0.341588
20	1	0	2.672643	-2.416386	-1.073679
21	1	0	3.498577	0.762934	1.681693
22	1	0	4.171461	-1.349311	0.581736

14_(P_V)_method_B_DKM.log

Input orientation:

Center Number	Atomic Number	Atomic Type	Coordinates (Angstroms) X	Y	Z
1	15	0	-0.807830	1.042685	-0.682353
2	1	0	-0.848431	1.127842	-2.080868
3	8	0	-1.038516	2.321109	0.027959
4	8	0	-1.926680	-0.084764	-0.514645
5	6	0	-2.301871	-0.599026	0.791554
6	1	0	-2.694657	0.224764	1.386410
7	1	0	-1.410206	-0.991246	1.282689
8	6	0	-3.338809	-1.680705	0.596234
9	1	0	-2.939512	-2.500549	0.000133
10	1	0	-3.637832	-2.075466	1.567488
11	1	0	-4.223683	-1.284487	0.099458
12	6	0	0.774065	0.279464	-0.272813
13	6	0	1.159015	-0.922004	-0.875720
14	6	0	1.631688	0.900850	0.636195
15	6	0	2.385736	-1.495042	-0.568133
16	1	0	0.502435	-1.410695	-1.583998
17	6	0	2.857950	0.323148	0.945440
18	1	0	1.333175	1.832614	1.095614

19	6	0	3.234502	-0.872727	0.343637
20	1	0	2.679815	-2.423983	-1.037071
21	1	0	3.518418	0.807072	1.651819
22	1	0	4.189804	-1.320201	0.582632

14_(P_V)_method_B_DMSO.log

Input orientation:

Center Number	Atomic Number	Atomic Type	Coordinates (Angstroms) X	Y	Z
1	15	0	-0.808126	1.048470	-0.687205
2	1	0	-0.849300	1.131862	-2.085284
3	8	0	-1.035456	2.332159	0.017943
4	8	0	-1.932775	-0.071334	-0.514426
5	6	0	-2.295331	-0.599058	0.791181
6	1	0	-2.687711	0.217822	1.395723
7	1	0	-1.397833	-0.990699	1.271415
8	6	0	-3.328180	-1.684146	0.594873
9	1	0	-2.929074	-2.497049	-0.010739
10	1	0	-3.616851	-2.088072	1.565409
11	1	0	-4.219453	-1.288669	0.108950
12	6	0	0.770502	0.282790	-0.273204
13	6	0	1.151470	-0.921535	-0.873197
14	6	0	1.629685	0.903208	0.635174
15	6	0	2.375951	-1.498135	-0.563206
16	1	0	0.493940	-1.409704	-1.580777
17	6	0	2.853729	0.321971	0.946659
18	1	0	1.335154	1.836915	1.093144
19	6	0	3.226365	-0.876727	0.347759
20	1	0	2.666798	-2.429276	-1.029636
21	1	0	3.515301	0.805013	1.652553
22	1	0	4.179771	-1.327154	0.588553

14_(P_V)_method_B_DMSO_smd.log

Input orientation:

Center Number	Atomic Number	Atomic Type	Coordinates (Angstroms) X	Y	Z
1	15	0	-0.814191	1.019814	-0.785811
2	1	0	-0.847855	0.994220	-2.186407
3	8	0	-1.028514	2.351061	-0.172436
4	8	0	-1.952553	-0.072973	-0.529117
5	6	0	-2.298509	-0.504573	0.816587
6	1	0	-2.748974	0.336689	1.342846
7	1	0	-1.386191	-0.795464	1.339676
8	6	0	-3.259472	-1.663633	0.715960
9	1	0	-2.805277	-2.506136	0.193759
10	1	0	-3.534385	-1.990475	1.719962
11	1	0	-4.169395	-1.373027	0.190086
12	6	0	0.760661	0.264147	-0.330490
13	6	0	1.225228	-0.853767	-1.029392
14	6	0	1.524673	0.793054	0.710871
15	6	0	2.437469	-1.437858	-0.685241
16	1	0	0.645524	-1.266613	-1.845729
17	6	0	2.735045	0.203075	1.057495
18	1	0	1.171931	1.664648	1.244888
19	6	0	3.191154	-0.910835	0.360163
20	1	0	2.794553	-2.300775	-1.231339
21	1	0	3.322399	0.614851	1.867378
22	1	0	4.135256	-1.366778	0.627951

14_(P_V)_method_B_MeOH.log

Input orientation:

Center Number	Atomic Number	Atomic Type	Coordinates (Angstroms) X	Y	Z
1	15	0	-0.808096	1.047800	-0.686734
2	1	0	-0.849201	1.131324	-2.084865
3	8	0	-1.035825	2.330933	0.018931
4	8	0	-1.932081	-0.072871	-0.514480
5	6	0	-2.296044	-0.599023	0.791235
6	1	0	-2.688474	0.218664	1.394654
7	1	0	-1.399188	-0.990704	1.272704
8	6	0	-3.329351	-1.683744	0.595111
9	1	0	-2.930222	-2.497471	-0.009384
10	1	0	-3.619182	-2.086581	1.565763
11	1	0	-4.219912	-1.288209	0.107947
12	6	0	0.770901	0.282420	-0.273201
13	6	0	1.152370	-0.921531	-0.873597
14	6	0	1.629846	0.902888	0.635346
15	6	0	2.377088	-1.497746	-0.563841
16	1	0	0.495003	-1.409704	-1.581348
17	6	0	2.854126	0.322035	0.946612
18	1	0	1.334835	1.836341	1.093529
19	6	0	3.227251	-0.876299	0.347319
20	1	0	2.668342	-2.428598	-1.030612
21	1	0	3.515527	0.805132	1.652635
22	1	0	4.180860	-1.326403	0.587937

14_(P_V)_method_B_MeOH_smd.log

Input orientation:

Center Number	Atomic Number	Atomic Type	Coordinates (Angstroms) X	Y	Z
1	15	0	-0.805065	1.034540	-0.702035
2	1	0	-0.845771	1.119125	-2.097841
3	8	0	-1.041881	2.327272	-0.002686
4	8	0	-1.935378	-0.074093	-0.514851
5	6	0	-2.304456	-0.589971	0.800001
6	1	0	-2.707826	0.233817	1.387700
7	1	0	-1.406745	-0.968470	1.289493
8	6	0	-3.326289	-1.682353	0.606238
9	1	0	-2.916790	-2.501406	0.014222
10	1	0	-3.618852	-2.076094	1.580789
11	1	0	-4.218344	-1.299383	0.109678
12	6	0	0.774229	0.282130	-0.277393
13	6	0	1.161929	-0.913842	-0.890429
14	6	0	1.624310	0.894552	0.644776
15	6	0	2.385191	-1.491061	-0.577504
16	1	0	0.511150	-1.393653	-1.610828
17	6	0	2.847362	0.311860	0.957579
18	1	0	1.329981	1.822439	1.115649
19	6	0	3.227095	-0.878883	0.347585
20	1	0	2.681969	-2.416150	-1.053488
21	1	0	3.502659	0.788629	1.674335
22	1	0	4.180099	-1.330352	0.590671

14_(P_V)_method_B_THF.log

Input orientation:

Center Number	Atomic Number	Atomic Type	Coordinates (Angstroms) X	Y	Z
1	15	0	-0.807759	1.041473	-0.681057
2	1	0	-0.848247	1.127241	-2.079660
3	8	0	-1.039144	2.318613	0.030603
4	8	0	-1.925348	-0.087663	-0.514628
5	6	0	-2.303338	-0.599161	0.791577
6	1	0	-2.696133	0.226065	1.384451
7	1	0	-1.412998	-0.991649	1.285035
8	6	0	-3.341248	-1.679979	0.596270
9	1	0	-2.941979	-2.501237	0.002090
10	1	0	-3.642552	-2.072902	1.567583
11	1	0	-4.224686	-1.283460	0.097207
12	6	0	0.774854	0.278731	-0.272603
13	6	0	1.160519	-0.922275	-0.875914
14	6	0	1.632325	0.900519	0.636236
15	6	0	2.387773	-1.494488	-0.568948
16	1	0	0.503977	-1.411255	-1.584060
17	6	0	2.859113	0.323635	0.944897
18	1	0	1.333035	1.831967	1.095813
19	6	0	3.236376	-0.871773	0.342672
20	1	0	2.682428	-2.423079	-1.038250
21	1	0	3.519485	0.807897	1.651148
22	1	0	4.192122	-1.318571	0.581201

14_(P_V)_method_B_THF_smd.log

Input orientation:

Center Number	Atomic Number	Atomic Type	Coordinates (Angstroms) X	Y	Z
1	15	0	-0.810395	1.054547	-0.695983
2	1	0	-0.843893	1.122229	-2.096039
3	8	0	-1.029269	2.341898	0.000085
4	8	0	-1.939593	-0.062795	-0.518979
5	6	0	-2.310573	-0.566604	0.793181
6	1	0	-2.745780	0.251697	1.366487
7	1	0	-1.411354	-0.912337	1.305793
8	6	0	-3.298903	-1.692811	0.609976
9	1	0	-2.860039	-2.510927	0.038292
10	1	0	-3.593603	-2.076903	1.587732
11	1	0	-4.194737	-1.345874	0.094474
12	6	0	0.767139	0.279802	-0.279310
13	6	0	1.155920	-0.913480	-0.895707
14	6	0	1.617271	0.884981	0.647407
15	6	0	2.377336	-1.495267	-0.582584
16	1	0	0.507846	-1.389445	-1.620835
17	6	0	2.838283	0.298759	0.962092
18	1	0	1.319244	1.812367	1.116718
19	6	0	3.217850	-0.889827	0.347931
20	1	0	2.673865	-2.418233	-1.062748
21	1	0	3.492693	0.771164	1.682476
22	1	0	4.169271	-1.344291	0.591201

14_(P_V)_method_B_toluene.log

Input orientation:

Center Number	Atomic Number	Atomic Type	Coordinates (Angstroms) X	Y	Z
1	15	0	-0.807057	1.031332	-0.662703
2	1	0	-0.846451	1.129604	-2.061758
3	8	0	-1.045148	2.292365	0.067965
4	8	0	-1.912321	-0.114848	-0.513804
5	6	0	-2.317537	-0.604762	0.789836
6	1	0	-2.707549	0.232459	1.367794
7	1	0	-1.440970	-1.004324	1.303186
8	6	0	-3.367554	-1.673679	0.589953
9	1	0	-2.971137	-2.506428	0.009962
10	1	0	-3.691004	-2.052814	1.559807
11	1	0	-4.236332	-1.269926	0.071510
12	6	0	0.782610	0.271944	-0.267394
13	6	0	1.171076	-0.928905	-0.868525
14	6	0	1.643459	0.902461	0.631949
15	6	0	2.404346	-1.492347	-0.569789
16	1	0	0.510695	-1.424947	-1.568367
17	6	0	2.876217	0.334361	0.932659
18	1	0	1.339326	1.833726	1.089007
19	6	0	3.256385	-0.860672	0.332084
20	1	0	2.701115	-2.421107	-1.037734
21	1	0	3.539365	0.825560	1.631621
22	1	0	4.217043	-1.300404	0.564401

14_(P_V)_method_B_toluene_smd.log

Input orientation:

Center Number	Atomic Number	Atomic Type	Coordinates (Angstroms) X	Y	Z
1	15	0	-0.806723	1.025673	-0.669096
2	1	0	-0.843495	1.113949	-2.069081
3	8	0	-1.043581	2.291651	0.053202
4	8	0	-1.914751	-0.117563	-0.513616
5	6	0	-2.328021	-0.592945	0.793423
6	1	0	-2.731160	0.248579	1.356656
7	1	0	-1.453913	-0.978428	1.322069
8	6	0	-3.366681	-1.672060	0.600741
9	1	0	-2.960287	-2.511651	0.036580
10	1	0	-3.694417	-2.039430	1.574394
11	1	0	-4.236462	-1.284314	0.070518
12	6	0	0.784828	0.267779	-0.271465
13	6	0	1.182743	-0.923606	-0.884984
14	6	0	1.637572	0.890741	0.640623
15	6	0	2.416163	-1.485705	-0.584660
16	1	0	0.530444	-1.413504	-1.596927
17	6	0	2.870489	0.323942	0.942765
18	1	0	1.328714	1.816331	1.106426
19	6	0	3.259410	-0.862223	0.330792
20	1	0	2.720058	-2.407270	-1.062760
21	1	0	3.527217	0.809867	1.651808
22	1	0	4.220430	-1.301164	0.564254

14_(P_V)_method_B_water.log

Input orientation:

Center	Atomic	Atomic	Coordinates (Angstroms)

Number	Number	Type	X	Y	Z
1	15	0	-0.808154	1.049106	-0.687634
2	1	0	-0.849395	1.132386	-2.085663
3	8	0	-1.035101	2.333313	0.017039
4	8	0	-1.933433	-0.069875	-0.514366
5	6	0	-2.294666	-0.599095	0.791129
6	1	0	-2.687001	0.217018	1.396736
7	1	0	-1.396566	-0.990701	1.270197
8	6	0	-3.327079	-1.684529	0.594629
9	1	0	-2.927992	-2.496645	-0.012044
10	1	0	-3.614659	-2.089495	1.565048
11	1	0	-4.219022	-1.289105	0.109880
12	6	0	0.770126	0.283136	-0.273198
13	6	0	1.150611	-0.921550	-0.872795
14	6	0	1.629547	0.903521	0.635000
15	6	0	2.374871	-1.498512	-0.562589
16	1	0	0.492914	-1.409728	-1.580193
17	6	0	2.853371	0.321924	0.946685
18	1	0	1.335475	1.837473	1.092760
19	6	0	3.225535	-0.877127	0.348168
20	1	0	2.665325	-2.429933	-1.028688
21	1	0	3.515115	0.804924	1.652440
22	1	0	4.178752	-1.327856	0.589122

14_(P_V)_method_B_water_smd.log

Input orientation:

Center Number	Atomic Number	Atomic Type	Coordinates (Angstroms) X	Y	Z
1	15	0	-0.806345	1.040684	-0.707362
2	1	0	-0.844805	1.123649	-2.102321
3	8	0	-1.042035	2.335410	-0.009224
4	8	0	-1.939460	-0.065379	-0.519478
5	6	0	-2.292594	-0.592127	0.796032
6	1	0	-2.689245	0.226612	1.394437
7	1	0	-1.388517	-0.973171	1.270543
8	6	0	-3.315392	-1.683986	0.606549
9	1	0	-2.912070	-2.497462	0.003604
10	1	0	-3.594793	-2.085011	1.581425
11	1	0	-4.213607	-1.298345	0.124393
12	6	0	0.769461	0.285337	-0.277931
13	6	0	1.153438	-0.913357	-0.887691
14	6	0	1.620160	0.897076	0.643915
15	6	0	2.374198	-1.494026	-0.571762
16	1	0	0.501709	-1.391860	-1.607492
17	6	0	2.840731	0.310987	0.959676
18	1	0	1.328283	1.826676	1.112126
19	6	0	3.216990	-0.882498	0.352925
20	1	0	2.668259	-2.420950	-1.044839
21	1	0	3.496503	0.786908	1.675892
22	1	0	4.167707	-1.336514	0.598244

14_(P_V)_method_C_DCM.log

Input orientation:

Center Number	Atomic Number	Atomic Type	Coordinates (Angstroms) X	Y	Z
1	15	0	-0.808007	1.044742	-0.668436
2	1	0	-0.849476	1.150792	-2.069281
3	8	0	-1.054835	2.318019	0.069091
4	8	0	-1.930359	-0.100399	-0.519758
5	6	0	-2.305761	-0.618362	0.786382
6	1	0	-2.695589	0.203811	1.385948
7	1	0	-1.415239	-1.016104	1.275885
8	6	0	-3.346305	-1.695698	0.586658
9	1	0	-2.949903	-2.514330	-0.013487
10	1	0	-3.646817	-2.094294	1.556204
11	1	0	-4.230151	-1.294355	0.091593
12	6	0	0.784027	0.282916	-0.265424
13	6	0	1.179208	-0.903144	-0.890472
14	6	0	1.631295	0.896149	0.657926
15	6	0	2.410232	-1.470610	-0.589256
16	1	0	0.529053	-1.383680	-1.610075
17	6	0	2.861920	0.323344	0.959304
18	1	0	1.322611	1.816639	1.133134
19	6	0	3.250734	-0.857746	0.336422
20	1	0	2.714294	-2.387984	-1.074253
21	1	0	3.516384	0.799552	1.676413
22	1	0	4.209377	-1.300898	0.569732

14_(P_V)_method_C_DCM_smd.log

Input orientation:

Center Number	Atomic Number	Atomic Type	Coordinates (Angstroms) X	Y	Z
1	15	0	-0.819495	1.046369	-0.614419
2	1	0	-0.867704	1.200117	-2.010504
3	8	0	-1.076680	2.290341	0.169183
4	8	0	-1.929106	-0.115314	-0.497896
5	6	0	-2.295983	-0.677747	0.793708
6	1	0	-2.719018	0.116144	1.409316
7	1	0	-1.396766	-1.057401	1.282018
8	6	0	-3.297323	-1.783065	0.560741
9	1	0	-2.870221	-2.575812	-0.054436
10	1	0	-3.590410	-2.213168	1.519948
11	1	0	-4.192544	-1.401026	0.068961
12	6	0	0.786656	0.290105	-0.250108
13	6	0	1.200975	-0.851911	-0.941482
14	6	0	1.624084	0.862963	0.707530
15	6	0	2.440593	-1.416736	-0.670787
16	1	0	0.559563	-1.299838	-1.690067
17	6	0	2.863533	0.293022	0.977728
18	1	0	1.303792	1.751042	1.234735
19	6	0	3.271072	-0.845001	0.289795
20	1	0	2.759270	-2.300381	-1.207331
21	1	0	3.510314	0.738651	1.721646
22	1	0	4.236629	-1.286151	0.499218

14_(P_V)_method_D_DCM.log

Input orientation:

Center Number	Atomic Number	Atomic Type	Coordinates (Angstroms) X	Y	Z
1	15	0	-0.834403	1.190725	-0.564663
2	1	0	-0.892161	1.420838	-1.949677
3	8	0	-1.038727	2.412090	0.285522
4	8	0	-1.977186	0.040919	-0.502751

Center Number	Atomic Number	Atomic Type	X	Y	Z
5	6	0	-2.276480	-0.612518	0.764679
6	1	0	-2.858839	0.082168	1.376258
7	1	0	-1.337713	-0.835610	1.283246
8	6	0	-3.048912	-1.880107	0.465238
9	1	0	-2.449311	-2.565031	-0.141485
10	1	0	-3.307311	-2.381808	1.403208
11	1	0	-3.974540	-1.652608	-0.071543
12	6	0	0.724359	0.338940	-0.218757
13	6	0	0.989607	-0.917749	-0.785895
14	6	0	1.676859	0.949502	0.608338
15	6	0	2.201074	-1.557740	-0.523408
16	1	0	0.249762	-1.395566	-1.420997
17	6	0	2.887642	0.304208	0.870226
18	1	0	1.461000	1.918962	1.044883
19	6	0	3.149583	-0.946625	0.304220
20	1	0	2.404586	-2.530475	-0.959800
21	1	0	3.623658	0.776173	1.513341
22	1	0	4.091383	-1.446776	0.507846

14_(P_V)_method_E_DCM.log

Input orientation:

Center Number	Atomic Number	Atomic Type	Coordinates (Angstroms) X	Y	Z
1	15	0	-0.822688	1.104861	-0.660119
2	1	0	-0.857325	1.235967	-2.053275
3	8	0	-1.053997	2.354009	0.087102
4	8	0	-1.925690	-0.029089	-0.527030
5	6	0	-2.219234	-0.623337	0.751542
6	1	0	-2.609823	0.149738	1.413566
7	1	0	-1.294348	-1.014452	1.181240
8	6	0	-3.226555	-1.726123	0.543998
9	1	0	-2.826511	-2.497501	-0.113101
10	1	0	-3.467537	-2.180602	1.504536
11	1	0	-4.143445	-1.332867	0.107037
12	6	0	0.742463	0.320434	-0.259017
13	6	0	1.077214	-0.907188	-0.827971
14	6	0	1.623835	0.937593	0.622527
15	6	0	2.283636	-1.510651	-0.515948
16	1	0	0.392325	-1.394433	-1.510824
17	6	0	2.830942	0.330295	0.935751
18	1	0	1.357737	1.890079	1.060518
19	6	0	3.159546	-0.891170	0.366714
20	1	0	2.540847	-2.464055	-0.956582
21	1	0	3.513794	0.810688	1.623089
22	1	0	4.101507	-1.363836	0.610494

14_(P_V)_method_E_DCM_smd.log

Input orientation:

Center Number	Atomic Number	Atomic Type	Coordinates (Angstroms) X	Y	Z
1	15	0	-0.842838	1.083019	-0.589648
2	1	0	-0.910287	1.268335	-1.975373
3	8	0	-1.082907	2.295830	0.213334
4	8	0	-1.919256	-0.078569	-0.476330
5	6	0	-2.171372	-0.730459	0.784141
6	1	0	-2.574778	0.003730	1.482399
7	1	0	-1.228010	-1.109208	1.184030
8	6	0	-3.147202	-1.854426	0.551011
9	1	0	-2.736806	-2.588315	-0.143061
10	1	0	-3.354749	-2.353083	1.498288
11	1	0	-4.085813	-1.474563	0.147348
12	6	0	0.750603	0.318751	-0.259231
13	6	0	1.112597	-0.855429	-0.917649
14	6	0	1.625861	0.897454	0.654074
15	6	0	2.339041	-1.445185	-0.660940
16	1	0	0.434558	-1.311543	-1.628832
17	6	0	2.853198	0.303864	0.911527
18	1	0	1.341857	1.810304	1.160905
19	6	0	3.208416	-0.865240	0.254789
20	1	0	2.617569	-2.357510	-1.171322
21	1	0	3.531554	0.754654	1.623683
22	1	0	4.166157	-1.327531	0.455187

15_(P_III)_method_A.log

Input orientation:

Center Number	Atomic Number	Atomic Type	Coordinates (Angstroms) X	Y	Z
1	15	0	0.040310	0.871676	-1.298686
2	6	0	1.498717	0.186127	-0.384729
3	6	0	2.682640	-0.014095	-1.111795
4	6	0	1.491146	-0.100626	0.990467
5	6	0	3.838170	-0.485570	-0.481181
6	1	0	2.699349	0.198836	-2.178127
7	6	0	2.645144	-0.566117	1.622810
8	1	0	0.581461	0.040212	1.566584
9	6	0	3.819971	-0.760790	0.887926
10	1	0	4.746846	-0.637320	-1.056959
11	1	0	2.628316	-0.780819	2.687787
12	1	0	4.715640	-1.127306	1.381629
13	6	0	-1.361901	0.060261	-0.407099
14	6	0	-1.677809	-1.292883	-0.676869
15	6	0	-2.168245	0.798029	0.472963
16	6	0	-2.789117	-1.860902	-0.039975
17	6	0	-3.270349	0.211549	1.100208
18	1	0	-1.924850	1.838365	0.661119
19	6	0	-3.582293	-1.123627	0.842547
20	1	0	-3.037543	-2.899471	-0.245427
21	1	0	-3.880504	0.797588	1.781833
22	1	0	-4.439968	-1.590216	1.318943
23	6	0	-0.843772	-2.132619	-1.618604
24	1	0	-0.673865	-1.621155	-2.573807
25	1	0	0.142561	-2.354223	-1.193971
26	1	0	-1.336824	-3.085417	-1.830596
27	8	0	-0.055792	2.408195	-0.591409
28	1	0	0.000390	3.086185	-1.276460

15_(P_III)_method_A_DCM.log

Input orientation:

Center Number	Atomic Number	Atomic Type	Coordinates (Angstroms) X	Y	Z
1	15	0	0.121533	1.196827	-0.940967
2	6	0	1.514999	0.265524	-0.155137
3	6	0	2.727001	0.203828	-0.863033
4	6	0	1.436965	-0.341734	1.109692

5	6	0	3.841193	-0.441918	-0.317876
6	1	0	2.799451	0.661858	-1.846506
7	6	0	2.549420	-0.985985	1.656404
8	1	0	0.505977	-0.313453	1.668036
9	6	0	3.752916	-1.037203	0.943647
10	1	0	4.771516	-0.482261	-0.877064
11	1	0	2.477169	-1.450380	2.635866
12	1	0	4.615587	-1.541878	1.369120
13	6	0	-1.354482	0.351846	-0.211610
14	6	0	-1.768083	-0.903819	-0.719800
15	6	0	-2.124314	0.986780	0.775436
16	6	0	-2.936720	-1.482982	-0.205192
17	6	0	-3.285591	0.392053	1.277012
18	1	0	-1.806844	1.954541	1.149223
19	6	0	-3.693666	-0.848656	0.784059
20	1	0	-3.259715	-2.446150	-0.592896
21	1	0	-3.865442	0.897640	2.044076
22	1	0	-4.596922	-1.320212	1.160727
23	6	0	-0.978036	-1.632054	-1.784281
24	1	0	-0.746204	-0.979129	-2.634403
25	1	0	-0.021830	-2.000137	-1.393936
26	1	0	-1.536306	-2.492303	-2.162827
27	8	0	0.151322	2.597663	0.011299
28	1	0	0.137262	3.379621	-0.557903

15_(P_III)_method_A_DCM_smd.log

Input orientation:

Center Number	Atomic Number	Atomic Type	Coordinates (Angstroms) X	Y	Z
1	15	0	0.128424	1.183516	-0.933143
2	6	0	1.525522	0.261930	-0.142178
3	6	0	2.726409	0.170612	-0.865774
4	6	0	1.461786	-0.304782	1.142496
5	6	0	3.844329	-0.465921	-0.316489
6	1	0	2.788387	0.598743	-1.863560
7	6	0	2.578784	-0.937836	1.693353
8	1	0	0.540244	-0.252943	1.714971
9	6	0	3.771267	-1.020072	0.964706
10	1	0	4.766393	-0.529424	-0.887786
11	1	0	2.518206	-1.369404	2.688863
12	1	0	4.637603	-1.516055	1.393884
13	6	0	-1.353272	0.338475	-0.212568
14	6	0	-1.782741	-0.903503	-0.742155
15	6	0	-2.112432	0.963815	0.789204
16	6	0	-2.955547	-1.479372	-0.231534
17	6	0	-3.277545	0.372458	1.286283
18	1	0	-1.784905	1.922246	1.179163
19	6	0	-3.700751	-0.855269	0.773204
20	1	0	-3.290497	-2.432022	-0.635394
21	1	0	-3.848290	0.870655	2.065390
22	1	0	-4.607147	-1.324642	1.146105
23	6	0	-1.007985	-1.621485	-1.823787
24	1	0	-0.781094	-0.959778	-2.668919
25	1	0	-0.049970	-2.001803	-1.448819
26	1	0	-1.576205	-2.473772	-2.207173
27	8	0	0.155657	2.584864	0.016264
28	1	0	0.113526	3.368698	-0.553441

15_(P_III)_method_B.log

Input orientation:

Center Number	Atomic Number	Atomic Type	Coordinates (Angstroms) X	Y	Z
1	15	0	0.108817	1.234298	-0.899797
2	6	0	1.492542	0.286219	-0.136561
3	6	0	2.649516	0.106431	-0.897164
4	6	0	1.457712	-0.220712	1.165668
5	6	0	3.751085	-0.561598	-0.371955
6	1	0	2.686213	0.491181	-1.908852
7	6	0	2.557388	-0.883030	1.694266
8	1	0	0.566878	-0.098533	1.766794
9	6	0	3.705342	-1.055820	0.925451
10	1	0	4.639998	-0.695045	-0.973993
11	1	0	2.519594	-1.268496	2.704618
12	1	0	4.559442	-1.576623	1.337648
13	6	0	-1.353049	0.364681	-0.203336
14	6	0	-1.735695	-0.886343	-0.727425
15	6	0	-2.140361	0.966813	0.780171
16	6	0	-2.889231	-1.492374	-0.232991
17	6	0	-3.285085	0.343417	1.262694
18	1	0	-1.850177	1.932653	1.167300
19	6	0	-3.661293	-0.891785	0.754454
20	1	0	-3.188428	-2.452598	-0.634723
21	1	0	-3.878871	0.824683	2.028453
22	1	0	-4.553173	-1.385123	1.117220
23	6	0	-0.926000	-1.580414	-1.792215
24	1	0	-0.672419	-0.901694	-2.609327
25	1	0	0.015570	-1.961519	-1.391917
26	1	0	-1.475335	-2.422241	-2.210420
27	8	0	0.110993	2.591587	0.072510
28	1	0	0.216183	3.379910	-0.465406

15_(P_III)_method_B_DCM_smd.log

Input orientation:

Center Number	Atomic Number	Atomic Type	Coordinates (Angstroms) X	Y	Z
1	15	0	0.119137	1.208916	-0.896570
2	6	0	1.509605	0.273067	-0.134366
3	6	0	2.679181	0.132815	-0.886020
4	6	0	1.470992	-0.258321	1.158728
5	6	0	3.789762	-0.519434	-0.358725
6	1	0	2.721382	0.535496	-1.890593
7	6	0	2.580171	-0.905947	1.688645
8	1	0	0.572626	-0.168328	1.754701
9	6	0	3.740780	-1.038515	0.929997
10	1	0	4.688354	-0.621758	-0.952862
11	1	0	2.539198	-1.310459	2.691639
12	1	0	4.602110	-1.547222	1.342872
13	6	0	-1.351676	0.351306	-0.198168
14	6	0	-1.753886	-0.893006	-0.726496
15	6	0	-2.134051	0.965332	0.783006
16	6	0	-2.921202	-1.479957	-0.237045
17	6	0	-3.292212	0.361884	1.260725
18	1	0	-1.832064	1.926299	1.174501
19	6	0	-3.687383	-0.867127	0.748610
20	1	0	-3.235220	-2.434316	-0.641898
21	1	0	-3.880638	0.852647	2.025081
22	1	0	-4.589306	-1.345927	1.107084
23	6	0	-0.953495	-1.602755	-1.786998
24	1	0	-0.660757	-0.926410	-2.593323

25	1	0	-0.033752	-2.025532	-1.376644
26	1	0	-1.527192	-2.419208	-2.223217
27	8	0	0.151427	2.576878	0.054844
28	1	0	0.116266	3.367508	-0.496341

15_(P_III)_method_B_DKM.log

Input orientation:

Center Number	Atomic Number	Atomic Type	Coordinates (Angstroms) X	Y	Z
1	15	0	0.114676	1.223516	-0.903916
2	6	0	1.499869	0.278619	-0.142298
3	6	0	2.673802	0.141165	-0.887145
4	6	0	1.450769	-0.266657	1.144356
5	6	0	3.778018	-0.521640	-0.360082
6	1	0	2.723480	0.553936	-1.887005
7	6	0	2.553158	-0.924841	1.674812
8	1	0	0.547606	-0.179401	1.733060
9	6	0	3.718093	-1.054302	0.922474
10	1	0	4.679580	-0.622194	-0.949327
11	1	0	2.503896	-1.340344	2.672556
12	1	0	4.573977	-1.571605	1.335033
13	6	0	-1.351052	0.362887	-0.200924
14	6	0	-1.740549	-0.890034	-0.717467
15	6	0	-2.138335	0.976776	0.775812
16	6	0	-2.900190	-1.485409	-0.221939
17	6	0	-3.289537	0.364989	1.259700
18	1	0	-1.844355	1.943393	1.158316
19	6	0	-3.672143	-0.872270	0.759003
20	1	0	-3.204199	-2.446617	-0.617459
21	1	0	-3.882229	0.855653	2.020317
22	1	0	-4.568096	-1.357439	1.122706
23	6	0	-0.933265	-1.598031	-1.774992
24	1	0	-0.681382	-0.930638	-2.601866
25	1	0	0.009400	-1.973289	-1.371967
26	1	0	-1.483155	-2.445540	-2.179925
27	8	0	0.143189	2.588618	0.053976
28	1	0	0.147130	3.378626	-0.494643

15_(P_III)_method_B_DMSO.log

Input orientation:

Center Number	Atomic Number	Atomic Type	Coordinates (Angstroms) X	Y	Z
1	15	0	0.116888	1.207055	-0.915762
2	6	0	1.503993	0.269080	-0.150049
3	6	0	2.698395	0.186785	-0.871118
4	6	0	1.438681	-0.323888	1.114525
5	6	0	3.806819	-0.465865	-0.340713
6	1	0	2.760777	0.634873	-1.854916
7	6	0	2.544729	-0.974847	1.647410
8	1	0	0.520253	-0.280167	1.684048
9	6	0	3.730291	-1.046817	0.920191
10	1	0	4.724214	-0.522653	-0.910991
11	1	0	2.482138	-1.428790	2.627471
12	1	0	4.589063	-1.557751	1.334578
13	6	0	-1.350833	0.359269	-0.201283
14	6	0	-1.748202	-0.896260	-0.705737
15	6	0	-2.134702	0.987928	0.768902
16	6	0	-2.912139	-1.479232	-0.204963
17	6	0	-3.290629	0.388946	1.257727
18	1	0	-1.834697	1.956297	1.142289
19	6	0	-3.680971	-0.850915	0.769007
20	1	0	-3.222194	-2.442386	-0.590839
21	1	0	-3.880771	0.891219	2.012707
22	1	0	-4.580471	-1.326335	1.136737
23	6	0	-0.944494	-1.620000	-1.755278
24	1	0	-0.679774	-0.960272	-2.584270
25	1	0	-0.008701	-2.004865	-1.345185
26	1	0	-1.502567	-2.462833	-2.158626
27	8	0	0.161027	2.583081	0.025736
28	1	0	0.122036	3.367266	-0.530434

15_(P_III)_method_B_MeOH.log

Input orientation:

Center Number	Atomic Number	Atomic Type	Coordinates (Angstroms) X	Y	Z
1	15	0	0.116670	1.210787	-0.913224
2	6	0	1.503263	0.271222	-0.148357
3	6	0	2.693215	0.176050	-0.874956
4	6	0	1.441258	-0.310518	1.121619
5	6	0	3.800535	-0.479019	-0.345076
6	1	0	2.753012	0.615923	-1.862610
7	6	0	2.546336	-0.963160	1.654237
8	1	0	0.526110	-0.256544	1.695551
9	6	0	3.727403	-1.048661	0.921121
10	1	0	4.714489	-0.546128	-0.919753
11	1	0	2.486517	-1.408082	2.638601
12	1	0	4.585391	-1.561132	1.335246
13	6	0	-1.350660	0.360092	-0.201314
14	6	0	-1.746442	-0.894744	-0.708710
15	6	0	-2.135105	0.985251	0.770641
16	6	0	-2.909332	-1.480604	-0.208958
17	6	0	-3.289925	0.383338	1.258489
18	1	0	-1.836349	1.953135	1.146271
19	6	0	-3.678659	-0.855863	0.766905
20	1	0	-3.218118	-2.443256	-0.597113
21	1	0	-3.880458	0.882859	2.014992
22	1	0	-4.577280	-1.333549	1.133860
23	6	0	-0.942231	-1.614722	-1.760472
24	1	0	-0.681766	-0.953399	-2.589548
25	1	0	-0.004130	-1.996386	-1.352709
26	1	0	-1.498087	-2.459295	-2.163248
27	8	0	0.158105	2.584326	0.032005
28	1	0	0.124396	3.370004	-0.522324

15_(P_III)_method_B_THF.log

Input orientation:

Center Number	Atomic Number	Atomic Type	Coordinates (Angstroms) X	Y	Z
1	15	0	0.114204	1.225091	-0.902749
2	6	0	1.499269	0.279579	-0.141501
3	6	0	2.671146	0.137156	-0.888534
4	6	0	1.451924	-0.261447	1.146999
5	6	0	3.775057	-0.526551	-0.361967
6	1	0	2.719399	0.546753	-1.889777

7	6	0	2.554031	-0.920353	1.676995
8	1	0	0.550314	-0.170225	1.737487
9	6	0	3.716901	-1.054943	0.922374
10	1	0	4.675042	-0.630986	-0.952958
11	1	0	2.506178	-1.332467	2.676218
12	1	0	4.572580	-1.572838	1.334634
13	6	0	-1.351259	0.363262	-0.200853
14	6	0	-1.739814	-0.889518	-0.718361
15	6	0	-2.139007	0.975829	0.776319
16	6	0	-2.899039	-1.486071	-0.223392
17	6	0	-3.289727	0.362788	1.259684
18	1	0	-1.845705	1.942368	1.159519
19	6	0	-3.671438	-0.874304	0.758002
20	1	0	-3.202359	-2.447165	-0.619736
21	1	0	-3.882801	0.852421	2.020670
22	1	0	-4.567070	-1.360398	1.121271
23	6	0	-0.931958	-1.596125	-1.776369
24	1	0	-0.680508	-0.927909	-2.602711
25	1	0	0.010927	-1.971045	-1.373555
26	1	0	-1.481257	-2.443698	-2.182006
27	8	0	0.140393	2.589193	0.056679
28	1	0	0.152734	3.379526	-0.491218

15_(P_III)_method_B_toluene.log

Input orientation:

Center Number	Atomic Number	Atomic Type	Coordinates (Angstroms) X	Y	Z
1	15	0	0.110950	1.232759	-0.898197
2	6	0	1.495391	0.284586	-0.137763
3	6	0	2.656984	0.117970	-0.895010
4	6	0	1.456802	-0.235660	1.159333
5	6	0	3.759390	-0.549926	-0.370913
6	1	0	2.697578	0.512213	-1.902814
7	6	0	2.557412	-0.898125	1.686899
8	1	0	0.562840	-0.124663	1.758003
9	6	0	3.709964	-1.057551	0.921662
10	1	0	4.651632	-0.673154	-0.970097
11	1	0	2.516559	-1.293873	2.693087
12	1	0	4.564634	-1.578335	1.332556
13	6	0	-1.352660	0.365013	-0.201223
14	6	0	-1.736155	-0.887301	-0.722683
15	6	0	-2.142241	0.971241	0.778181
16	6	0	-2.892678	-1.489833	-0.229754
17	6	0	-3.289939	0.351681	1.259578
18	1	0	-1.852047	1.937620	1.163999
19	6	0	-3.666960	-0.884744	0.753781
20	1	0	-3.192421	-2.450666	-0.629563
21	1	0	-3.884851	0.836349	2.022325
22	1	0	-4.560729	-1.375500	1.115423
23	6	0	-0.925172	-1.586906	-1.782865
24	1	0	-0.672804	-0.913528	-2.604728
25	1	0	0.017127	-1.963226	-1.379951
26	1	0	-1.472774	-2.432698	-2.194871
27	8	0	0.122523	2.591830	0.069555
28	1	0	0.189802	3.382355	-0.472786

15_(P_III)_method_B_water.log

Input orientation:

Center Number	Atomic Number	Atomic Type	Coordinates (Angstroms) X	Y	Z
1	15	0	0.117027	1.204515	-0.917402
2	6	0	1.504509	0.267580	-0.151227
3	6	0	2.701859	0.194076	-0.868511
4	6	0	1.437096	-0.333072	1.109591
5	6	0	3.811084	-0.456868	-0.337778
6	1	0	2.765932	0.647725	-1.849653
7	6	0	2.543871	-0.982805	1.642632
8	1	0	0.516514	-0.296462	1.676116
9	6	0	3.732415	-1.045486	0.919476
10	1	0	4.730744	-0.506585	-0.905053
11	1	0	2.479495	-1.442874	2.619714
12	1	0	4.591771	-1.555294	1.334024
13	6	0	-1.350965	0.358692	-0.201207
14	6	0	-1.749405	-0.897317	-0.703640
15	6	0	-2.134525	0.989811	0.767655
16	6	0	-2.914121	-1.478264	-0.202250
17	6	0	-3.291266	0.392882	1.257070
18	1	0	-1.833723	1.958547	1.139458
19	6	0	-3.682683	-0.847448	0.770332
20	1	0	-3.225038	-2.441760	-0.586562
21	1	0	-3.881211	0.897095	2.010907
22	1	0	-4.582832	-1.321280	1.138518
23	6	0	-0.945997	-1.623703	-1.751571
24	1	0	-0.677789	-0.965002	-2.580251
25	1	0	-0.012110	-2.011261	-1.339646
26	1	0	-1.505797	-2.465012	-2.155709
27	8	0	0.163145	2.582120	0.021692
28	1	0	0.120161	3.365373	-0.535560

15_(P_III)_method_D_DCM.log

Input orientation:

Center Number	Atomic Number	Atomic Type	Coordinates (Angstroms) X	Y	Z
1	15	0	0.098132	1.344174	-0.850418
2	6	0	1.464266	0.317656	-0.154827
3	6	0	2.652816	0.218273	-0.892882
4	6	0	1.368700	-0.346718	1.078412
5	6	0	3.731060	-0.528111	-0.408347
6	1	0	2.732446	0.721869	-1.852749
7	6	0	2.445731	-1.088040	1.565974
8	1	0	0.449018	-0.289317	1.652276
9	6	0	3.627880	-1.181347	0.822330
10	1	0	4.644967	-0.601158	-0.990069
11	1	0	2.363006	-1.597580	2.521429
12	1	0	4.462584	-1.763896	1.200419
13	6	0	-1.358208	0.427099	-0.190695
14	6	0	-1.691745	-0.833159	-0.738186
15	6	0	-2.170755	0.992535	0.801628
16	6	0	-2.832866	-1.489709	-0.260712
17	6	0	-3.303471	0.320541	1.268109
18	1	0	-1.905166	1.961553	1.209648
19	6	0	-3.636019	-0.925415	0.734053
20	1	0	-3.095072	-2.458213	-0.678501
21	1	0	-3.920197	0.769821	2.040852
22	1	0	-4.516303	-1.455894	1.084671
23	6	0	-0.834182	-1.484425	-1.796476
24	1	0	-0.564586	-0.777640	-2.590157
25	1	0	0.104371	-1.857301	-1.370835

26	1	0	-1.354796	-2.327839	-2.256923
27	8	0	0.123573	2.639354	0.238858
28	1	0	0.173043	3.473615	-0.247275

15_(P_V)_method_A.log

Input orientation:

Center	Atomic	Atomic	Coordinates (Angstroms)		
Number	Number	Type	X	Y	Z
1	15	0	0.048395	0.851080	-1.154495
2	1	0	-0.099602	2.155857	-0.614254
3	8	0	0.156031	0.797167	-2.654454
4	6	0	1.545589	0.214899	-0.314514
5	6	0	2.680416	-0.035067	-1.098571
6	6	0	1.599421	0.009913	1.072603
7	6	0	3.858848	-0.488421	-0.499082
8	1	0	2.625737	0.122971	-2.171657
9	6	0	2.778987	-0.440672	1.668459
10	1	0	0.721998	0.190786	1.688125
11	6	0	3.909202	-0.689928	0.882688
12	1	0	4.735035	-0.683550	-1.110643
13	1	0	2.815391	-0.600929	2.742153
14	1	0	4.825446	-1.042250	1.347890
15	6	0	-1.393117	0.026544	-0.396187
16	6	0	-1.724380	-1.317052	-0.693596
17	6	0	-2.195460	0.773124	0.479440
18	6	0	-2.856921	-1.864287	-0.076887
19	6	0	-3.317564	0.205895	1.087113
20	1	0	-1.947156	1.811534	0.685725
21	6	0	-3.645754	-1.119838	0.804695
22	1	0	-3.126884	-2.894038	-0.296243
23	1	0	-3.929205	0.795783	1.763024
24	1	0	-4.518494	-1.576219	1.263121
25	6	0	-0.901873	-2.153118	-1.646967
26	1	0	-0.822937	-1.673267	-2.626988
27	1	0	0.121198	-2.289448	-1.276987
28	1	0	-1.346275	-3.143862	-1.773166

15_(P_V)_method_A_DCM.log

Input orientation:

Center	Atomic	Atomic	Coordinates (Angstroms)		
Number	Number	Type	X	Y	Z
1	15	0	-0.100874	1.012688	-1.009801
2	1	0	-0.051806	0.657979	-2.378850
3	8	0	-0.206645	2.504105	-0.767386
4	6	0	-1.542156	0.087600	-0.372921
5	6	0	-2.636798	0.805368	0.128670
6	6	0	-1.589289	-1.315079	-0.415227
7	6	0	-3.767919	0.125683	0.590736
8	1	0	-2.595749	1.889688	0.158760
9	6	0	-2.721558	-1.990667	0.044756
10	1	0	-0.746393	-1.883030	-0.800203
11	6	0	-3.811013	-1.270796	0.548569
12	1	0	-4.611917	0.686231	0.981557
13	1	0	-2.753010	-3.075620	0.012149
14	1	0	-4.689860	-1.798470	0.907340
15	6	0	1.410072	0.195938	-0.399276
16	6	0	1.800842	0.246317	0.961134
17	6	0	2.209196	-0.474113	-1.339485
18	6	0	2.989476	-0.399919	1.325714
19	6	0	3.389126	-1.113604	-0.952359
20	1	0	1.911083	-0.497883	-2.384452
21	6	0	3.776959	-1.074162	0.387233
22	1	0	3.304614	-0.370873	2.365318
23	1	0	3.996275	-1.629347	-1.689794
24	1	0	4.693066	-1.563144	0.705598
25	6	0	0.984093	0.966869	2.009304
26	1	0	0.847689	2.021755	1.752444
27	1	0	-0.015537	0.528895	2.109392
28	1	0	1.472837	0.909627	2.984871

15_(P_V)_method_A_DCM_smd.log

Input orientation:

Center	Atomic	Atomic	Coordinates (Angstroms)		
Number	Number	Type	X	Y	Z
1	15	0	-0.107977	1.008097	-0.993521
2	1	0	-0.051465	0.660212	-2.363247
3	8	0	-0.218718	2.497588	-0.739393
4	6	0	-1.554875	0.073921	-0.375254
5	6	0	-2.641460	0.782299	0.156646
6	6	0	-1.612895	-1.326272	-0.463244
7	6	0	-3.774396	0.095397	0.604650
8	1	0	-2.596061	1.865387	0.220341
9	6	0	-2.746575	-2.009100	-0.016860
10	1	0	-0.776839	-1.886023	-0.874438
11	6	0	-3.827473	-1.298867	0.518586
12	1	0	-4.612198	0.649127	1.018991
13	1	0	-2.785723	-3.092625	-0.084866
14	1	0	-4.707692	-1.832220	0.866738
15	6	0	1.407212	0.195224	-0.381397
16	6	0	1.820836	0.274955	0.971054
17	6	0	2.186128	-0.503047	-1.318441
18	6	0	3.010002	-0.374137	1.331821
19	6	0	3.366773	-1.143881	-0.935256
20	1	0	1.869966	-0.546723	-2.357607
21	6	0	3.776563	-1.077248	0.397093
22	1	0	3.340909	-0.323634	2.365980
23	1	0	3.957847	-1.681832	-1.670615
24	1	0	4.693523	-1.567522	0.712510
25	6	0	1.036510	1.035817	2.014018
26	1	0	1.001584	2.105469	1.782003
27	1	0	-0.000498	0.687465	2.076936
28	1	0	1.491798	0.914208	3.000562

15_(P_V)_method_B.log

Input orientation:

Center	Atomic	Atomic	Coordinates (Angstroms)		
Number	Number	Type	X	Y	Z
1	15	0	-0.104512	1.046598	-0.952860
2	1	0	-0.048822	0.730369	-2.330439
3	8	0	-0.217727	2.493946	-0.651645
4	6	0	-1.533723	0.086434	-0.368748
5	6	0	-2.637334	0.784069	0.122471
6	6	0	-1.568161	-1.308590	-0.423472
7	6	0	-3.763671	0.092576	0.554641

8	1	0	-2.599203	1.864080	0.163909
9	6	0	-2.695091	-1.996611	0.006443
10	1	0	-0.713751	-1.860999	-0.792963
11	6	0	-3.793424	-1.295800	0.496155
12	1	0	-4.616180	0.637942	0.936518
13	1	0	-2.715856	-3.077269	-0.036025
14	1	0	-4.669698	-1.833260	0.833274
15	6	0	1.394446	0.203688	-0.375331
16	6	0	1.814748	0.270344	0.967996
17	6	0	2.152035	-0.502859	-1.310825
18	6	0	2.989614	-0.392333	1.317515
19	6	0	3.317664	-1.161248	-0.939203
20	1	0	1.832478	-0.533132	-2.344918
21	6	0	3.734445	-1.103247	0.382599
22	1	0	3.328387	-0.347679	2.344688
23	1	0	3.894520	-1.703475	-1.675691
24	1	0	4.642811	-1.604517	0.689242
25	6	0	1.036363	1.028207	2.011172
26	1	0	0.861405	2.057584	1.700777
27	1	0	0.056282	0.575906	2.177013
28	1	0	1.566758	1.031310	2.961499

15_(P_V)_method_B_DCM_smd.log

Input orientation:

Center Number	Atomic Number	Atomic Type	Coordinates (Angstroms) X	Y	Z
1	15	0	-0.103208	0.980021	-1.016731
2	1	0	-0.043473	0.590229	-2.369805
3	8	0	-0.207727	2.453492	-0.809030
4	6	0	-1.543512	0.067060	-0.390511
5	6	0	-2.578172	0.766886	0.230074
6	6	0	-1.644829	-1.317474	-0.550187
7	6	0	-3.701193	0.088312	0.692824
8	1	0	-2.500517	1.838972	0.348277
9	6	0	-2.767945	-1.992464	-0.089659
10	1	0	-0.849341	-1.870648	-1.033143
11	6	0	-3.796179	-1.289826	0.533994
12	1	0	-4.499879	0.636003	1.175183
13	1	0	-2.841513	-3.064472	-0.215855
14	1	0	-4.669737	-1.817375	0.893777
15	6	0	1.401628	0.189566	-0.384495
16	6	0	1.803151	0.287582	0.963709
17	6	0	2.186284	-0.516949	-1.299689
18	6	0	2.987134	-0.345515	1.339969
19	6	0	3.361468	-1.142657	-0.901158
20	1	0	1.877844	-0.577023	-2.335702
21	6	0	3.759700	-1.054972	0.425623
22	1	0	3.309105	-0.278658	2.371438
23	1	0	3.958149	-1.686142	-1.621219
24	1	0	4.673501	-1.533487	0.752829
25	6	0	1.002237	1.045456	1.988613
26	1	0	0.869999	2.087442	1.696781
27	1	0	0.004783	0.618558	2.110060
28	1	0	1.497047	1.020123	2.957822

15_(P_V)_method_B_DKM.log

Input orientation:

Center Number	Atomic Number	Atomic Type	Coordinates (Angstroms) X	Y	Z
1	15	0	-0.095430	0.996549	-1.016397
2	1	0	-0.046003	0.621149	-2.374953
3	8	0	-0.192104	2.468911	-0.798543
4	6	0	-1.531874	0.088984	-0.380215
5	6	0	-2.605579	0.806688	0.146735
6	6	0	-1.595002	-1.305717	-0.437619
7	6	0	-3.730580	0.136595	0.615666
8	1	0	-2.552735	1.885562	0.189114
9	6	0	-2.720541	-1.972379	0.028618
10	1	0	-0.767591	-1.874589	-0.841777
11	6	0	-3.788504	-1.251354	0.556646
12	1	0	-4.559100	0.697933	1.025664
13	1	0	-2.764327	-3.051963	-0.017285
14	1	0	-4.663339	-1.772363	0.921574
15	6	0	1.404605	0.190272	-0.399912
16	6	0	1.786249	0.240757	0.956208
17	6	0	2.208077	-0.473970	-1.330006
18	6	0	2.972726	-0.391227	1.324238
19	6	0	3.385078	-1.100846	-0.939497
20	1	0	1.914543	-0.499769	-2.371489
21	6	0	3.765198	-1.057088	0.394493
22	1	0	3.282472	-0.358819	2.360813
23	1	0	3.996924	-1.610579	-1.670685
24	1	0	4.680473	-1.536041	0.715455
25	6	0	0.955446	0.942248	1.998762
26	1	0	0.717165	1.961767	1.697902
27	1	0	0.007540	0.424971	2.159590
28	1	0	1.481019	0.976353	2.950690

15_(P_V)_method_B_DMSO.log

Input orientation:

Center Number	Atomic Number	Atomic Type	Coordinates (Angstroms) X	Y	Z
1	15	0	-0.094851	0.989464	-1.024073
2	1	0	-0.046918	0.607813	-2.380155
3	8	0	-0.188661	2.465501	-0.817530
4	6	0	-1.532151	0.090217	-0.379947
5	6	0	-2.605381	0.810907	0.144050
6	6	0	-1.595624	-1.304869	-0.430331
7	6	0	-3.730418	0.143554	0.616976
8	1	0	-2.553621	1.889976	0.181762
9	6	0	-2.721226	-1.968765	0.039794
10	1	0	-0.769043	-1.875941	-0.832845
11	6	0	-3.788802	-1.244750	0.564771
12	1	0	-4.558479	0.707159	1.024696
13	1	0	-2.765357	-3.048488	-0.000979
14	1	0	-4.663646	-1.763629	0.932580
15	6	0	1.406143	0.189252	-0.403480
16	6	0	1.783137	0.235275	0.954335
17	6	0	2.215683	-0.467430	-1.334132
18	6	0	2.971549	-0.392510	1.323464
19	6	0	3.394618	-1.089938	-0.942365
20	1	0	1.924714	-0.492340	-2.376241
21	6	0	3.770386	-1.050150	0.393105
22	1	0	3.277340	-0.363945	2.361258
23	1	0	4.010637	-1.594421	-1.673633
24	1	0	4.686653	-1.526543	0.714930
25	6	0	0.945829	0.926771	1.998489
26	1	0	0.712006	1.950450	1.708095
27	1	0	-0.004230	0.409920	2.146762

28	1	0	1.464517	0.949495	2.954434

15_(P_V)_method_B_MeOH.log

Input orientation:

Center	Atomic	Atomic	Coordinates (Angstroms)		
Number	Number	Type	X	Y	Z
1	15	0	-0.094849	0.990505	-1.023086
2	1	0	-0.046893	0.609647	-2.379479
3	8	0	-0.188870	2.466150	-0.815246
4	6	0	-1.532040	0.090380	-0.379751
5	6	0	-2.605740	0.810777	0.143664
6	6	0	-1.595078	-1.304705	-0.430080
7	6	0	-3.730813	0.143106	0.616022
8	1	0	-2.554140	1.889857	0.181306
9	6	0	-2.720721	-1.968929	0.039467
10	1	0	-0.768013	-1.875519	-0.831978
11	6	0	-3.788777	-1.245204	0.563831
12	1	0	-4.559255	0.706485	1.023292
13	1	0	-2.764503	-3.048674	-0.001240
14	1	0	-4.663672	-1.764322	0.931193
15	6	0	1.406024	0.189417	-0.403137
16	6	0	1.783440	0.235499	0.954525
17	6	0	2.214938	-0.467821	-1.333888
18	6	0	2.971630	-0.392834	1.323418
19	6	0	3.393634	-1.090921	-0.942379
20	1	0	1.923748	-0.492491	-2.375955
21	6	0	3.769809	-1.051098	0.392967
22	1	0	3.277827	-0.364110	2.361097
23	1	0	4.009251	-1.595744	-1.673756
24	1	0	4.685974	-1.527835	0.714586
25	6	0	0.946681	0.927652	1.998667
26	1	0	0.710259	1.950158	1.706330
27	1	0	-0.002057	0.409146	2.149748
28	1	0	1.467011	0.953465	2.953652

15_(P_V)_method_B_THF.log

Input orientation:

Center	Atomic	Atomic	Coordinates (Angstroms)		
Number	Number	Type	X	Y	Z
1	15	0	-0.095539	0.997605	-1.015386
2	1	0	-0.045876	0.622629	-2.374223
3	8	0	-0.192755	2.469312	-0.796127
4	6	0	-1.531813	0.088725	-0.380324
5	6	0	-2.605540	0.806130	0.146933
6	6	0	-1.594953	-1.305914	-0.438522
7	6	0	-3.730554	0.135769	0.615414
8	1	0	-2.552378	1.884981	0.189745
9	6	0	-2.720495	-1.972848	0.027286
10	1	0	-0.767431	-1.874566	-0.842813
11	6	0	-3.788469	-1.252123	0.555652
12	1	0	-4.559123	0.696885	1.025635
13	1	0	-2.764285	-3.052421	-0.019150
14	1	0	-4.663317	-1.773344	0.920275
15	6	0	1.404348	0.190374	-0.399436
16	6	0	1.786592	0.241362	0.956468
17	6	0	2.207137	-0.474677	-1.329454
18	6	0	2.972809	-0.391185	1.324323
19	6	0	3.383888	-1.102132	-0.939150
20	1	0	1.913373	-0.500360	-2.370900
21	6	0	3.764508	-1.058004	0.394652
22	1	0	3.283048	-0.358315	2.360747
23	1	0	3.995282	-1.612382	-1.670368
24	1	0	4.679672	-1.537271	0.715487
25	6	0	0.956667	0.944156	1.998830
26	1	0	0.717664	1.963088	1.696601
27	1	0	0.009119	0.426749	2.161418
28	1	0	1.483223	0.979815	2.950179

15_(P_V)_method_B_toluene.log

Input orientation:

Center	Atomic	Atomic	Coordinates (Angstroms)		
Number	Number	Type	X	Y	Z
1	15	0	-0.098993	1.018479	-0.992075
2	1	0	-0.047151	0.664834	-2.358438
3	8	0	-0.203479	2.480603	-0.741109
4	6	0	-1.531750	0.087839	-0.376113
5	6	0	-2.617252	0.797202	0.137737
6	6	0	-1.583031	-1.307045	-0.432370
7	6	0	-3.742319	0.118237	0.593108
8	1	0	-2.569914	1.876531	0.179412
9	6	0	-2.708575	-1.982608	0.020567
10	1	0	-0.744624	-1.869371	-0.823102
11	6	0	-3.788608	-1.269922	0.534380
12	1	0	-4.580377	0.673097	0.992618
13	1	0	-2.742789	-3.062730	-0.023707
14	1	0	-4.663638	-1.797772	0.889161
15	6	0	1.400086	0.195878	-0.390984
16	6	0	1.795868	0.252050	0.960575
17	6	0	2.186153	-0.484873	-1.322975
18	6	0	2.976840	-0.393527	1.321900
19	6	0	3.358060	-1.125382	-0.939480
20	1	0	1.883937	-0.510448	-2.362176
21	6	0	3.751111	-1.077349	0.390238
22	1	0	3.297106	-0.357021	2.355234
23	1	0	3.957054	-1.647231	-1.672887
24	1	0	4.663140	-1.565943	0.706127
25	6	0	0.985965	0.978046	2.002488
26	1	0	0.783430	2.005033	1.700991
27	1	0	0.019652	0.494058	2.158076
28	1	0	1.508902	0.991372	2.956597

15_(P_V)_method_B_water.log

Input orientation:

Center	Atomic	Atomic	Coordinates (Angstroms)		
Number	Number	Type	X	Y	Z
1	15	0	-0.094928	0.988338	-1.024854
2	1	0	-0.046978	0.605985	-2.380652
3	8	0	-0.188588	2.464734	-0.819454
4	6	0	-1.532373	0.089850	-0.380144
5	6	0	-2.605014	0.810780	0.144763
6	6	0	-1.596445	-1.305204	-0.431084
7	6	0	-3.730050	0.143708	0.618097
8	1	0	-2.552952	1.889819	0.182892
9	6	0	-2.722051	-1.968805	0.039464

10	1	0	-0.770449	-1.876486	-0.834487
11	6	0	-3.789018	-1.244561	0.565388
12	1	0	-4.557644	0.707488	1.026518
13	1	0	-2.766666	-3.048485	-0.001772
14	1	0	-4.663855	-1.763225	0.933505
15	6	0	1.406230	0.189017	-0.403648
16	6	0	1.783027	0.235322	0.954245
17	6	0	2.216215	-0.467369	-1.334163
18	6	0	2.971686	-0.391893	1.323574
19	6	0	3.395425	-1.089233	-0.942179
20	1	0	1.925296	-0.492762	-2.376261
21	6	0	3.770999	-1.049171	0.393345
22	1	0	3.277237	-0.363255	2.361429
23	1	0	4.011700	-1.593588	-1.673311
24	1	0	4.687401	-1.525173	0.715348
25	6	0	0.945478	0.926613	1.998356
26	1	0	0.715599	1.951859	1.710249
27	1	0	-0.006582	0.412521	2.143130
28	1	0	1.462103	0.945213	2.955493

15_(P_V)_method_D_DCM.log

Input orientation:

Center Number	Atomic Number	Atomic Type	Coordinates (Angstroms) X	Y	Z
1	15	0	-0.076918	1.179368	-0.916592
2	1	0	-0.023374	0.987766	-2.316910
3	8	0	-0.177574	2.632633	-0.503473
4	6	0	-1.500181	0.183854	-0.375387
5	6	0	-2.615973	0.831425	0.168820
6	6	0	-1.492963	-1.212881	-0.503214
7	6	0	-3.720598	0.084310	0.586042
8	1	0	-2.607571	1.911663	0.272150
9	6	0	-2.599112	-1.955587	-0.088515
10	1	0	-0.624241	-1.719970	-0.913164
11	6	0	-3.712857	-1.307236	0.456691
12	1	0	-4.583139	0.587196	1.012085
13	1	0	-2.591028	-3.036735	-0.185229
14	1	0	-4.570916	-1.887250	0.782202
15	6	0	1.404609	0.272531	-0.389721
16	6	0	1.705683	0.077821	0.978108
17	6	0	2.251736	-0.238574	-1.383680
18	6	0	2.865835	-0.636707	1.298172
19	6	0	3.401943	-0.951980	-1.041738
20	1	0	2.012765	-0.079286	-2.431458
21	6	0	3.706293	-1.149167	0.305515
22	1	0	3.113993	-0.793403	2.344053
23	1	0	4.051374	-1.343414	-1.817994
24	1	0	4.599016	-1.698769	0.587992
25	6	0	0.810993	0.602564	2.074583
26	1	0	0.579236	1.661262	1.928673
27	1	0	-0.142690	0.063204	2.094821
28	1	0	1.286352	0.482617	3.050708

16_(P_III)_method_A.log

Input orientation:

Center Number	Atomic Number	Atomic Type	Coordinates (Angstroms) X	Y	Z
1	15	0	0.003848	1.739787	0.605670
2	8	0	-0.113426	2.633118	-0.826612
3	1	0	0.130898	3.553308	-0.664153
4	6	0	1.421512	0.617498	0.220640
5	6	0	1.515310	-0.117932	-0.974256
6	6	0	2.462903	0.525317	1.155920
7	6	0	2.621465	-0.925123	-1.232556
8	1	0	0.719508	-0.056417	-1.710491
9	6	0	3.575219	-0.289944	0.921689
10	1	0	2.409822	1.097048	2.078964
11	6	0	3.627501	-0.995703	-0.272006
12	1	0	2.711812	-1.496560	-2.150295
13	6	0	-1.417479	0.602919	0.295330
14	6	0	-1.581039	-0.525378	1.117458
15	6	0	-2.391002	0.878455	-0.676021
16	6	0	-2.679827	-1.371464	0.969051
17	1	0	-0.842121	-0.758168	1.880296
18	6	0	-3.498604	0.039921	-0.838639
19	1	0	-2.276801	1.747690	-1.314435
20	6	0	-3.618610	-1.067627	-0.011128
21	1	0	-2.813767	-2.247901	1.593914
22	1	0	-4.255454	0.238204	-1.590230
23	1	0	4.385486	-0.374272	1.637802
24	9	0	4.703892	-1.787288	-0.516482
25	9	0	-4.693962	-1.886908	-0.161246

16_(P_III)_method_A_DCM.log

Input orientation:

Center Number	Atomic Number	Atomic Type	Coordinates (Angstroms) X	Y	Z
1	15	0	-0.011989	1.343975	-0.775614
2	8	0	0.111050	2.457719	0.490342
3	1	0	-0.051478	3.352556	0.160539
4	6	0	-1.429939	0.304589	-0.204276
5	6	0	-1.470109	-0.320637	1.055109
6	6	0	-2.530941	0.168521	-1.063945
7	6	0	-2.583413	-1.060240	1.452689
8	1	0	-0.627927	-0.231528	1.734669
9	6	0	-3.652849	-0.578768	-0.689505
10	1	0	-2.518646	0.648831	-2.038548
11	6	0	-3.648802	-1.173003	0.564113
12	1	0	-2.630079	-1.545344	2.421869
13	6	0	1.411629	0.261551	-0.312601
14	6	0	1.551867	-0.988025	-0.942279
15	6	0	2.412247	0.689091	0.572757
16	6	0	2.654135	-1.804350	-0.685574
17	1	0	0.794127	-1.339924	-1.637560
18	6	0	3.524226	-0.116321	0.842891
19	1	0	2.320160	1.652909	1.061241
20	6	0	3.617303	-1.345103	0.205734
21	1	0	2.768170	-2.772064	-1.162075
22	1	0	4.300299	0.201839	1.530990
23	1	0	-4.507365	-0.694486	-1.347352
24	9	0	-4.736561	-1.901723	0.944971
25	9	0	4.700279	-2.137345	0.461794

16_(P_III)_method_A_DCM_smd.log

Input orientation:

Center Number	Atomic Number	Atomic Type	Coordinates (Angstroms) X	Y	Z
1	15	0	0.030024	1.741325	0.507531
2	8	0	-0.008804	2.573069	-0.963434
3	1	0	0.052708	3.527605	-0.802153
4	6	0	1.432526	0.577327	0.199095
5	6	0	1.422558	-0.407455	-0.805297
6	6	0	2.580632	0.723388	0.994692
7	6	0	2.533401	-1.224073	-1.019238
8	1	0	0.544300	-0.544027	-1.429156
9	6	0	3.702259	-0.089665	0.801675
10	1	0	2.605996	1.478268	1.776287
11	6	0	3.646062	-1.042405	-0.204941
12	1	0	2.541110	-1.986460	-1.791770
13	6	0	-1.424643	0.630080	0.255270
14	6	0	-1.732504	-0.310176	1.254650
15	6	0	-2.274937	0.744464	-0.854378
16	6	0	-2.851419	-1.137324	1.144178
17	1	0	-1.095953	-0.406544	2.130942
18	6	0	-3.403823	-0.072978	-0.980517
19	1	0	-2.056049	1.473092	-1.627736
20	6	0	-3.660594	-0.995148	0.023221
21	1	0	-3.096192	-1.868754	1.907481
22	1	0	-4.067142	0.003681	-1.836190
23	1	0	4.593403	0.011635	1.412440
24	9	0	4.734279	-1.844608	-0.405131
25	9	0	-4.763387	-1.798204	-0.091045

16_(P_III)_method_B.log

Input orientation:

Center Number	Atomic Number	Atomic Type	Coordinates (Angstroms) X	Y	Z
1	15	0	0.004670	1.738710	0.571874
2	8	0	-0.096816	2.619097	-0.841349
3	1	0	0.122174	3.539572	-0.676002
4	6	0	1.414566	0.617385	0.206996
5	6	0	1.524565	-0.111041	-0.982489
6	6	0	2.435676	0.515319	1.152206
7	6	0	2.624097	-0.919080	-1.224908
8	1	0	0.744169	-0.044400	-1.728425
9	6	0	3.541434	-0.301174	0.934436
10	1	0	2.370042	1.082084	2.072249
11	6	0	3.611647	-1.000202	-0.254685
12	1	0	2.724529	-1.484837	-2.140097
13	6	0	-1.408832	0.606403	0.275411
14	6	0	-1.552371	-0.525079	1.085050
15	6	0	-2.400877	0.884431	-0.665522
16	6	0	-2.645444	-1.368799	0.954129
17	1	0	-0.799156	-0.761116	1.826290
18	6	0	-3.503497	0.048050	-0.811081
19	1	0	-2.308019	1.756070	-1.296539
20	6	0	-3.604310	-1.063081	0.003035
21	1	0	-2.760892	-2.247714	1.571805
22	1	0	-4.273460	0.251420	-1.541572
23	1	0	4.335626	-0.391349	1.661295
24	9	0	4.678637	-1.790063	-0.483476
25	9	0	-4.671072	-1.878028	-0.130446

16_(P_III)_method_B_DCM.log

Input orientation:

Center Number	Atomic Number	Atomic Type	Coordinates (Angstroms) X	Y	Z
1	15	0	0.017189	1.758525	0.486402
2	8	0	-0.069082	2.580223	-0.958761
3	1	0	0.090269	3.518928	-0.821253
4	6	0	1.421983	0.615295	0.174459
5	6	0	1.517683	-0.183478	-0.970682
6	6	0	2.453629	0.569510	1.113198
7	6	0	2.615181	-1.005250	-1.178136
8	1	0	0.729312	-0.165908	-1.710961
9	6	0	3.557919	-0.258200	0.931118
10	1	0	2.399693	1.186930	2.000383
11	6	0	3.612255	-1.025539	-0.215463
12	1	0	2.702661	-1.624459	-2.059345
13	6	0	-1.404026	0.620708	0.245165
14	6	0	-1.544739	-0.481198	1.096030
15	6	0	-2.406617	0.874294	-0.691947
16	6	0	-2.645818	-1.321318	1.007973
17	1	0	-0.785749	-0.697598	1.837042
18	6	0	-3.517603	0.041798	-0.795639
19	1	0	-2.319280	1.723769	-1.353192
20	6	0	-3.612461	-1.039072	0.058215
21	1	0	-2.757653	-2.176950	1.658160
22	1	0	-4.294418	0.227662	-1.523533
23	1	0	4.358982	-0.303779	1.654530
24	9	0	4.680715	-1.830469	-0.409657
25	9	0	-4.690487	-1.853469	-0.034519

16_(P_III)_method_B_DCM_smd.log

Input orientation:

Center Number	Atomic Number	Atomic Type	Coordinates (Angstroms) X	Y	Z
1	15	0	0.019128	1.740768	0.511468
2	8	0	-0.060579	2.594013	-0.914011
3	1	0	0.062765	3.536238	-0.748137
4	6	0	1.426027	0.606110	0.177036
5	6	0	1.515274	-0.186144	-0.973310
6	6	0	2.466588	0.562902	1.106489
7	6	0	2.616559	-0.999660	-1.195326
8	1	0	0.720292	-0.171928	-1.706972
9	6	0	3.574966	-0.255931	0.909283
10	1	0	2.417452	1.175066	1.997885
11	6	0	3.621365	-1.016178	-0.241730
12	1	0	2.699639	-1.614575	-2.080553
13	6	0	-1.408106	0.612823	0.257294
14	6	0	-1.581017	-0.462115	1.136520
15	6	0	-2.379606	0.845002	-0.717325
16	6	0	-2.683911	-1.298978	1.039307
17	1	0	-0.847159	-0.657019	1.908479
18	6	0	-3.492874	0.016225	-0.830031
19	1	0	-2.269108	1.674702	-1.400434
20	6	0	-3.617990	-1.037694	0.052315
21	1	0	-2.822023	-2.133993	1.711636
22	1	0	-4.247063	0.185139	-1.585976
23	1	0	4.383957	-0.299810	1.624744
24	9	0	4.695666	-1.814529	-0.450239
25	9	0	-4.700703	-1.849473	-0.048827

16_(P_III)_method_D_DCM.log

Input orientation:

Center Number	Atomic Number	Atomic Type	Coordinates (Angstroms) X	Y	Z
1	15	0	0.012438	1.772371	0.585158
2	8	0	-0.059325	2.639205	-0.864231
3	1	0	0.094749	3.578760	-0.694818
4	6	0	1.406535	0.618123	0.240932
5	6	0	1.389194	-0.299184	-0.823832
6	6	0	2.539442	0.684054	1.064666
7	6	0	2.480822	-1.129956	-1.068844
8	1	0	0.515843	-0.369494	-1.464186
9	6	0	3.641355	-0.147275	0.842218
10	1	0	2.565395	1.388293	1.891182
11	6	0	3.582032	-1.033021	-0.223776
12	1	0	2.484659	-1.841654	-1.887025
13	6	0	-1.401235	0.633956	0.276717
14	6	0	-1.540478	-0.512589	1.076926
15	6	0	-2.382334	0.917290	-0.682678
16	6	0	-2.629471	-1.369310	0.919568
17	1	0	-0.789430	-0.751929	1.824533
18	6	0	-3.480264	0.068347	-0.855735
19	1	0	-2.281980	1.798178	-1.306498
20	6	0	-3.576628	-1.055149	-0.048236
21	1	0	-2.744589	-2.260189	1.526828
22	1	0	-4.242354	0.271780	-1.599993
23	1	0	4.521633	-0.110813	1.474081
24	9	0	4.649234	-1.849291	-0.453903
25	9	0	-4.645706	-1.889544	-0.209468

16_(P_III)_method_E_DCM.log

Input orientation:

Center Number	Atomic Number	Atomic Type	Coordinates (Angstroms) X	Y	Z
1	15	0	-0.015254	1.448313	-0.691774
2	8	0	0.112863	2.470740	0.595928
3	1	0	-0.015381	3.378790	0.319865
4	6	0	-1.401641	0.377903	-0.172833
5	6	0	-1.481066	-0.169626	1.108319
6	6	0	-2.406569	0.092377	-1.089853
7	6	0	-2.539853	-0.980350	1.471055
8	1	0	-0.703173	0.036706	1.832286
9	6	0	-3.471384	-0.733149	-0.751925
10	1	0	-2.361740	0.512671	-2.086688
11	6	0	-3.514260	-1.248216	0.525177
12	1	0	-2.616033	-1.406110	2.461297
13	6	0	1.371531	0.323951	-0.298411
14	6	0	1.425341	-0.922347	-0.922267
15	6	0	2.407707	0.693432	0.551729
16	6	0	2.479907	-1.789449	-0.698314
17	1	0	0.628592	-1.233428	-1.587222
18	6	0	3.473397	-0.164097	0.791152
19	1	0	2.382798	1.656479	1.041977
20	6	0	3.486367	-1.388833	0.159657
21	1	0	2.525416	-2.759705	-1.171735
22	1	0	4.278105	0.111562	1.457775
23	1	0	-4.253828	-0.967397	-1.459208
24	9	0	-4.539355	-2.042020	0.868501
25	9	0	4.512903	-2.225478	0.385893

16_(P_III)_method_E_DCM_smd.log

Input orientation:

Center Number	Atomic Number	Atomic Type	Coordinates (Angstroms) X	Y	Z
1	15	0	0.004859	1.782255	0.598676
2	8	0	-0.089348	2.680704	-0.779099
3	1	0	-0.000157	3.615558	-0.577976
4	6	0	1.395609	0.663634	0.207892
5	6	0	1.488677	-0.015014	-1.008124
6	6	0	2.391337	0.479448	1.160927
7	6	0	2.553447	-0.855965	-1.272988
8	1	0	0.718531	0.109665	-1.759048
9	6	0	3.461599	-0.373359	0.922204
10	1	0	2.335689	1.001511	2.108152
11	6	0	3.517109	-1.018229	-0.293597
12	1	0	2.641211	-1.383336	-2.212625
13	6	0	-1.385979	0.633099	0.302131
14	6	0	-1.494951	-0.498306	1.111030
15	6	0	-2.365276	0.871709	-0.655378
16	6	0	-2.547051	-1.384566	0.962945
17	1	0	-0.744118	-0.701854	1.865381
18	6	0	-3.429327	-0.006154	-0.819283
19	1	0	-2.299923	1.745237	-1.289314
20	6	0	-3.495005	-1.115663	-0.005378
21	1	0	-2.635653	-2.267008	1.581068
22	1	0	-4.192383	0.166430	-1.565595
23	1	0	4.237763	-0.529618	1.658173
24	9	0	4.549753	-1.841367	-0.541673
25	9	0	-4.522140	-1.971868	-0.157760

16_(P_V)_method_A.log

Input orientation:

Center Number	Atomic Number	Atomic Type	Coordinates (Angstroms) X	Y	Z
1	15	0	0.003293	1.660556	0.100839
2	1	0	-0.095076	2.150458	1.430435
3	8	0	0.117421	2.707963	-0.970923
4	6	0	-1.476632	0.601378	0.021258
5	6	0	-2.250925	0.351792	1.163019
6	6	0	-1.862676	0.044649	-1.209304
7	6	0	-3.387065	-0.458324	1.092253
8	1	0	-1.979550	0.795625	2.117535
9	6	0	-2.995020	-0.763109	-1.297935
10	1	0	-1.283201	0.254789	-2.103450
11	6	0	-3.730528	-0.999573	-0.140200
12	1	0	-4.000812	-0.661314	1.963037
13	1	0	-3.313211	-1.200076	-2.238167
14	6	0	1.457049	0.554892	0.149139
15	6	0	1.509475	-0.598383	0.947266
16	6	0	2.566251	0.906512	-0.633804
17	6	0	2.654841	-1.394373	0.969131
18	1	0	0.653914	-0.891070	1.549824
19	6	0	3.718483	0.117478	-0.625521

20	1	0	2.514568	1.797311	-1.252423
21	6	0	3.735599	-1.015199	0.178959
22	1	0	2.716973	-2.291569	1.575286
23	1	0	4.586438	0.368164	-1.225648
24	9	0	4.850843	-1.787721	0.194155
25	9	0	-4.833064	-1.784737	-0.220576

16_(P_V)_method_A_DCM.log

Input orientation:

Center Number	Atomic Number	Atomic Type	Coordinates (Angstroms) X	Y	Z
1	15	0	-0.033858	1.113990	-0.861955
2	1	0	0.017671	0.863118	-2.253203
3	8	0	-0.121612	2.580425	-0.497805
4	6	0	1.460732	0.250191	-0.289280
5	6	0	2.418418	-0.187680	-1.216772
6	6	0	1.693163	0.064775	1.084939
7	6	0	3.592809	-0.811301	-0.788085
8	1	0	2.257823	-0.045835	-2.281631
9	6	0	2.861010	-0.553069	1.529082
10	1	0	0.963685	0.399335	1.816564
11	6	0	3.783183	-0.977078	0.577231
12	1	0	4.342738	-1.157333	-1.490696
13	1	0	3.057697	-0.706955	2.584311
14	6	0	-1.471307	0.160462	-0.273152
15	6	0	-1.569260	-1.225317	-0.478266
16	6	0	-2.512116	0.842448	0.372773
17	6	0	-2.692804	-1.927594	-0.042454
18	1	0	-0.769807	-1.768387	-0.974194
19	6	0	-3.641705	0.152483	0.820115
20	1	0	-2.435870	1.913912	0.526986
21	6	0	-3.702445	-1.216998	0.598546
22	1	0	-2.788465	-2.997541	-0.190622
23	1	0	-4.455871	0.660600	1.325099
24	9	0	-4.800176	-1.897968	1.028520
25	9	0	4.923316	-1.583121	1.005825

16_(P_V)_method_A_DCM_smd.log

Input orientation:

Center Number	Atomic Number	Atomic Type	Coordinates (Angstroms) X	Y	Z
1	15	0	-0.011595	1.137657	-0.829464
2	1	0	0.044074	0.912135	-2.224044
3	8	0	-0.079272	2.596145	-0.428090
4	6	0	1.471847	0.246064	-0.264743
5	6	0	2.400740	-0.232278	-1.201306
6	6	0	1.720111	0.074169	1.108344
7	6	0	3.564420	-0.882865	-0.782225
8	1	0	2.224384	-0.100426	-2.265363
9	6	0	2.877322	-0.570609	1.543280
10	1	0	1.011708	0.441726	1.845705
11	6	0	3.769557	-1.033597	0.582060
12	1	0	4.293568	-1.260047	-1.491651
13	1	0	3.087690	-0.714632	2.597911
14	6	0	-1.468666	0.188725	-0.275648
15	6	0	-1.630037	-1.165054	-0.612976
16	6	0	-2.452365	0.838572	0.482756
17	6	0	-2.760126	-1.868538	-0.195372
18	1	0	-0.876043	-1.681546	-1.200638
19	6	0	-3.588245	0.147087	0.912847
20	1	0	-2.331626	1.887043	0.736644
21	6	0	-3.709966	-1.189712	0.560088
22	1	0	-2.905383	-2.914413	-0.444722
23	1	0	-4.360193	0.630789	1.502175
24	9	0	-4.817057	-1.873255	0.973452
25	9	0	4.902101	-1.667582	1.002855

16_(P_V)_method_B.log

Input orientation:

Center Number	Atomic Number	Atomic Type	Coordinates (Angstroms) X	Y	Z
1	15	0	0.002647	1.651261	0.136837
2	1	0	-0.097937	2.111137	1.471316
3	8	0	0.108938	2.705689	-0.897718
4	6	0	-1.466070	0.598565	0.035240
5	6	0	-2.258262	0.350650	1.155797
6	6	0	-1.829763	0.041753	-1.194426
7	6	0	-3.389229	-0.452474	1.065545
8	1	0	-2.002750	0.792326	2.110520
9	6	0	-2.955716	-0.759214	-1.303017
10	1	0	-1.234721	0.246978	-2.074158
11	6	0	-3.712211	-0.992753	-0.165530
12	1	0	-4.015937	-0.651680	1.922546
13	1	0	-3.254298	-1.195384	-2.245147
14	6	0	1.450712	0.559025	0.168517
15	6	0	1.531041	-0.564975	0.993580
16	6	0	2.525424	0.877317	-0.662539
17	6	0	2.666697	-1.361560	0.996051
18	1	0	0.702622	-0.832870	1.636516
19	6	0	3.668230	0.087271	-0.674568
20	1	0	2.455232	1.747682	-1.300259
21	6	0	3.714285	-1.016338	0.157606
22	1	0	2.747728	-2.235712	1.625546
23	1	0	4.509748	0.315971	-1.312255
24	9	0	4.816878	-1.787598	0.153314
25	9	0	-4.805897	-1.768949	-0.265128

16_(P_V)_method_B_DCM.log

Input orientation:

Center Number	Atomic Number	Atomic Type	Coordinates (Angstroms) X	Y	Z
1	15	0	0.005446	1.611592	0.344164
2	1	0	-0.077526	1.916723	1.718439
3	8	0	0.078948	2.803484	-0.547556
4	6	0	-1.457003	0.572449	0.130535
5	6	0	-2.431034	0.517893	1.129049
6	6	0	-1.652484	-0.142453	-1.055982
7	6	0	-3.584175	-0.238651	0.957489
8	1	0	-2.297696	1.068531	2.050678
9	6	0	-2.798044	-0.900132	-1.244394
10	1	0	-0.909061	-0.112604	-1.841119
11	6	0	-3.739990	-0.930467	-0.228858
12	1	0	-4.346383	-0.290719	1.720960
13	1	0	-2.964280	-1.459329	-2.153424

14	6	0	1.460739	0.544077	0.211352
15	6	0	1.584563	-0.619239	0.975525
16	6	0	2.489736	0.908856	-0.657009
17	6	0	2.719097	-1.411189	0.877770
18	1	0	0.794761	-0.917696	1.652131
19	6	0	3.630590	0.123306	-0.771089
20	1	0	2.395356	1.810786	-1.244800
21	6	0	3.718106	-1.019319	0.001940
22	1	0	2.833148	-2.312923	1.461288
23	1	0	4.436115	0.388658	-1.440074
24	9	0	4.823024	-1.787408	-0.099922
25	9	0	-4.854921	-1.668307	-0.406373

16_(P_V)_method_B_DCM_smd.log

Input orientation:

Center Number	Atomic Number	Atomic Type	Coordinates (Angstroms) X	Y	Z
1	15	0	0.004714	1.612473	0.357826
2	1	0	-0.085143	1.902170	1.734190
3	8	0	0.080249	2.810365	-0.526727
4	6	0	-1.458560	0.576058	0.123536
5	6	0	-2.414075	0.474270	1.136001
6	6	0	-1.669159	-0.090851	-1.088039
7	6	0	-3.565456	-0.283173	0.953569
8	1	0	-2.266732	0.988432	2.076816
9	6	0	-2.812942	-0.849038	-1.287523
10	1	0	-0.939479	-0.021498	-1.884295
11	6	0	-3.734881	-0.926527	-0.257156
12	1	0	-4.314703	-0.371804	1.727334
13	1	0	-2.992491	-1.371724	-2.216262
14	6	0	1.463899	0.546422	0.227781
15	6	0	1.615211	-0.577994	1.043803
16	6	0	2.461462	0.868142	-0.692516
17	6	0	2.746207	-1.375369	0.944601
18	1	0	0.850399	-0.840365	1.763189
19	6	0	3.598712	0.076933	-0.808432
20	1	0	2.349394	1.741555	-1.319568
21	6	0	3.711742	-1.026189	0.015689
22	1	0	2.882343	-2.247864	1.567521
23	1	0	4.381180	0.309524	-1.516632
24	9	0	4.815816	-1.801226	-0.087921
25	9	0	-4.850677	-1.666802	-0.446062

16_(P_V)_method_D_DCM.log

Input orientation:

Center Number	Atomic Number	Atomic Type	Coordinates (Angstroms) X	Y	Z
1	15	0	0.004792	1.669108	0.342989
2	1	0	-0.068745	2.003726	1.714795
3	8	0	0.080983	2.866594	-0.578684
4	6	0	-1.446688	0.600850	0.152353
5	6	0	-2.459597	0.606753	1.121518
6	6	0	-1.578939	-0.209157	-0.987630
7	6	0	-3.595454	-0.190081	0.965478
8	1	0	-2.368834	1.229864	2.006033
9	6	0	-2.707580	-1.007196	-1.159757
10	1	0	-0.797866	-0.225754	-1.741318
11	6	0	-3.689476	-0.976588	-0.174704
12	1	0	-4.388328	-0.202975	1.704469
13	1	0	-2.829337	-1.641795	-2.030077
14	6	0	1.445623	0.573699	0.194011
15	6	0	1.494149	-0.660145	0.860511
16	6	0	2.528498	0.985525	-0.593405
17	6	0	2.615415	-1.480187	0.744891
18	1	0	0.655746	-0.991435	1.465566
19	6	0	3.656415	0.172729	-0.723580
20	1	0	2.482459	1.939027	-1.108623
21	6	0	3.670212	-1.040094	-0.047720
22	1	0	2.675170	-2.438279	1.248411
23	1	0	4.504190	0.468010	-1.331249
24	9	0	4.765659	-1.838923	-0.167686
25	9	0	-4.791436	-1.757355	-0.335872

16_(P_V)_method_E_DCM.log

Input orientation:

Center Number	Atomic Number	Atomic Type	Coordinates (Angstroms) X	Y	Z
1	15	0	-0.030784	1.177534	-0.857104
2	1	0	0.018502	0.947314	-2.245511
3	8	0	-0.114312	2.606637	-0.469521
4	6	0	1.432663	0.295628	-0.297934
5	6	0	2.344925	-0.227004	-1.207851
6	6	0	1.659594	0.139386	1.069191
7	6	0	3.473437	-0.902506	-0.769065
8	1	0	2.180787	-0.113666	-2.271707
9	6	0	2.781038	-0.527090	1.524756
10	1	0	0.952711	0.535239	1.787169
11	6	0	3.665668	-1.035194	0.590439
12	1	0	4.192503	-1.314627	-1.461745
13	1	0	2.973649	-0.656941	2.579619
14	6	0	-1.436064	0.205200	-0.287319
15	6	0	-1.485268	-1.170952	-0.498091
16	6	0	-2.482213	0.839473	0.372698
17	6	0	-2.568333	-1.910750	-0.060369
18	1	0	-0.671511	-1.677056	-1.002504
19	6	0	-3.572881	0.112200	0.823849
20	1	0	-2.439840	1.907731	0.536297
21	6	0	-3.590530	-1.248221	0.594450
22	1	0	-2.625711	-2.978702	-0.211951
23	1	0	-4.393319	0.586227	1.342692
24	9	0	-4.639539	-1.959548	1.026203
25	9	0	4.751773	-1.684750	1.025184

16_(P_V)_method_E_DCM_smd.log

Input orientation:

Center Number	Atomic Number	Atomic Type	Coordinates (Angstroms) X	Y	Z
1	15	0	0.011340	1.672300	0.394354
2	1	0	-0.079348	1.940758	1.773041
3	8	0	0.096390	2.871489	-0.475067
4	6	0	-1.432761	0.629405	0.138169
5	6	0	-2.366107	0.444240	1.152350
6	6	0	-1.623233	0.006613	-1.095056
7	6	0	-3.480851	-0.355482	0.949497

8	1	0	-2.228379	0.923032	2.113728
9	6	0	-2.730727	-0.790368	-1.316771
10	1	0	-0.899392	0.138149	-1.889926
11	6	0	-3.635470	-0.952601	-0.283807
12	1	0	-4.217035	-0.510732	1.725406
13	1	0	-2.896465	-1.280500	-2.265640
14	6	0	1.440564	0.583022	0.250144
15	6	0	1.549346	-0.549984	1.053455
16	6	0	2.433381	0.868036	-0.679862
17	6	0	2.638792	-1.393672	0.934842
18	1	0	0.778685	-0.783683	1.777852
19	6	0	3.529971	0.029937	-0.816524
20	1	0	2.349168	1.749088	-1.302153
21	6	0	3.604957	-1.081468	-0.003371
22	1	0	2.743152	-2.277106	1.548571
23	1	0	4.310401	0.231332	-1.536583
24	9	0	4.662405	-1.898340	-0.127831
25	9	0	-4.710457	-1.727670	-0.491431

17_(P_III)_method_A.log

Input orientation:

Center Number	Atomic Number	Atomic Type	Coordinates (Angstroms) X	Y	Z
1	15	0	-0.010745	1.546284	-0.669661
2	8	0	0.115254	2.514892	0.699888
3	1	0	-0.008100	3.445255	0.470615
4	6	0	-1.425568	0.456331	-0.166403
5	6	0	-1.792954	0.284237	1.177803
6	6	0	-2.160523	-0.192019	-1.170780
7	6	0	-2.866157	-0.535851	1.518021
8	1	0	-1.242270	0.800485	1.957109
9	6	0	-3.229657	-1.027056	-0.845543
10	1	0	-1.909730	-0.038722	-2.217140
11	6	0	-3.564107	-1.184086	0.497912
12	1	0	-3.164132	-0.682237	2.549059
13	6	0	1.393590	0.391847	-0.315003
14	6	0	1.619541	-0.688438	-1.185720
15	6	0	2.275151	0.606841	0.754929
16	6	0	2.691092	-1.553714	-0.985668
17	1	0	0.958044	-0.862136	-2.030352
18	6	0	3.355314	-0.250329	0.965936
19	1	0	2.111915	1.443308	1.424964
20	6	0	3.545516	-1.319303	0.092658
21	1	0	2.875382	-2.392296	-1.645700
22	1	0	4.041976	-0.101176	1.790277
23	1	0	-3.805900	-1.537232	-1.607526
24	7	0	-4.699705	-2.056693	0.852593
25	7	0	4.685767	-2.226823	0.309987
26	8	0	-4.983344	-2.170268	2.045628
27	8	0	-5.297066	-2.620628	-0.065190
28	8	0	4.830433	-3.163975	-0.476799
29	8	0	5.428483	-1.996195	1.265527

17_(P_III)_method_A_DCM.log

Input orientation:

Center Number	Atomic Number	Atomic Type	Coordinates (Angstroms) X	Y	Z
1	15	0	-0.010789	1.563760	-0.632625
2	8	0	0.111261	2.508751	0.746119
3	1	0	0.018291	3.446554	0.525715
4	6	0	-1.424913	0.460966	-0.148844
5	6	0	-1.829876	0.316090	1.188148
6	6	0	-2.123546	-0.217683	-1.160143
7	6	0	-2.902186	-0.508804	1.517058
8	1	0	-1.308660	0.853814	1.972582
9	6	0	-3.192298	-1.056188	-0.847057
10	1	0	-1.846253	-0.088513	-2.202343
11	6	0	-3.563902	-1.188004	0.491153
12	1	0	-3.221252	-0.631306	2.544459
13	6	0	1.389407	0.394974	-0.298948
14	6	0	1.591022	-0.695400	-1.164595
15	6	0	2.296844	0.617945	0.748477
16	6	0	2.663598	-1.561648	-0.980714
17	1	0	0.911197	-0.877910	-1.991741
18	6	0	3.377849	-0.240017	0.944777
19	1	0	2.153651	1.460823	1.414530
20	6	0	3.544431	-1.319623	0.076844
21	1	0	2.822875	-2.405356	-1.640024
22	1	0	4.078793	-0.079391	1.754320
23	1	0	-3.735876	-1.585694	-1.619153
24	7	0	-4.692412	-2.061941	0.831100
25	7	0	4.678889	-2.224431	0.276701
26	8	0	-5.015046	-2.162932	2.018607
27	8	0	-5.267127	-2.656784	-0.085724
28	8	0	4.814577	-3.172929	-0.503248
29	8	0	5.448954	-1.998817	1.215989

17_(P_III)_method_A_DCM_smd.log

Input orientation:

Center Number	Atomic Number	Atomic Type	Coordinates (Angstroms) X	Y	Z
1	15	0	-0.016141	1.560268	-0.607558
2	8	0	0.113710	2.501899	0.771235
3	1	0	0.083007	3.444083	0.539409
4	6	0	-1.426114	0.459699	-0.112642
5	6	0	-1.934353	0.444491	1.196002
6	6	0	-2.025608	-0.343812	-1.096987
7	6	0	-3.012838	-0.373061	1.526391
8	1	0	-1.489645	1.073551	1.959413
9	6	0	-3.097745	-1.174475	-0.780869
10	1	0	-1.663541	-0.320494	-2.120936
11	6	0	-3.576195	-1.174698	0.531062
12	1	0	-3.407478	-0.393147	2.535012
13	6	0	1.393128	0.390714	-0.309765
14	6	0	1.654267	-0.613827	-1.258727
15	6	0	2.242242	0.528419	0.799364
16	6	0	2.726227	-1.485458	-1.094732
17	1	0	1.022726	-0.720655	-2.135977
18	6	0	3.323227	-0.333606	0.975369
19	1	0	2.058551	1.308831	1.529295
20	6	0	3.547286	-1.331602	0.025582
21	1	0	2.928711	-2.263633	-1.820163
22	1	0	3.978070	-0.235945	1.832697
23	1	0	-3.561885	-1.798371	-1.534768
24	7	0	-4.710500	-2.037439	0.871352
25	7	0	4.679847	-2.242090	0.204766
26	8	0	-5.139182	-2.012055	2.029693
27	8	0	-5.188740	-2.753158	-0.014997
28	8	0	4.861718	-3.124799	-0.640914

29	8	0	5.404747	-2.089327	1.193815

17_(P_III)_method_B_DCM_smd.log

Input orientation:

Center Number	Atomic Number	Atomic Type	Coordinates (Angstroms) X	Y	Z
1	15	0	0.001130	2.234466	-0.671480
2	8	0	0.049345	3.117115	0.721775
3	1	0	0.011897	4.062369	0.529470
4	6	0	-1.408337	1.101218	-0.308395
5	6	0	-1.960650	0.983701	0.969522
6	6	0	-1.962537	0.372097	-1.364220
7	6	0	-3.036737	0.140431	1.197365
8	1	0	-1.552450	1.552640	1.792116
9	6	0	-3.031988	-0.483195	-1.151421
10	1	0	-1.565731	0.475717	-2.365476
11	6	0	-3.554487	-0.584531	0.131269
12	1	0	-3.465742	0.041591	2.182270
13	6	0	1.398429	1.071850	-0.363550
14	6	0	1.687495	0.096172	-1.324392
15	6	0	2.216113	1.183513	0.763049
16	6	0	2.753398	-0.770384	-1.156847
17	1	0	1.079267	0.008745	-2.214797
18	6	0	3.290993	0.325486	0.943251
19	1	0	2.013909	1.940740	1.505793
20	6	0	3.542394	-0.642455	-0.019310
21	1	0	2.976713	-1.527030	-1.892280
22	1	0	3.922147	0.403308	1.814664
23	1	0	-3.462081	-1.049774	-1.962235
24	7	0	-4.693400	-1.479492	0.365537
25	7	0	4.675369	-1.553957	0.165953
26	8	0	-5.152609	-1.545758	1.498457
27	8	0	-5.130342	-2.118752	-0.582923
28	8	0	4.883025	-2.400330	-0.694734
29	8	0	5.361150	-1.427653	1.172731

17_(P_III)_method_D_DCM.log

Input orientation:

Center Number	Atomic Number	Atomic Type	Coordinates (Angstroms) X	Y	Z
1	15	0	0.010471	2.310133	-0.723580
2	8	0	0.061207	3.166578	0.715162
3	1	0	-0.057990	4.112782	0.552667
4	6	0	-1.392166	1.155113	-0.370254
5	6	0	-1.809336	0.886722	0.942477
6	6	0	-2.043258	0.537182	-1.447746
7	6	0	-2.854645	0.000727	1.181696
8	1	0	-1.315737	1.374449	1.775425
9	6	0	-3.083365	-0.362746	-1.224681
10	1	0	-1.748562	0.760906	-2.468417
11	6	0	-3.471791	-0.614929	0.090453
12	1	0	-3.186507	-0.219372	2.188081
13	6	0	1.379375	1.118208	-0.378515
14	6	0	1.579366	0.036895	-1.254098
15	6	0	2.250845	1.301279	0.704708
16	6	0	2.620760	-0.859653	-1.046969
17	1	0	0.919529	-0.115046	-2.102621
18	6	0	3.299686	0.411179	0.925105
19	1	0	2.100898	2.135260	1.379661
20	6	0	3.468228	-0.657436	0.045136
21	1	0	2.780763	-1.698439	-1.711631
22	1	0	3.974777	0.537191	1.761702
23	1	0	-3.592824	-0.849484	-2.045980
24	7	0	-4.570796	-1.552610	0.335538
25	7	0	4.567886	-1.594960	0.270501
26	8	0	-4.914942	-1.754870	1.503804
27	8	0	-5.101093	-2.097433	-0.637300
28	8	0	4.707458	-2.533171	-0.521086
29	8	0	5.307442	-1.406607	1.241922

17_(P_III)_method_E_DCM.log

Input orientation:

Center Number	Atomic Number	Atomic Type	Coordinates (Angstroms) X	Y	Z
1	15	0	-0.008550	1.631481	-0.670106
2	8	0	0.105568	2.583998	0.659612
3	1	0	-0.027328	3.506015	0.435527
4	6	0	-1.389565	0.518609	-0.194194
5	6	0	-1.630226	0.176970	1.135888
6	6	0	-2.189513	-0.016777	-1.197103
7	6	0	-2.648406	-0.696323	1.462215
8	1	0	-1.014736	0.593950	1.921239
9	6	0	-3.207330	-0.905992	-0.889135
10	1	0	-2.024581	0.259778	-2.230240
11	6	0	-3.416079	-1.227832	0.437499
12	1	0	-2.843624	-0.975826	2.485726
13	6	0	1.351628	0.459845	-0.302127
14	6	0	1.433579	-0.725837	-1.033680
15	6	0	2.324284	0.742284	0.650898
16	6	0	2.452747	-1.627926	-0.806131
17	1	0	0.687920	-0.958819	-1.783247
18	6	0	3.355922	-0.150922	0.888345
19	1	0	2.272700	1.657784	1.221705
20	6	0	3.400898	-1.322830	0.157377
21	1	0	2.517934	-2.551467	-1.359844
22	1	0	4.112228	0.053762	1.630153
23	1	0	-3.832203	-1.333655	-1.657470
24	7	0	-4.493157	-2.167229	0.776412
25	7	0	4.484685	-2.275819	0.412386
26	8	0	-4.689772	-2.406869	1.949749
27	8	0	-5.130135	-2.655988	-0.133635
28	8	0	4.482327	-3.320810	-0.206122
29	8	0	5.330285	-1.973252	1.229726

17_(P_V)_method_A.log

Input orientation:

Center Number	Atomic Number	Atomic Type	Coordinates (Angstroms) X	Y	Z
1	15	0	-0.030947	1.309655	-0.700387
2	1	0	-0.006325	1.237554	-2.117221
3	8	0	-0.111482	2.680799	-0.097423
4	6	0	1.471913	0.362949	-0.265191
5	6	0	2.112209	-0.460759	-1.202035
6	6	0	1.996551	0.488672	1.030464

Center Number	Atomic Number	Atomic Type	X	Y	Z
7	6	0	3.255288	-1.177358	-0.847860
8	1	0	1.733477	-0.540651	-2.217519
9	6	0	3.139269	-0.219131	1.397088
10	1	0	1.519981	1.153078	1.744637
11	6	0	3.744624	-1.044951	0.449955
12	1	0	3.767913	-1.818574	-1.554214
13	1	0	3.563725	-0.137690	2.390115
14	6	0	-1.465515	0.258802	-0.251409
15	6	0	-1.587477	-1.075899	-0.667641
16	6	0	-2.476736	0.848611	0.519316
17	6	0	-2.710781	-1.821891	-0.318579
18	1	0	-0.808165	-1.545966	-1.260667
19	6	0	-3.605750	0.111303	0.878668
20	1	0	-2.371066	1.882797	0.831844
21	6	0	-3.701225	-1.211065	0.451166
22	1	0	-2.828032	-2.853277	-0.627116
23	1	0	-4.400163	0.543834	1.474314
24	7	0	-4.895571	-1.998425	0.825490
25	7	0	4.956563	-1.801044	0.832856
26	8	0	5.375001	-1.662974	1.981464
27	8	0	5.470894	-2.523224	-0.020888
28	8	0	-4.958167	-3.162198	0.429074
29	8	0	-5.753863	-1.442702	1.509841

17_(P_V)_method_A_DCM.log

Input orientation:

Center Number	Atomic Number	Atomic Type	Coordinates (Angstroms) X	Y	Z
1	15	0	-0.030381	1.271392	-0.799836
2	1	0	-0.010845	1.131406	-2.205601
3	8	0	-0.095146	2.688195	-0.285192
4	6	0	1.463345	0.338912	-0.311049
5	6	0	2.223051	-0.340731	-1.274159
6	6	0	1.861232	0.325055	1.036121
7	6	0	3.367332	-1.044944	-0.899838
8	1	0	1.935285	-0.322905	-2.320863
9	6	0	3.002921	-0.370825	1.423217
10	1	0	1.286711	0.859731	1.785754
11	6	0	3.734225	-1.046949	0.444620
12	1	0	3.964771	-1.575056	-1.630422
13	1	0	3.323938	-0.392758	2.456765
14	6	0	-1.467255	0.262228	-0.277039
15	6	0	-1.584375	-1.089590	-0.635988
16	6	0	-2.472288	0.880216	0.478883
17	6	0	-2.699659	-1.825075	-0.243927
18	1	0	-0.809581	-1.579281	-1.217751
19	6	0	-3.593115	0.154525	0.882159
20	1	0	-2.375791	1.926070	0.749953
21	6	0	-3.685397	-1.185936	0.510567
22	1	0	-2.807741	-2.868246	-0.511706
23	1	0	-4.378869	0.614445	1.467771
24	7	0	-4.866804	-1.958261	0.928816
25	7	0	4.941771	-1.787225	0.847510
26	8	0	5.257625	-1.776308	2.039257
27	8	0	5.576184	-2.381874	-0.026837
28	8	0	-4.939661	-3.139856	0.582772
29	8	0	-5.725341	-1.386080	1.604186

17_(P_V)_method_A_DCM_smd.log

Input orientation:

Center Number	Atomic Number	Atomic Type	Coordinates (Angstroms) X	Y	Z
1	15	0	-0.030244	1.227015	-0.846931
2	1	0	-0.005725	1.035430	-2.245917
3	8	0	-0.091859	2.663277	-0.384013
4	6	0	1.464802	0.314179	-0.322776
5	6	0	2.257089	-0.340707	-1.276749
6	6	0	1.836905	0.297573	1.031565
7	6	0	3.410363	-1.021161	-0.887123
8	1	0	1.983308	-0.321099	-2.327535
9	6	0	2.987268	-0.374663	1.434520
10	1	0	1.234645	0.808571	1.776590
11	6	0	3.753115	-1.025144	0.464309
12	1	0	4.029942	-1.531503	-1.613911
13	1	0	3.285342	-0.396538	2.475418
14	6	0	-1.474557	0.239977	-0.298805
15	6	0	-1.647540	-1.090646	-0.711431
16	6	0	-2.428217	0.851835	0.525377
17	6	0	-2.766144	-1.811461	-0.302118
18	1	0	-0.915897	-1.572674	-1.353022
19	6	0	-3.552507	0.141412	0.945305
20	1	0	-2.294927	1.883545	0.833895
21	6	0	-3.700025	-1.179016	0.521959
22	1	0	-2.914288	-2.838329	-0.612631
23	1	0	-4.297854	0.602100	1.581608
24	7	0	-4.885603	-1.933557	0.954827
25	7	0	4.969072	-1.738848	0.883682
26	8	0	5.266393	-1.728201	2.080982
27	8	0	5.634163	-2.315307	0.018787
28	8	0	-5.010223	-3.097243	0.563376
29	8	0	-5.700654	-1.368539	1.688905

17_(P_V)_method_B_DCM_smd.log

Input orientation:

Center Number	Atomic Number	Atomic Type	Coordinates (Angstroms) X	Y	Z
1	15	0	-0.013166	2.050322	-0.469579
2	1	0	0.039440	2.300784	-1.853971
3	8	0	-0.072844	3.253203	0.402949
4	6	0	1.457887	1.011833	-0.231635
5	6	0	2.249780	0.654155	-1.323174
6	6	0	1.814455	0.596163	1.054331
7	6	0	3.386193	-0.121501	-1.142141
8	1	0	1.987988	0.983381	-2.319695
9	6	0	2.947756	-0.175153	1.250692
10	1	0	1.211769	0.875230	1.907725
11	6	0	3.713647	-0.521794	0.144436
12	1	0	4.006858	-0.404004	-1.977571
13	1	0	3.234608	-0.503099	2.237271
14	6	0	-1.464330	0.965981	-0.292712
15	6	0	-1.643911	-0.144045	-1.121622
16	6	0	-2.415741	1.273293	0.679049
17	6	0	-2.765170	-0.945307	-0.983138
18	1	0	-0.913757	-0.389282	-1.881033
19	6	0	-3.543199	0.478400	0.830620
20	1	0	-2.277279	2.137438	1.312952
21	6	0	-3.696638	-0.617320	-0.005603
22	1	0	-2.918945	-1.804725	-1.616313
23	1	0	-4.287820	0.705140	1.577083

24	7	0	-4.892649	-1.463149	0.144801
25	7	0	4.920280	-1.342217	0.346200
26	8	0	5.205330	-1.672332	1.488371
27	8	0	5.575779	-1.652215	-0.638507
28	8	0	-5.016935	-2.420562	-0.605906
29	8	0	-5.701638	-1.166253	1.012257

17_(P_V)_method_D_DCM.log

Input orientation:

Center	Atomic	Atomic	Coordinates (Angstroms)		
Number	Number	Type	X	Y	Z
1	15	0	-0.013819	2.136423	-0.460216
2	1	0	0.033475	2.425168	-1.841558
3	8	0	-0.083685	3.333875	0.454116
4	6	0	1.445001	1.067808	-0.248938
5	6	0	2.280195	0.783468	-1.337000
6	6	0	1.726667	0.532144	1.017807
7	6	0	3.392084	-0.040886	-1.170921
8	1	0	2.071337	1.200834	-2.316690
9	6	0	2.834257	-0.289344	1.198272
10	1	0	1.083975	0.753686	1.863466
11	6	0	3.645786	-0.561815	0.095728
12	1	0	4.049066	-0.273745	-1.998501
13	1	0	3.069037	-0.713948	2.165377
14	6	0	-1.443682	1.017090	-0.278347
15	6	0	-1.511904	-0.191236	-0.987327
16	6	0	-2.478345	1.390834	0.587377
17	6	0	-2.613425	-1.027600	-0.836484
18	1	0	-0.707934	-0.489339	-1.652406
19	6	0	-3.585484	0.559896	0.751738
20	1	0	-2.411980	2.326698	1.130988
21	6	0	-3.631019	-0.633534	0.033748
22	1	0	-2.687533	-1.964191	-1.373045
23	1	0	-4.395880	0.826750	1.417260
24	7	0	-4.796255	-1.514434	0.200067
25	7	0	4.816082	-1.433378	0.278866
26	8	0	5.026592	-1.891659	1.403846
27	8	0	5.527044	-1.662922	-0.701994
28	8	0	-4.830752	-2.559914	-0.453228
29	8	0	-5.681155	-1.164366	0.984138

17_(P_V)_method_E_DCM.log

Input orientation:

Center	Atomic	Atomic	Coordinates (Angstroms)		
Number	Number	Type	X	Y	Z
1	15	0	-0.029598	1.367499	-0.765030
2	1	0	-0.012018	1.279405	-2.168652
3	8	0	-0.101050	2.733188	-0.202131
4	6	0	1.438943	0.420487	-0.304990
5	6	0	2.066708	-0.414625	-1.222432
6	6	0	1.914990	0.510280	1.000274
7	6	0	3.157121	-1.176829	-0.839227
8	1	0	1.714210	-0.476941	-2.243481
9	6	0	3.007052	-0.239476	1.394893
10	1	0	1.435157	1.169769	1.710403
11	6	0	3.602505	-1.073345	0.464722
12	1	0	3.654798	-1.833399	-1.535194
13	1	0	3.393120	-0.182753	2.400350
14	6	0	-1.428082	0.320520	-0.287667
15	6	0	-1.507886	-1.005349	-0.704677
16	6	0	-2.423924	0.863989	0.514197
17	6	0	-2.579508	-1.790381	-0.325613
18	1	0	-0.734336	-1.438781	-1.324500
19	6	0	-3.503095	0.087964	0.904347
20	1	0	-2.353053	1.894717	0.832024
21	6	0	-3.556929	-1.223873	0.474545
22	1	0	-2.659989	-2.820168	-0.635570
23	1	0	-4.285641	0.490029	1.528263
24	7	0	-4.700132	-2.055579	0.884878
25	7	0	4.759435	-1.883404	0.880964
26	8	0	5.145646	-1.772899	2.024973
27	8	0	5.261831	-2.618123	0.057344
28	8	0	-4.737808	-3.201494	0.489181
29	8	0	-5.542327	-1.550154	1.595954

18_(P_III)_method_A.log

Input orientation:

Center	Atomic	Atomic	Coordinates (Angstroms)		
Number	Number	Type	X	Y	Z
1	15	0	-0.027093	0.714030	-1.508666
2	8	0	0.125984	2.178370	-0.691253
3	1	0	-0.329386	2.866652	-1.197718
4	6	0	-1.530442	0.025615	-0.603221
5	6	0	-2.839459	0.369426	-0.995312
6	6	0	-2.541186	-1.422806	1.094994
7	6	0	-3.981291	-0.157603	-0.394358
8	6	0	-3.808322	-1.046424	0.655871
9	1	0	-2.441719	-2.117695	1.920066
10	6	0	1.370350	-0.123190	-0.601226
11	6	0	1.845929	-1.404944	-0.945203
12	6	0	3.001549	-1.965663	-0.406736
13	6	0	3.288162	0.060200	0.906421
14	6	0	3.703712	-1.215748	0.525815
15	1	0	3.334005	-2.951070	-0.705819
16	1	0	3.862924	0.614902	1.638317
17	1	0	-4.969310	0.124062	-0.733751
18	7	0	-5.002974	-1.601131	1.325471
19	7	0	4.924513	-1.790733	1.127533
20	8	0	-4.814864	-2.395160	2.245999
21	8	0	-6.103483	-1.232787	0.920453
22	8	0	5.256698	-2.919164	0.769998
23	8	0	5.527310	-1.100632	1.948357
24	7	0	-3.081438	1.335813	-2.084209
25	7	0	1.109323	-2.213282	-1.923672
26	8	0	-0.005073	-1.786572	-2.260459
27	8	0	1.621216	-3.240591	-2.356314
28	8	0	-2.155573	2.101413	-2.383505
29	8	0	-4.178400	1.334284	-2.630127
30	6	0	-1.424325	-0.890828	0.455404
31	1	0	-0.443972	-1.195580	0.801411
32	6	0	2.137855	0.597607	0.332948
33	1	0	1.819958	1.596113	0.605855

18_(P_III)_method_A_DCM.log

Input orientation:

Center Number	Atomic Number	Atomic Type	Coordinates (Angstroms) X	Y	Z
1	15	0	-0.035473	0.792346	-1.450821
2	8	0	0.076908	2.226614	-0.581665
3	1	0	-0.122863	2.977320	-1.159949
4	6	0	-1.531735	0.063976	-0.573186
5	6	0	-2.844863	0.332949	-1.010277
6	6	0	-2.516610	-1.370599	1.150121
7	6	0	-3.975239	-0.228258	-0.421593
8	6	0	-3.787141	-1.073109	0.662931
9	1	0	-2.398413	-2.028764	2.001657
10	6	0	1.359279	-0.086640	-0.579756
11	6	0	1.823781	-1.361740	-0.964771
12	6	0	2.973288	-1.948975	-0.443428
13	6	0	3.278110	0.034303	0.931762
14	6	0	3.681598	-1.233479	0.511376
15	1	0	3.295217	-2.927742	-0.771511
16	1	0	3.851554	0.566270	1.680514
17	1	0	-4.965001	-0.002462	-0.793791
18	7	0	-4.968454	-1.663537	1.313256
19	7	0	4.892806	-1.835588	1.090240
20	8	0	-4.779277	-2.413153	2.272134
21	8	0	-6.078307	-1.376614	0.863140
22	8	0	5.218898	-2.959639	0.705775
23	8	0	5.513934	-1.180281	1.928389
24	7	0	-3.099584	1.249131	-2.133859
25	7	0	1.087276	-2.128260	-1.968530
26	8	0	-0.018648	-1.681676	-2.308840
27	8	0	1.586812	-3.151368	-2.432906
28	8	0	-2.212856	2.059717	-2.424149
29	8	0	-4.175100	1.167075	-2.724189
30	6	0	-1.409125	-0.806182	0.521205
31	1	0	-0.427175	-1.051015	0.906385
32	6	0	2.132801	0.597643	0.374805
33	1	0	1.824779	1.588616	0.682897

18_(P_III)_method_A_DCM_smd.log

Input orientation:

Center Number	Atomic Number	Atomic Type	Coordinates (Angstroms) X	Y	Z
1	15	0	-0.036814	0.840392	-1.391240
2	8	0	0.052000	2.254209	-0.487434
3	1	0	0.005578	3.025616	-1.075676
4	6	0	-1.535854	0.092768	-0.538369
5	6	0	-2.838715	0.286231	-1.040096
6	6	0	-2.534263	-1.286541	1.219732
7	6	0	-3.970413	-0.288785	-0.469131
8	6	0	-3.794198	-1.065657	0.668088
9	1	0	-2.421911	-1.892986	2.110230
10	6	0	1.356762	-0.068660	-0.550784
11	6	0	1.805305	-1.340329	-0.965026
12	6	0	2.957919	-1.945458	-0.471957
13	6	0	3.298025	0.011420	0.933581
14	6	0	3.686171	-1.251953	0.484473
15	1	0	3.267367	-2.920787	-0.822613
16	1	0	3.882691	0.530692	1.683158
17	1	0	-4.952130	-0.120054	-0.891537
18	7	0	-4.977233	-1.663697	1.305460
19	7	0	4.899407	-1.874103	1.033894
20	8	0	-4.800181	-2.362185	2.305147
21	8	0	-6.081293	-1.435266	0.808156
22	8	0	5.211859	-2.994959	0.626874
23	8	0	5.540557	-1.241170	1.875191
24	7	0	-3.078704	1.138546	-2.214403
25	7	0	1.047660	-2.079775	-1.970267
26	8	0	-0.045668	-1.600467	-2.309796
27	8	0	1.513797	-3.116398	-2.441522
28	8	0	-2.253261	2.025699	-2.454892
29	8	0	-4.084887	0.932520	-2.892334
30	6	0	-1.422803	-0.713392	0.604698
31	1	0	-0.448221	-0.900422	1.039137
32	6	0	2.148522	0.592303	0.404267
33	1	0	1.854108	1.579528	0.738355

18_(P_III)_method_D_DCM.log

Input orientation:

Center Number	Atomic Number	Atomic Type	Coordinates (Angstroms) X	Y	Z
1	15	0	0.010677	1.840535	-0.210082
2	8	0	0.032884	2.250174	-1.839439
3	1	0	0.461910	3.110895	-1.951245
4	6	0	1.300961	0.479994	-0.253213
5	6	0	2.669248	0.762333	-0.082458
6	6	0	1.915733	-1.881198	-0.413232
7	6	0	3.653246	-0.221190	-0.053226
8	6	0	3.252637	-1.537251	-0.228119
9	1	0	1.633759	-2.916751	-0.552716
10	6	0	-1.537926	0.819863	-0.286402
11	6	0	-2.123790	0.233008	0.852665
12	6	0	-3.358662	-0.406873	0.834518
13	6	0	-3.503456	0.096636	-1.541142
14	6	0	-4.027684	-0.468733	-0.378696
15	1	0	-3.773379	-0.841846	1.732897
16	1	0	-4.051021	0.026451	-2.472132
17	1	0	4.693135	0.036416	0.089651
18	7	0	4.275651	-2.592639	-0.221535
19	7	0	-5.327145	-1.152338	-0.431885
20	8	0	3.899806	-3.754929	-0.378987
21	8	0	5.449211	-2.256663	-0.058846
22	8	0	-5.763365	-1.643487	0.610160
23	8	0	-5.907664	-1.195382	-1.517513
24	7	0	3.140791	2.145247	0.070145
25	7	0	-1.423233	0.285078	2.132096
26	8	0	-0.255495	0.703287	2.110860
27	8	0	-2.007361	-0.068320	3.154454
28	8	0	2.404284	3.050154	-0.339188
29	8	0	4.237584	2.335734	0.591440
30	6	0	0.958663	-0.870731	-0.414278
31	1	0	-0.077696	-1.150352	-0.551775
32	6	0	-2.272885	0.743959	-1.481373
33	1	0	-1.864126	1.197695	-2.374442

18_(P_V)_method_A.log

Input orientation:

Center Number	Atomic Number	Atomic Type	Coordinates (Angstroms) X	Y	Z
1	15	0	0.018754	0.849735	-0.777511

2	1	0	0.112016	0.577915	-2.156532
3	8	0	-0.076462	2.303803	-0.427575
4	6	0	1.608474	0.087521	-0.206107
5	6	0	2.505986	-0.281188	-1.219777
6	6	0	2.092931	0.055250	1.113199
7	6	0	3.810606	-0.683086	-0.939504
8	1	0	2.179699	-0.264721	-2.253782
9	6	0	3.391948	-0.330848	1.428929
10	6	0	4.231689	-0.700405	0.386456
11	1	0	4.492954	-0.976684	-1.727665
12	1	0	3.732742	-0.341867	2.455851
13	6	0	-1.535004	-0.035862	-0.275159
14	6	0	-1.924266	-1.366781	-0.502395
15	6	0	-3.162486	-1.866418	-0.105810
16	6	0	-3.709032	0.337775	0.767548
17	6	0	-4.037673	-0.993879	0.526401
18	1	0	-3.432143	-2.897764	-0.290808
19	1	0	-4.419142	0.987789	1.263942
20	7	0	-5.361854	-1.502714	0.949742
21	7	0	5.614509	-1.121966	0.702030
22	8	0	5.945864	-1.136712	1.885053
23	8	0	6.337971	-1.428796	-0.243495
24	8	0	-5.612112	-2.685131	0.726353
25	8	0	-6.121994	-0.706214	1.495969
26	7	0	1.224855	0.418001	2.251173
27	8	0	1.748928	0.607373	3.342635
28	8	0	0.015381	0.492269	2.026641
29	6	0	-2.458376	0.803587	0.364684
30	1	0	-2.170625	1.833411	0.548873
31	7	0	-1.027494	-2.305094	-1.196012
32	8	0	0.033258	-1.841214	-1.632952
33	8	0	-1.370101	-3.475333	-1.310055

18_(P_V)_method_A_DCM.log

Input orientation:

Center Number	Atomic Number	Atomic Type	Coordinates (Angstroms) X	Y	Z
1	15	0	0.011332	0.868586	-0.842723
2	1	0	0.104342	0.541823	-2.205867
3	8	0	-0.102661	2.345614	-0.574779
4	6	0	1.605418	0.142429	-0.238179
5	6	0	2.528169	-0.165518	-1.249281
6	6	0	2.050582	0.014725	1.090394
7	6	0	3.823352	-0.591579	-0.961369
8	1	0	2.235441	-0.079583	-2.289615
9	6	0	3.334791	-0.412223	1.413763
10	6	0	4.202887	-0.709834	0.371977
11	1	0	4.521611	-0.828330	-1.753947
12	1	0	3.642558	-0.504199	2.446101
13	6	0	-1.526494	-0.012607	-0.290707
14	6	0	-1.882436	-1.364504	-0.444318
15	6	0	-3.107553	-1.871483	-0.020941
16	6	0	-3.715520	0.364779	0.721465
17	6	0	-4.006861	-0.986980	0.559181
18	1	0	-3.348038	-2.917912	-0.148332
19	1	0	-4.439477	1.030210	1.174395
20	7	0	-5.312071	-1.504980	1.010514
21	7	0	5.568571	-1.161235	0.697132
22	8	0	5.873886	-1.258754	1.885394
23	8	0	6.323810	-1.414091	-0.241218
24	8	0	-5.533924	-2.707220	0.866750
25	8	0	-6.104011	-0.703376	1.505273
26	7	0	1.166070	0.333937	2.222691
27	8	0	1.608250	0.222644	3.363503
28	8	0	0.019592	0.700165	1.956308
29	6	0	-2.474975	0.836628	0.295153
30	1	0	-2.223967	1.883551	0.420968
31	7	0	-0.965955	-2.317010	-1.084738
32	8	0	0.078514	-1.857915	-1.560362
33	8	0	-1.275430	-3.504006	-1.124247

18_(P_V)_method_A_DCM_smd.log

Input orientation:

Center Number	Atomic Number	Atomic Type	Coordinates (Angstroms) X	Y	Z
1	15	0	0.011193	0.869447	-0.869956
2	1	0	0.093587	0.517045	-2.227230
3	8	0	-0.102502	2.352670	-0.632934
4	6	0	1.607289	0.156082	-0.255754
5	6	0	2.547467	-0.104248	-1.263888
6	6	0	2.031661	-0.024889	1.073955
7	6	0	3.841295	-0.532186	-0.972941
8	1	0	2.271529	0.022255	-2.305164
9	6	0	3.312867	-0.459659	1.399925
10	6	0	4.199814	-0.706026	0.360205
11	1	0	4.550676	-0.728370	-1.767288
12	1	0	3.605297	-0.594175	2.432568
13	6	0	-1.520610	-0.006381	-0.289569
14	6	0	-1.867648	-1.365240	-0.404951
15	6	0	-3.092842	-1.866980	0.024931
16	6	0	-3.717837	0.386411	0.699391
17	6	0	-4.001362	-0.970850	0.572877
18	1	0	-3.327577	-2.918289	-0.074681
19	1	0	-4.444721	1.065326	1.128055
20	7	0	-5.306730	-1.481955	1.027342
21	7	0	5.561113	-1.163171	0.687899
22	8	0	5.856488	-1.293615	1.876283
23	8	0	6.329553	-1.390741	-0.247282
24	8	0	-5.528533	-2.687491	0.907172
25	8	0	-6.104928	-0.674217	1.503558
26	7	0	1.131684	0.251315	2.203302
27	8	0	1.516794	-0.007820	3.342253
28	8	0	0.029411	0.738518	1.942702
29	6	0	-2.476971	0.852565	0.267801
30	1	0	-2.237250	1.905246	0.367339
31	7	0	-0.944000	-2.329019	-1.013817
32	8	0	0.092808	-1.877803	-1.513849
33	8	0	-1.237214	-3.522004	-1.007915

18_(P_V)_method_D_DCM.log

Input orientation:

Center Number	Atomic Number	Atomic Type	Coordinates (Angstroms) X	Y	Z
1	15	0	-0.002217	0.359855	1.928564
2	1	0	-0.075851	-0.791386	2.728643
3	8	0	0.045731	1.643352	2.711581
4	6	0	-1.535254	0.146064	0.924826
5	6	0	-2.470595	-0.747557	1.466569
6	6	0	-1.909231	0.835621	-0.240638

7	6	0	-3.714832	-0.954253	0.876984
8	1	0	-2.223490	-1.306080	2.361927
9	6	0	-3.137403	0.643115	-0.863452
10	6	0	-4.023414	-0.254455	-0.285233
11	1	0	-4.427896	-1.646757	1.304524
12	1	0	-3.391336	1.183606	-1.764185
13	6	0	1.547236	0.123724	0.949373
14	6	0	1.918543	-0.955979	0.131421
15	6	0	3.139000	-1.007186	-0.533625
16	6	0	3.706674	1.141754	0.457887
17	6	0	4.015350	0.053448	-0.353405
18	1	0	3.394905	-1.850005	-1.159928
19	1	0	4.414653	1.951315	0.577961
20	7	0	5.315903	0.014323	-1.043503
21	7	0	-5.331652	-0.464806	-0.927559
22	8	0	-5.571793	0.161225	-1.959527
23	8	0	-6.108280	-1.256002	-0.393179
24	8	0	5.564155	-0.965657	-1.745680
25	8	0	6.078652	0.965635	-0.877127
26	7	0	-1.009869	1.817413	-0.862321
27	8	0	-1.334989	2.311885	-1.939098
28	8	0	0.027224	2.093947	-0.256457
29	6	0	2.470799	1.166247	1.099701
30	1	0	2.199589	2.010136	1.722963
31	7	0	1.028350	-2.107800	-0.041620
32	8	0	0.015450	-2.145093	0.665898
33	8	0	1.326777	-2.970609	-0.861774

19_(P_III)_method_A.log

Input orientation:

Center	Atomic	Atomic	Coordinates (Angstroms)		
Number	Number	Type	X	Y	Z
1	15	0	-0.002763	1.225858	-0.938440
2	8	0	-0.094988	2.403836	0.231534
3	1	0	0.106099	3.275702	-0.141385
4	6	0	-1.511299	0.268177	-0.283066
5	6	0	-1.581715	-0.620734	0.803731
6	6	0	-2.715481	0.376811	-1.009360
7	6	0	-2.707532	-1.371994	1.130390
8	6	0	-3.870887	-0.343891	-0.725457
9	6	0	-3.846209	-1.212231	0.355165
10	1	0	-2.698999	-2.056788	1.969438
11	6	0	1.479387	0.224879	-0.311562
12	6	0	1.736126	-1.116594	-0.672195
13	6	0	2.523999	0.815187	0.433606
14	6	0	2.842503	-1.851108	-0.247254
15	6	0	3.627777	0.121610	0.911334
16	6	0	3.763839	-1.219106	0.567992
17	1	0	2.968733	-2.883467	-0.546440
18	1	0	4.373011	0.615750	1.522697
19	1	0	-4.760246	-0.228165	-1.331590
20	7	0	-5.062737	-1.983200	0.692695
21	7	0	4.936522	-1.974033	1.056714
22	8	0	-4.993662	-2.745815	1.653239
23	8	0	-6.052586	-1.804638	-0.011437
24	8	0	5.025407	-3.152156	0.721662
25	8	0	5.737780	-1.367033	1.763246
26	7	0	-2.835840	1.323751	-2.143373
27	7	0	-0.442101	-0.814441	1.730694
28	7	0	0.823737	-1.815441	-1.592264
29	7	0	2.583328	2.276016	0.683397
30	8	0	2.465862	2.999009	-0.308873
31	8	0	2.828967	2.652909	1.819285
32	8	0	-0.121926	-1.153169	-2.043635
33	8	0	1.053628	-2.980531	-1.886591
34	8	0	0.068702	0.188671	2.214979
35	8	0	-0.111740	-1.976143	1.967760
36	8	0	-2.177482	2.362544	-2.072066
37	8	0	-3.597191	1.025948	-3.056361

19_(P_III)_method_A_DCM.log

Input orientation:

Center	Atomic	Atomic	Coordinates (Angstroms)		
Number	Number	Type	X	Y	Z
1	15	0	-0.000272	1.202893	-0.946177
2	8	0	-0.040669	2.427675	0.177129
3	1	0	0.045250	3.291062	-0.256683
4	6	0	-1.513917	0.267445	-0.262490
5	6	0	-1.597654	-0.588211	0.851020
6	6	0	-2.708557	0.347951	-1.008698
7	6	0	-2.725123	-1.336784	1.179446
8	6	0	-3.865088	-0.369831	-0.723659
9	6	0	-3.850198	-1.208646	0.379737
10	1	0	-2.726879	-1.988062	2.043919
11	6	0	1.479150	0.214671	-0.314566
12	6	0	1.753272	-1.112672	-0.717939
13	6	0	2.511236	0.789929	0.457379
14	6	0	2.868442	-1.846158	-0.318095
15	6	0	3.625614	0.096252	0.909708
16	6	0	3.780574	-1.228918	0.518255
17	1	0	3.008505	-2.864218	-0.655229
18	1	0	4.364924	0.578021	1.537348
19	1	0	-4.747122	-0.269933	-1.342267
20	7	0	-5.063220	-1.977506	0.715934
21	7	0	4.958559	-1.982134	0.981940
22	8	0	-5.008314	-2.726680	1.689170
23	8	0	-6.050194	-1.820820	0.000144
24	8	0	5.067048	-3.150729	0.615100
25	8	0	5.758120	-1.393450	1.707902
26	7	0	-2.816620	1.260450	-2.167751
27	7	0	-0.483676	-0.732023	1.815741
28	7	0	0.853028	-1.789707	-1.659359
29	7	0	2.547506	2.236849	0.789855
30	8	0	2.615490	3.011895	-0.162853
31	8	0	2.600849	2.550565	1.971729
32	8	0	-0.127594	-1.142818	-2.054831
33	8	0	1.118188	-2.926984	-2.029543
34	8	0	0.071421	0.294645	2.195669
35	8	0	-0.215128	-1.869061	2.200226
36	8	0	-2.194544	2.322440	-2.104977
37	8	0	-3.532387	0.918583	-3.104024

19_(P_III)_method_A_DCM_smd.log

Input orientation:

Center	Atomic	Atomic	Coordinates (Angstroms)		
Number	Number	Type	X	Y	Z
1	15	0	-0.002359	1.205831	-0.930507
2	8	0	-0.021168	2.442105	0.178809
3	1	0	0.045170	3.299605	-0.276775

4	6	0	-1.514929	0.271792	-0.238122
5	6	0	-1.607937	-0.572083	0.884946
6	6	0	-2.701107	0.338272	-0.998962
7	6	0	-2.734575	-1.326850	1.202360
8	6	0	-3.854948	-0.389137	-0.728190
9	6	0	-3.846503	-1.220298	0.381131
10	1	0	-2.745530	-1.963643	2.077833
11	6	0	1.483068	0.215963	-0.321472
12	6	0	1.752528	-1.108550	-0.739469
13	6	0	2.519606	0.783065	0.449017
14	6	0	2.867083	-1.847699	-0.349299
15	6	0	3.633604	0.083537	0.892671
16	6	0	3.782518	-1.239193	0.490609
17	1	0	3.003012	-2.864265	-0.694499
18	1	0	4.376927	0.561110	1.519647
19	1	0	-4.729803	-0.298401	-1.359668
20	7	0	-5.055036	-1.998495	0.705773
21	7	0	4.955943	-1.999302	0.950124
22	8	0	-5.013172	-2.731954	1.692394
23	8	0	-6.031209	-1.868656	-0.031041
24	8	0	5.079160	-3.157160	0.552851
25	8	0	5.742247	-1.431097	1.707481
26	7	0	-2.801618	1.246651	-2.160818
27	7	0	-0.514443	-0.679485	1.877253
28	7	0	0.842370	-1.776466	-1.674256
29	7	0	2.559344	2.227617	0.793301
30	8	0	2.654418	3.009320	-0.152364
31	8	0	2.590569	2.536077	1.977338
32	8	0	-0.142603	-1.128523	-2.058344
33	8	0	1.100509	-2.912644	-2.056727
34	8	0	0.074765	0.353440	2.182939
35	8	0	-0.292316	-1.788227	2.362533
36	8	0	-2.224377	2.332639	-2.076162
37	8	0	-3.469187	0.881081	-3.124126

19_(P_III)_method_D_DCM.log

Input orientation:

Center Number	Atomic Number	Atomic Type	Coordinates (Angstroms) X	Y	Z
1	15	0	-0.100593	-1.841151	0.405776
2	8	0	0.035240	-2.580039	-1.076234
3	1	0	0.132970	-3.538947	-0.969341
4	6	0	-1.481369	-0.647982	-0.110328
5	6	0	-1.366412	0.531524	-0.864588
6	6	0	-2.770819	-0.873154	0.408884
7	6	0	-2.399549	1.445924	-1.044300
8	6	0	-3.841257	-0.001163	0.256341
9	6	0	-3.630049	1.157133	-0.475319
10	1	0	-2.250425	2.347812	-1.623204
11	6	0	1.393707	-0.695865	0.359822
12	6	0	1.516261	0.460296	1.159393
13	6	0	2.540827	-0.978610	-0.406627
14	6	0	2.607152	1.325050	1.126655
15	6	0	3.640116	-0.138691	-0.501522
16	6	0	3.646554	1.019696	0.267401
17	1	0	2.631718	2.205500	1.753489
18	1	0	4.476027	-0.385725	-1.143069
19	1	0	-4.806246	-0.223839	0.690904
20	7	0	-4.745514	2.100483	-0.663228
21	7	0	4.802143	1.926387	0.186687
22	8	0	-4.518977	3.125910	-1.302426
23	8	0	-5.829573	1.802006	-0.166838
24	8	0	4.778085	2.941055	0.880547
25	8	0	5.717488	1.610810	-0.571698
26	7	0	-3.068649	-2.111378	1.154096
27	7	0	-0.122193	0.882087	-1.581208
28	7	0	0.469597	0.789261	2.133783
29	7	0	2.700236	-2.265883	-1.116867
30	8	0	2.578341	-3.282554	-0.433407
31	8	0	3.018244	-2.233959	-2.298351
32	8	0	-0.464492	-0.017171	2.244153
33	8	0	0.576931	1.810109	2.802065
34	8	0	0.404103	0.006716	-2.260525
35	8	0	0.280973	2.039178	-1.473260
36	8	0	-2.461698	-3.128327	0.812978
37	8	0	-3.910217	-2.058593	2.045598

19_(P_V)_method_A.log

Input orientation:

Center Number	Atomic Number	Atomic Type	Coordinates (Angstroms) X	Y	Z
1	15	0	-0.013916	1.127023	-0.668642
2	1	0	-0.068600	1.280045	-2.053848
3	8	0	0.098528	2.335973	0.202323
4	6	0	1.540753	0.117297	-0.333029
5	6	0	2.494331	-0.270264	-1.295312
6	6	0	1.953693	-0.076671	1.000183
7	6	0	3.721806	-0.846082	-0.979320
8	6	0	3.189559	-0.597065	1.368319
9	6	0	4.054059	-0.990967	0.358588
10	1	0	4.403084	-1.161050	-1.759511
11	1	0	3.463084	-0.698169	2.411001
12	6	0	-1.596588	0.172422	-0.291125
13	6	0	-1.784617	-1.217439	-0.316070
14	6	0	-2.680949	0.903097	0.221015
15	6	0	-2.892511	-1.863116	0.223771
16	6	0	-3.809657	0.313351	0.779433
17	6	0	-3.885590	-1.073851	0.784890
18	1	0	-2.977282	-2.942086	0.198616
19	1	0	-4.609581	0.919901	1.186279
20	7	0	-5.076983	-1.728281	1.372597
21	7	0	5.366079	-1.575481	0.718564
22	8	0	5.609557	-1.706269	1.914809
23	8	0	6.112067	-1.886067	-0.205466
24	8	0	-5.107597	-2.955701	1.352344
25	8	0	-5.944569	-0.995418	1.838271
26	7	0	-0.794058	-2.069696	-0.991950
27	7	0	-2.742348	2.379829	0.071669
28	7	0	2.286130	-0.068135	-2.753222
29	7	0	1.060914	0.241363	2.141751
30	8	0	-0.068913	-1.508065	-1.821884
31	8	0	-0.762512	-3.263624	-0.727688
32	8	0	-2.384763	2.815897	-1.020583
33	8	0	-3.215051	3.026530	0.994837
34	8	0	2.666469	-0.962161	-3.496322
35	8	0	1.793079	1.001403	-3.108233
36	8	0	1.568824	0.751750	3.127110
37	8	0	-0.120398	-0.088485	2.027070

19_(P_V)_method_A_DCM.log

Input orientation:

Center	Atomic	Atomic	Coordinates (Angstroms)		
Number	Number	Type	X	Y	Z
1	15	0	-0.058908	0.925407	-0.982736
2	1	0	-0.134239	0.497021	-2.308932
3	8	0	-0.114797	2.401560	-0.751899
4	6	0	1.566769	0.177374	-0.382851
5	6	0	2.598335	-0.098847	-1.304742
6	6	0	1.901136	-0.109211	0.955937
7	6	0	3.824327	-0.657560	-0.961042
8	6	0	3.121432	-0.651597	1.351970
9	6	0	4.065749	-0.923137	0.377517
10	1	0	4.569853	-0.863788	-1.717886
11	1	0	3.319610	-0.846725	2.397106
12	6	0	-1.569915	0.060377	-0.274350
13	6	0	-1.824497	-1.327407	-0.260005
14	6	0	-2.607771	0.846759	0.252443
15	6	0	-2.965832	-1.903410	0.287890
16	6	0	-3.767138	0.321028	0.814593
17	6	0	-3.918850	-1.057631	0.830783
18	1	0	-3.101494	-2.976347	0.281143
19	1	0	-4.534874	0.966540	1.223905
20	7	0	-5.137995	-1.638925	1.429002
21	7	0	5.367491	-1.496402	0.775145
22	8	0	5.538524	-1.737651	1.967520
23	8	0	6.192141	-1.694229	-0.113968
24	8	0	-5.240938	-2.863344	1.427387
25	8	0	-5.968032	-0.859245	1.890774
26	7	0	-0.868304	-2.269670	-0.868932
27	7	0	-2.601815	2.338887	0.221875
28	7	0	2.467954	0.232983	-2.743358
29	7	0	0.961250	0.178006	2.061505
30	8	0	0.083142	-1.779964	-1.488216
31	8	0	-1.065343	-3.471080	-0.748679
32	8	0	-2.966533	2.855837	-0.827805
33	8	0	-2.346868	2.920922	1.267230
34	8	0	2.972275	-0.538616	-3.551259
35	8	0	1.893582	1.284277	-3.026237
36	8	0	1.235415	-0.241304	3.180255
37	8	0	-0.039310	0.840850	1.790151

19_(P_V)_method_A_DCM_smd.log

Input orientation:

Center	Atomic	Atomic	Coordinates (Angstroms)		
Number	Number	Type	X	Y	Z
1	15	0	-0.027656	1.072095	-0.782038
2	1	0	-0.102720	1.062221	-2.174060
3	8	0	0.036011	2.406277	-0.100643
4	6	0	1.545340	0.139560	-0.339276
5	6	0	2.498208	-0.304549	-1.277541
6	6	0	1.959154	0.021272	1.001465
7	6	0	3.711620	-0.891440	-0.934160
8	6	0	3.182651	-0.509768	1.397844
9	6	0	4.032919	-0.982546	0.411232
10	1	0	4.392413	-1.241949	-1.699694
11	1	0	3.451327	-0.561136	2.445466
12	6	0	-1.584898	0.140063	-0.292460
13	6	0	-1.805484	-1.245216	-0.392795
14	6	0	-2.635795	0.859659	0.295920
15	6	0	-2.921249	-1.895796	0.122402
16	6	0	-3.775705	0.263744	0.826102
17	6	0	-3.888320	-1.117065	0.739642
18	1	0	-3.032729	-2.968337	0.028054
19	1	0	-4.558575	0.859786	1.280032
20	7	0	-5.087543	-1.772396	1.295894
21	7	0	5.324017	-1.581228	0.801433
22	8	0	5.590803	-1.625085	2.000694
23	8	0	6.050975	-1.999850	-0.097233
24	8	0	-5.148557	-2.998580	1.228811
25	8	0	-5.950261	-1.051764	1.794008
26	7	0	-0.844370	-2.082017	-1.121726
27	7	0	-2.655602	2.346667	0.325462
28	7	0	2.304983	-0.135180	-2.737288
29	7	0	1.098471	0.472834	2.116845
30	8	0	-0.068117	-1.494113	-1.885334
31	8	0	-0.872866	-3.296114	-0.968945
32	8	0	-2.701512	2.908659	-0.764774
33	8	0	-2.715360	2.892636	1.420132
34	8	0	2.630204	-1.066493	-3.463499
35	8	0	1.875514	0.951241	-3.126814
36	8	0	1.648069	1.004173	3.073942
37	8	0	-0.109828	0.261473	2.024138

19_(P_V)_method_D_DCM.log

Input orientation:

Center	Atomic	Atomic	Coordinates (Angstroms)		
Number	Number	Type	X	Y	Z
1	15	0	0.002702	1.767890	0.173474
2	1	0	-0.134926	2.195503	1.492821
3	8	0	0.189434	2.821099	-0.868191
4	6	0	-1.496430	0.676549	-0.116157
5	6	0	-2.695887	0.902203	0.585825
6	6	0	-1.530479	-0.429106	-0.986096
7	6	0	-3.815583	0.085374	0.494018
8	6	0	-2.628698	-1.274424	-1.117607
9	6	0	-3.757578	-1.000115	-0.365455
10	1	0	-4.705061	0.298691	1.071293
11	1	0	-2.597565	-2.113645	-1.798850
12	6	0	1.481527	0.627302	0.314590
13	6	0	1.541501	-0.581162	1.035193
14	6	0	2.646779	0.943575	-0.397986
15	6	0	2.625261	-1.451971	0.994841
16	6	0	3.753752	0.108237	-0.478589
17	6	0	3.713892	-1.090711	0.219005
18	1	0	2.616080	-2.373590	1.560067
19	1	0	4.624109	0.384642	-1.060025
20	7	0	4.874514	-1.998693	0.148511
21	7	0	-4.935131	-1.879820	-0.490938
22	8	0	-4.847748	-2.833837	-1.259770
23	8	0	-5.922036	-1.600280	0.184962
24	8	0	4.810075	-3.049048	0.782554
25	8	0	5.827082	-1.644301	-0.541779
26	7	0	0.440675	-0.972797	1.931884
27	7	0	2.821476	2.249724	-1.084209
28	7	0	-2.851423	2.074701	1.472038
29	7	0	-0.389757	-0.751952	-1.867716
30	8	0	-0.405968	-0.109008	2.186739
31	8	0	0.428671	-2.108065	2.387771
32	8	0	2.960218	3.223583	-0.352503
33	8	0	2.908859	2.238870	-2.304750
34	8	0	-3.612503	1.972284	2.426616
35	8	0	-2.224330	3.090542	1.168958
36	8	0	-0.311513	-1.891658	-2.313718

37	8	0	0.401302	0.157190	-2.114904

20_(P_III)_method_A.log

Input orientation:

Center	Atomic	Atomic	Coordinates (Angstroms)		
Number	Number	Type	X	Y	Z
1	15	0	-0.043896	1.423153	-0.521747
2	8	0	-0.103612	2.279613	0.914052
3	1	0	0.300807	3.152464	0.811213
4	6	0	-1.472225	0.308256	-0.143278
5	6	0	-1.642782	-0.374904	1.067039
6	6	0	-2.502165	0.191630	-1.081297
7	6	0	-2.778138	-1.131988	1.336740
8	6	0	-3.648276	-0.562957	-0.840842
9	6	0	-3.785000	-1.224998	0.376264
10	6	0	1.404597	0.276683	-0.197306
11	6	0	1.451068	-1.075348	-0.548122
12	6	0	2.603464	0.824383	0.272230
13	6	0	2.594942	-1.854877	-0.393938
14	6	0	3.763129	0.075124	0.443561
15	6	0	3.757319	-1.277494	0.110522
16	9	0	-2.910649	-1.776925	2.503591
17	9	0	-0.690140	-0.332852	2.011296
18	9	0	-4.878953	-1.949650	0.624580
19	9	0	-4.615738	-0.650815	-1.763267
20	9	0	-2.412541	0.817009	-2.269112
21	9	0	2.675940	2.142966	0.560026
22	9	0	4.882764	0.645882	0.907795
23	9	0	4.863193	-2.012672	0.262190
24	9	0	2.584999	-3.151768	-0.730655
25	9	0	0.364188	-1.685747	-1.066606

20_(P_III)_method_A_DCM.log

Input orientation:

Center	Atomic	Atomic	Coordinates (Angstroms)		
Number	Number	Type	X	Y	Z
1	15	0	0.041454	1.813769	-0.519214
2	8	0	0.091382	2.637007	0.932145
3	1	0	0.367453	3.555342	0.794956
4	6	0	-1.408852	0.730545	-0.106551
5	6	0	-1.543186	0.003676	1.081260
6	6	0	-2.489868	0.685813	-0.990807
7	6	0	-2.686751	-0.729021	1.378931
8	6	0	-3.646171	-0.041105	-0.721531
9	6	0	-3.743307	-0.750392	0.471194
10	6	0	1.446192	0.594029	-0.292286
11	6	0	1.416571	-0.698281	-0.825041
12	6	0	2.668939	0.994472	0.258482
13	6	0	2.502820	-1.565358	-0.769899
14	6	0	3.773018	0.150068	0.331523
15	6	0	3.689810	-1.139645	-0.182690
16	9	0	-2.781308	-1.416324	2.527971
17	9	0	-0.547041	-0.015143	1.984766
18	9	0	-4.848532	-1.451048	0.747390
19	9	0	-4.663649	-0.058972	-1.596451
20	9	0	-2.442496	1.357248	-2.157883
21	9	0	2.833621	2.247089	0.731217
22	9	0	4.921073	0.577745	0.880999
23	9	0	4.746540	-1.959887	-0.124070
24	9	0	2.415197	-2.802754	-1.283568
25	9	0	0.300644	-1.156518	-1.434118

20_(P_III)_method_A_DCM_smd.log

Input orientation:

Center	Atomic	Atomic	Coordinates (Angstroms)		
Number	Number	Type	X	Y	Z
1	15	0	0.039870	1.810516	-0.508537
2	8	0	0.090407	2.631916	0.942442
3	1	0	0.378205	3.549903	0.803717
4	6	0	-1.410536	0.726806	-0.098197
5	6	0	-1.558125	0.016334	1.097440
6	6	0	-2.481524	0.668063	-0.993074
7	6	0	-2.704530	-0.711651	1.392185
8	6	0	-3.639486	-0.055432	-0.727148
9	6	0	-3.750081	-0.746893	0.473769
10	6	0	1.448394	0.592984	-0.290892
11	6	0	1.422035	-0.694118	-0.835255
12	6	0	2.669908	0.989747	0.264013
13	6	0	2.509691	-1.558712	-0.788309
14	6	0	3.775332	0.147647	0.328278
15	6	0	3.694937	-1.136784	-0.196940
16	9	0	-2.812105	-1.383619	2.550796
17	9	0	-0.571673	0.009737	2.013305
18	9	0	-4.859143	-1.444527	0.747966
19	9	0	-4.648008	-0.086421	-1.614011
20	9	0	-2.422030	1.324281	-2.169817
21	9	0	2.833029	2.237846	0.752366
22	9	0	4.923306	0.573442	0.882689
23	9	0	4.754103	-1.956389	-0.145728
24	9	0	2.424646	-2.792168	-1.315281
25	9	0	0.306932	-1.150154	-1.449047

20_(P_III)_method_B.log

Input orientation:

Center	Atomic	Atomic	Coordinates (Angstroms)		
Number	Number	Type	X	Y	Z
1	15	0	0.021467	1.737666	-0.663429
2	8	0	0.079262	2.663839	0.701675
3	1	0	0.433537	3.537326	0.509179
4	6	0	-1.426161	0.709480	-0.170816
5	6	0	-1.571567	0.108269	1.078104
6	6	0	-2.489284	0.579096	-1.059114
7	6	0	-2.716116	-0.586277	1.432651
8	6	0	-3.646366	-0.113644	-0.733365
9	6	0	-3.758024	-0.695302	0.520335
10	6	0	1.426206	0.552323	-0.359506
11	6	0	1.390142	-0.806322	-0.658839
12	6	0	2.663606	1.056267	0.036172
13	6	0	2.494856	-1.633223	-0.521937
14	6	0	3.785869	0.257640	0.187546
15	6	0	3.699534	-1.098567	-0.090598
16	9	0	-2.826054	-1.153670	2.631206
17	9	0	-0.587953	0.170817	1.975936
18	9	0	-4.858461	-1.358763	0.848710

19	9	0	-4.644593	-0.219658	-1.606562
20	9	0	-2.423694	1.124398	-2.276807
21	9	0	2.816076	2.368827	0.265512
22	9	0	4.943761	0.782412	0.580800
23	9	0	4.766291	-1.877110	0.043369
24	9	0	2.408255	-2.931006	-0.803576
25	9	0	0.260916	-1.374903	-1.105438

20_(P_III)_method_B_DCM.log

Input orientation:

Center Number	Atomic Number	Atomic Type	Coordinates (Angstroms) X	Y	Z
1	15	0	-0.029813	0.495311	-1.892363
2	8	0	-0.001111	2.132910	-1.985154
3	1	0	0.091207	2.418749	-2.900529
4	6	0	-1.406123	0.299617	-0.668021
5	6	0	-1.486341	0.995955	0.535617
6	6	0	-2.459255	-0.559757	-0.963633
7	6	0	-2.554279	0.851151	1.404520
8	6	0	-3.540952	-0.727970	-0.112557
9	6	0	-3.586419	-0.016526	1.075484
10	6	0	1.396898	0.120450	-0.758548
11	6	0	1.528922	-1.206622	-0.357680
12	6	0	2.424293	0.978931	-0.376883
13	6	0	2.591315	-1.671452	0.395065
14	6	0	3.507219	0.540805	0.374470
15	6	0	3.592538	-0.785625	0.763067
16	9	0	-2.601986	1.531941	2.549222
17	9	0	-0.513746	1.839452	0.892907
18	9	0	-4.616799	-0.164373	1.899942
19	9	0	-4.532417	-1.560973	-0.426793
20	9	0	-2.459149	-1.265597	-2.099561
21	9	0	2.419737	2.272045	-0.711431
22	9	0	4.468693	1.394729	0.728582
23	9	0	4.626687	-1.207648	1.484446
24	9	0	2.662887	-2.951007	0.761295
25	9	0	0.581424	-2.091395	-0.711743

20_(P_III)_method_B_DMSO.log

Input orientation:

Center Number	Atomic Number	Atomic Type	Coordinates (Angstroms) X	Y	Z
1	15	0	0.022862	1.822109	-0.675348
2	8	0	0.105142	2.844641	0.604227
3	1	0	0.219389	3.752954	0.303329
4	6	0	-1.375864	0.750592	-0.102192
5	6	0	-1.455310	0.181477	1.166340
6	6	0	-2.445769	0.515785	-0.959238
7	6	0	-2.538123	-0.580645	1.569285
8	6	0	-3.542648	-0.245514	-0.585832
9	6	0	-3.586791	-0.793477	0.685694
10	6	0	1.419440	0.636616	-0.348843
11	6	0	1.512223	-0.451646	-1.212559
12	6	0	2.457738	0.778755	0.567262
13	6	0	2.547540	-1.366812	-1.174303
14	6	0	3.514192	-0.120706	0.626585
15	6	0	3.560871	-1.196521	-0.243701
16	9	0	-2.584553	-1.111257	2.791370
17	9	0	-0.466354	0.357992	2.047978
18	9	0	-4.632019	-1.522695	1.059795
19	9	0	-4.550426	-0.451091	-1.433692
20	9	0	-2.447627	1.025236	-2.196335
21	9	0	2.489508	1.787590	1.442890
22	9	0	4.487236	0.043867	1.524678
23	9	0	4.569630	-2.061487	-0.187330
24	9	0	2.581708	-2.397801	-2.018950
25	9	0	0.551559	-0.635605	-2.134380

20_(P_III)_method_B_MeOH.log

Input orientation:

Center Number	Atomic Number	Atomic Type	Coordinates (Angstroms) X	Y	Z
1	15	0	0.011132	1.727867	-0.901758
2	8	0	0.092288	2.901123	0.241388
3	1	0	0.203436	3.765642	-0.169502
4	6	0	-1.384550	0.732820	-0.198333
5	6	0	-1.460136	0.322740	1.130417
6	6	0	-2.456577	0.394710	-1.017416
7	6	0	-2.541219	-0.385193	1.626276
8	6	0	-3.551891	-0.315795	-0.550867
9	6	0	-3.592160	-0.704588	0.778151
10	6	0	1.411148	0.595382	-0.433140
11	6	0	1.503684	-0.593065	-1.152884
12	6	0	2.452406	0.854391	0.453593
13	6	0	2.541400	-1.493980	-1.003153
14	6	0	3.511308	-0.028237	0.622132
15	6	0	3.557586	-1.205361	-0.105373
16	9	0	-2.583877	-0.762945	2.903989
17	9	0	-0.469176	0.606298	1.981249
18	9	0	-4.635750	-1.383199	1.241281
19	9	0	-4.561765	-0.623692	-1.364482
20	9	0	-2.462199	0.749640	-2.307286
21	9	0	2.484888	1.966063	1.194183
22	9	0	4.487132	0.250583	1.488340
23	9	0	4.568712	-2.054147	0.055924
24	9	0	2.575229	-2.623314	-1.710874
25	9	0	0.540452	-0.893829	-2.040647

20_(P_III)_method_B_THF.log

Input orientation:

Center Number	Atomic Number	Atomic Type	Coordinates (Angstroms) X	Y	Z
1	15	0	0.042203	1.811799	-0.520981
2	8	0	0.098041	2.651892	0.892841
3	1	0	0.331178	3.573498	0.737026
4	6	0	-1.397759	0.732758	-0.101613
5	6	0	-1.525665	0.023159	1.089894
6	6	0	-2.469584	0.668187	-0.985896
7	6	0	-2.657043	-0.715587	1.391681
8	6	0	-3.614340	-0.064423	-0.712178
9	6	0	-3.706362	-0.757120	0.484352
10	6	0	1.439862	0.602424	-0.293764
11	6	0	1.407893	-0.669808	-0.858114
12	6	0	2.648053	0.978110	0.290047

13	6	0	2.479147	-1.545640	-0.802856
14	6	0	3.737532	0.123533	0.362555
15	6	0	3.652875	-1.146737	-0.183574
16	9	0	-2.748411	-1.386240	2.539554
17	9	0	-0.538407	0.027430	1.989502
18	9	0	-4.797049	-1.460871	0.763556
19	9	0	-4.622002	-0.104900	-1.582947
20	9	0	-2.425128	1.320317	-2.152819
21	9	0	2.816061	2.207207	0.794095
22	9	0	4.870102	0.520875	0.942907
23	9	0	4.693296	-1.972176	-0.124695
24	9	0	2.392078	-2.760667	-1.344109
25	9	0	0.306983	-1.094666	-1.497734

20_(P_III)_method_B_toluene.log

Input orientation:

Center	Atomic	Atomic	Coordinates (Angstroms)		
Number	Number	Type	X	Y	Z
1	15	0	0.043986	1.803370	-0.453005
2	8	0	0.106351	2.574476	1.002498
3	1	0	0.385329	3.489607	0.894036
4	6	0	-1.409517	0.728086	-0.085806
5	6	0	-1.563899	-0.007473	1.087108
6	6	0	-2.467979	0.698703	-0.987970
7	6	0	-2.710346	-0.735882	1.355655
8	6	0	-3.627218	-0.023745	-0.747832
9	6	0	-3.746834	-0.741547	0.431761
10	6	0	1.440649	0.584177	-0.277537
11	6	0	1.396135	-0.722843	-0.754130
12	6	0	2.677453	1.015986	0.198221
13	6	0	2.489053	-1.574179	-0.712876
14	6	0	3.787745	0.188939	0.256806
15	6	0	3.692230	-1.116759	-0.198919
16	9	0	-2.827412	-1.432931	2.484254
17	9	0	-0.586331	-0.044119	1.994762
18	9	0	-4.850270	-1.436367	0.678081
19	9	0	-4.621193	-0.030112	-1.633780
20	9	0	-2.395824	1.375780	-2.138477
21	9	0	2.844872	2.282828	0.601844
22	9	0	4.946326	0.642857	0.731778
23	9	0	4.749560	-1.919695	-0.155232
24	9	0	2.392609	-2.823087	-1.165913
25	9	0	0.268081	-1.213716	-1.288596

20_(P_III)_method_B_water.log

Input orientation:

Center	Atomic	Atomic	Coordinates (Angstroms)		
Number	Number	Type	X	Y	Z
1	15	0	0.013289	1.728131	-0.904616
2	8	0	0.098153	2.902815	0.236487
3	1	0	0.211881	3.765841	-0.176790
4	6	0	-1.383487	0.736095	-0.198767
5	6	0	-1.466396	0.343657	1.134784
6	6	0	-2.448253	0.381982	-1.020432
7	6	0	-2.548024	-0.361490	1.633105
8	6	0	-3.542999	-0.328114	-0.551993
9	6	0	-3.590972	-0.698431	0.782004
10	6	0	1.410756	0.592685	-0.434763
11	6	0	1.508558	-0.589328	-1.164282
12	6	0	2.444359	0.842620	0.463380
13	6	0	2.545091	-1.491621	-1.015106
14	6	0	3.501814	-0.041717	0.631921
15	6	0	3.554017	-1.211665	-0.106551
16	9	0	-2.598456	-0.720563	2.916029
17	9	0	-0.481727	0.642081	1.987981
18	9	0	-4.634615	-1.375745	1.247197
19	9	0	-4.545615	-0.652300	-1.368426
20	9	0	-2.446513	0.718881	-2.315301
21	9	0	2.470364	1.946357	1.216072
22	9	0	4.470316	0.228243	1.509301
23	9	0	4.563768	-2.062183	0.054955
24	9	0	2.584406	-2.614294	-1.733225
25	9	0	0.551787	-0.882020	-2.061755

20_(P_III)_method_D_DCM.log

Input orientation:

Center	Atomic	Atomic	Coordinates (Angstroms)		
Number	Number	Type	X	Y	Z
1	15	0	0.013281	1.828867	-0.804369
2	8	0	0.069142	2.874201	0.489398
3	1	0	0.317278	3.764138	0.199207
4	6	0	-1.378957	0.770054	-0.190118
5	6	0	-1.418840	0.199303	1.085685
6	6	0	-2.476143	0.532293	-1.019443
7	6	0	-2.492693	-0.568706	1.519771
8	6	0	-3.563758	-0.234611	-0.613485
9	6	0	-3.569658	-0.785825	0.663568
10	6	0	1.403728	0.644956	-0.410468
11	6	0	1.402494	-0.604559	-1.035691
12	6	0	2.528715	0.944206	0.361503
13	6	0	2.424431	-1.531886	-0.882403
14	6	0	3.570954	0.035747	0.533135
15	6	0	3.519500	-1.207287	-0.087470
16	9	0	-2.502162	-1.103111	2.750778
17	9	0	-0.398627	0.376713	1.943198
18	9	0	-4.608542	-1.523021	1.070331
19	9	0	-4.601691	-0.444151	-1.437568
20	9	0	-2.508809	1.043363	-2.264748
21	9	0	2.661492	2.133859	0.976664
22	9	0	4.631477	0.356761	1.291803
23	9	0	4.520060	-2.082812	0.071752
24	9	0	2.368605	-2.724438	-1.495981
25	9	0	0.363104	-0.944149	-1.831742

20_(P_III)_method_E_DCM.log

Input orientation:

Center	Atomic	Atomic	Coordinates (Angstroms)		
Number	Number	Type	X	Y	Z
1	15	0	-0.034007	-0.528213	1.926194
2	8	0	-0.004662	-2.155440	1.980858
3	1	0	0.136920	-2.464170	2.877718
4	6	0	-1.384733	-0.310319	0.698573
5	6	0	-1.430960	-0.966407	-0.525413
6	6	0	-2.438202	0.541970	0.993470

7	6	0	-2.471200	-0.788937	-1.416760
8	6	0	-3.493135	0.741942	0.120255
9	6	0	-3.506749	0.070342	-1.088195
10	6	0	1.375677	-0.131360	0.798516
11	6	0	1.459687	1.176789	0.341619
12	6	0	2.415170	-0.976183	0.437942
13	6	0	2.490586	1.633621	-0.453994
14	6	0	3.466363	-0.546342	-0.356228
15	6	0	3.504772	0.760213	-0.805241
16	9	0	-2.488980	-1.428223	-2.575597
17	9	0	-0.457378	-1.794725	-0.880415
18	9	0	-4.506180	0.247874	-1.930276
19	9	0	-4.482940	1.563765	0.430890
20	9	0	-2.462775	1.208898	2.141126
21	9	0	2.452776	-2.241231	0.835601
22	9	0	4.437497	-1.383429	-0.688707
23	9	0	4.504168	1.173639	-1.563023
24	9	0	2.519262	2.888650	-0.874096
25	9	0	0.502454	2.044173	0.675461

20_(P_III)_method_E_DCM_smd.log

Input orientation:

Center Number	Atomic Number	Atomic Type	Coordinates (Angstroms) X	Y	Z
1	15	0	-0.031296	-1.068813	1.673853
2	8	0	0.018747	-2.637588	1.242696
3	1	0	0.214460	-3.192959	2.003888
4	6	0	-1.385893	-0.521819	0.559776
5	6	0	-1.411824	-0.771348	-0.806364
6	6	0	-2.465745	0.166648	1.090788
7	6	0	-2.455302	-0.358210	-1.610292
8	6	0	-3.524586	0.595109	0.310548
9	6	0	-3.516051	0.328669	-1.045356
10	6	0	1.377617	-0.333169	0.728849
11	6	0	1.437188	1.051613	0.661462
12	6	0	2.443730	-1.020807	0.168861
13	6	0	2.468912	1.733213	0.049964
14	6	0	3.496027	-0.365131	-0.448468
15	6	0	3.509546	1.014729	-0.509604
16	9	0	-2.452256	-0.611329	-2.910461
17	9	0	-0.414339	-1.425419	-1.388728
18	9	0	-4.519579	0.728360	-1.803500
19	9	0	-4.539961	1.253256	0.849091
20	9	0	-2.513129	0.446899	2.388696
21	9	0	2.507510	-2.346935	0.196431
22	9	0	4.493529	-1.056477	-0.981357
23	9	0	4.511326	1.644810	-1.097628
24	9	0	2.473086	3.057047	-0.000006
25	9	0	0.454513	1.772727	1.205333

20_(P_V)_method_A.log

Input orientation:

Center Number	Atomic Number	Atomic Type	Coordinates (Angstroms) X	Y	Z
1	15	0	-0.032235	1.031785	-1.090409
2	1	0	-0.020143	0.495198	-2.390456
3	8	0	-0.067834	2.519231	-0.980657
4	6	0	1.450465	0.224844	-0.360264
5	6	0	2.484594	-0.263245	-1.165467
6	6	0	1.639031	0.154124	1.023757
7	6	0	3.646633	-0.810776	-0.628633
8	6	0	2.787851	-0.388413	1.589903
9	6	0	3.796025	-0.871752	0.755876
10	6	0	-1.462872	0.143040	-0.348392
11	6	0	-1.803669	-1.138696	-0.793852
12	6	0	-2.288500	0.717612	0.626077
13	6	0	-2.910340	-1.829711	-0.315338
14	6	0	-3.405456	0.045962	1.123012
15	6	0	-3.716834	-1.226838	0.649487
16	9	0	-2.028397	1.928177	1.122774
17	9	0	-4.178913	0.614291	2.054618
18	9	0	-4.783154	-1.873155	1.123568
19	9	0	-3.204389	-3.055041	-0.767021
20	9	0	-1.032533	-1.746540	-1.719630
21	9	0	0.685968	0.612249	1.852156
22	9	0	2.933513	-0.449777	2.918149
23	9	0	4.903956	-1.393229	1.284181
24	9	0	4.616293	-1.272576	-1.426193
25	9	0	2.381922	-0.211904	-2.505750

20_(P_V)_method_A_DCM.log

Input orientation:

Center Number	Atomic Number	Atomic Type	Coordinates (Angstroms) X	Y	Z
1	15	0	0.046786	0.432517	1.738293
2	1	0	0.148701	1.822015	1.905099
3	8	0	0.005061	-0.382042	2.997328
4	6	0	1.474816	0.079997	0.641614
5	6	0	2.546466	0.971175	0.525069
6	6	0	1.576488	-1.125601	-0.059449
7	6	0	3.660675	0.688375	-0.258564
8	6	0	2.676251	-1.434396	-0.850331
9	6	0	3.723372	-0.519928	-0.947853
10	6	0	-1.438091	0.286221	0.667495
11	6	0	-1.779241	1.325446	-0.205113
12	6	0	-2.307094	-0.809688	0.715516
13	6	0	-2.924937	1.297017	-0.989968
14	6	0	-3.461695	-0.862846	-0.061327
15	6	0	-3.771676	0.193668	-0.913072
16	9	0	-2.054276	-1.857361	1.510561
17	9	0	-4.275479	-1.925389	0.004779
18	9	0	-4.876995	0.147090	-1.660745
19	9	0	-3.218325	2.312767	-1.813178
20	9	0	-0.974177	2.401712	-0.306454
21	9	0	0.582963	-2.028439	0.018484
22	9	0	2.738817	-2.596370	-1.513582
23	9	0	4.787745	-0.803360	-1.701965
24	9	0	4.670261	1.564104	-0.351113
25	9	0	2.529715	2.145058	1.182127

20_(P_V)_method_A_DCM_smd.log

Input orientation:

Center Number	Atomic Number	Atomic Type	Coordinates (Angstroms) X	Y	Z

1	15	0	0.046552	0.434786	1.745268
2	1	0	0.151135	1.822841	1.917396
3	8	0	-0.000916	-0.387537	3.000138
4	6	0	1.476260	0.076410	0.649616
5	6	0	2.546384	0.967864	0.528929
6	6	0	1.576927	-1.127429	-0.053349
7	6	0	3.656365	0.687484	-0.260238
8	6	0	2.672504	-1.433605	-0.849678
9	6	0	3.716335	-0.517529	-0.953089
10	6	0	-1.439498	0.292647	0.672303
11	6	0	-1.779473	1.328094	-0.204245
12	6	0	-2.307637	-0.803071	0.718270
13	6	0	-2.920239	1.294073	-0.995115
14	6	0	-3.458385	-0.860630	-0.062527
15	6	0	-3.764562	0.190200	-0.920701
16	9	0	-2.055824	-1.851006	1.516222
17	9	0	-4.271647	-1.925712	0.004069
18	9	0	-4.865334	0.137527	-1.677495
19	9	0	-3.211224	2.307537	-1.824735
20	9	0	-0.978826	2.408824	-0.306820
21	9	0	0.584507	-2.033334	0.028187
22	9	0	2.734114	-2.596384	-1.514998
23	9	0	4.777294	-0.798403	-1.715665
24	9	0	4.665838	1.565654	-0.355697
25	9	0	2.535480	2.142441	1.187604

20_(P_V)_method_B.log

Input orientation:

Center Number	Atomic Number	Atomic Type	Coordinates (Angstroms) X	Y	Z
1	15	0	0.044693	0.387656	1.759243
2	1	0	0.134954	1.776477	1.928145
3	8	0	0.020338	-0.426785	2.983571
4	6	0	1.461588	0.073545	0.651639
5	6	0	2.516860	0.974366	0.538197
6	6	0	1.566790	-1.119972	-0.057842
7	6	0	3.626243	0.711314	-0.250871
8	6	0	2.661382	-1.410558	-0.854678
9	6	0	3.695419	-0.487127	-0.948043
10	6	0	-1.430863	0.256376	0.689240
11	6	0	-1.724507	1.274231	-0.215063
12	6	0	-2.329992	-0.806684	0.765265
13	6	0	-2.859672	1.264485	-1.005846
14	6	0	-3.476624	-0.841643	-0.018113
15	6	0	-3.741240	0.196141	-0.900587
16	9	0	-2.116497	-1.828347	1.581168
17	9	0	-4.320823	-1.863650	0.067783
18	9	0	-4.833082	0.165770	-1.650184
19	9	0	-3.109552	2.254616	-1.856985
20	9	0	-0.881232	2.308164	-0.340675
21	9	0	0.588279	-2.023872	0.015015
22	9	0	2.732342	-2.554747	-1.525776
23	9	0	4.749791	-0.751167	-1.704826
24	9	0	4.618915	1.589708	-0.342008
25	9	0	2.491019	2.133493	1.201102

20_(P_V)_method_B_DKM.log

Input orientation:

Center Number	Atomic Number	Atomic Type	Coordinates (Angstroms) X	Y	Z
1	15	0	0.045008	0.400203	1.752936
2	1	0	0.139318	1.783713	1.937717
3	8	0	0.011629	-0.420767	2.981759
4	6	0	1.464830	0.072594	0.660282
5	6	0	2.516540	0.975535	0.529428
6	6	0	1.569819	-1.128180	-0.036512
7	6	0	3.619390	0.705774	-0.264749
8	6	0	2.658177	-1.424020	-0.837349
9	6	0	3.687442	-0.498834	-0.949059
10	6	0	-1.429469	0.266398	0.688243
11	6	0	-1.737039	1.295210	-0.199119
12	6	0	-2.314238	-0.808481	0.743071
13	6	0	-2.870652	1.279618	-0.991054
14	6	0	-3.457263	-0.849210	-0.041425
15	6	0	-3.735860	0.197945	-0.906777
16	9	0	-2.088463	-1.843731	1.546523
17	9	0	-4.286666	-1.887110	0.028052
18	9	0	-4.826472	0.163140	-1.659431
19	9	0	-3.134282	2.281126	-1.826089
20	9	0	-0.912483	2.344129	-0.306400
21	9	0	0.595124	-2.036458	0.057628
22	9	0	2.728714	-2.578042	-1.494281
23	9	0	4.738096	-0.768137	-1.710468
24	9	0	4.610140	1.586721	-0.372427
25	9	0	2.493190	2.142651	1.178370

20_(P_V)_method_B_DMSO.log

Input orientation:

Center Number	Atomic Number	Atomic Type	Coordinates (Angstroms) X	Y	Z
1	15	0	0.039515	0.301954	1.768459
2	1	0	0.132952	1.670792	2.038908
3	8	0	0.001525	-0.593267	2.946048
4	6	0	1.463203	0.038177	0.665189
5	6	0	2.507536	0.954583	0.575720
6	6	0	1.575302	-1.126023	-0.089992
7	6	0	3.609370	0.732382	-0.234216
8	6	0	2.662839	-1.373926	-0.907393
9	6	0	3.684318	-0.436343	-0.976992
10	6	0	-1.430767	0.229525	0.693499
11	6	0	-1.740967	1.311540	-0.127298
12	6	0	-2.308586	-0.851928	0.673421
13	6	0	-2.870067	1.338675	-0.925218
14	6	0	-3.446450	-0.850408	-0.118739
15	6	0	-3.727742	0.248186	-0.916436
16	9	0	-2.080635	-1.936076	1.411170
17	9	0	-4.268885	-1.896780	-0.120767
18	9	0	-4.813928	0.254339	-1.676610
19	9	0	-3.136617	2.390323	-1.695435
20	9	0	-0.924355	2.370885	-0.162156
21	9	0	0.607646	-2.045669	-0.036883
22	9	0	2.740215	-2.494456	-1.619817
23	9	0	4.734668	-0.659980	-1.753795
24	9	0	4.593446	1.625254	-0.301212
25	9	0	2.478151	2.088490	1.280856

20_(P_V)_method_B_MeOH.log

Input orientation:

Center Number	Atomic Number	Atomic Type	Coordinates (Angstroms) X	Y	Z
1	15	0	0.039550	0.302207	1.767917
2	1	0	0.132553	1.671314	2.037467
3	8	0	0.002074	-0.592259	2.945879
4	6	0	1.463195	0.038633	0.664378
5	6	0	2.508516	0.954119	0.576994
6	6	0	1.574756	-1.124712	-0.092214
7	6	0	3.610872	0.731910	-0.232254
8	6	0	2.662794	-1.372611	-0.909023
9	6	0	3.685303	-0.435964	-0.976476
10	6	0	-1.430909	0.229153	0.693082
11	6	0	-1.740826	1.310430	-0.128778
12	6	0	-2.309338	-0.851865	0.674639
13	6	0	-2.870301	1.337470	-0.926183
14	6	0	-3.447657	-0.850425	-0.116964
15	6	0	-3.728673	0.247497	-0.915723
16	9	0	-2.081594	-1.935254	1.413299
17	9	0	-4.270726	-1.896230	-0.117486
18	9	0	-4.815241	0.253516	-1.675308
19	9	0	-3.136539	2.388413	-1.697433
20	9	0	-0.923469	2.369227	-0.165148
21	9	0	0.606201	-2.043461	-0.041180
22	9	0	2.739659	-2.492219	-1.622884
23	9	0	4.736079	-0.659625	-1.752657
24	9	0	4.595844	1.623882	-0.297269
25	9	0	2.479565	2.087100	1.283637

20_(P_V)_method_B_THF.log

Input orientation:

Center Number	Atomic Number	Atomic Type	Coordinates (Angstroms) X	Y	Z
1	15	0	0.044883	0.402043	1.743136
2	1	0	0.138735	1.785825	1.927217
3	8	0	0.011677	-0.418634	2.971740
4	6	0	1.464925	0.075284	0.650311
5	6	0	2.517198	0.977939	0.521446
6	6	0	1.569830	-1.124731	-0.047895
7	6	0	3.620745	0.708495	-0.271991
8	6	0	2.658896	-1.420247	-0.848070
9	6	0	3.688820	-0.495435	-0.957660
10	6	0	-1.429551	0.268437	0.678225
11	6	0	-1.736327	1.296721	-0.210116
12	6	0	-2.315436	-0.805534	0.734567
13	6	0	-2.870459	1.281696	-1.001436
14	6	0	-3.459054	-0.845670	-0.049297
15	6	0	-3.736912	0.201027	-0.915577
16	9	0	-2.090184	-1.840096	1.538625
17	9	0	-4.289541	-1.882432	0.021529
18	9	0	-4.827876	0.166762	-1.667590
19	9	0	-3.133299	2.282563	-1.837392
20	9	0	-0.910493	2.344455	-0.319033
21	9	0	0.594444	-2.032323	0.043887
22	9	0	2.729377	-2.573326	-1.506463
23	9	0	4.739973	-0.764318	-1.718398
24	9	0	4.611983	1.588991	-0.377778
25	9	0	2.493777	2.144248	1.171660

20_(P_V)_method_B_toluene.log

Input orientation:

Center Number	Atomic Number	Atomic Type	Coordinates (Angstroms) X	Y	Z
1	15	0	0.046036	0.396941	1.748093
2	1	0	0.138354	1.783425	1.922066
3	8	0	0.017472	-0.416921	2.977344
4	6	0	1.463717	0.073890	0.647944
5	6	0	2.521470	0.971985	0.532822
6	6	0	1.565270	-1.120012	-0.061364
7	6	0	3.628123	0.705164	-0.257842
8	6	0	2.657157	-1.413323	-0.859739
9	6	0	3.693052	-0.492948	-0.954978
10	6	0	-1.430011	0.262605	0.682569
11	6	0	-1.730597	1.284309	-0.215249
12	6	0	-2.323441	-0.805001	0.752106
13	6	0	-2.866413	1.271574	-1.004535
14	6	0	-3.469858	-0.842798	-0.029566
15	6	0	-3.741398	0.198105	-0.905531
16	9	0	-2.103596	-1.831763	1.563646
17	9	0	-4.308068	-1.871246	0.052034
18	9	0	-4.834016	0.165541	-1.654492
19	9	0	-3.122997	2.265864	-1.849561
20	9	0	-0.894990	2.324131	-0.335940
21	9	0	0.584898	-2.022527	0.015144
22	9	0	2.724268	-2.558995	-1.529973
23	9	0	4.745918	-0.759881	-1.713330
24	9	0	4.623628	1.581186	-0.350605
25	9	0	2.500212	2.131614	1.194952

20_(P_V)_method_B_water.log

Input orientation:

Center Number	Atomic Number	Atomic Type	Coordinates (Angstroms) X	Y	Z
1	15	0	0.044657	0.401852	1.747603
2	1	0	0.142051	1.783369	1.940856
3	8	0	0.007304	-0.425952	2.973744
4	6	0	1.464966	0.072643	0.657759
5	6	0	2.509718	0.981127	0.511227
6	6	0	1.573311	-1.133754	-0.028582
7	6	0	3.608294	0.710869	-0.288429
8	6	0	2.657510	-1.430049	-0.834122
9	6	0	3.679479	-0.499206	-0.962045
10	6	0	-1.428258	0.272461	0.681719
11	6	0	-1.737934	1.307341	-0.197914
12	6	0	-2.308241	-0.806551	0.723940
13	6	0	-2.868392	1.291838	-0.994171
14	6	0	-3.447489	-0.847254	-0.065087
15	6	0	-3.728109	0.205337	-0.922733
16	9	0	-2.081163	-1.848079	1.521218
17	9	0	-4.271967	-1.890491	-0.006906
18	9	0	-4.815686	0.170972	-1.680240
19	9	0	-3.134401	2.299173	-1.821742
20	9	0	-0.919424	2.361427	-0.293549
21	9	0	0.604999	-2.047701	0.081685
22	9	0	2.731320	-2.590365	-1.480240

23	9	0	4.726768	-0.768899	-1.728360
24	9	0	4.592914	1.597305	-0.410998
25	9	0	2.483905	2.154329	1.149015

20_(P_V)_method_D_DCM.log

Input orientation:

Center Number	Atomic Number	Atomic Type	Coordinates (Angstroms) X	Y	Z
1	15	0	0.045817	0.404095	1.818799
2	1	0	0.130955	1.790234	2.018516
3	8	0	0.022516	-0.443278	3.054922
4	6	0	1.447885	0.094905	0.687710
5	6	0	2.507894	0.996277	0.563631
6	6	0	1.519829	-1.081955	-0.062485
7	6	0	3.587447	0.750400	-0.277613
8	6	0	2.585041	-1.353245	-0.910894
9	6	0	3.623358	-0.429593	-1.015937
10	6	0	-1.419628	0.254968	0.732658
11	6	0	-1.686791	1.254885	-0.206632
12	6	0	-2.313384	-0.817716	0.793822
13	6	0	-2.791370	1.213072	-1.046098
14	6	0	-3.428778	-0.883377	-0.036917
15	6	0	-3.667788	0.134063	-0.956021
16	9	0	-2.121155	-1.829873	1.648716
17	9	0	-4.271472	-1.922087	0.039448
18	9	0	-4.735219	0.074134	-1.755728
19	9	0	-3.017652	2.189919	-1.934473
20	9	0	-0.842164	2.299105	-0.319871
21	9	0	0.530955	-1.988763	0.024067
22	9	0	2.624520	-2.487034	-1.622426
23	9	0	4.655143	-0.677755	-1.825239
24	9	0	4.589437	1.633133	-0.380222
25	9	0	2.509391	2.142755	1.266411

20_(P_V)_method_E_DCM.log

Input orientation:

Center Number	Atomic Number	Atomic Type	Coordinates (Angstroms) X	Y	Z
1	15	0	0.044743	0.325908	1.832916
2	1	0	0.133593	1.699919	2.076530
3	8	0	0.020850	-0.543848	3.019141
4	6	0	1.436906	0.053309	0.707237
5	6	0	2.438691	0.996458	0.529507
6	6	0	1.543910	-1.135220	-0.002044
7	6	0	3.504232	0.774512	-0.323422
8	6	0	2.596299	-1.384523	-0.859632
9	6	0	3.580081	-0.421014	-1.016828
10	6	0	-1.407282	0.226030	0.749536
11	6	0	-1.601611	1.214680	-0.206109
12	6	0	-2.354100	-0.786455	0.821741
13	6	0	-2.689316	1.220860	-1.054681
14	6	0	-3.455189	-0.804025	-0.017639
15	6	0	-3.622343	0.201824	-0.952917
16	9	0	-2.234628	-1.775673	1.688033
17	9	0	-4.346408	-1.776210	0.067101
18	9	0	-4.668303	0.188172	-1.752587
19	9	0	-2.846547	2.177431	-1.952386
20	9	0	-0.709893	2.194231	-0.321575
21	9	0	0.609790	-2.067007	0.132522
22	9	0	2.674008	-2.522708	-1.525451
23	9	0	4.590456	-0.644598	-1.830378
24	9	0	4.446153	1.687880	-0.478755
25	9	0	2.400438	2.151856	1.179012

20_(P_V)_method_E_DCM_smd.log

Input orientation:

Center Number	Atomic Number	Atomic Type	Coordinates (Angstroms) X	Y	Z
1	15	0	0.020082	-0.225940	1.857723
2	1	0	0.139376	1.008235	2.503090
3	8	0	-0.052438	-1.410016	2.728612
4	6	0	1.425535	-0.198905	0.714002
5	6	0	2.446844	0.733292	0.816455
6	6	0	1.516405	-1.133914	-0.307355
7	6	0	3.512881	0.744931	-0.063352
8	6	0	2.568787	-1.147563	-1.199030
9	6	0	3.571034	-0.200262	-1.071511
10	6	0	-1.412307	0.042785	0.773753
11	6	0	-1.585231	1.284704	0.178017
12	6	0	-2.360682	-0.931725	0.496526
13	6	0	-2.651857	1.567077	-0.649299
14	6	0	-3.440533	-0.674556	-0.329967
15	6	0	-3.585927	0.576802	-0.899968
16	9	0	-2.260059	-2.148673	1.003008
17	9	0	-4.332576	-1.618609	-0.581041
18	9	0	-4.612505	0.825470	-1.688185
19	9	0	-2.786989	2.762108	-1.200144
20	9	0	-0.693309	2.247135	0.395622
21	9	0	0.563366	-2.047086	-0.448289
22	9	0	2.627435	-2.047002	-2.166435
23	9	0	4.582274	-0.198341	-1.916296
24	9	0	4.472910	1.647518	0.050816
25	9	0	2.428769	1.653848	1.771793

Supplementary references

S1 Becke, A. D. Density-functional thermochemistry. III. The role of exact exchange. *J. Chem. Phys.* **1993**, *98,* 5648−5652, doi:10.1063/1.464913.

S2 Stephens, P.; Devlin, F.; Chabalowski, C. F.; Frisch, M. J. Ab initio calculation of vibrational absorption and circular dichroism spectra using density functional force fields. *J. Phys. Chem.* **1994**, *98*, 11623−11627, doi:10.1021/j100096a001.

S3 Petersson, G. A.; Bennett, A.; Tensfeldt, T. G.; Al- Laham, M. A.; Shirley, W. A. A complete basis set model chemistry. I. The total energies of closed- shell atoms and hydrides of the first- row elements. J. Chem. Phys. 1988, 89, 2193−2218, doi: 10.1063/1.455064.

S4 Becke, A. D. Perspective: Fifty years of density-functional theory in chemical physics. *J. Chem. Phys*. **2014**, *140*, 18A301−18A301, doi:10.1063/1.4869598.

S5 McLean, A. D.; Chandler, G. S. Contracted Gaussian basis sets for molecular calculations. I. Second row atoms, Z=11-18. *J. Chem. Phys.* **1980**, *72*, 5639−5648, doi:10.1063/1.438980.

S6 Woon, D. E.; Dunning Jr., T. H. Gaussian-basis sets for use in correlated molecular calculations. 3. The atoms aluminum through argon. *J. Chem. Phys.* **1993**, *98,* 1358−1371, doi: 10.1063/1.464303.

S7 Grimme, S.; Antony, J.; Ehrlich, S.; Krieg, H. A consistent and accurate ab initio parametrization of density functional dispersion correction (DFT-D) for the 94 elements H-Pu. *J.Chem. Phys.* **2010**, *132*, 154104 doi:10.1063/1.3382344.

S8 Chai, J-D.; Head-Gordon, M. Long-range corrected hybrid density functionals with damped atom-atom dispersion corrections, *Phys. Chem. Chem. Phys.* **2008**, *10,* 6615−6620, doi:10.1039/B810189B.

S9 Frisch, M. J.; Trucks, G. W.; Schlegel, H. B.; Scuseria, G. E.; Robb, M. A.; Cheeseman, J. R.; Scalmani, G.; Barone, V.; Mennucci, B.; Petersson, G. A.; Nakatsuji, H.; Caricato, M.; Li, X.; Hratchian, H. P.; Izmaylov, A. F.; Bloino, J.; Zheng, G.; Sonnenberg, J. L.; Hada, M.; Ehara, M.; Toyota, K.; Fukuda, R.; Hasegawa, J.; Ishida, M.; Nakajima, T.; Honda, Y.; Kitao, O.; Nakai, H.; Vreven, T.; Montgomery, J. A., Jr.; Peralta, J. E.; Ogliaro, F.; Bearpark, M.; Heyd, J. J.; Brothers, E.; Kudin, K. N.; Staroverov, V. N.; Kobayashi, R.; Normand, J.; Raghavachari, K.; Rendell, A.; Burant, J. C.; Iyengar, S. S.; Tomasi, J.; Cossi, M.; Rega, N.; Millam, J. M.; Klene, M.; Knox, J. E.; Cross, J. B.; Bakken, V.; Adamo, C.; Jaramillo, J.; Gomperts, R.; Stratmann, R. E.; Yazyev, O.; Austin, A. J.; Cammi, R.; Pomelli, C.; Ochterski, J. W.; Martin, R. L.; Morokuma, K.; Zakrzewski, V. G.; Voth, G. A.; Salvador, P.; Dannenberg, J. J.; Dapprich, S.; Daniels, A. D.; Ö. Farkas, Foresman, J. B.; Ortiz, J. V.; Cioslowski, J.; Fox, D. J. Gaussian 09, revision D.01; Gaussian, Inc.: Wallingford, CT, 2009

S10 Miertuš, S.; Scrocco, E.; Tomasi, J. Electrostatic interaction of a solute with a continuum. A direct utilizaion of AB initio molecular potentials for the prevision of solvent effects. *J. Chem. Phys.* **1981**, *55*, 117–129, doi:10.1016/0301-0104(81)85090-2.

S11 Marenich, A. V.; Cramer, C. J.; Truhlar, D. G. Universal solvation model based on solute electron density and on a continuum model of the solvent defined by the bulk dielectric constant and atomic surface tensions. *J. Phys. Chem. B* **2009**, *113*, 6378−6396, doi:10.1021/jp810292n.

S12 Glendening, E. D.; Reed, A. E.; Carpenter, J. E.; Weinhold, F. NBO Version 3.1.

S13 Foster, J. P.; Weinhold, F. Natural hybrid orbitals. *J. Am. Chem. Soc.* **1980**, *102*, 7211−7218, doi:10.1021/ja00544a007.

S14 Hratchian, H. P.; Schlegel, H. B. Accurate reaction paths using a Hessian based predictor-corrector integrator. *J. Chem. Phys*. **2004**, *120*, 9918−9924, doi: 10.1063/1.1724823.
